# The Patient, Investigator, Nurse, Carer Questionnaire (PINC-Q): a cross-sectional, retrospective, non-interventional study exploring the impact of less frequent medication administration with paliperidone palmitate 3-monthly as maintenance treatment for schizophrenia

**DOI:** 10.1186/s12888-021-03305-z

**Published:** 2021-06-09

**Authors:** Katalin Pungor, Pedro Sanchez, Sofia Pappa, Jerome Attal, Karolina Leopold, Geertje Steegen, Antonio Vita, Carol Marsella, Caroline Verrijcken, Marjolein Lahaye, Annette Wooller

**Affiliations:** 1 Janssen-Cilag, Medical Affairs EMEA, Dusseldorf, Germany; 2 Treatment Resistant Psychosis Unit, Hospital Psiquiatrico de Alava, Osakidetza, Vitoria, Spain; 3 West London NHS Trust, London, UK; 4 Academic Department of Adult Psychiatry, Hospital La Colombière, CHU Montpellier, Montpellier, France; 5 Department of Psychiatry, Psychotherapy and Psychosomatic Medicine with Early Intervention and Recognition Centre (FRITZ), Vivantes Klinikum Am Urban and Klinikum im Friedrichshain, Teaching Hospital of Charité-Universitätsmedizin, Berlin, Germany; 6 Psychiatry Department, CHU Brugmann, Brussels, Belgium; 7 Department of Clinical and Experimental Sciences, University of Brescia, Brescia, Italy; 8 Janssen-Cilag, Medical Affairs EMEA, Zug, Switzerland; 9 Janssen R&D BE, Global Trial Management, Antwerp, Belgium; 10 Janssen-Cilag BV, Statistics & Decision Sciences, Breda, The Netherlands; 11 Janssen-Cilag, Medical Affairs EMEA, High Wycombe, UK

**Keywords:** Carer, Cross-sectional survey, Long-acting antipsychotic, Nurse, Paliperidone palmitate 3-monthly, Real world practice, Schizophrenia

## Abstract

**Background:**

To understand the implications of switching from paliperidone palmitate 1-monthly (PP1M) to paliperidone palmitate 3-monthly (PP3M) treatment of schizophrenia from the perspective of four key stakeholders: patients, physicians, nurses and carers.

**Methods:**

This was a cross-sectional, retrospective, non-interventional study comprising a one-time questionnaire (PINC-Q) for adult patients (aged ≥18 years) with schizophrenia (International Classification of Diseases; ICD-10) and their physician, nurse and carer. Questionnaires were developed in association with patient and carer advocacy groups (GAMIAN and EUFAMI) and following an advisory board formed of psychiatrists and nurses. The degree of alignment between stakeholders was also examined.

**Results:**

Responses were received from a total of 224 evaluable patients. For most patients (88.4%), responses were received from at least two other stakeholders. Patients were moderately ill with mild-to-moderate lack of insight and had received PP1M for a mean (standard deviation [SD]) of 23.9 (21.28) months before switching to PP3M (duration mean [SD] 12.8 [3.72] months). The most frequently reported reasons to switch from PP1M to PP3M were ‘to live life as normally as possible’ and ‘patient convenience’. Over 79% of responses within each stakeholder group stated that PP3M helped the patients, with increased patient activity and social involvement, improved frequency and quality of physician–patient and nurse–patient communication and decreased perceived stigma.

**Conclusions:**

The results of this study add to the increasing body of evidence supporting the benefits of PP3M in a population of patients with schizophrenia representative of real-world clinical practice.

**Supplementary Information:**

The online version contains supplementary material available at 10.1186/s12888-021-03305-z.

## Background

Non-adherence to antipsychotic treatment is common in patients with schizophrenia [1, 2]. Lack of continuous maintenance treatment can put the patient at risk of relapse, as well as increased treatment resistance, residual symptom severity, cerebral toxicity, cognitive decline, functional impairment and premature mortality [3–7]. Long-acting injectable antipsychotic treatments (LATs) have been developed to overcome the need for daily dosing of oral antipsychotic medication and are a valuable treatment option to enhance treatment continuation [8, 9]. LATs not only reduce dose administration frequency versus oral antipsychotics, but also offer reliable medication delivery, less fluctuation in plasma levels and transparency of dose administration compared with oral antipsychotics, aiding treatment continuation [8, 9].

Paliperidone palmitate 3-monthly (PP3M) is a LAT formulation approved for maintenance treatment of schizophrenia in patients previously stabilised with paliperidone palmitate 1-monthly (PP1M) [10, 11] and requires only four administrations per year. The efficacy and safety of PP3M have been established in two pivotal phase 3 studies [12, 13]. PP3M demonstrated favourable clinical outcomes, including delayed time to relapse, symptomatic remission and functional recovery [12, 13]. More recent naturalistic studies have demonstrated the efficacy of PP3M in terms of achieving remission [14]; however, the real impact of PP3M for patients and, consequently, for physicians, nurses and carers may not be fully understood.

The objective of the present study was to explore the experience of PP3M treatment in patients with schizophrenia and their corresponding physicians, nurses and carers to understand a more complete view of the qualitative impact of less frequent administration. The study was also designed to investigate the degree of alignment between these stakeholders at an individual patient level.

## Methods

This was a cross-sectional, retrospective, non-interventional study conducted in 37 centres across seven countries. Patients and their physician, nurse and carer (where applicable) each completed a one-time questionnaire (Patient, Investigator, Nurse, Carer Questionnaire [PINC-Q]).

The questionnaires were developed specifically for this study, with input from an advisory board of physicians and nurses, and in collaboration with representatives from Global Alliance of Mental Illness Advocacy Networks (GAMIAN) Europe and European Families Affected by Mental Health (EUFAMI).

Input from these groups was critical in ensuring that the questionnaires were designed and worded appropriately, considering potential variation in healthcare and cultural differences between countries. Questionnaires assessed the same topics across all stakeholders, but language was tailored as appropriate to ensure that each point was examined consistently. All questionnaires were translated into local language.

The study was conducted in accordance with the Declaration of Helsinki and was approved by all relevant institutional ethics committees. All participating patients and carers provided written informed consent prior to taking part in the study.

Physicians at participating centres offered enrolment to all eligible patients aged ≥18 years with a diagnosis of schizophrenia (according to International Classification of Diseases; ICD-10) who were currently receiving PP3M, previously received 4–6 PP3M administrations (i.e. approximately 9 to 15 months of PP3M treatment, thereby ensuring that PP3M treatment was well established prior to the questionnaire and to allow sufficient time to assess its impact), and were capable and willing to participate. Patients were excluded if they had received involuntary treatment with PP3M or were switched to PP3M during a clinical trial. Patients were evaluable if they, and at least one other associated stakeholder, completed the questionnaire.

Questionnaires were completed separately by patients and their physician, nurse and/or carer at a single data collection point. After receiving guidance on questionnaire completion, the questionnaire was completed by patients at the study site during a routine clinic visit and by carers at the same visit or within 2 weeks after the patient’s visit.

Physician questionnaires included an assessment of the patient’s level of illness for correlation with questionnaire responses. Level of illness was assessed using the Clinical Global Impression-Severity (CGI-S) scale and the Positive and Negative Syndrome Scale (PANSS) item G12 (lack of judgement and insight). Patients reported their level of illness using the European Quality of Life-5 dimension-5 level (EQ-5D-5L) questionnaire (patient-reported general health status), consisting of the EQ-5D-5L descriptive system and the EQ visual analogue scale [15].

Data from each question were summarised separately for patients, physicians, nurses and carers; questionnaire items were presented descriptively using frequency and percentages for categorical data, as well as count, mean, standard deviation (SD) and minimum and maximum for ordinal responses. The following categories were used in the questionnaire:

- Impact on relationship/interaction between medical team and carer/patient: 13 items
- Involvement in treatment decision: two items
- Reasons for selecting PP3M: two items
- Impact on patient: five items
- Impact on carer: eight items
- Treatment experience with PP3M: one item

There was no imputation of missing data for the questionnaires.

Inter-rater alignment assesses the level of agreement between stakeholders when answering questions about a specific patient. The degree of inter-rater alignment of responses across the stakeholders overall and for individual pairings was examined using Cohen’s (weighted) kappa for categorical data and Lin’s concordance correlation coefficient (CCC) for continuous/ordinal data.

Spearman’s correlation was used to explore correlations between patients’ level of judgement and insight (PANSS item G12) and their level of illness (CGI-S and EQ-5D-5L) and the number of years since their schizophrenia diagnosis. Furthermore, associations between patients’ level of judgement/insight and treatment experience with PP3M and the change in carer time required for patient support were explored using the Chi-square test.

## Results

### Patient characteristics

In total, 228 patients were enrolled, of whom 227 were considered eligible for study participation. The evaluable cohort for analysis consisted of 224 patients who had a patient-completed questionnaire item along with an item completed by at least one other respondent (physician, nurse or carer). Three patients were not included in the analysis either due to missing patient data (*n* = 2) or to lack of other stakeholder data (*n* = 1).

**Fig. 1:**
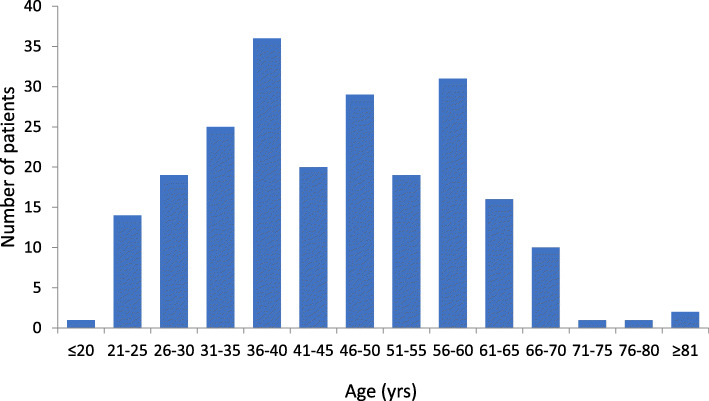
Age distribution of patients included in the study

Patient demographics and disease and concomitant treatment characteristics are presented in Table 1 and Fig. 1. Overall wellbeing of patients was high, and patients’ lack of insight was mild to moderate. Patients had been treated with PP1M for a mean (SD) of 23.9 (21.28) months before switching to PP3M treatment. Patients had received PP3M for a mean (SD) of 12.8 (3.72) months. At the time of questionnaire completion, patients were most frequently receiving PP3M 525 mg eq. (40.5%) or PP3M 350 mg eq. (32.4%).

For most evaluable patients (198/224; 88.4%), questionnaires were obtained from at least three different stakeholders; 26 patients (11.6%) had questionnaires completed by two stakeholders.

Alignment of responses was assessed overall and for paired groupings of stakeholders. For many of the results, there was limited inter-rater alignment (pairwise and overall agreement) across the different stakeholders; however, some parameters demonstrated moderate or higher inter-rater alignment. Full details of inter-rater alignment for all responses are available in Additional file 2.

### Other stakeholders

A total of 29 physicians and 28 nurses were included in the analysis; some completed questionnaires for multiple patients, therefore, results are presented as the number of responses rather than number of respondents.

Approximately 90% of physicians and 68% of nurses had > 10 years’ experience in the psychiatric setting and saw a mean (SD) of 62.8 (34.94) patients and 71.3 (73.18) patients with schizophrenia per month, respectively.

Of the 224 patients in the study, 112 patients reported that they had a carer available to participate. A total of 100 carers completed the demographic questionnaire, of whom 98 completed the question asking whether they are a carer for one patient or > 1 patient: 86 carers (87.8%) were caring for one patient and 12 carers (12.2%) were caring for > 1 patient. The results are presented as the number of responses. Carers were most frequently a relative of the patient (69.7%), either a parent (27.3%), child (21.2%) or sibling (21.2%), and 49% of carers lived with the patient.

**Table 1:** Patient demographics, and disease and concomitant treatment characteristics

Characteristic	Total evaluable patients (***N*** = 224)
Age, years, mean (SD)	45.3 (13.45)
Males, n (%)	160 (71.4)
Family status, single, n (%); *n* = 222	144 (64.9)
Living arrangement, n (%)	
Alone	65 (29.0)
With family (parents/siblings)	97 (43.3)
With partner/children	42 (18.8)
Other	20 (8.8)
In education/employment, yes, n (%)	54 (24.3)^a^
Student	7 (3.2)
Employed (paid, voluntary, self-employed)	46 (20.8)
Country, n (%)	
Belgium	4 (1.8)
France	63 (28.1)
Germany	6 (2.7)
Hungary	23 (10.3)
Italy	37 (16.5)
Spain	84 (37.5)
United Kingdom	7 (3.1)
Patients receiving other non-pharmacological treatments or care currently, yes (%); *n* = 218	96 (44.0)
Years living with schizophrenia diagnosis, mean (SD); *n* = 214	12.0 (10.15)
Years taking antipsychotic medication, mean (SD); *n* = 211	12.1 (9.69)
EQ-5D-5L VAS, mean (SD); *n* = 190^b^	70.8 (19.60)
CGI-Severity score, mean (SD); *n* = 221	3.7 (0.99)
CGI-Severity categories,^c^ n (%); *n* = 221	
Normal, not at all ill	5 (2.3)
Borderline mentally ill	17 (7.7)
Mildly ill	60 (27.1)
Moderately ill	101 (45.7)
Markedly ill	31 (14.0)
Severely ill	7 (3.2)
PANSS item G12 lack of judgement and insight, mean (SD); *n* = 212	3.2 (1.30)
PANSS item G12 lack of judgement and insight categories, n (%);^d^ *n* = 212	
Absent	19 (9.0)
Minimal	38 (17.9)
Mild	77 (36.3)
Moderate	44 (20.8)
Moderate severe	20 (9.4)
Severe	13 (6.1)
Extreme	1 (0.5)
Current psychiatric comorbidities, yes, n (%);^e^ *n* = 222	59 (26.6)
Other concomitant psychotropic treatments currently prescribed in addition to PP3M, yes, n (%);^f^ *n* = 221	139 (62.9)
Oral antipsychotic	78 (35.3)
Anxiolytic/hypnotic	77 (34.8)
Anticholinergic	18 (8.1)
Antidepressant	47 (21.3)
Mood stabiliser	25 (11.3)

^a^One patient was confirmed to be either in employment or education; however, this was not further specified; ^b^Patients recorded their own assessment of their overall health status on a scale of 0 (worst health) to 100 (best health) [15]; ^c^Severity of patient’s psychotic condition at a particular time on a 7-point scale ranging from 1 (normal, not at all ill) to 7 (among the most extremely ill patients) [16]; ^d^A scale of 1 (absent) to 7 (extreme) [17]; ^e^Patients may have had more than one comorbidity; ^f^Patients may have been taking more than one medication. CGI, Clinical Global Impression; EQ-5D-5L, EuroQoL-5D; PANSS, Positive and Negative Syndrome Scale; PP3M, paliperidone palmitate 3-monthly; SD, standard deviation; VAS, visual analogue scale

### Changes in patient, carer and medical team experiences following switch to PP3M

**Fig. 2:**
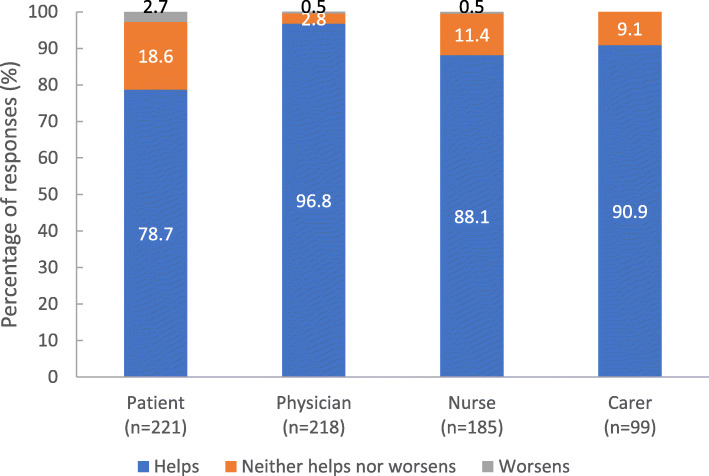
Changes in patient, physician, nurse and carer experiences as a result of switch to PP3M. footnote: Stakeholders were asked ‘To what extent do you feel the PP3M treatment is helping the patient?’. PP3M, paliperidone palmitate 3-monthly

The majority of responses (79–97%) from all stakeholder groups indicated that PP3M helped the patient (Fig. 2).

**Fig. 3:**
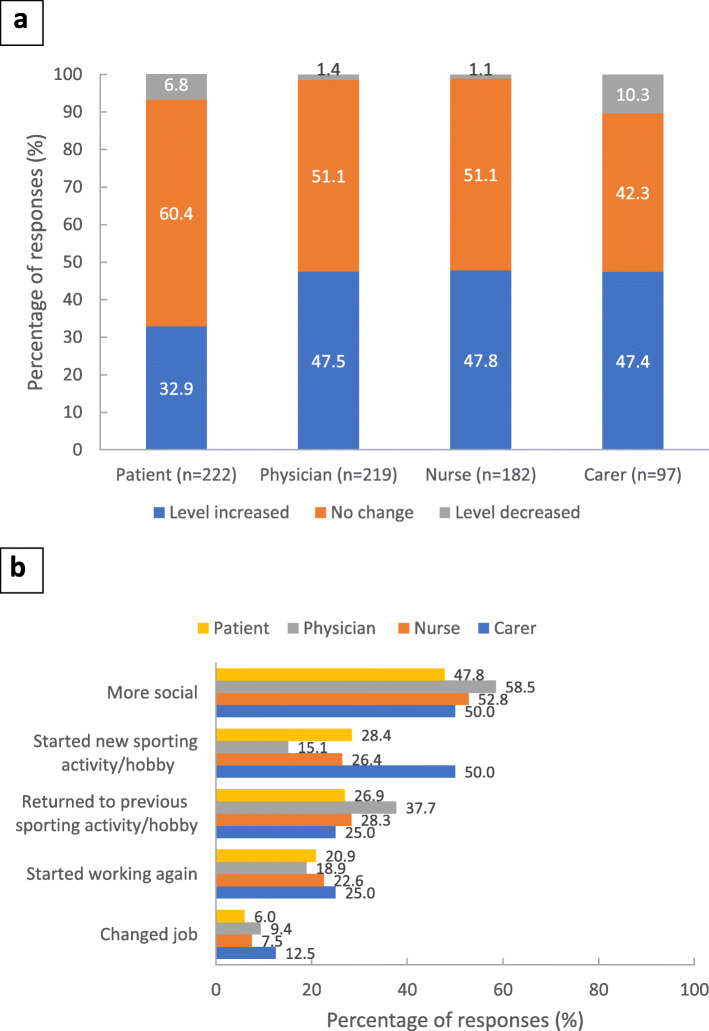
Patient activity. Footnote: **a** Change in patient activity levels as a result of the switch to PP3M. Stakeholders were asked ‘Has the patient’s activity level changed as a result of switching to PP3M (e.g. sports, hobbies, education, work, seeing friends and/or family)?’. **b** Type of activity changes following switch to PP3M: proportion of patients who answered ‘yes’ to positive changes. Stakeholders were asked ‘Please indicate in what way patient’s activity was increased as a result of switching to PP3M?’ Respondents could choose multiple answers from a predefined list. PP3M, paliperidone palmitate 3-monthly

Change in activity levels: Almost all responses indicated either an increase or no change in patient activity levels (Fig. 3a), with increased sociability and returning to previous sports and hobbies as the most commonly reported positive changes in activity levels (Fig. 3b).

**Fig. 4:**
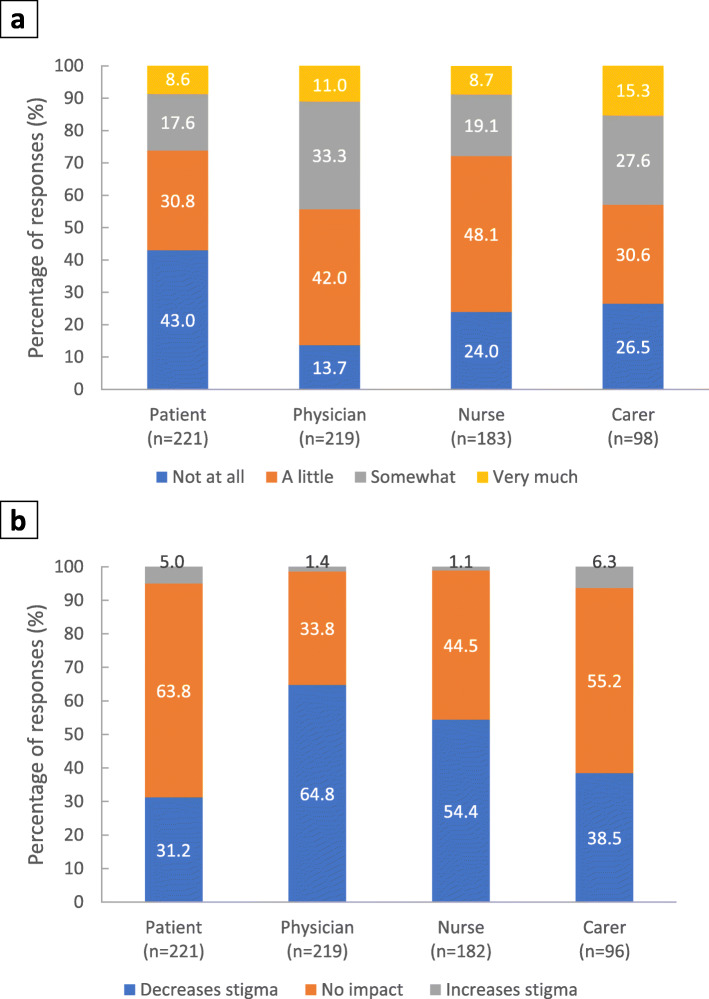
Perceptions of stigmatisation and impact of switch to PP3M on stigma. Footnote: **a** Stigma related to the diagnosis of schizophrenia. Stakeholders were asked ‘How stigmatised do you feel the patient is?’. **b** Impact of switch from PP1M to PP3M on stigma. Stakeholders were asked ‘To what extent do you think that PP3M impacts on the patient’s feeling of being stigmatised?’. PP3M, paliperidone palmitate 3-monthly

Impact on stigma: Physicians, nurses and carers most frequently reported that the patients with schizophrenia were stigmatised due to their diagnosis (Fig. 4a). Responses from patients most frequently indicated that they experienced at least some stigma, although almost half of patients reported experiencing no stigma at all. Following PP3M treatment, physicians and nurses most frequently reported a decrease in the patient’s feeling of being stigmatised, whereas patients and carers predominantly reported no change in the patient’s feeling of stigmatisation; however, around one-third reported a decrease in feeling of stigmatisation (Fig. 4b).

#### Changes in communication between patients, carers and the medical team

**Fig. 5:**
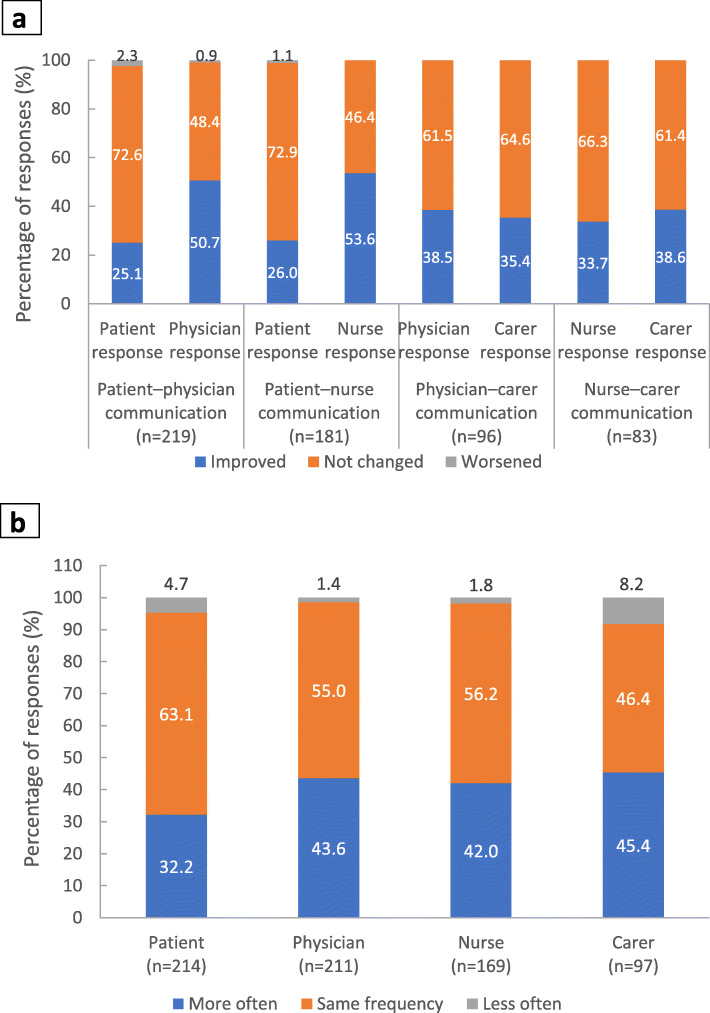
Communication quality and frequency. Footnote: **a** Changes in communication quality following the switch from PP1M to PP3M. Stakeholders were asked ‘Has the quality of communication improved as a result of switching to PP3M?’. **b** Change in frequency of patient communication. Stakeholders were asked ‘As a result of switching to PP3M have you noticed any change in the frequency of communications between the patient and family members, friends and/or other people?’. PP3M, paliperidone palmitate 3-monthly

Half of responses from physicians indicated that the quality of physician–patient communication improved following the switch to PP3M, compared with one-quarter of responses from patients (Fig. 5a). A similar pattern was observed for nurse–patient communication, whereas perspectives on changes in communication quality were closely aligned between carers and healthcare professionals. With regard to changes in patient communication with family and others, most respondents reported either no change or an increase in the frequency of communication (Fig. 5b).

The majority of responses indicated there was either no change or an increase in discussion of non-medication topics between stakeholders following the switch to PP3M. Overall, 52.7% of responses from physicians stated that they discussed non-medication-related topics with the patient more frequently following switch to PP3M than before the switch, and 44.4 and 33.8% of responses from nurses and patients, respectively, reported more discussion. However, 51.3% of nurses and 60.8% of patients reported no change/maintained level in the discussion of non-medication-related topics; less discussion was reported in 2.7% of responses from physicians, 4.3% of responses from nurses and 5.4% of responses from patients. Physicians, nurses and patients responded that the non-medication-related topics most frequently discussed with patients were: family relationships (79.6, 70.5 and 62.7%, respectively), social communication (55.1, 54.5 and 30.5%), hobbies (51.0, 50.0, and 52.5%) and healthy lifestyle guidance (44.9, 63.6 and 45.8%).

With regard to discussions carers had with the medical team, 39.5, 31.5 and 31.3% of responses from physicians, nurses and carers, respectively, indicated an increase in the discussion of non-medication-related topics. Physicians, nurses and carers however, most commonly reported no change in the frequency of the discussion of non–medication-related topics after the patient switched to PP3M (52.9, 59.3 and 58.3% of the responses, respectively); 7.6, 9.3 and 10.4% of responses from physicians, nurses and carers, respectively, indicated a decrease in these discussions.

Family relationships were reported by physicians, nurses and carers as the most frequently discussed topic following the treatment switch (84.6, 68.8 and 68.8% of the responses, respectively).

Regarding discussion of non-medication topics between patients and carers, 36.5% of patients’ responses indicated an increase in discussions, 59.4% reported ‘no change’ and 4.2% reported less discussion. Conversely, 50.0% of carers’ responses indicated increased discussion of non-medication topics, 45.8% indicated ‘no change’ and 4.2% reported less discussion.

#### Impact on carer time

**Fig. 6:**
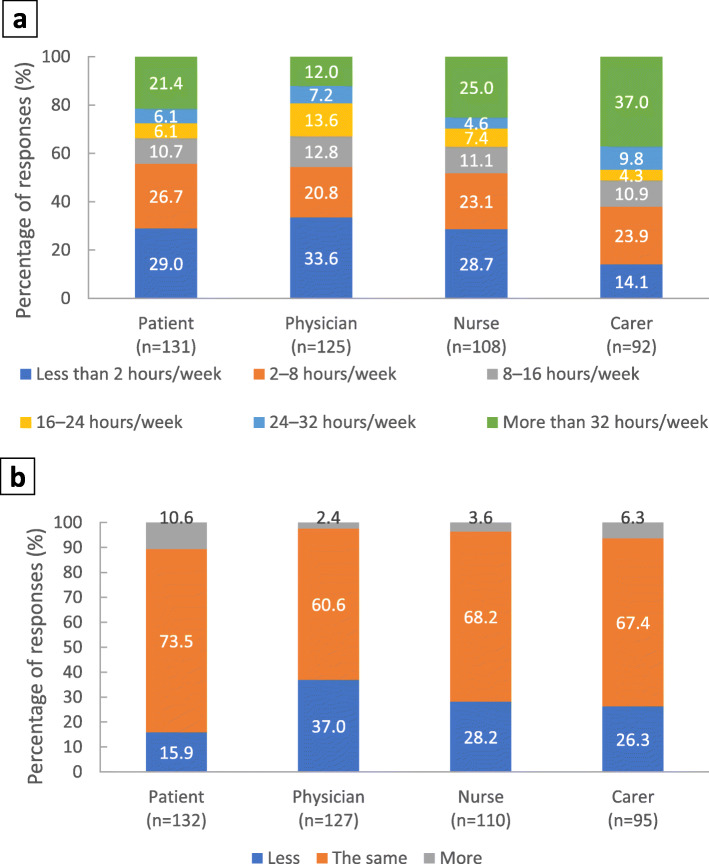
Impact on carers. 6 footnote: **a** Total hours the carer supports the patient per week. Stakeholders were asked ‘What is your estimate on the total hours per week the carer spends supporting the patient?’. **b** Change in carer time required for patient support since the switch from PP1M to PP3M. Stakeholders were asked ‘Do you feel that the time required for support from the carer is less or more than before switching to PP3M?’. PP3M, paliperidone palmitate 3-monthly

There was a notable disconnect between the number of hours the carers reported to spend supporting the patients (most frequently reported as > 32 h/week) and the number of hours recognised by the patient, physician and nurse (most frequently reported as < 2 h/week; Fig. 6a).

Whilst the amount of carer time required was underestimated by patients, physicians and nurses, there was greater alignment on the impact that switching to PP3M had on carer time. Approximately, one-quarter of responses from carers indicated that the amount of time carers spent supporting patients had decreased following the switch; however, the majority of responses from all stakeholders indicated carer time was ‘the same’ after the switch (Fig. 6b).

Similarly, while all stakeholders most frequently reported that there was ‘no change’ in the extent of support patients required from their carer(s) following the treatment switch (66.4, 54.0, 58.0 and 56.5% of responses, respectively), 27.5, 42.9, 39.3 and 41.3% of the responses, respectively, indicated that patients required less support from their carer after treatment switch; a small proportion of responses indicated an increase in the extent of carer support required by the patient after treatment switch (6.1, 3.2, 2.7 and 2.2%, respectively).

#### Decision to switch from PP1M to PP3M

Main reason to switch from PP1M to PP3M: Physicians and nurses most frequently selected ‘patient convenience’ (64.6 and 66.5% of the responses, respectively) and ‘to live life as normally as possible’ (61.9 and 59.0% of the responses, respectively) as the reasons for treatment switch. Similarly, patients and carers most frequently chose ‘to live life as normally as possible’ (65.2 and 72.0% of responses, respectively) as the main reason for the switch, but reported ‘patient convenience’ as the second most frequent reason for switching (54.0 and 49.0%, respectively).

Importance of involvement in treatment decisions: Patients, physicians and nurses most frequently considered patients as key stakeholders (64.0, 96.4, 100%, of the responses, respectively) and nurses as key stakeholders (71.4, 92.9, 80.2% of the responses, respectively) in treatment decisions. Carers most frequently considered the physician (83.0%) and patient (73.0%) as the most important stakeholders in treatment decisions. Notably, fewer patients (29.7%) responded that it was important to involve the carer in treatment decisions compared with the number of physicians (53.6%) and nurses (78.6%).

**Fig. 7:**
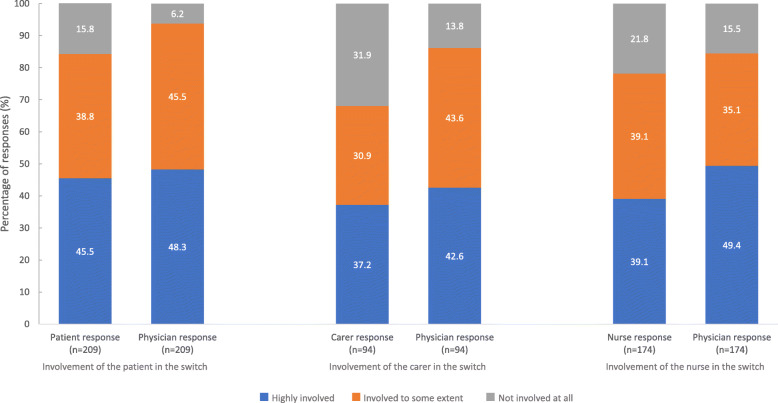
Involvement of stakeholders in the decision to switch treatment from PP1M to PP3M. footnote: PP3M, paliperidone palmitate 3-monthly

Involvement in the decision to switch from PP1M to PP3M: Physicians and patients most frequently reported that patients were ‘highly’ involved in the decision to switch treatment. Overall, the proportion of physicians that considered patients, carers and nurses to be highly involved in treatment decisions was higher than reported by the other stakeholders themselves (Fig. 7).

**Fig. 8:**
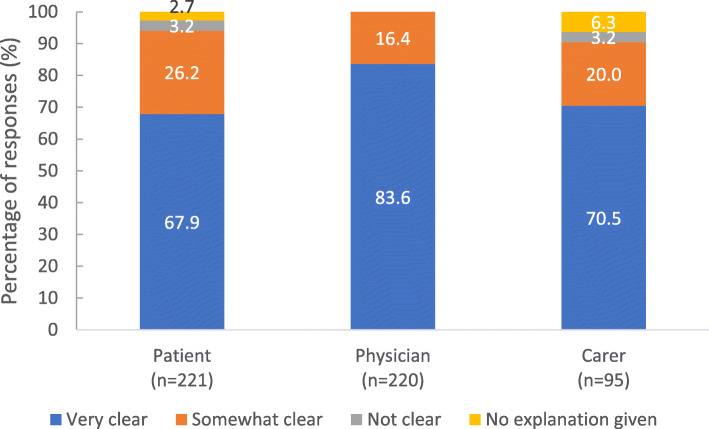
Clarity of explanation from the physician to the patient regarding the switch to PP3M. footnote: Stakeholders were asked ‘How clear was the explanation on the switch from PP1M to PP3M to the patient by his/her doctor?’. It should be noted that nurses were not asked this question. PP3M, paliperidone palmitate 3-monthly

Discussion to switch to PP3M: The majority of responses indicated that the physician initiated the discussion to switch from PP1M to PP3M (≥87.0% within each respondent group). While physicians, patients and carers most frequently responded that the explanation given by the physician was ‘very clear’, a larger proportion of physicians indicated this compared with patient and carer responses. A minority of responses from patients and carers indicated that no explanation was given (Fig. 8).

#### Frequency of encounters following switch from PP1M to PP3M

Physicians and patients reported a mean (SD) of 3.9 (4.82) and 4.0 (5.10) physician–patient encounters in the 6 months prior to the study, roughly equating to one visit every 6 weeks. Conversely, carers perceived more frequent physician–patient encounters, reporting a mean (SD) of 6.2 (15.51) encounters.

Once every 3 months was the most commonly preferred frequency of physician–patient encounters, although this frequency was opted for by fewer patients and carers (48.2 and 49.0% of responses, respectively) than by physicians and nurses (57.9 and 57.6% of responses, respectively).

Stakeholders most frequently responded that there was no change in the frequency of physician–patient, physician–carer, nurse–carer and patient–carer encounters following switch to PP3M. Overall, just over one-third of responses from physicians, patients and carers reported a decrease in the frequency of physician–patient encounters (38.7, 43.1 and 38.1%, respectively.

Most responses received from patients, physicians and carers reported that they were ‘satisfied’ or ‘very satisfied’ with the current frequency of physician–patient visits (91.9, 93.7 and 94.0%, respectively).

#### Patient encounters with carers in the 2 months prior to the study

For patients and carers who did not live together, the mean (SD) number of encounters in the last 2 months reported by patients and carers was similar and equated to contact every other day. Patients and carers most frequently reported no change in the frequency of patient–carer encounters (59.4 and 52.2% of responses, respectively). Only 4.3% of responses from patients and carers indicated that encounters decreased, while 8.7% of responses from patients and 7.2% of responses from carers indicated that the number of encounters increased.

Of responses from patients and carers regarding the current frequency of encounters, the majority were ‘very satisfied’ or ‘satisfied’ (97.9 and 95.7%, respectively).

### Hospitalisation

In the 12 months prior to the switch, 24.9% of patients were hospitalised for psychiatric reasons, with a mean (SD) of 1.3 (0.72) hospitalisations and 67.6 (80.55) days spent hospitalised. Following switch to PP3M, 9.0% of patients were hospitalised over a treatment duration of 1–1.5 years, with a mean (SD) number of hospitalisations of 2.5 (3.00) and 63.2 (59.11) days hospitalised reported by the physicians.

### Correlations between patient judgement/insight and level of illness, and relationship between judgement and insight and whether PP3M helped reduce carer time and support

In the exploratory post hoc analysis, level of illness (as measured by CGI-S) was significantly correlated with patient’s Lack of Judgement and Insight (PANSS item G12; Spearman’s correlation 0.59; *p* < 0.0001). This substantial positive correlation possibly suggests that lower disease severity may be linked with better insight. No statistically significant correlations were observed between the patient’s level of judgement/insight and EQ-5D-5L (patient’s assessments of illness) or the number of years since schizophrenia diagnosis.

With regard to the relationship between the patient’s level of judgement/insight and treatment experience with PP3M (i.e. whether PP3M is helping the patient), poorer insight was significantly associated with reduced likelihood of the patient responding that PP3M ‘helps’ (*p* < 0.0001). However, patient judgement/insight had no significant correlation with physicians’, carers’ or nurses’ responses on patients’ treatment experience with PP3M. For change in carer time required for patient support, no statistically significant relationship with patient insight/judgement was observed for any stakeholder.

## Discussion

The outcomes of this cross-sectional study show the qualitative impact of successfully switching adult patients with schizophrenia from PP1M to PP3M on the patient as well as their physician, nurse and carer.

The majority of responses from all four stakeholders indicated that switching to PP3M helped patients, despite patients being stable on PP1M and receiving PP1M for an average of 2 years before switching to PP3M. Furthermore, a degree of improvement was seen across multiple areas of schizophrenia management, suggesting gains in addition to maintaining symptom control over time with continued medication. Improvements included increases in patient activity, improved quality of physician–patient and nurse–patient communication, increased communication between the patient and family, friends or other people, and reductions in the amount of time required for the carer to support the patient. The results of the exploratory analysis suggest that lower illness severity in this patient population was associated with better patient judgement and insight. It should however be noted that other studies have reported a persistent lack of patient judgement and insight despite a favourable treatment response in patients with schizophrenia [18]. Less frequent dosing with LATs, particularly 3-monthly administration, may be perceived by patients and carers as the patient being ‘on the road to recovery’, and associated with hope, improved social acceptability, reduced stigma and greater involvement in daily activities [19]. In the current study, stakeholders most commonly reported that the reason for switch to PP3M was to allow the patient ‘to live life as normally as possible’ (with ‘patient convenience’ also an important reason for the switch).

Encouragingly, one-third of patients and almost half of other stakeholders, reported an increase in patient activity following switch to PP3M, with social interactions being most commonly improved. This is a key benefit in patients with schizophrenia, for whom reduced social participation is associated with negative outcomes [20, 21], while social networks provide multiple benefits including reducing feelings of psychological distress, increasing engagement with mental health services and improving quality of life [20, 22]. In addition, 31–65% of stakeholders reported that switch to PP3M decreased the patient’s feeling of being stigmatised. These results, along with the finding that switch to PP3M was perceived by all stakeholders to have ‘helped the patient’ overall, indicate that the impact of PP3M addressed stakeholders’ main reasons for the treatment switch. Switching to PP3M from PP1M provides a longer interval between dosing, resulting in less frequent reminders for patients about their schizophrenia, which may account for the decrease in patients’ feeling of being stigmatised.

Promisingly, all stakeholders most frequently considered that the explanation given by the physician regarding the switch to PP3M was ‘very clear’ and very few reported that no explanation was given at all. However, it should be noted that a larger proportion of physicians considered their explanation to be very clear compared with the proportion of patients and carers. Similarly, following the switch to PP3M, an improvement was reported in the frequency and the quality of communication between physicians/nurses and patients/carers; however, a larger proportion of physicians and nurses reported these improvements in communication compared with patients and carers.

A strong therapeutic alliance between patients and healthcare professionals is important in creating patient-centred, individualised treatment plans and optimising patient outcomes [23–25]. This is particularly applicable to the initiation of LAT, where patient preconceptions may be a barrier to medication acceptance, therefore, a clear explanation from the medical team regarding the clinical and personal benefits of a treatment is required [26]. In a recent Scandinavian study, patients treated with PP3M who were interviewed about several aspects of their treatment, highlighted the importance of their relationship with their healthcare teams in the success of their schizophrenia treatment [27]. In light of this, the findings of this study encouragingly demonstrate positive overall perceptions of communication between the patient and/or carer and healthcare team, which is an important aspect of shared decision-making and optimisation of personalised care. However, the differences in responses across stakeholders highlight opportunities for improved communication within treatment teams, and with patients and carers. The discrepancy among stakeholders regarding the clarity of explanation provided by the physician regarding the treatment switch indicates a gap in the clinical management of schizophrenia, whereby physicians and treatment teams as a whole should evaluate the clarity and the impact that the information they provide regarding treatment has on both patients and carers. Techniques such as psychoeducation may help to improve the transmission of medical information from healthcare professionals to patients and carers.

Theoretically, switching to less frequent antipsychotic administration should also allow more time to discuss other important topics with the patient, including functional goals that are important to a patient’s recovery, such as improving relationships with friends and family, gaining employment and increasing social activities [19, 28, 29]. In the current study, switching to PP3M resulted in increased time spent discussing non-medication topics for some; however, it was most frequently reported that there was ‘no change’.

A smaller proportion of patients were hospitalised for psychiatric reasons in the 1–1.5 years patients received PP3M compared with the 12 months prior to the treatment switch. It should be noted that the hospitalisation rate was already low in the 12 months prior to the switch, reinforcing the stability of the patient population included in this study.

Previous studies have reported a reduced risk of rehospitalisation and relapse in patients receiving LAT compared with oral antipsychotic treatment [30–32]. A decrease in hospitalisation rate has also been demonstrated specifically with the introduction of PP1M [33] and in those switching from PP1M to PP3M [14].

The observed decrease in hospitalisation rate in the study may, in part, be due to the longer half-life and therapeutic plasma level of PP3M compared with PP1M, thereby increasing the duration of effective treatment and protection from relapse [10, 11]. This theory is supported with findings from Weiden et al. who compared time to relapse when oral paliperidone, PP1M and PP3M were discontinued; time to relapse was longest for PP3M, followed by PP1M, then oral paliperidone [32]. These findings have also been corroborated by Mathews et al. [31].

It is also possible that with less frequent dosing than PP1M, PP3M may have improved compliance and retention rates. The compliance and retention rate of PP1M have been reported to be relatively high; furthermore, compliance has been shown to be directly associated with rehospitalisation rate [34]. Further research into the role of PP3M in reducing hospitalisation may be of value.

A recent prospective trial in a pragmatic clinical setting reported that, among other benefits, switching stable patients from PP1M to PP3M resulted in reduced carer burden following 12 months of treatment [14]. In the current study, most carers reported no change in time spent caring following switch to PP3M, but over one-quarter reported a decrease, further indicating that the switch has the potential to alleviate carer burden, even in patients who were stable at baseline. Data were not collected regarding the supportive activities that carers engaged in; however, PP3M has the potential to shift a carer’s focus from medication-related issues and non-adherence to other important supportive activities relating to the patient’s health and functioning [35, 36]. Such a change could impact the carer’s perception of their role, thereby reducing carer burden.

Notably, the current study reported a disconnect between the number of hours the carer reported to spend supporting the patient and the hours recognised by the patient, physician and nurse, suggesting that the role of carers is not fully understood and appreciated by other stakeholders. This underscores the issue of ensuring that carers have appropriate support (e.g. psychosocial interventions) to help alleviate carer burden [37].

### Strengths and limitations

Whereas previous studies/surveys on treatment experience with LAT have primarily been patient focussed, presenting experiences relating to a switch from PP1M to PP3M [14, 38], the current questionnaire is the first to present the perspectives of all major stakeholders (patients, physicians, nurses, and carers) on the switch from PP1M to PP3M. However, this was a retrospective, cross-sectional study providing qualitative data using a questionnaire; it is, therefore, subject to the limitations of a non-controlled, non-randomised study and is dependent on the stakeholder accurately recalling past events. The study design also does not allow for the long-term evaluation of the impact of PP3M.

Patients were required to have successfully switched to PP3M from PP1M and have received 4–6 administrations of PP3M. These inclusion criteria resulted in a biased selection of patients with positive experience of PP3M treatment and exclusion of those who discontinued PP3M after 1–3 doses, potentially due to compliance issues, worsening symptoms or adverse events.

In addition, although the questionnaire was developed in association with GAMIAN and EUFAMI, translated into local languages and adapted for the different stakeholders ensuring comparable answers, it is not validated and therefore, the possibility of unintended bias within the results should be considered.

Furthermore, the potential for social desirability bias to impact the outcomes of this study was acknowledged in the study design. Self-completion of the questionnaires and anonymity were ensured for all participants in order to minimise any social desirability bias.

## Conclusions

The results of the PINC-Q study add to the increasing body of evidence supporting the benefits of PP3M in a population representative of real-world clinical practice.

The most frequently reported reasons to switch from PP1M to PP3M were ‘to live life as normally as possible’ and ‘patient convenience’; the improvements reported following the switch, such as increased patient activity and social involvement, as well as a reduction in perceived stigma, appear to address these needs.

This study identified some differences in perception across the different stakeholders, highlighting the need for alignment within the treatment teams as well as improved communication between healthcare professionals and patients/carers, which could further optimise treatment of this patient population.

## Supplementary Information

**Additional file 1.** Details of Independent Ethics Committees involved in the PINC-Q study. Excel table containing details of Independent Ethics Committees involved in the PINC-Q study.

**Additional file 2.** Inter-rater alignment: pairwise and overall agreement across different stakeholders. Table containing all results of inter-rater alignment assessments made as part of the PINC-Q study.

**Additional file 3.** English physician one time questionnaire. English questionnaire on physician demographics.

**Additional file 4.** English physician questionnaire.

**Additional file 5.** English nurse one time questionnaire.

**Additional file 6.** English nurse questionnaire.

**Additional file 7.** English patient questionnaire.

**Additional file 8.** English carer questionnaire.

**Additional file 9.** English inclusion exclusion survey.

**Additional file 10.** English enrolment task.

## Data Availability

The datasets used and/or analysed during the current study are available from the corresponding author on reasonable request, subject to possible IP, privacy, regulatory and/or other constraints. An interim analysis of this study was presented at European College of Neuropsychopharmacology 2019 (Lahaye M et al. Presented at ECNP 2019, Copenhagen, 7–10 September 2019. Poster 391). A summary of the full analysis was presented as a poster at European Psychiatric Association (EPA) 2020.

## Abbreviations

CCC: Concordance correlation coefficient
CGI-S: Clinical Global Impression-Severity
EQ-5D-5L: European Quality of Life-5 dimension-5 level
EUFAMI: Europe and European Families Affected by Mental Health
GAMIAN: Global Alliance of Mental Illness Advocacy Networks
ICD-10: International Classification of Diseases
LAT: long-acting injectable antipsychotic treatment
PANSS: Positive and Negative Syndrome Scale
PINC-Q: Patient, Investigator, Nurse, Carer questionnaire
PP1M: paliperidone palmitate 1-monthly
PP3M: paliperidone palmitate 3-monthly
SD: standard deviation
