# Supplementary material for: The Patient, Investigator, Nurse, Carer Questionnaire (PINC-Q): a cross-sectional, retrospective, non-interventional study exploring the impact of less frequent medication administration with paliperidone palmitate 3-monthly as maintenance treatment for schizophrenia
Source: BMC Psychiatry. 2021 Jun 9;21:300. doi: 10.1186/s12888-021-03305-z (PMC8191017; doi:10.1186/s12888-021-03305-z)
Supplement: Supplementary file 2 — Additional file 2. Inter-rater alignment: pairwise and overall agreement across different stakeholders. Table containing all results of inter-rater alignment assessments made as part of the PINC-Q study. [file 12888_2021_3305_MOESM2_ESM.docx]

| **Result** | **n** | **Weighted kappa (95% CI)/Lin’s CCC (95% CI)^a^** | **SE** |
| --- | --- | --- | --- |
| **Involvement of patient in the decision to switch**  Physician–patient | 209 | 0.18 (0.06, 0.29) | 0.06 |
| **Involvement of carer in the decision to switch**  Physician–carer | 94 | 0.40 (0.26, 0.54) | 0.07 |
| **Involvement of nurse in the decision to switch**  Physician–nurse | 174 | 0.39 (0.28, 0.50) | 0.06 |
| **Who initiated the discussion to switch from PP1M to PP3M?**  Physician–nurse  Physician–patient  Physician–carer  Nurse–patient  Nurse–carer  Patient–carer  Overall | 185  221  96  185  86  97  86 | 0.53 (0.36, 0.71)  0.44 (0.26, 0.62)  0.24 (−0.03, 0.50)  0.48 (0.30, 0.65)  0.26 (−0.04, 0.57)  0.50 (0.23, 0.77)  0.38 (0.30, 0.46) | 0.09  0.09  0.14  0.09  0.15  0.14  0.04 |
| **Clarity of explanation from physician for switch from PP1M to PP3M**  Physician–patient  Physician–carer  Patient–carer  Overall | 220  94  95  94 | 0.14 (0.04, 0.25)  0.10 (−0.04, 0.24)  0.10 (−0.04, 0.23)  0.08 (−0.01, 0.18) | 0.05  0.07  0.07  0.05 |
| **Change in the frequency of physician–patient encounters**  Physician–patient  Physician–carer  Patient–carer  Overall | 217  96  97  96 | 0.47 (0.36, 0.58)  0.34 (0.17, 0.50)  0.49 (0.33, 0.64)  0.42 (0.32, 0.51) | 0.05  0.09  0.08  0.05 |
| **Change in the frequency of nurse–patient encounters**  Nurse–patient  Nurse–carer  Patient–carer  Overall | 181  87  98  87 | 0.49 (0.37, 0.60)  0.40 (0.23, 0.56)  0.50 (0.34, 0.66)  0.40 (0.30, 0.50) | 0.06  0.08  0.08  0.05 |
| **Change in the frequency of physician–carer encounters**  Physician–patient  Physician–carer  Patient–carer  Overall | 130  94  95  94 | 0.39 (0.24, 0.54)  0.31 (0.15, 0.48)  0.56 (0.40, 0.72)  0.38 (0.28, 0.48) | 0.08  0.08  0.08  0.05 |
| **Change in the frequency of nurse–carer encounters**  Nurse–patient  Nurse–carer  Patient–care  Overall | 113  82  95  82 | 0.48 (0.33, 0.64)  0.44 (0.26, 0.62)  0.51 (0.36, 0.66)  0.45 (0.35, 0.56) | 0.08  0.09  0.08  0.05 |
| **Change in the frequency of patient–carer encounters**  Patient–carer | 69 | 0.21 (−0.00, 0.41) | 0.11 |
| **Number of physician–patient encounters in previous 6 months**  Physician–patient  Physician–carer  Patient–carer  Overall | 205  84  91  84 | 0.81 (0.50, 0.95)  0.08 (−0.01, 0.87)  0.06 (0.00, 0.68)  0.13 (−0.00, 0.83) | 0.004  0.009  0.005  0.008 |
| **Satisfaction with the frequency of physician–patient encounters**  Physician–patient  Physician–carer  Patient–carer  Overall | 222  98  99  98 | 0.09 (−0.00, 0.19)  0.22 (0.07, 0.38)  0.17 (−0.00, 0.34)  0.10 (0.00, 0.20) | 0.05  0.08  0.09  0.05 |
| **Preferred frequency of physician–patient encounter**  Physician–nurse  Physician–patient  Physician–carer  Nurse–patient  Nurse–carer  Patient–carer  Overall | 181  216  95  184  85  96  85 | 0.45 (0.34, 0.56)  0.37 (0.26, 0.47)  0.38 (0.23, 0.52)  0.33 (0.22, 0.45)  0.31 (0.16, 0.46)  0.23 (0.09, 0.37)  0.24 (0.18, 0.29) | 0.06  0.05  0.08  0.06  0.08  0.07  0.03 |
| **Number of nurse–patient encounters in previous 6 months**  Nurse–patient  Nurse–carer  Patient–carer  Overall | 180  84  93  84 | 0.23 (0.05, 0.68)  0.67 (0.15, 0.98)  0.59 (0.05, 0.99)  0.57 (0.49, 0.82) | 0.005  0.008  0.008  0.003 |
| **Satisfaction with the frequency of nurse–patient encounters**  Nurse–patient  Nurse–carer  Patient–carer  Overall | 180  85  97  85 | 0.17 (0.04, 0.30)  0.22 (0.05, 0.39)  0.15 (−0.03, 0.33)  0.15 (0.03, 0.26) | 0.07  0.09  0.09  0.06 |
| **Preferred frequency of  nurse–patient encounter**  Physician–nurse  Physician–patient  Physician–carer  Nurse–patient  Nurse–carer  Patient–carer  Overall | 176  199  93  178  84  94  84 | 0.32 (0.20, 0.44)  0.24 (0.14, 0.35)  0.41 (0.25, 0.58)  0.37 (0.26, 0.48)  0.42 (0.27, 0.58)  0.48 (0.33, 0.64)  0.30 (0.24, 0.36) | 0.06  0.05  0.09  0.06  0.08  0.08  0.03 |
| **Number of physician–carer encounters in previous 6 months**  Physician–patient  Physician–carer  Patient–carer  Overall | 119  82  85  82 | 0.08 (0.00, 0.58)  0.02 (−0.05, 0.70)  0.15 (−0.04, 0.74)  0.47 (0.44, 0.67) | 0.005  0.009  0.009  0.002 |
| **Satisfaction with the frequency of physician–carer encounters**  Physician–patient  Physician–carer  Patient–carer  Overall | 123  91  92  91 | 0.12 (−0.02, 0.27)  0.18 (0.02, 0.34)  0.15 (−0.04, 0.34)  0.11 (0.01, 0.21) | 0.07  0.08  0.10  0.05 |
| **Number of nurse–carer encounters in previous 6 months**  Nurse–patient  Nurse–carer  Patient–carer  Overall | 102  74  88  74 | 0.04 (−0.01, 0.69)  0.04 (0.01, 0.51)  0.63 (−0.03, 0.94)  0.32 (−0.04, 0.51) | 0.004  0.004  0.011  0.006 |
| **Satisfaction with the frequency of nurse–carer encounters**  Nurse–patient  Nurse–carer  Patient–carer  Overall | 106  77  87  77 | 0.15 (0.00, 0.29)  0.14 (−0.02, 0.31)  0.12 (−0.06, 0.30)  0.07 (−0.04, 0.18) | 0.07  0.08  0.09  0.06 |
| **Number of patient–carer encounters in previous 2 months**  Patient–carer | 27 | 0.69 (0.38, 0.95) | 0.005 |
| **Satisfaction with the frequency of patient–carer encounters**  Patient–carer | 91 | 0.13 (−0.04, 0.30) | 0.09 |
| **Total hours per week the carer supports the patient**  Physician–nurse  Physician–patient  Physician–carer  Nurse–patient  Nurse–carer  Patient–carer  Overall | 103  125  91  108  78  92  78 | 0.54 (0.43, 0.65)  0.57 (0.46, 0.68)  0.39 (0.27, 0.52)  0.59 (0.48, 0.70)  0.49 (0.34, 0.63)  0.64 (0.52, 0.75)  0.34 (0.30, 0.39) | 0.06  0.06  0.06  0.06  0.07  0.06  0.02 |
| **Change in communication quality between physician–patient**  Physician–patient | 219 | 0.17 (0.07, 0.28) | 0.05 |
| **Change in communication quality between nurse–patient**  Nurse–patient | 181 | 0.16 (0.05, 0.28) | 0.06 |
| **Change in communication quality between physician–carer**  Physician–carer | 96 | 0.31 (0.11, 0.50) | 0.10 |
| **Change in communication quality between nurse–carer**  Nurse–carer | 83 | 0.43 (0.23, 0.63) | 0.10 |
| **Discussion of non-medication topics by physician–nurse–patient**  Physician–nurse  Physician–patient  Nurse–patient  Overall | 185  220  187  185 | 0.27 (0.15, 0.39)  0.14 (0.04, 0.25)  0.28 (0.16, 0.40)  0.23 (0.16, 0.31) | 0.06  0.05  0.06  0.04 |
| **Discussion of non-medication topics by physician–nurse–carer**  Physician–nurse  Physician–carer  Nurse–carer  Overall | 106  94  83  81 | 0.29 (0.14, 0.44)  0.27 (0.10, 0.44)  0.40 (0.23, 0.57)  0.32 (0.22, 0.43) | 0.08  0.09  0.09  0.05 |
| **Discussion of non-medication topics by carer–patient**  Patient–carer | 96 | 0.41 (0.24, 0.58) | 0.09 |
| **PP3M helps the patient**  Physician–nurse  Physician–patient  Physician–carer  Nurse–patient  Nurse–carer  Patient–carer  Overall | 183  218  98  185  89  99  89 | 0.09 (−0.15, 0.34)  0.09 (-0.01, 0.19)  −0.03 (−0.07, 0.00)  0.28 (0.13, 0.43)  0.11 (−0.14, 0.36)  0.05 (−0.12, 0.22)  0.12 (0.04, 0.20) | 0.13  0.05  0.02  0.08  0.13  0.09  0.04 |
| **Change in activity levels following switch to PP3M**  Physician–nurse  Physician–patient  Physician–carer  Nurse–patient  Nurse–carer  Patient–carer  Overall | 180  219  96  182  85  97  85 | 0.45 (0.32, 0.57)  0.33 (0.22, 0.45)  0.30 (0.14, 0.46)  0.42 (0.30, 0.54)  0.45 (0.28, 0.63)  0.28 (0.12, 0.44)  0.38 (0.31, 0.46) | 0.06  0.06  0.08  0.06  0.09  0.08  0.04 |
| **Change in patient communication with family, friends and other people following switch to PP3M**  Physician–nurse  Physician–patient  Physician–carer  Nurse–patient  Nurse–carer  Patient–carer  Overall | 167  211  96  169  82  97  82 | 0.36 (0.23, 0.50)  0.24 (0.13, 0.36)  0.21 (0.04, 0.37)  0.36 (0.22, 0.50)  0.37 (0.19, 0.56)  0.33 (0.17, 0.49)  0.35 (0.27, 0.43) | 0.07  0.06  0.09  0.07  0.09  0.08  0.04 |
| **Change in carer time required for patient support following switch to PP3M**  Physician–nurse  Physician–patient  Physician–carer  Nurse–patient  Nurse–carer  Patient–carer  Overall | 106  127  94  110  82  95  82 | 0.49 (0.32, 0.65)  0.24 (0.11, 0.37)  0.40 (0.24, 0.57)  0.28 (0.12, 0.43)  0.47 (0.27, 0.66)  0.40 (0.23, 0.58)  0.39 (0.32, 0.47) | 0.09  0.07  0.08  0.08  0.10  0.09  0.04 |
| **Change in amount of carer–patient support following switch to PP3M**  Physician–nurse  Physician–patient  Physician–carer  Nurse–patient  Nurse–carer  Patient–carer  Overall | 108  126  91  112  81  92  81 | 0.31 (0.14, 0.47)  0.30 (0.16, 0.45)  0.26 (0.08, 0.45)  0.20 (0.03, 0.37)  0.36 (0.16, 0.57)  0.35 (0.18, 0.53)  0.34 (0.25, 0.42) | 0.08  0.07  0.09  0.09  0.10  0.09  0.04 |
| **Stigmatisation**  Physician–nurse  Physician–patient  Physician–carer  Nurse–patient  Nurse–carer  Patient–carer  overall | 182  219  97  183  86  98  86 | 0.28 (0.17, 0.38)  0.20 (0.11, 0.28)  0.22 (0.08, 0.36)  0.27 (0.17, 0.37)  0.16 (0.01, 0.31)  0.25 (0.10, 0.41)  0.18 (0.12, 0.23) | 0.05  0.04  0.07  0.05  0.08  0.08  0.03 |
| **Impact on stigma of switch to PP3M**  Physician–nurse  Physician–patient  Physician–carer  Nurse–patient  Nurse–carer  Patient–carer  Overall | 181  219  95  182  84  96  84 | 0.30 (0.17, 0.43)  0.18 (0.09, 0.27)  0.28 (0.12, 0.43)  0.20 (0.08, 0.31)  0.23 (0.06, 0.40)  0.25 (0.08, 0.41)  0.25 (0.17, 0.32) | 0.07  0.05  0.08  0.06  0.09  0.08  0.04 |

^a^All results are reported as a weighted kappa, except for the number of physician–patient encounters in the previous 6 months, number of nurse–patient encounters in the previous 6 months, number of physician–carer encounters in the previous 6 months, number of nurse–carer encounters in the previous 6 months, number of patient–carer encounters in the previous 2 months, which are Lin’s CCC.

Both the (weighted) kappa statistic and Lin’s CCC value vary from 0 to 1, where: 0 = no agreement/agreement equivalent to chance; 0.1–0.20 = non-slight agreement; 0.21–0.40 = fair agreement; 0.41–0.60 = moderate agreement; 0.61–0.80 = substantial agreement; 0.81–1.00 = almost perfect agreement.
CCC, concordance correlation coefficient; CI, confidence interval; PP1M, paliperidone palmitate 1-monthly; PP3M, paliperidone palmitate 3-monthly; SE, standard error.
