# Supplementary material for: The Patient, Investigator, Nurse, Carer Questionnaire (PINC-Q): a cross-sectional, retrospective, non-interventional study exploring the impact of less frequent medication administration with paliperidone palmitate 3-monthly as maintenance treatment for schizophrenia
Source: BMC Psychiatry. 2021 Jun 9;21:300. doi: 10.1186/s12888-021-03305-z (PMC8191017; doi:10.1186/s12888-021-03305-z)
Supplement: Supplementary file 3 — Additional file 3. English physician one time questionnaire. English questionnaire on physician demographics. [file 12888_2021_3305_MOESM3_ESM.pdf]

Survey 360
PHYSICIAN QUESTIONNAIRE
(should be completed only by physician)

Questions on slide 3-4 are asked only once

PHYSICIAN GENERAL CHARACTERISTICS

Age: |__|__| years

Gender:
❑ Male
❑ Female

Practice Setting (please select the facility you are working in):
❑ Inpatient facility (i.e. ward for >24- hours hospitalizations)
❑ Outpatient facility
❑ Day hospital
❑ Other

Type of Facility (please select the option that applies):
❑ Private Facility
❑ Public Facility

PHYSICIAN GENERAL CHARACTERISTICS

Experience as a certified psychiatrists (in years)

- ❑ Less than 5
- ❑ 5-10
- ❑ 11-15
- ❑ >15

How many patients living with schizophrenia do you see in a month?

|__|__|__| number

How many of those are on second generation long acting treatments

|__|__|__| number

How many of those are on PP3M?

|__|__|__| number

Whom do you find important to involve in the treatment decision? (multiple answers)

- ❑ Patient
- ❑ Caregiver
- ❑ Doctor
- ❑ Nurse
- ❑ Other
