# Supplementary material for: The Patient, Investigator, Nurse, Carer Questionnaire (PINC-Q): a cross-sectional, retrospective, non-interventional study exploring the impact of less frequent medication administration with paliperidone palmitate 3-monthly as maintenance treatment for schizophrenia
Source: BMC Psychiatry. 2021 Jun 9;21:300. doi: 10.1186/s12888-021-03305-z (PMC8191017; doi:10.1186/s12888-021-03305-z)
Supplement: Supplementary file 4 — Additional file 4. English physician questionnaire. [file 12888_2021_3305_MOESM4_ESM.pdf]

English Psychiatrist Survey

Welcome to the English Psychiatrist Survey. This survey has approximately 50 questions, we anticipate it will take 15-20 minutes to complete.

Get Started

Cancel

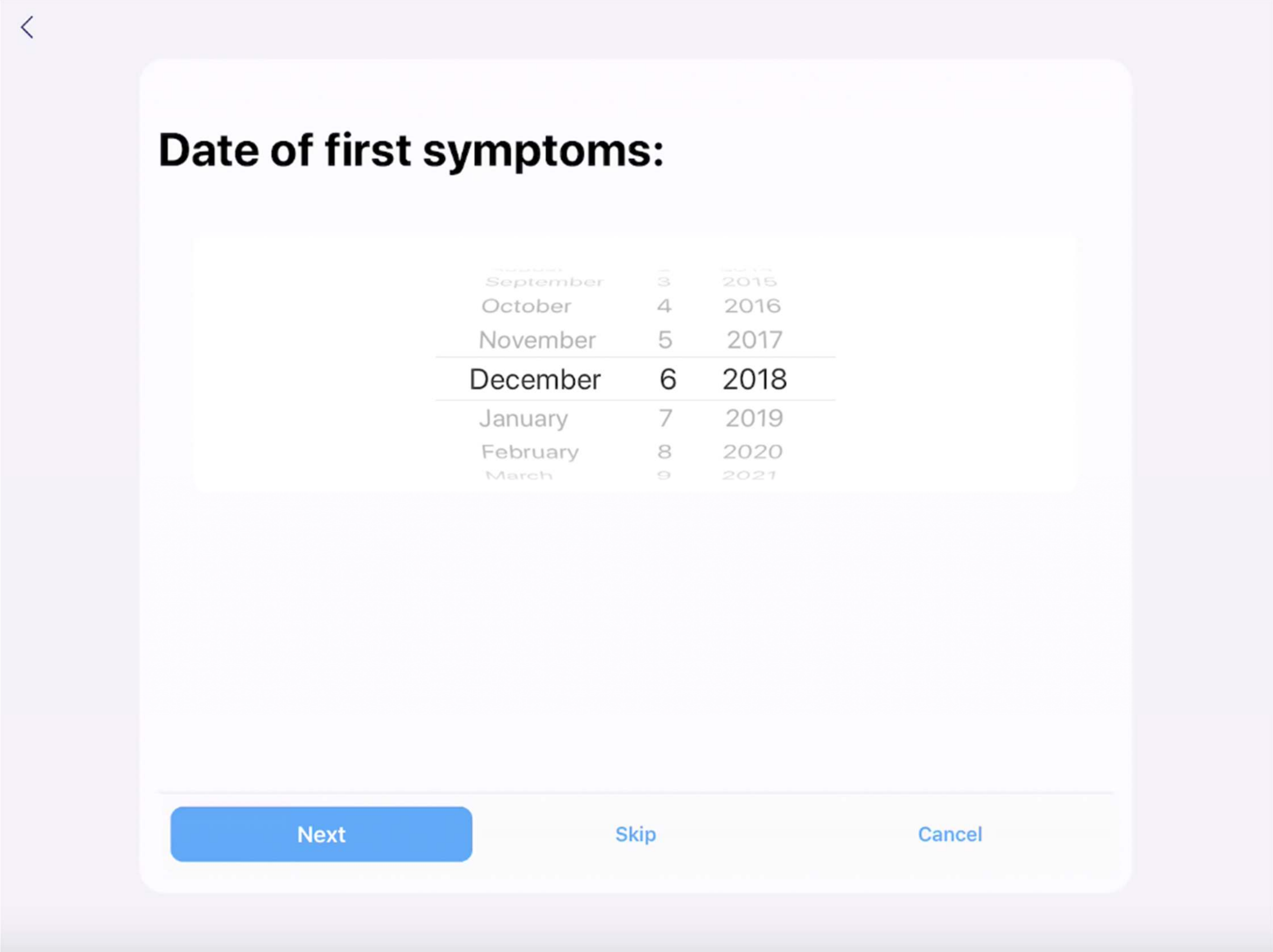
Date of first symptoms:
September 3 2015
October 4 2016
November 5 2017
December 6 2018
January 7 2019
February 8 2020
March 9 2021
Next
Skip
Cancel

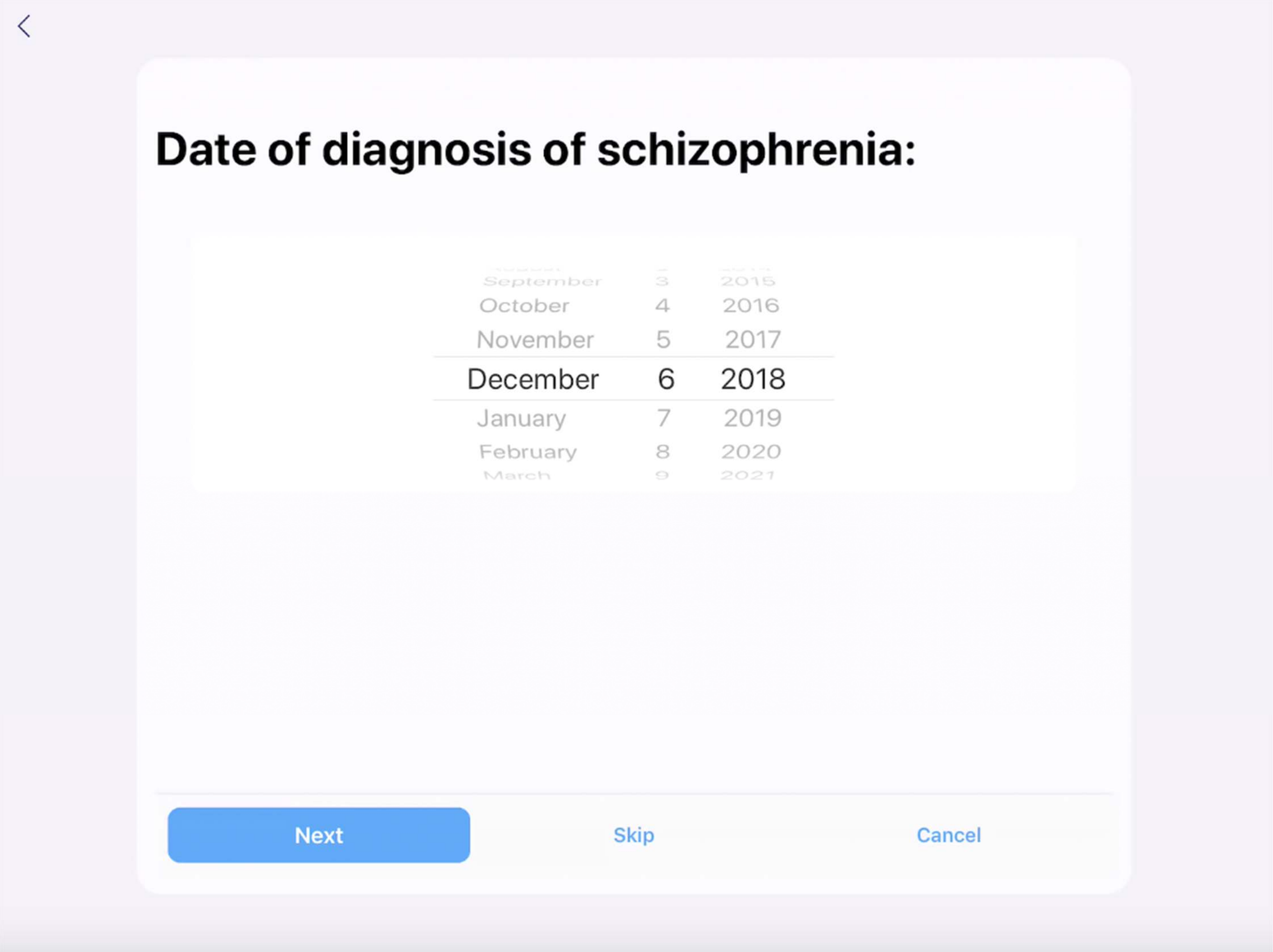
Date of diagnosis of schizophrenia:
September 3 2015
October 4 2016
November 5 2017
December 6 2018
January 7 2019
February 8 2020
March 9 2021
Next
Skip
Cancel

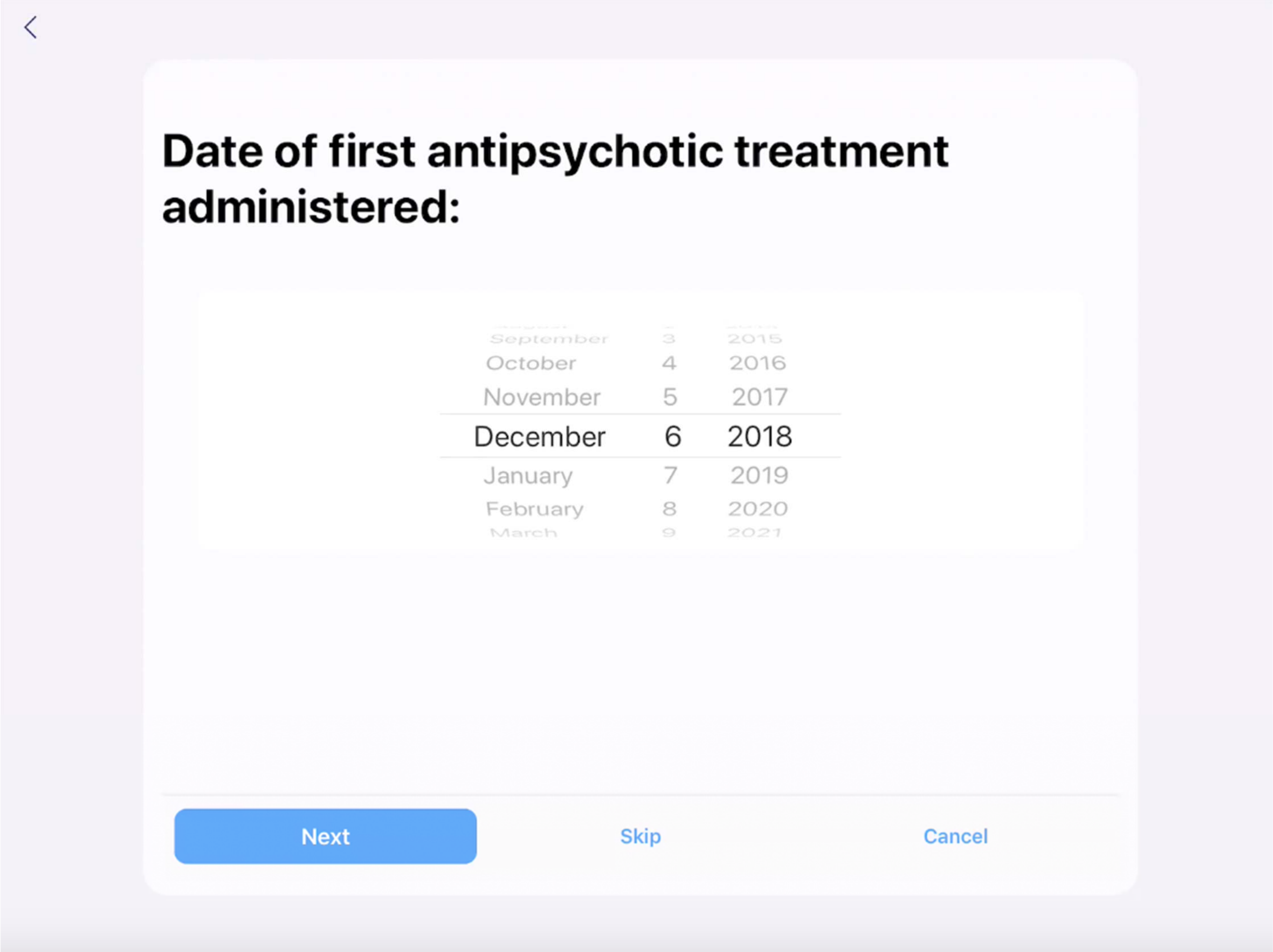
Date of first antipsychotic treatment administered:
September 3 2015
October 4 2016
November 5 2017
December 6 2018
January 7 2019
February 8 2020
March 9 2021
Next
Skip
Cancel

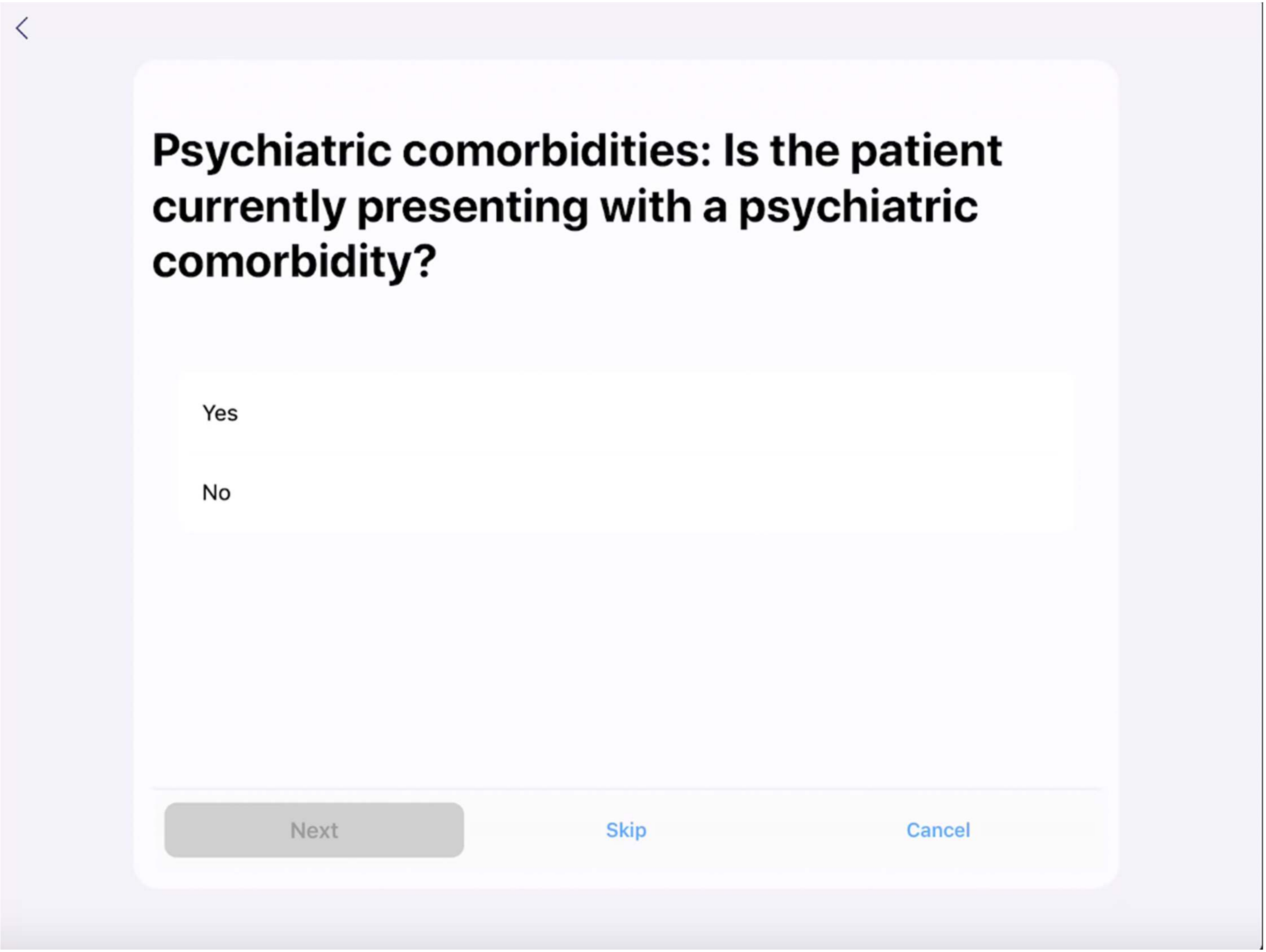
Psychiatric comorbidities: Is the patient currently presenting with a psychiatric comorbidity?
Yes
No
Next
Skip
Cancel

If YES, please precise psychiatric comorbidities (multiple answers). Scroll to view all

Major Depressive Disorder

Generalized Anxiety Disorder

Agoraphobia

Social phobia

Obsessive Compulsive Disorder

Generalized Anxiety Disorder

Panic Disorder

Post-Traumatic Stress

Other

Next Skip Cancel

How many cigarettes does patient smoke per day?

Doesn't smoke

1-5

5-10

10-20

More than 20

Next

Skip

Cancel

Is there any notion of alcohol abuse or dependence (according to ICD-10)?

Yes

No

Next | Skip | Cancel

Is there any notion of psychotropic substance abuse or dependence (according to ICD-10)?

Yes

No

Next Skip Cancel

Non-psychiatric comorbidities: Is the patient currently presenting a non-psychiatric comorbidity?

Yes

No

Next Skip Cancel

If YES, please precise non-psychiatric comorbidity (multiple answers). Scroll to view all

- Endocrine disorders
- Metabolic disorders
- Nervous system disorders
- Cardio-Vascular disorders
- Gastrointestinal and hepatobiliary disorders
- Reproductive system and breast disorders
- Other

Next | Skip | Cancel

Clinical Global Impression – Severity scale (CGI-S) At the inclusion, how do you assess the current severity of the patient's illness? Scroll to view all

Normal, not at all ill

Borderline mentally ill

Mildly ill

Moderately ill

Markedly ill

Severely ill

Among the most extremely ill patients

Next

Skip

Cancel

This question is two pages:

G12. LACK OF JUDGEMENT AND INSIGHT

Impaired awareness or understanding of one's own psychiatric condition and life situation. This is evidenced by failure to recognise past or present psychiatric illness or symptoms, denial of need for psychiatric hospitalisation or treatment, decisions characterised by poor anticipation or consequences, and unrealistic short-term and long-range planning. Scroll to view all.

Basis for rating – Thought content expressed during the interview.

1. Absent – Definition does not apply

2. Minimal – Questionable pathology; may be at the upper extreme of normal limits

3. Mild – Recognises having a psychiatric disorder but clearly underestimates its seriousness, the implications for treatment, or the importance of taking measures to avoid relapse. Future planning may be poorly conceived

4. Moderate – Patient shows only a vague or shallow recognition of illness. There may be fluctuations in acknowledgment of being ill or little awareness of major symptoms which are present, such as delusions, disorganised thinking, suspiciousness and social withdrawal. The patient may rationalise the need for treatment in terms of its relieving lesser symptoms, such as anxiety, tension and sleep difficulty.

Next | Skip | Cancel

‹

G12. LACK OF JUDGEMENT AND INSIGHT

4. Moderate – Patient shows only a vague or shallow recognition of illness. There may be fluctuations in acknowledgment of being ill or little awareness of major symptoms which are present, such as delusions, disorganised thinking, suspiciousness and social withdrawal. The patient may rationalise the need for treatment in terms of its relieving lesser symptoms, such as anxiety, tension and sleep difficulty.

5. Moderate Severe – Acknowledges past but not present psychiatric disorder. If challenged, the patient may concede the presence of some unrelated or insignificant symptoms, which tend to be explained away by gross misinterpretation or delusional thinking. The need for psychiatric treatment similarly goes unrecognised.

6. Severe – Patient denies ever having had a psychiatric disorder. He disavows the presence of any psychiatric symptoms in the past or present and, though compliant, denies the need for treatment and hospitalisation.

7. Extreme – Emphatic denial of past and present psychiatric illness. Current hospitalisation and treatment are given a delusional interpretation (e.g. as punishment for misdeeds, as persecution by tormentors, etc), and the patient thus refuse to cooperate with therapists, medicine or other aspects of treatment

Select one:

Next Skip Cancel

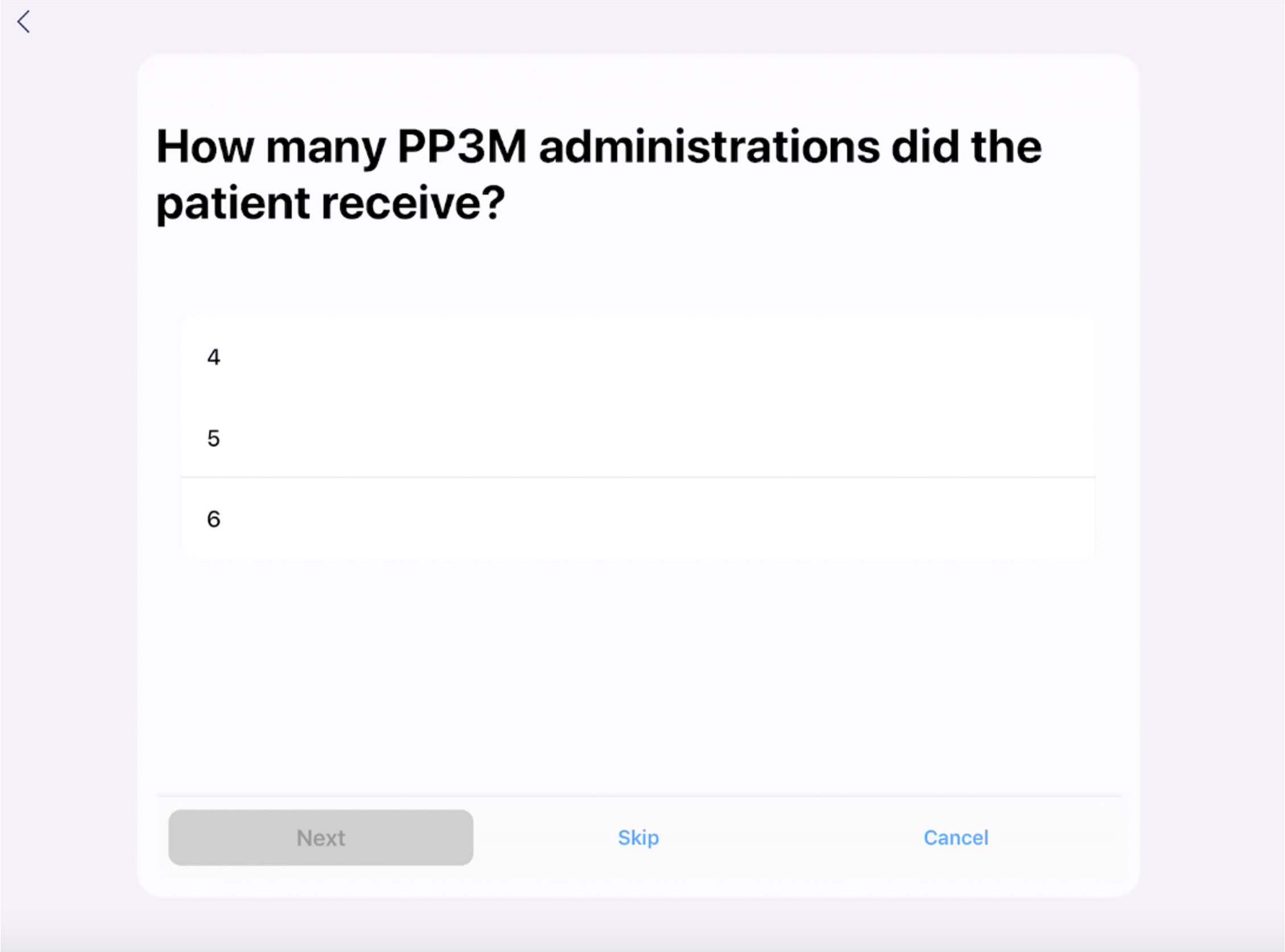
How many PP3M administrations did the patient receive?
4
5
6
Next
Skip
Cancel

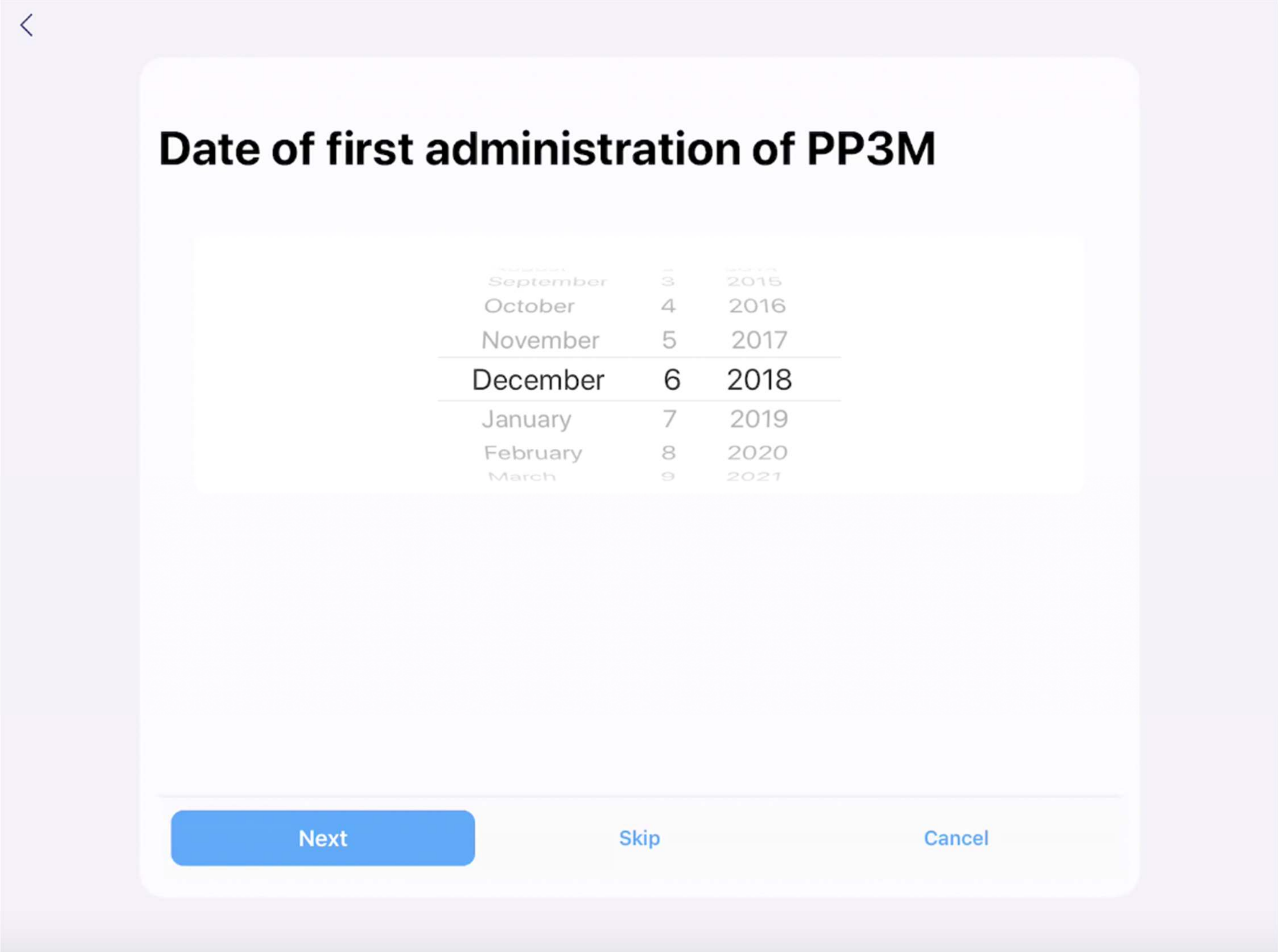
Date of first administration of PP3M
September 3 2015
October 4 2016
November 5 2017
December 6 2018
January 7 2019
February 8 2020
March 9 2021
Next
Skip
Cancel

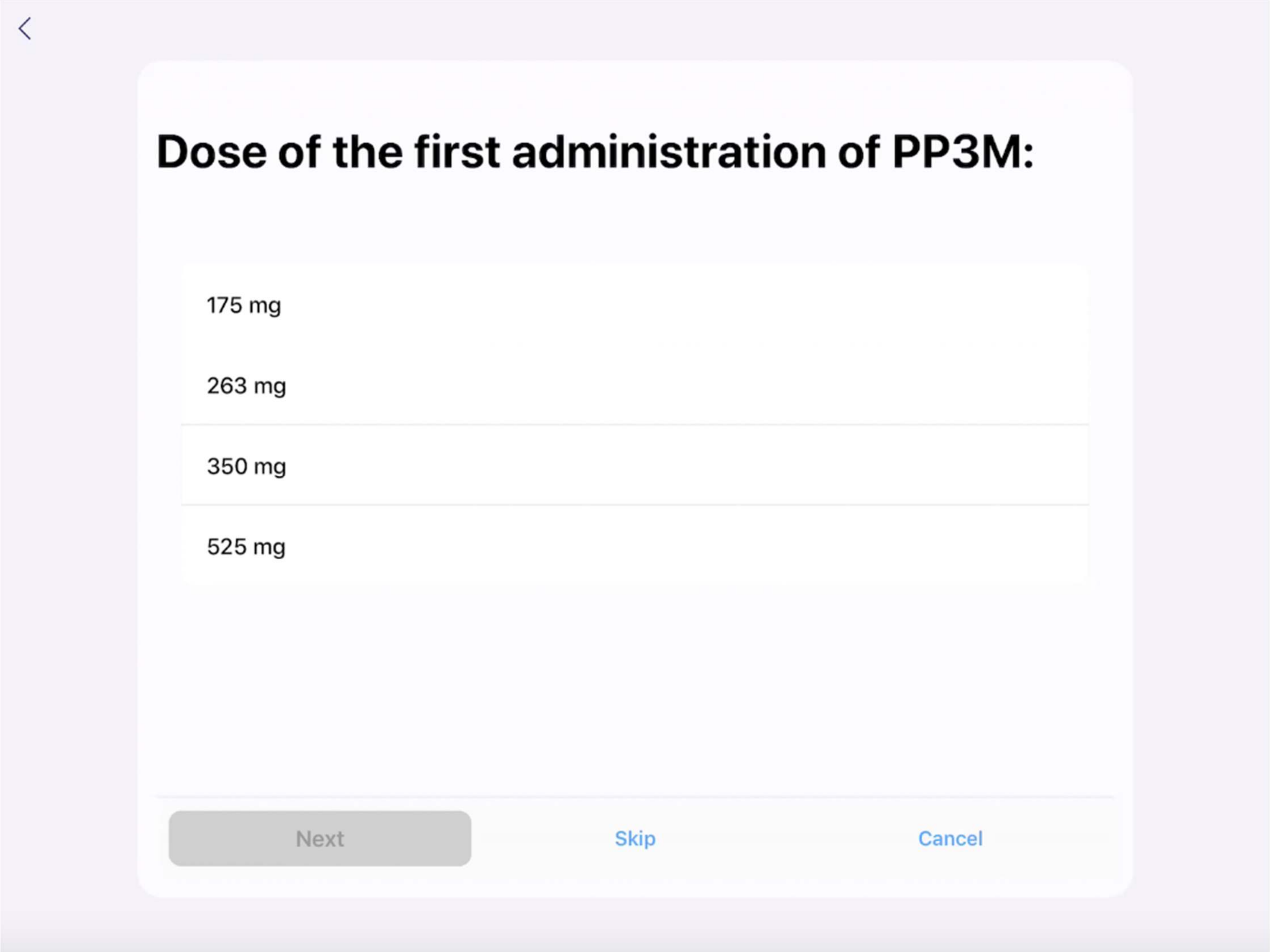
Dose of the first administration of PP3M:
175 mg
263 mg
350 mg
525 mg
Next
Skip
Cancel

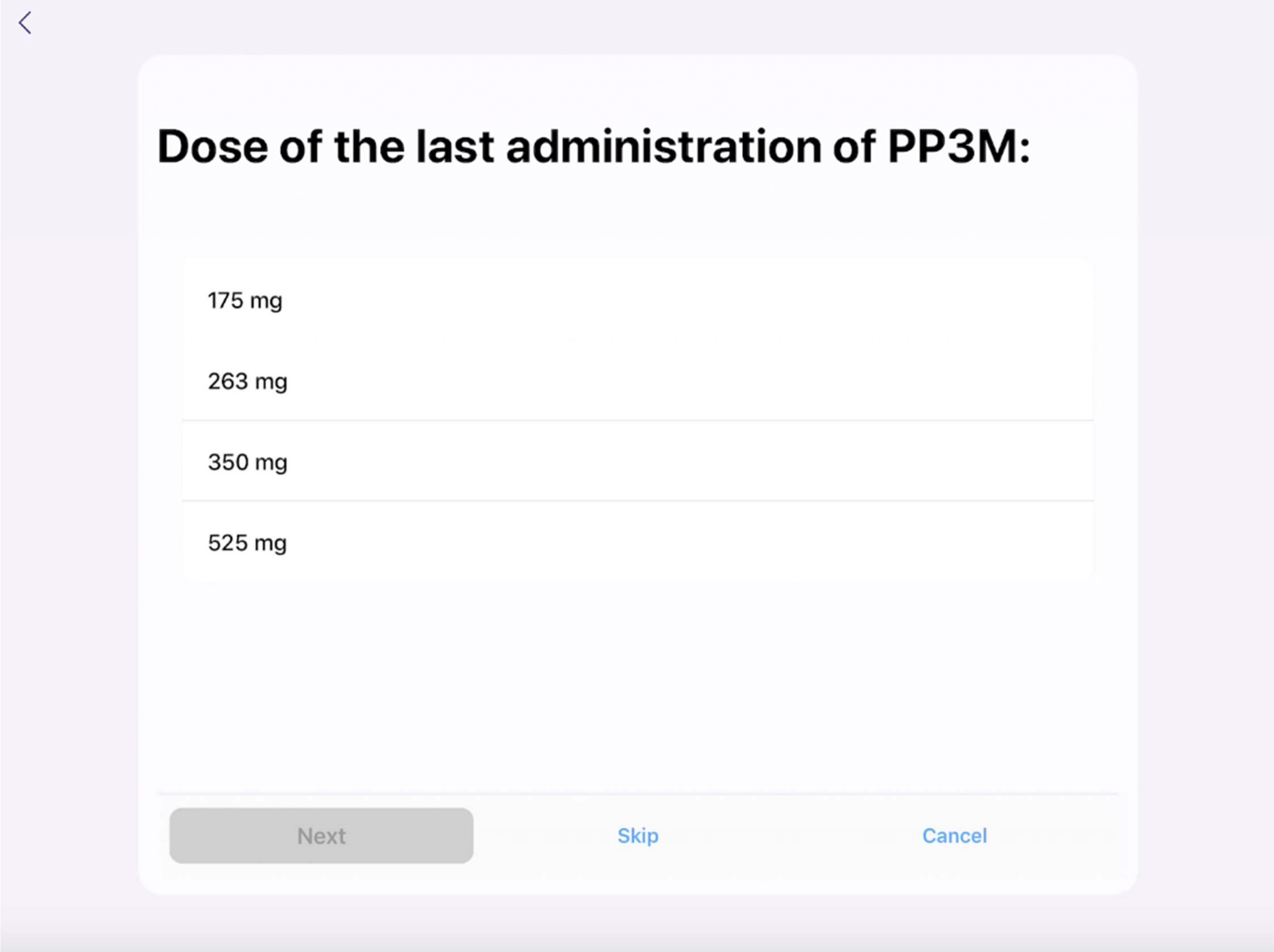
Dose of the last administration of PP3M:
175 mg
263 mg
350 mg
525 mg
Next
Skip
Cancel

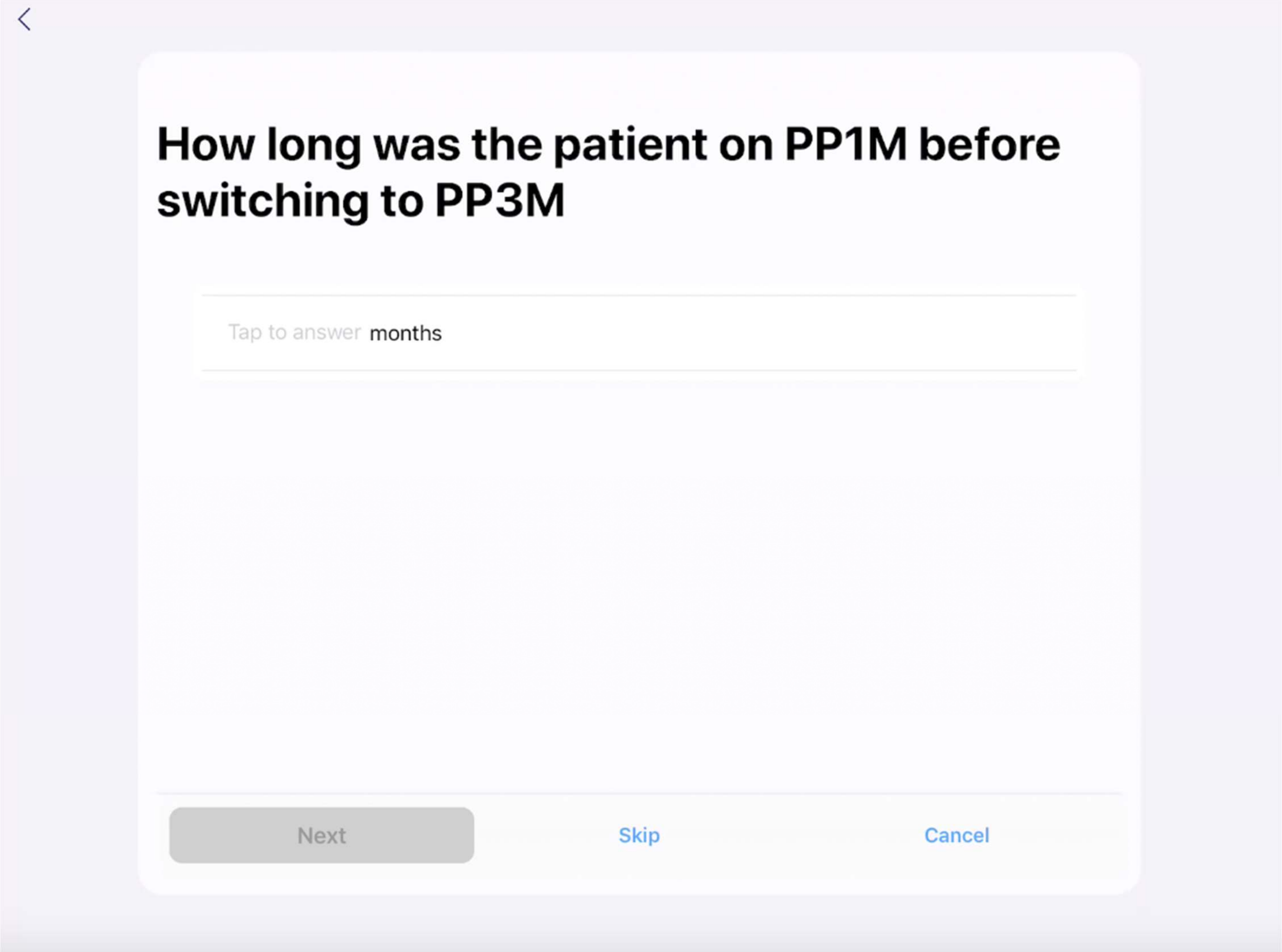
How long was the patient on PP1M before switching to PP3M
Tap to answer months
Next
Skip
Cancel

What was the last dose of PP1M before switching to PP3M:

25 mg

50 mg

75 mg

100 mg

150 mg

Next Skip Cancel

During the past 12 months, in which care facilities have the PP3M injections been administered the most of time to the patient? (multiple answers)

Hospital nurse office

Private nurse office

Patient housing

Day hospital

Private psychiatrist office

Other

Next

Skip

Cancel

<

What other concomitant psychotropic treatments are currently prescribed in addition to PP3M ? (multiple answers) Scroll to view all

None

Oral antipsychotic

Anxiolytic / hypnotic

Anticholinergic

Antidepressant

Mood stabilizer

Other

Next

Skip

Cancel

<

What type of non-pharmacological treatment does the patient receive? Cares does the patient currently follow? Scroll to view all

- None
- Cognitive-behavioural treatment
- Analytically oriented psychotherapy
- Mindfulness
- Systemic therapy
- Psychoeducation therapy
- Cognitive remediation
- Social abilities training
- Mediation (painting, sport, ...)
- Relaxation
- Electroconvulsive therapy
- Neuromodulation (tDCS, rTMS)
- Other

Next Skip Cancel

Since PP3M initiation, has the patient been hospitalized (full hospitalization) for psychiatric reason?

Yes

No

Next Skip Cancel

If yes: number of hospitalizations

Tap to answer

Next   Skip   Cancel

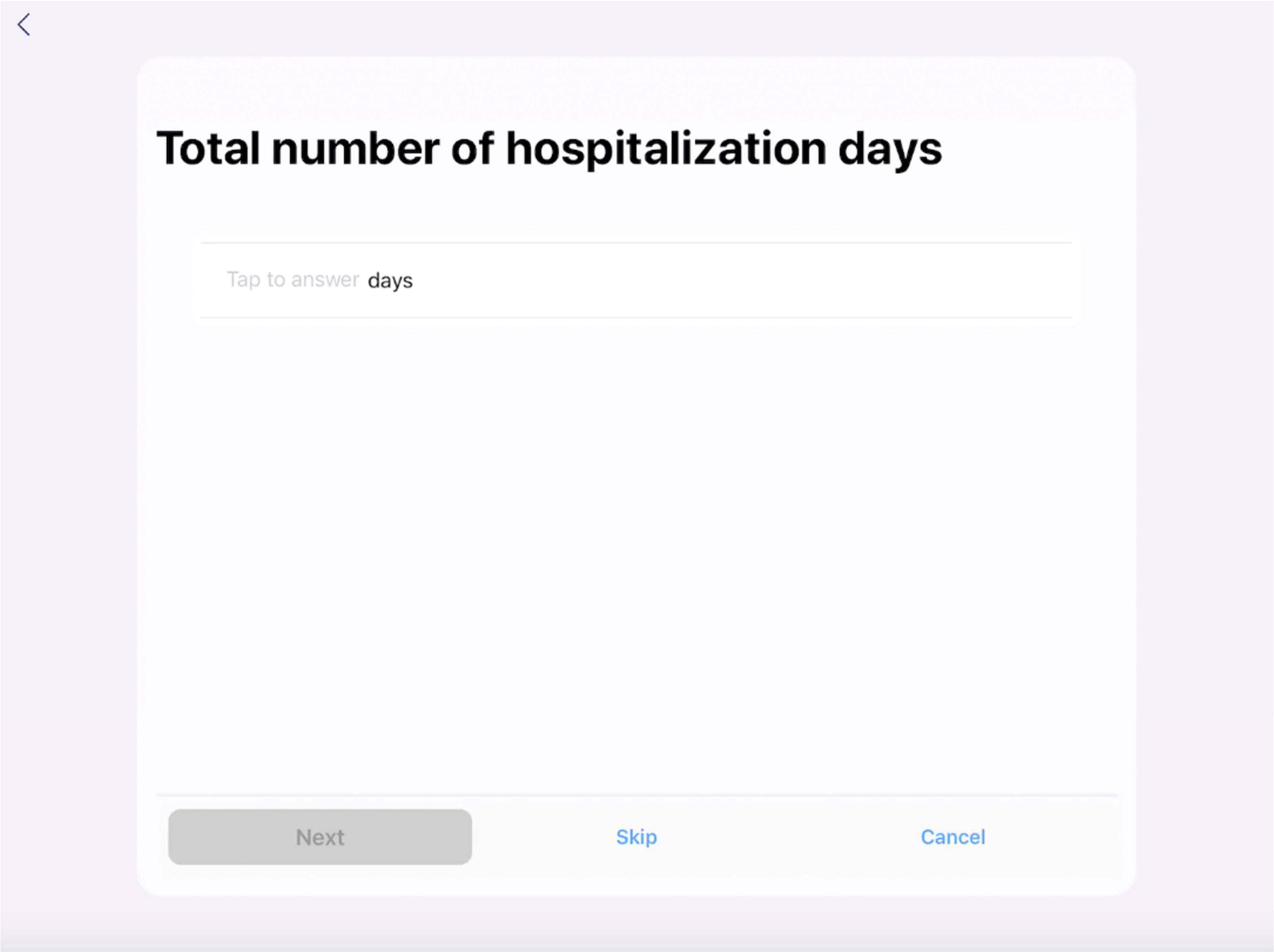
Total number of hospitalization days
Tap to answer days
Next
Skip
Cancel

<
12 months before PP3M initiation, has the patient been hospitalized (full hospitalization) for psychiatric reason?

Yes

No

Next Skip Cancel

If yes: number of hospitalizations

Tap to answer

Next Skip Cancel

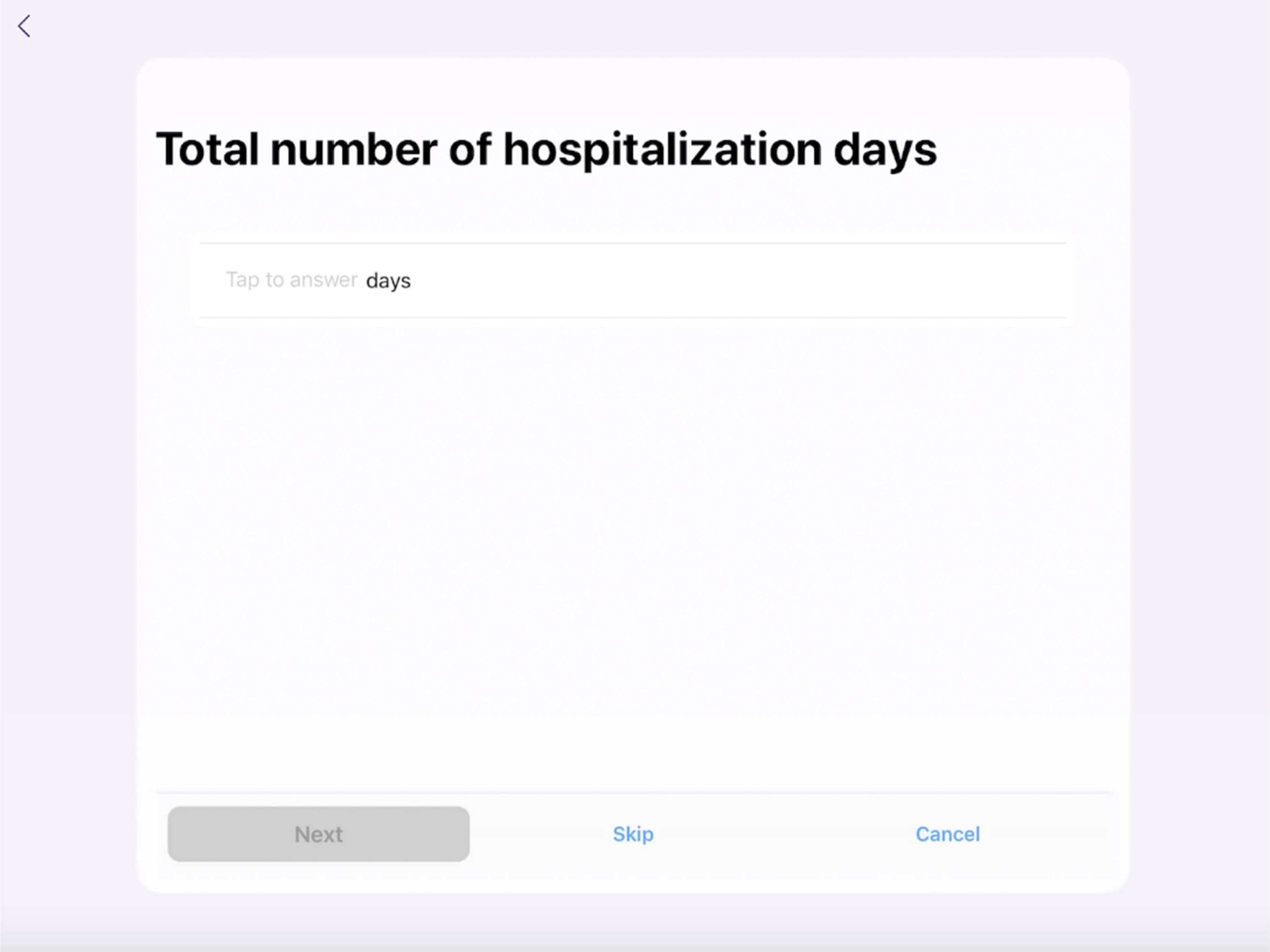
Total number of hospitalization days
Tap to answer days
Next
Skip
Cancel

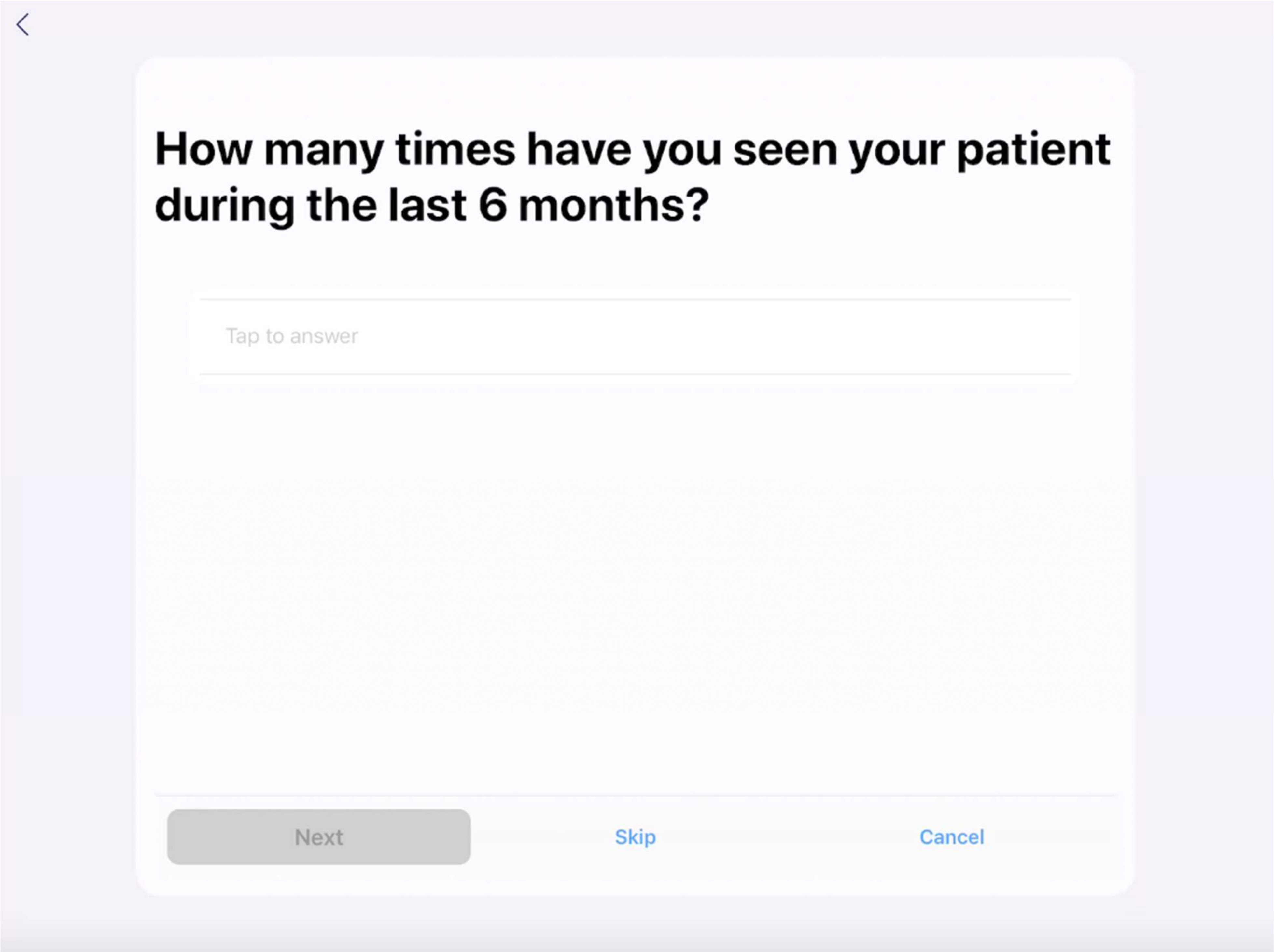
How many times have you seen your patient during the last 6 months?
Tap to answer
Next
Skip
Cancel

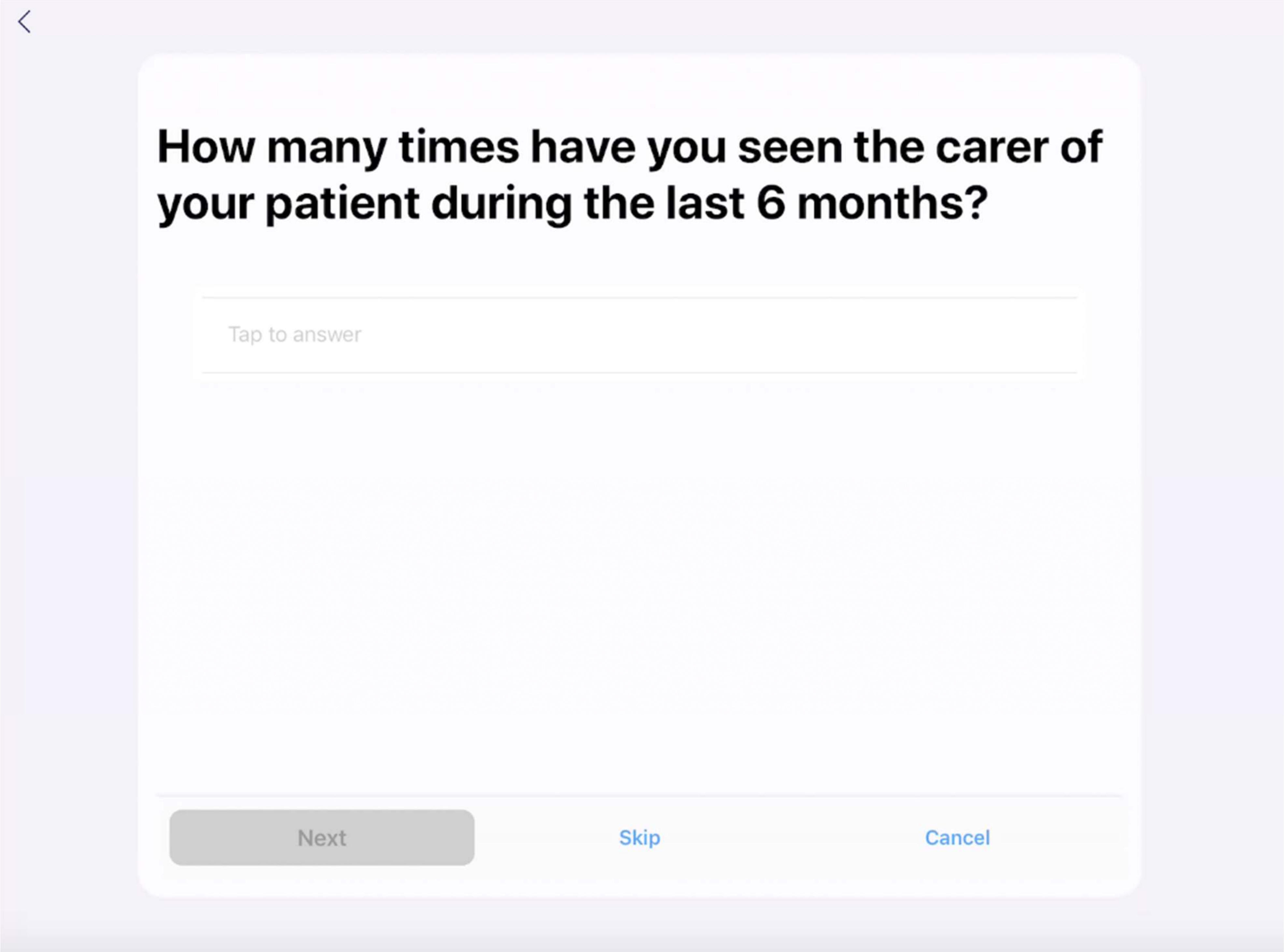
How many times have you seen the carer of your patient during the last 6 months?
Tap to answer
Next
Skip
Cancel

Has the frequency in which you see your patient changed as a result of switching to PP3M?

We meet more frequently

We meet less frequently

There is no change

Next | Skip | Cancel

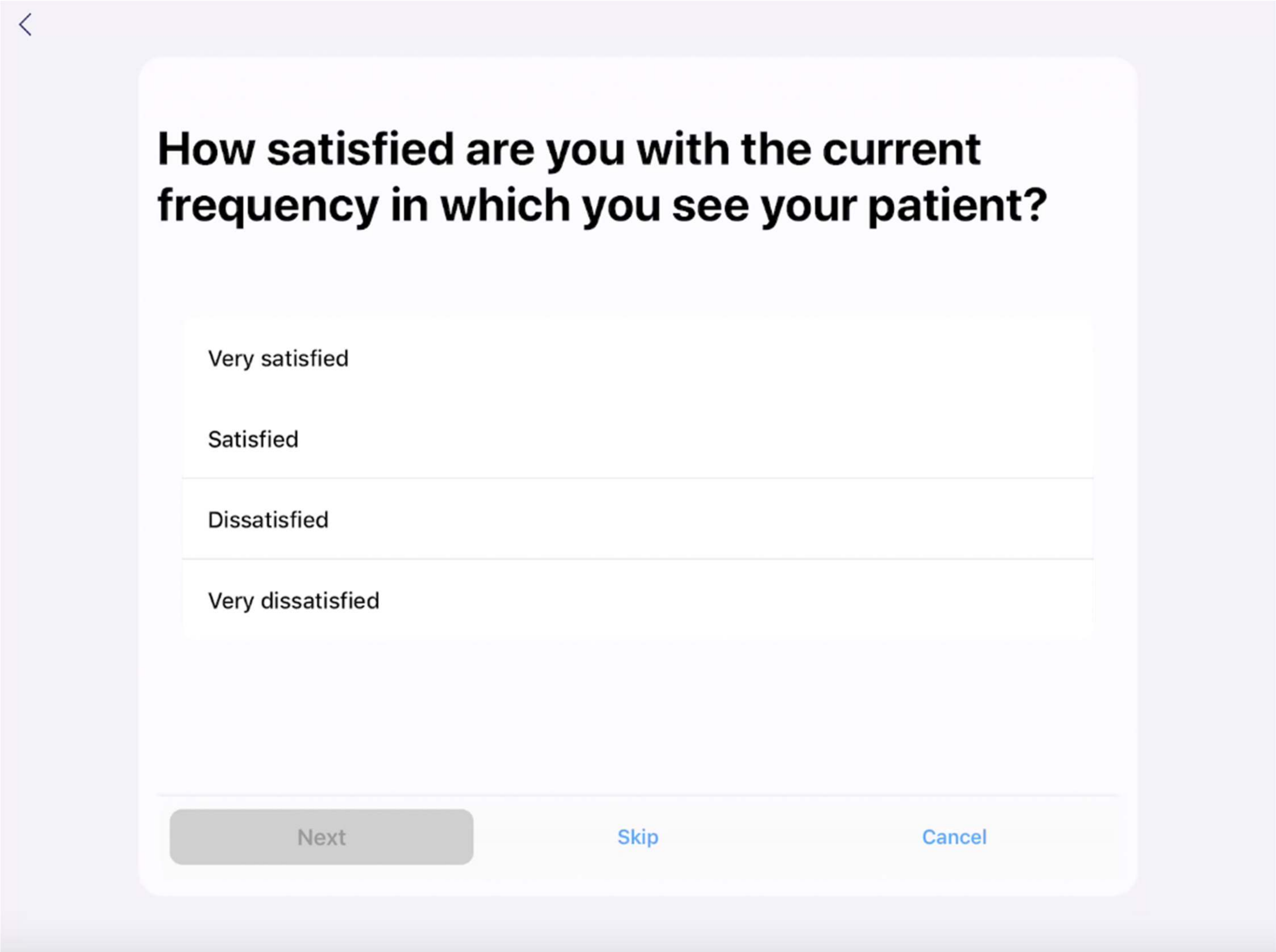
How satisfied are you with the current frequency in which you see your patient?
Very satisfied
Satisfied
Dissatisfied
Very dissatisfied
Next
Skip
Cancel

‹

Has the frequency in which you see the carer of your patient changed as a result of switching to PP3M?

- We meet more frequently
- We meet less frequently
- There is no change

Next | Skip | Cancel

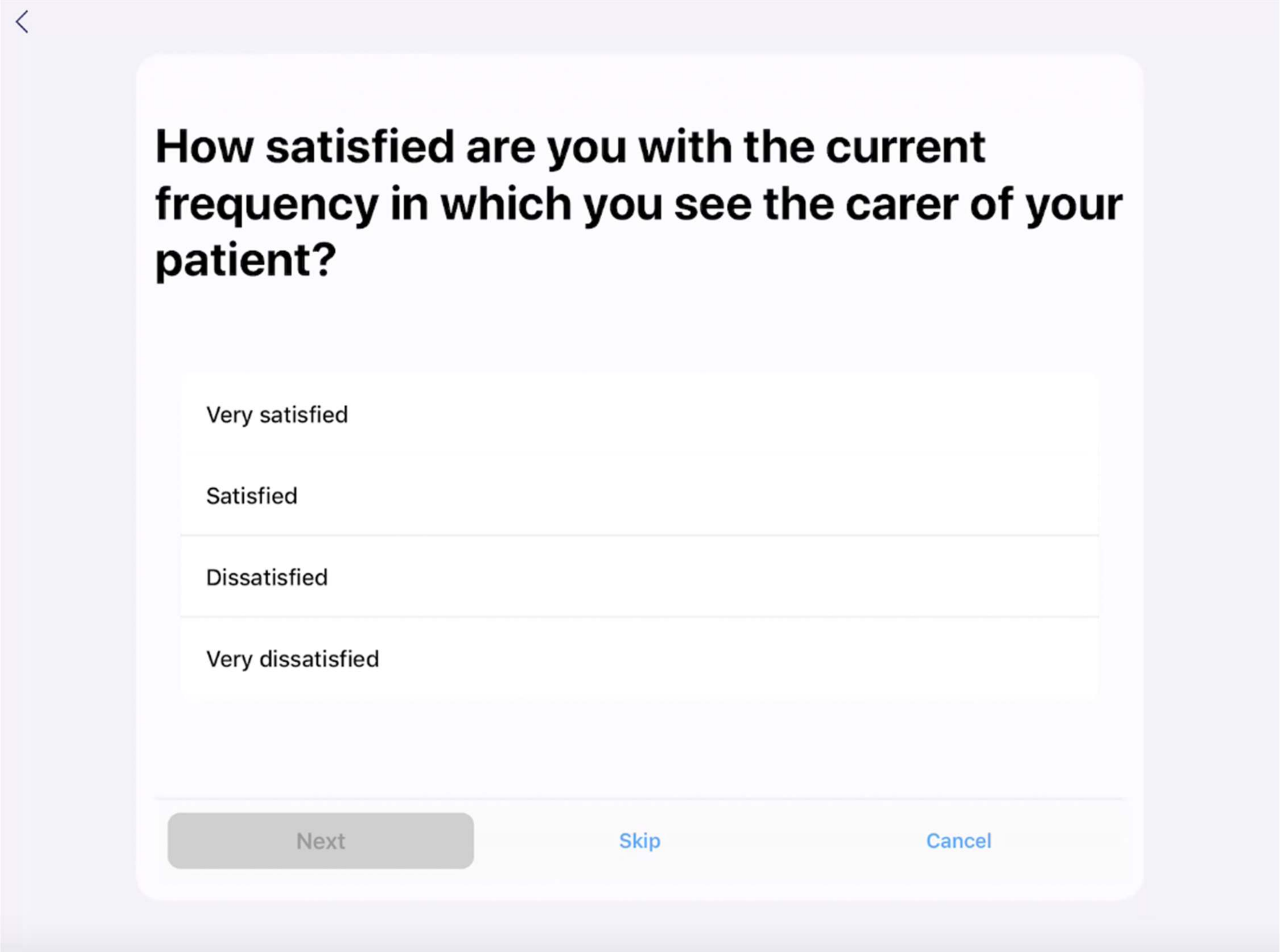
How satisfied are you with the current frequency in which you see the carer of your patient?
Very satisfied
Satisfied
Dissatisfied
Very dissatisfied
Next
Skip
Cancel

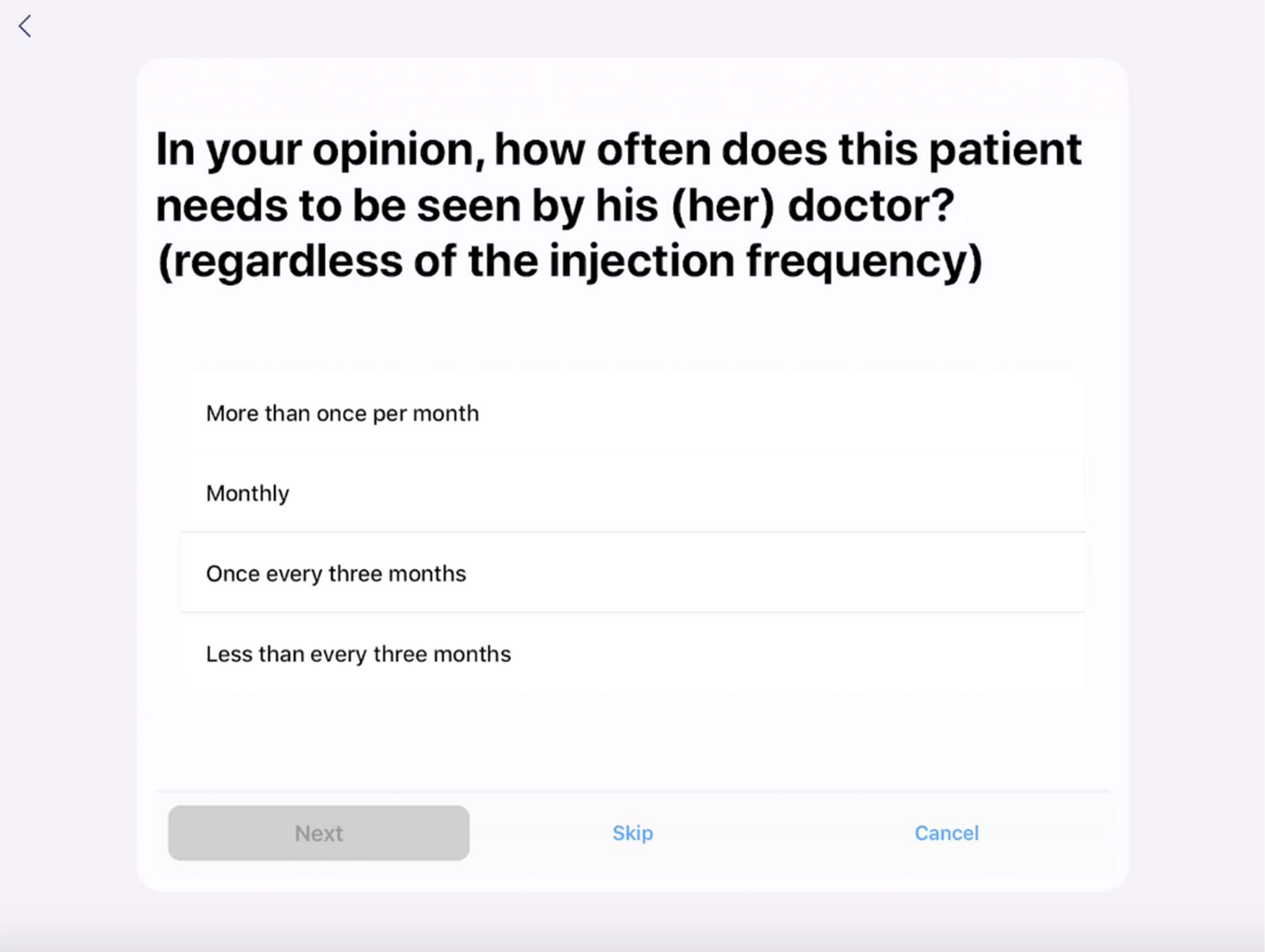
In your opinion, how often does this patient needs to be seen by his (her) doctor? (regardless of the injection frequency)
More than once per month
Monthly
Once every three months
Less than every three months
Next
Skip
Cancel

In your opinion, how often does this patient needs to be seen by his (her) nurse? (regardless of the injection frequency

More than once per month

Monthly

Once every three months

Less than every three months

Next

Skip

Cancel

How much do you talk with your patient about topics other than medication as a result of switching to PP3M?

Talk more

Talk less

Same amount

Next

Skip

Cancel

If "Talk More":

Please indicate topics that you were able to discuss more with your patient as a result of switching to PP3M? (multiple answers) Scroll to view all

- Family relationships
- Work
- Education
- External/ social communication
- Hobbies
- Healthy life style guidance (e.g. alcohol, smoking, diet, physical exercise)
- Non medication therapy (e.g. psychotherapy)
- Other topics

Next Skip Cancel

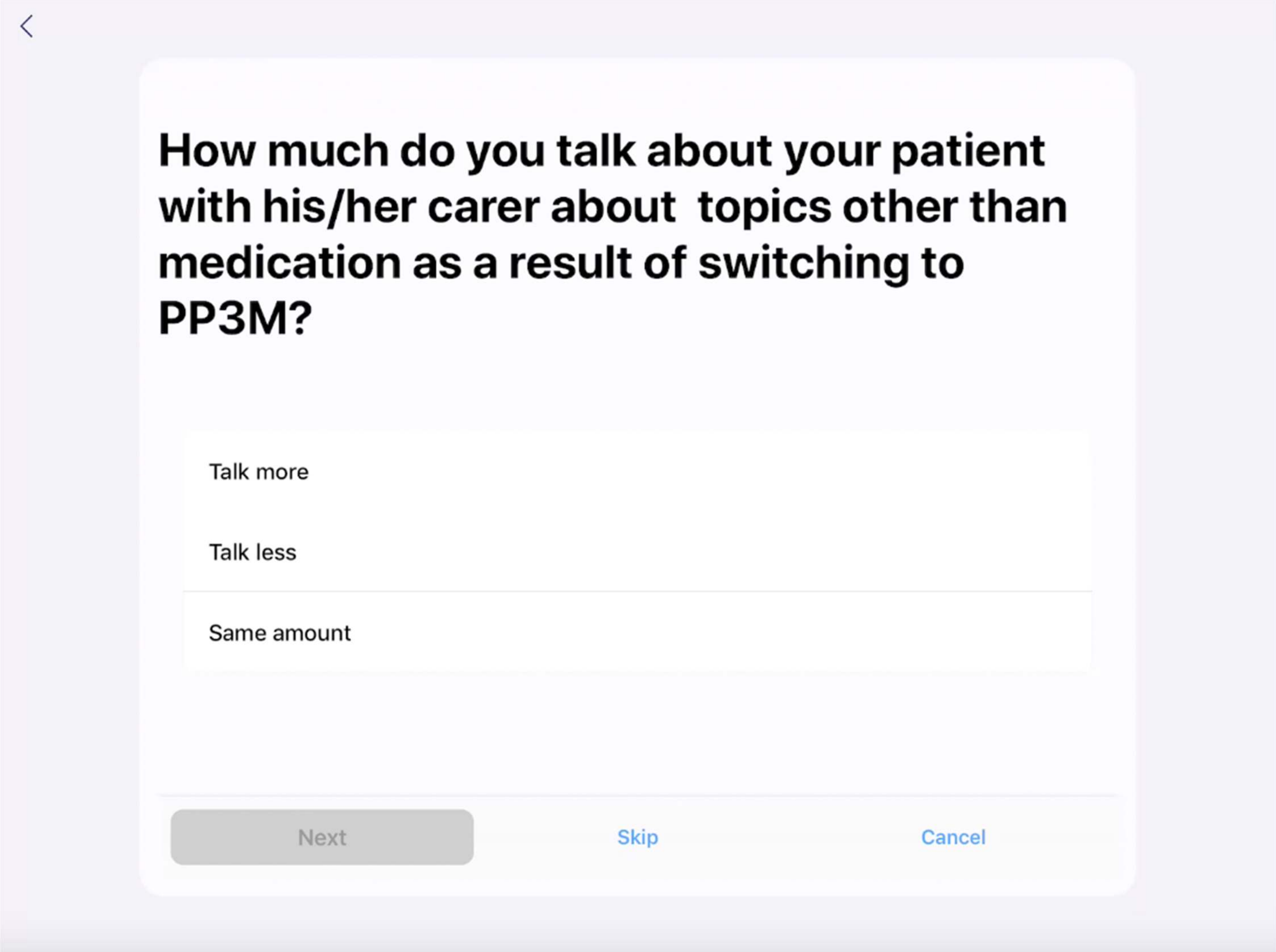
How much do you talk about your patient with his/her carer about topics other than medication as a result of switching to PP3M?
Talk more
Talk less
Same amount
Next
Skip
Cancel

If "Talk More":

Please indicate topics that you were able to discuss more (about the patient) with the patients carer as a result of switching to PP3M? (multiple answers) Scroll to view all

- Patients family relationships
- Patients work
- Patients education
- Patients external/ social communication
- Patients hobbies
- Patients healthy life style guidance (e.g. alcohol, smoking, diet, physical exercise)
- Patients non medication therapy (e.g. psychotherapy)
- Other topics

Next | Skip | Cancel

Has the quality of your communication with the patient changed as a result of switching to PP3M?

It has improved

It has not changed

It has worsened

Next

Skip

Cancel

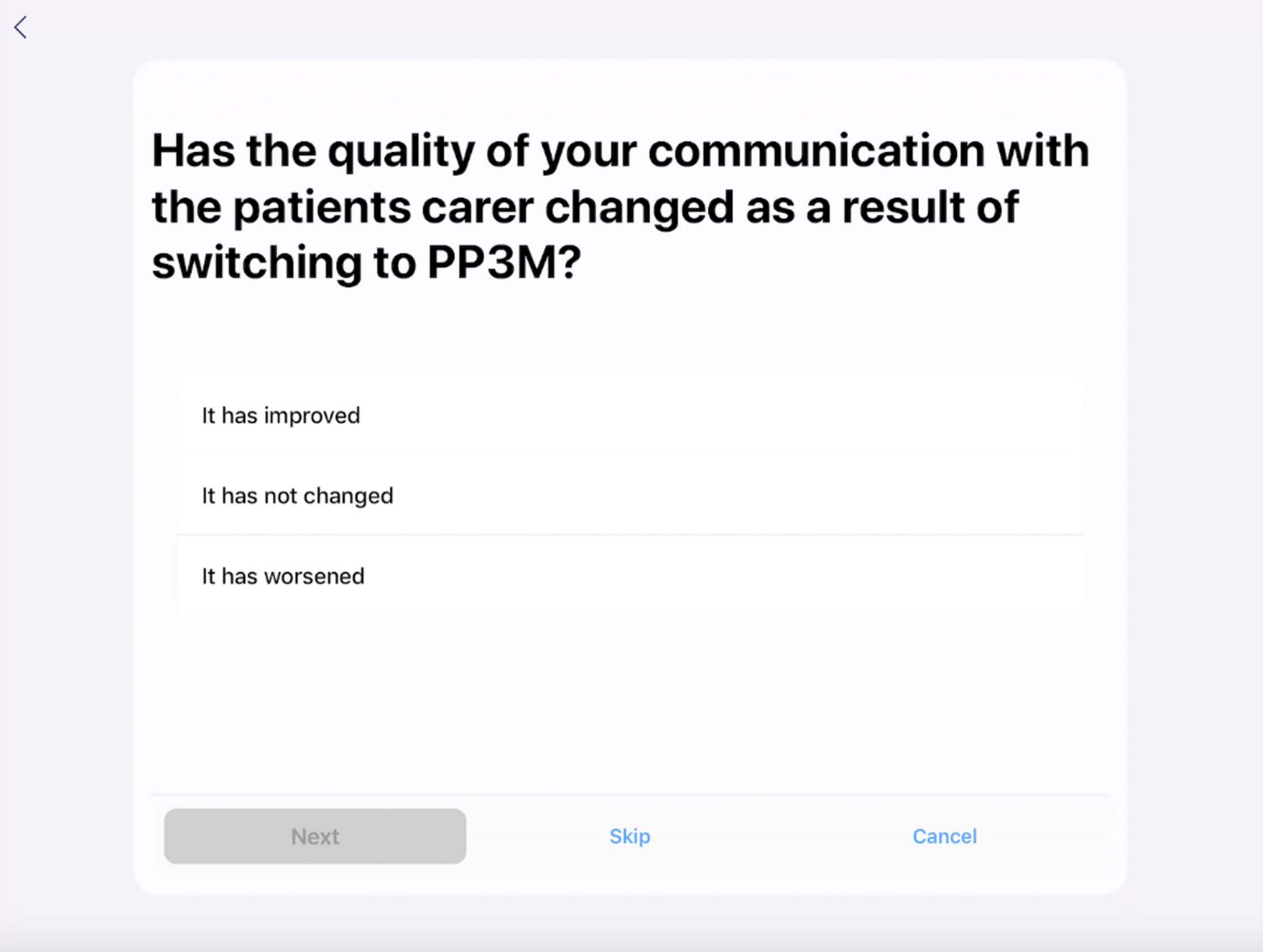
Has the quality of your communication with the patients carer changed as a result of switching to PP3M?
It has improved
It has not changed
It has worsened
Next
Skip
Cancel

To what extent were the nurse, patient and carer involved in the decision to switch from PP1M to PP3M? Patient was:

Highly involved

Involved to some extent

Not involved at all

Next   Skip   Cancel

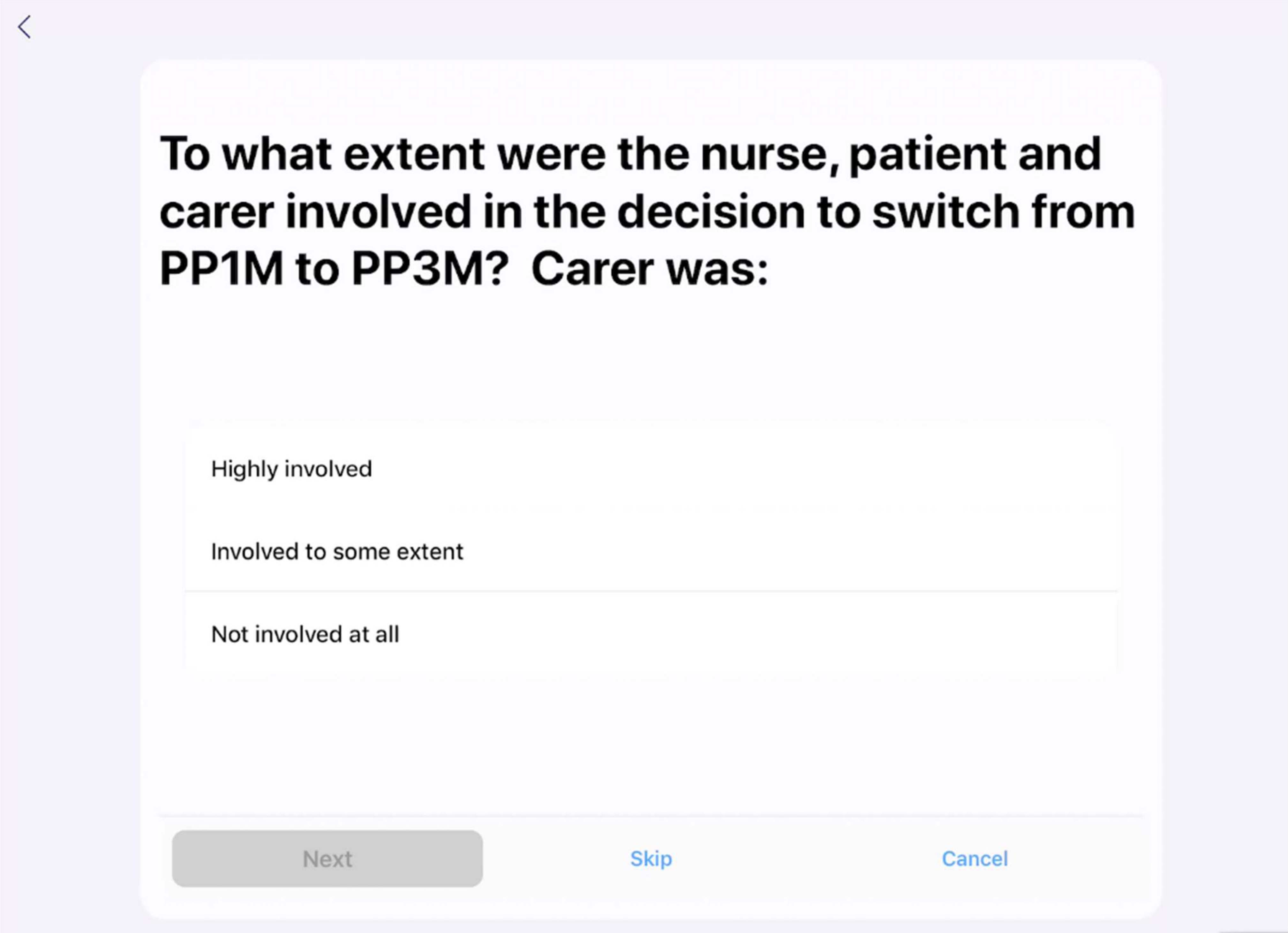
To what extent were the nurse, patient and carer involved in the decision to switch from PP1M to PP3M? Carer was:
Highly involved
Involved to some extent
Not involved at all
Next
Skip
Cancel

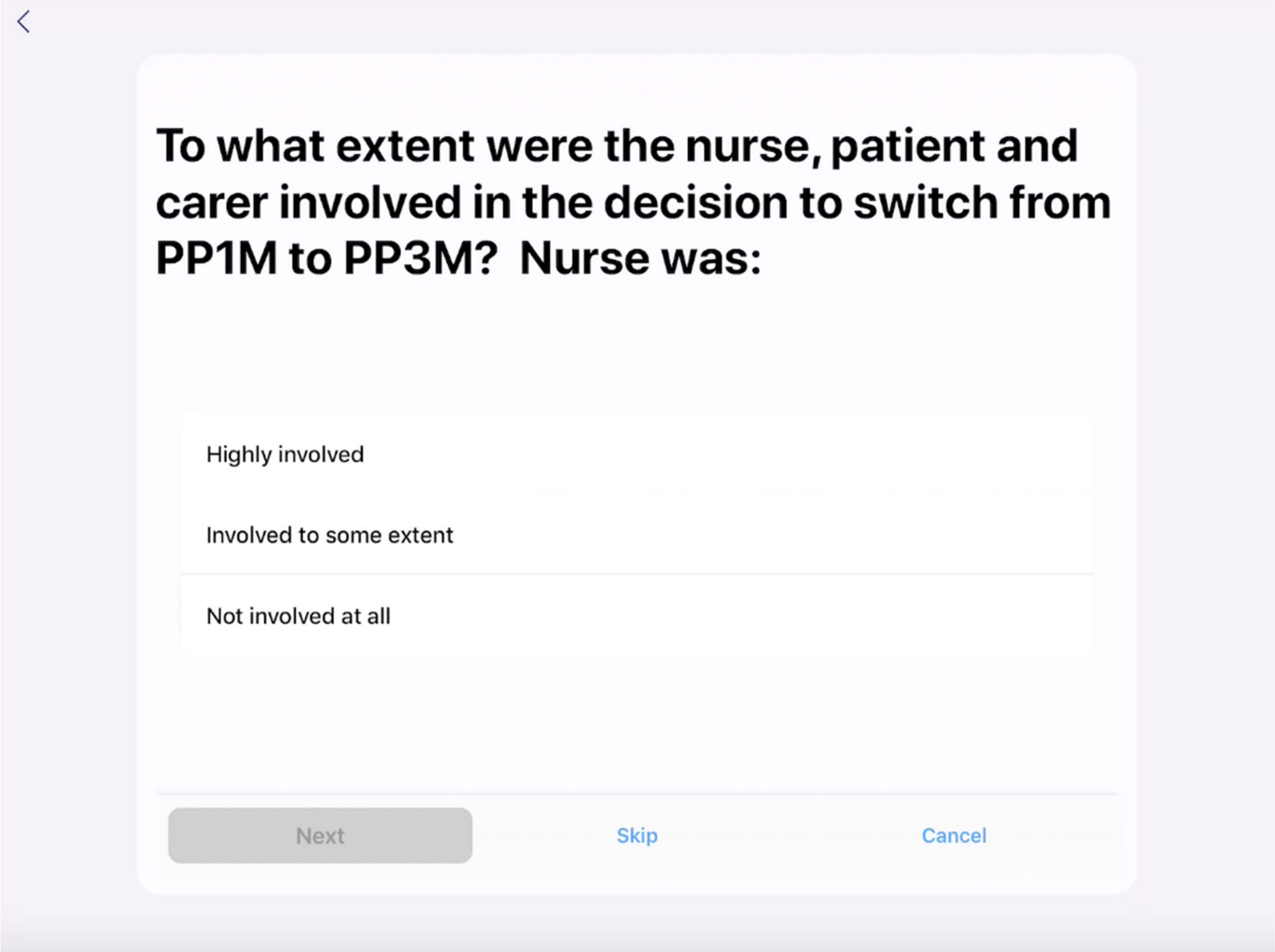
To what extent were the nurse, patient and carer involved in the decision to switch from PP1M to PP3M? Nurse was:
Highly involved
Involved to some extent
Not involved at all
Next
Skip
Cancel

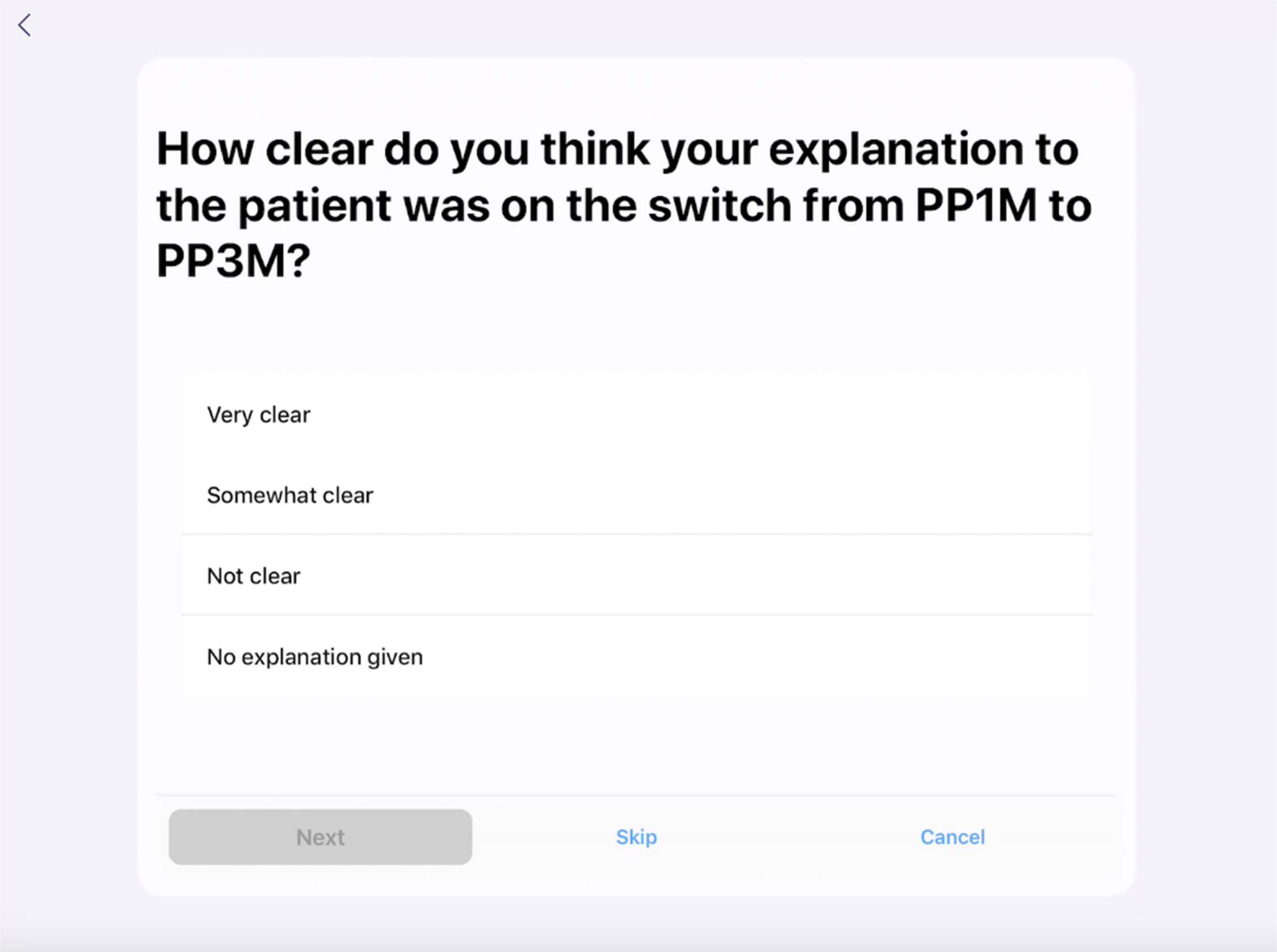
How clear do you think your explanation to the patient was on the switch from PP1M to PP3M?
Very clear
Somewhat clear
Not clear
No explanation given
Next
Skip
Cancel

Who initiated discussion about switching from PP1M to PP3M?

- It was me
- The nurse
- The patient
- The carer
- Private service

Next Skip Cancel

What were the main reasons to initiate/switch treatment of this patient to PP3M? Please check all that apply. Scroll to view all

To live a life that is as normal as possible

To reduce side effects

To improve treatment efficacy

To improve treatment adherence

To decrease stigma

Patient convenience

Reduce the risk of relapse

Medical staff convenience

Next

Skip

Cancel

What was the MOST important reason to initiate/switch treatment of this patient to PP3M? Scroll to view all

To live a life that is as normal as possible

To reduce side effects

To improve treatment efficacy

To improve treatment adherence

To decrease stigma

Patient convenience

Reduce the risk of relapse

Medical staff convenience

Next

Skip

Cancel

If there was more than one reason to initiate/switch treatment of this patient to PP3M, please select the SECOND MOST important reason here. Scroll to view all

- To live a life that is as normal as possible
- To reduce side effects
- To improve treatment efficacy
- To improve treatment adherence
- To decrease stigma
- Patient convenience
- Reduce the risk of relapse
- Medical staff convenience

Next | Skip | Cancel

‹

If there was more than one reason to initiate/switch treatment of this patient to PP3M, please select the THIRD MOST important reason here. Scroll to view all

To live a life that is as normal as possible

To reduce side effects

To improve treatment efficacy

To improve treatment adherence

To decrease stigma

Patient convenience

Reduce the risk of relapse

Medical staff convenience

Next | Skip | Cancel

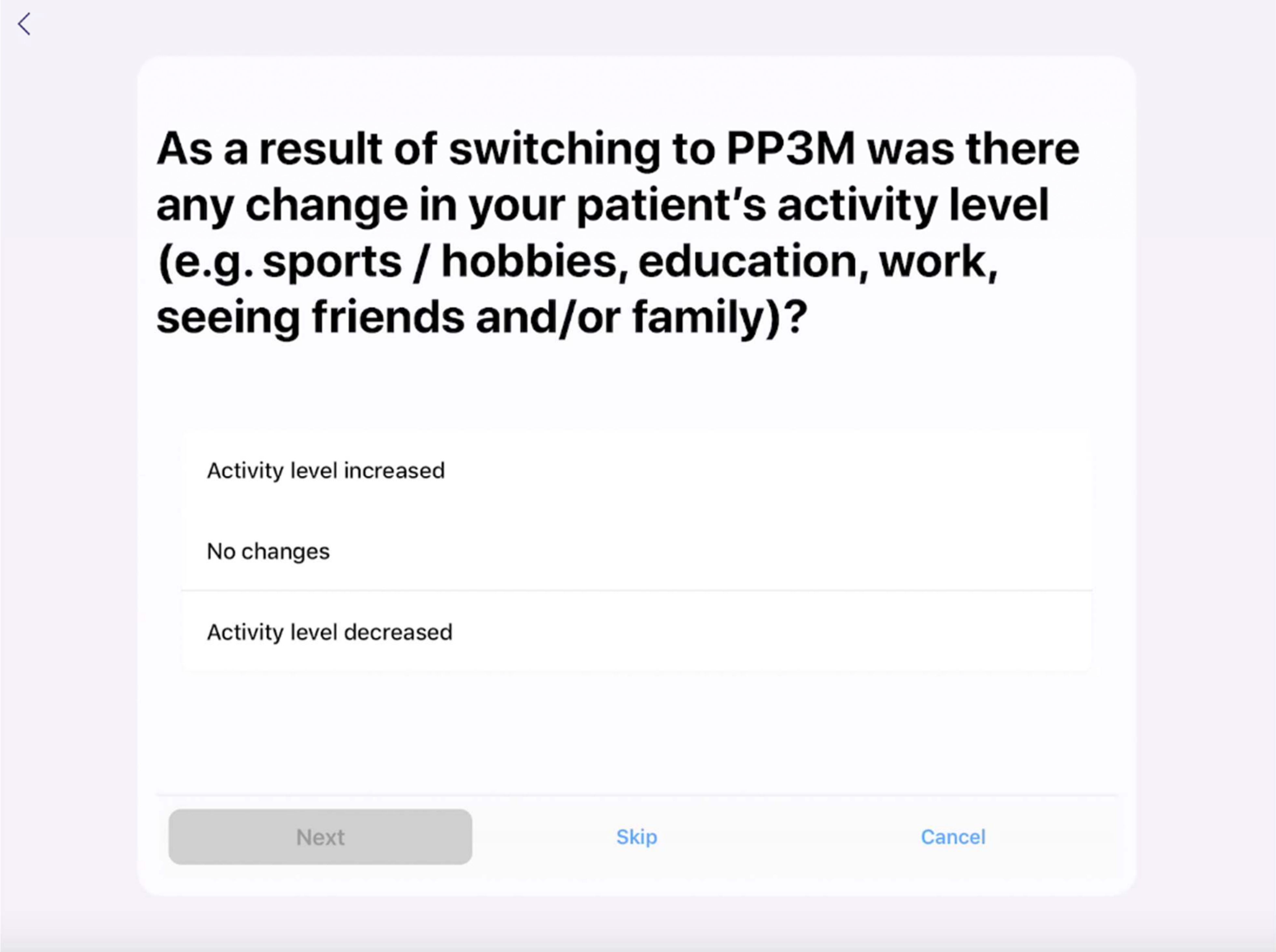
As a result of switching to PP3M was there any change in your patient's activity level (e.g. sports / hobbies, education, work, seeing friends and/or family)?
Activity level increased
No changes
Activity level decreased
Next
Skip
Cancel

If “Activity Level Increased”:

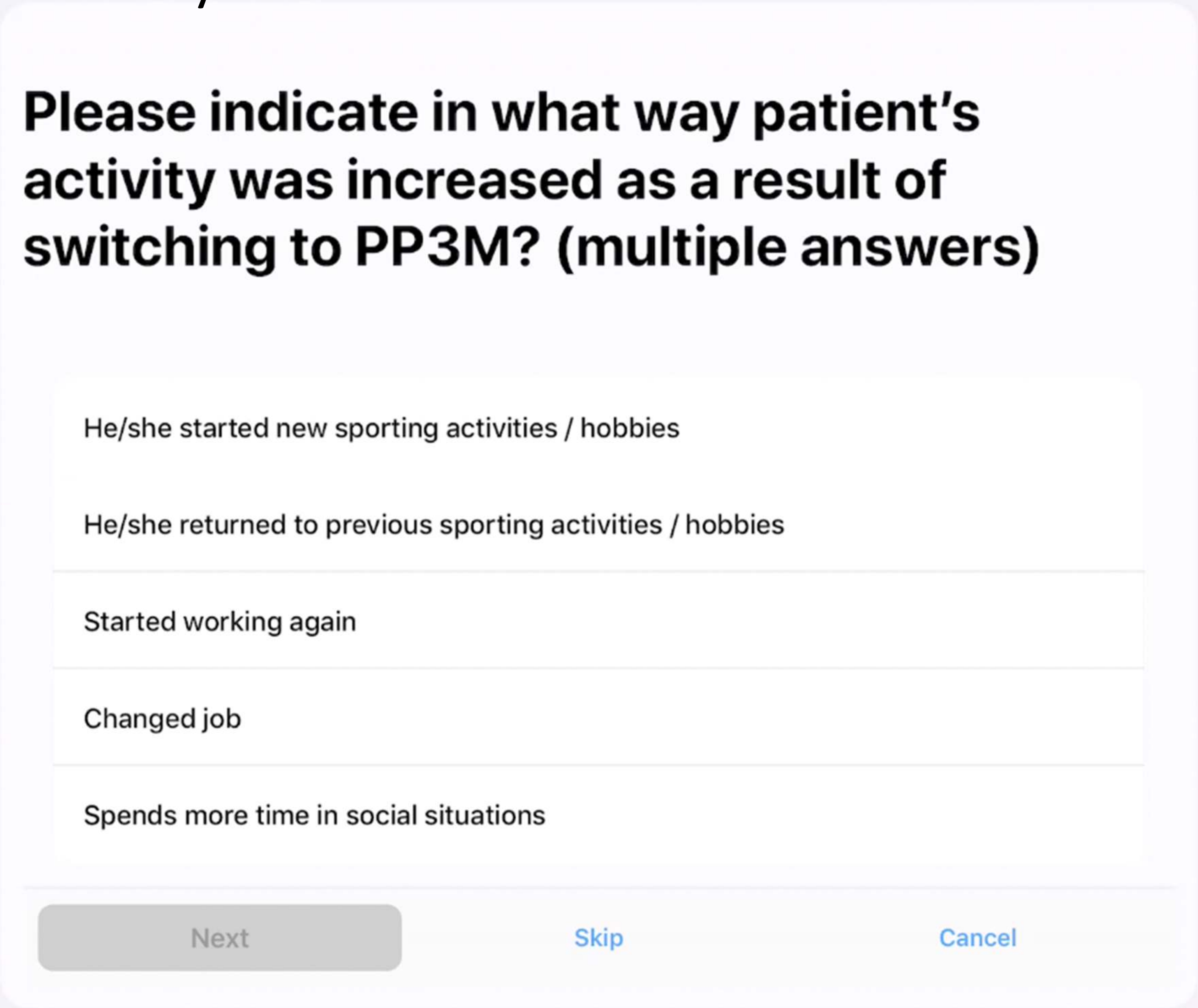

As a result of switching to PP3M have you noticed any change in the frequency of your patient's communications with his/her family, friends and/or other people?

He/she sees and meets his/her friends/ family members/ other people more often

He/she sees and meets his/her friends/ family members/ other people with the same frequency

He/she sees and meets his/her friends/ family members/ other people less often

Next

Skip

Cancel

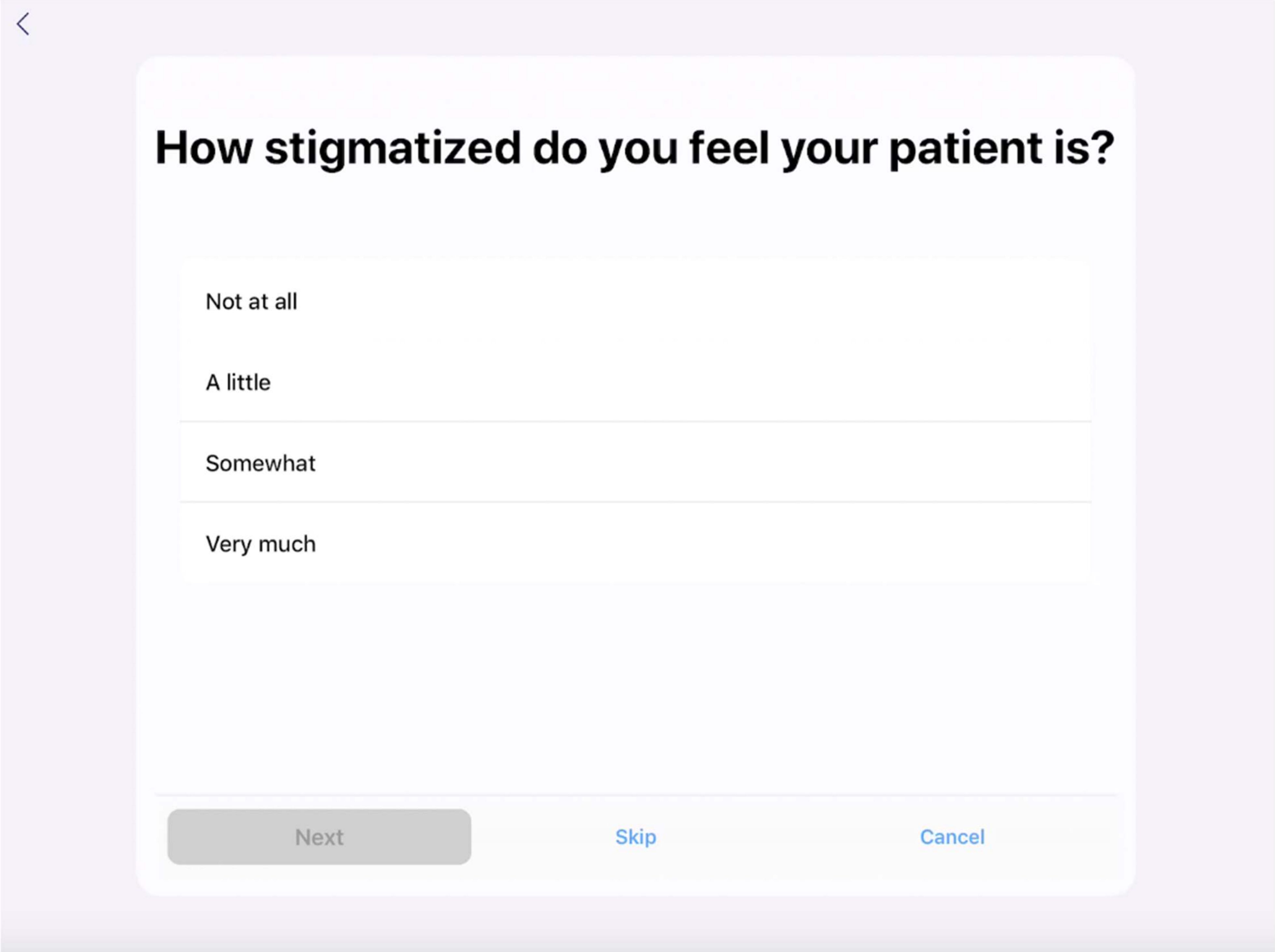
How stigmatized do you feel your patient is?
Not at all
A little
Somewhat
Very much
Next
Skip
Cancel

To what extent do you think that PP3M impacts on your patients feeling of being stigmatized?

No impact

Increases stigma

Decreases stigma

Next

Skip

Cancel

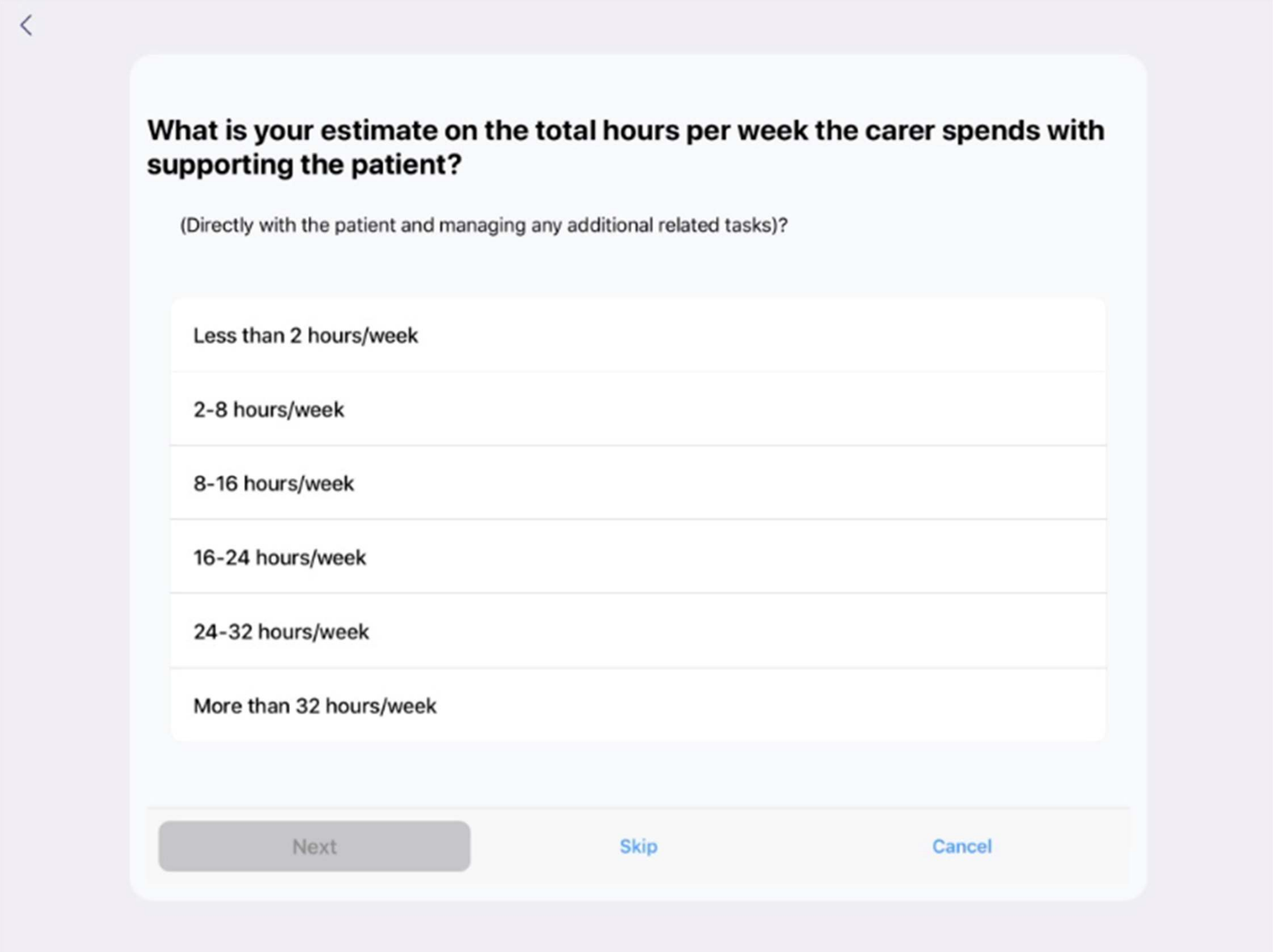
What is your estimate on the total hours per week the carer spends with supporting the patient?
(Directly with the patient and managing any additional related tasks)?
Less than 2 hours/week
2-8 hours/week
8-16 hours/week
16-24 hours/week
24-32 hours/week
More than 32 hours/week
Next
Skip
Cancel

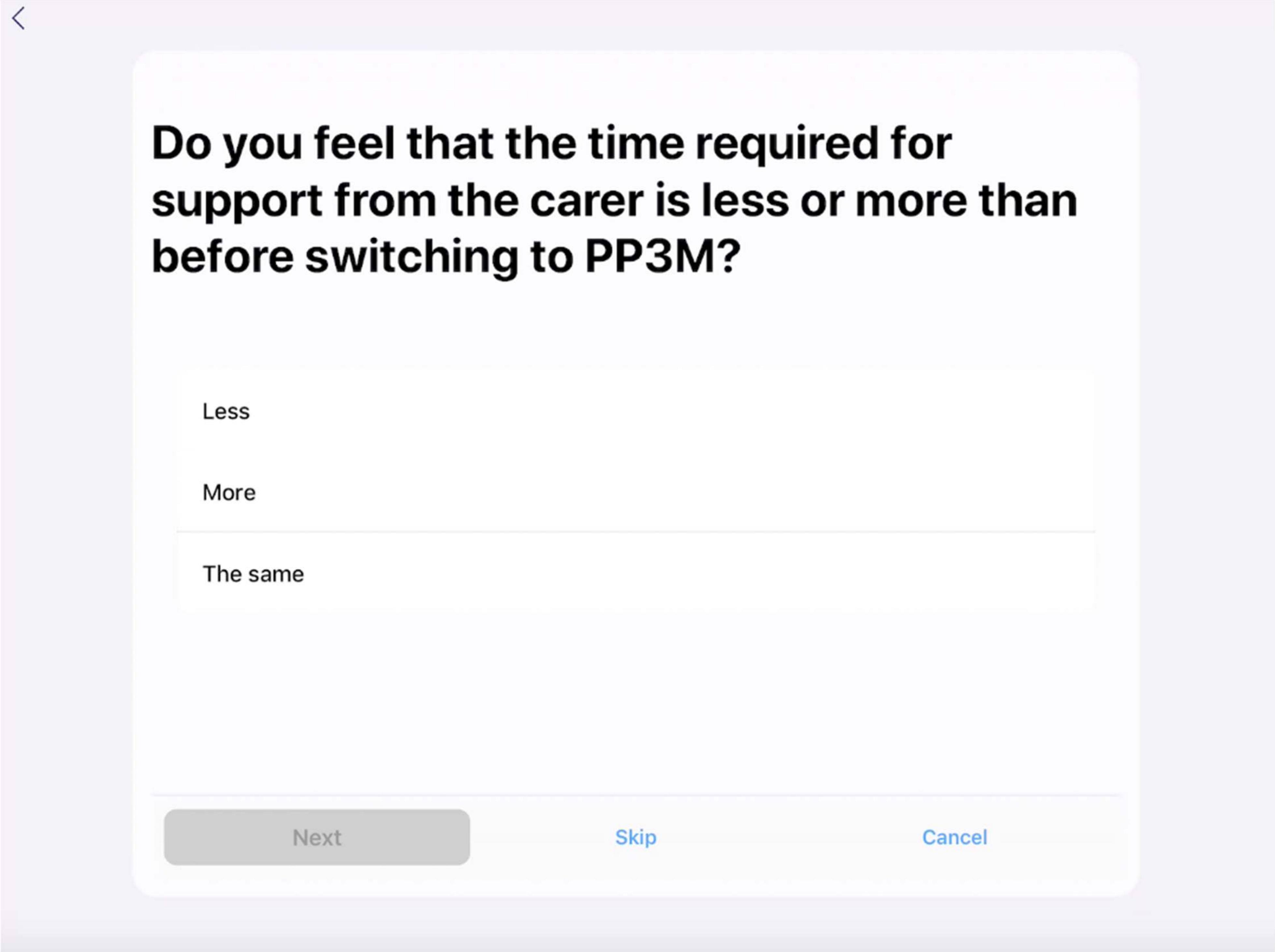
Do you feel that the time required for support from the carer is less or more than before switching to PP3M?
Less
More
The same
Next
Skip
Cancel

As a result of switching to PP3M have you noticed/ felt any change in the amount of support needed for your patient from their carer?

I feel the patient needs less support from his/her carer to take care of himself/herself

I feel the patient needs the same amount of support from his/her carer to take care of himself/herself

I feel the patient needs more support from his/her carer to take care of himself/herself

Next

Skip

Cancel

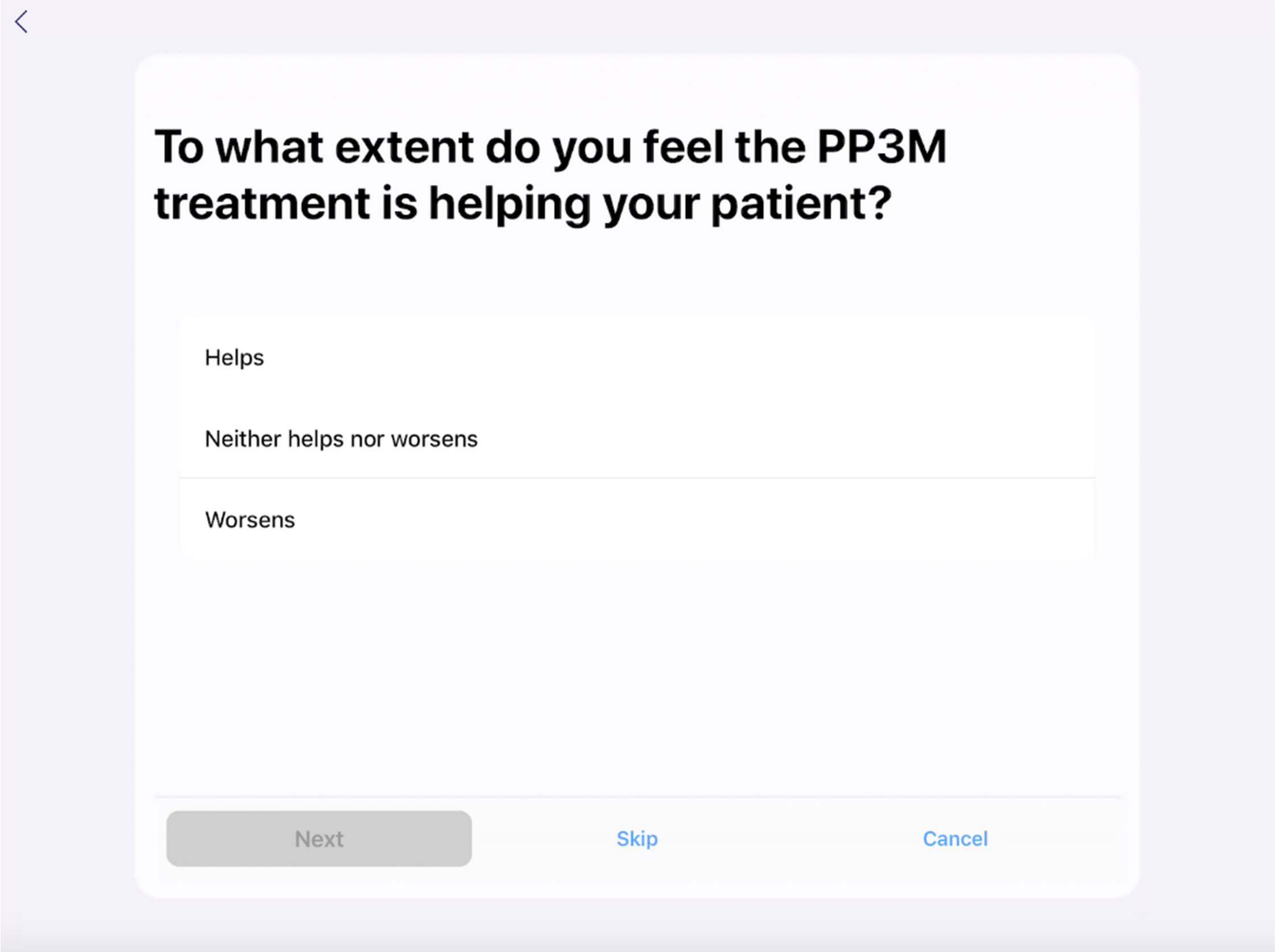
To what extent do you feel the PP3M treatment is helping your patient?
Helps
Neither helps nor worsens
Worsens
Next
Skip
Cancel

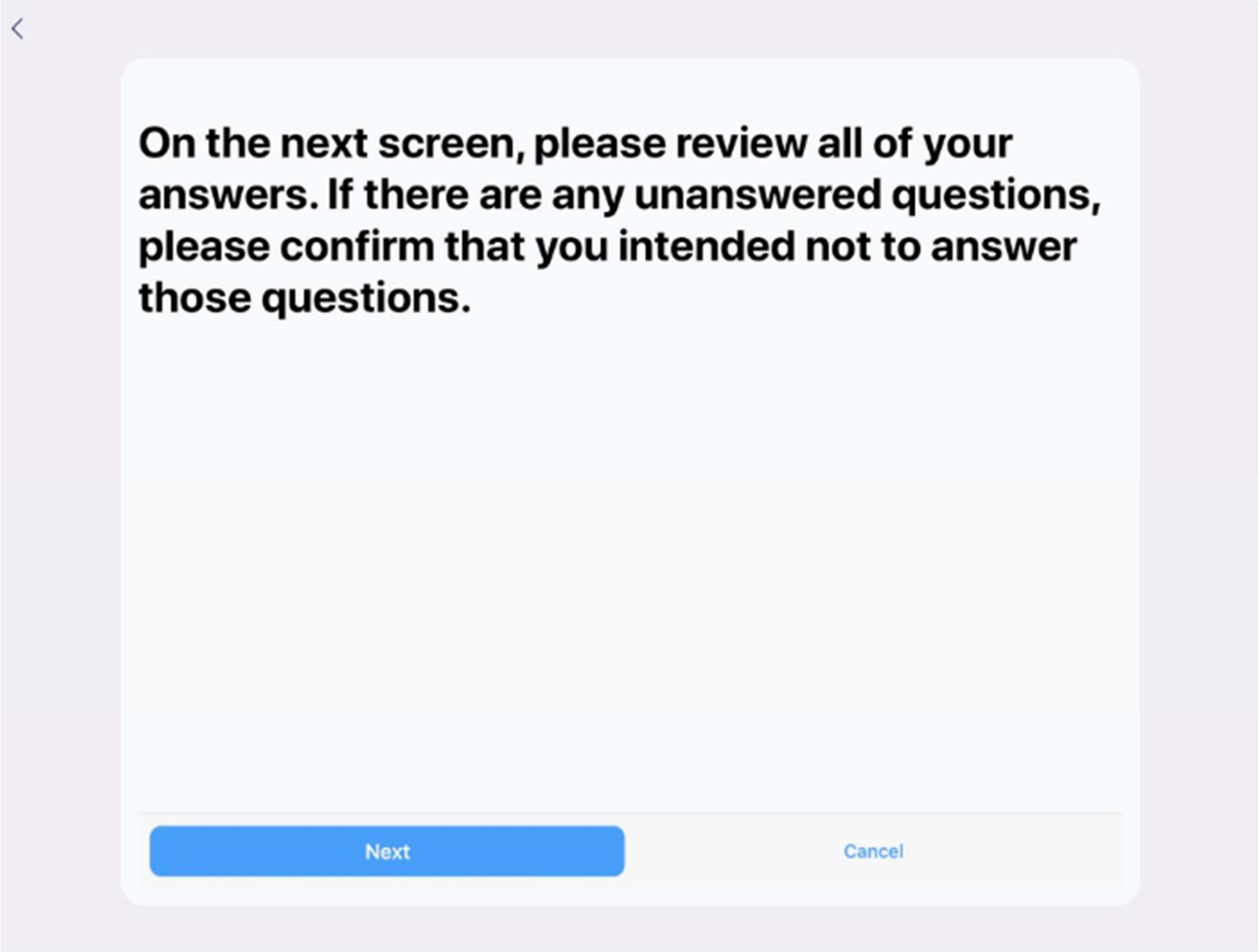
On the next screen, please review all of your answers. If there are any unanswered questions, please confirm that you intended not to answer those questions.
Next
Cancel

Please review your answers. Pressing “Next” will submit your answers

You can jump directly to update question by clicking on it in the list below

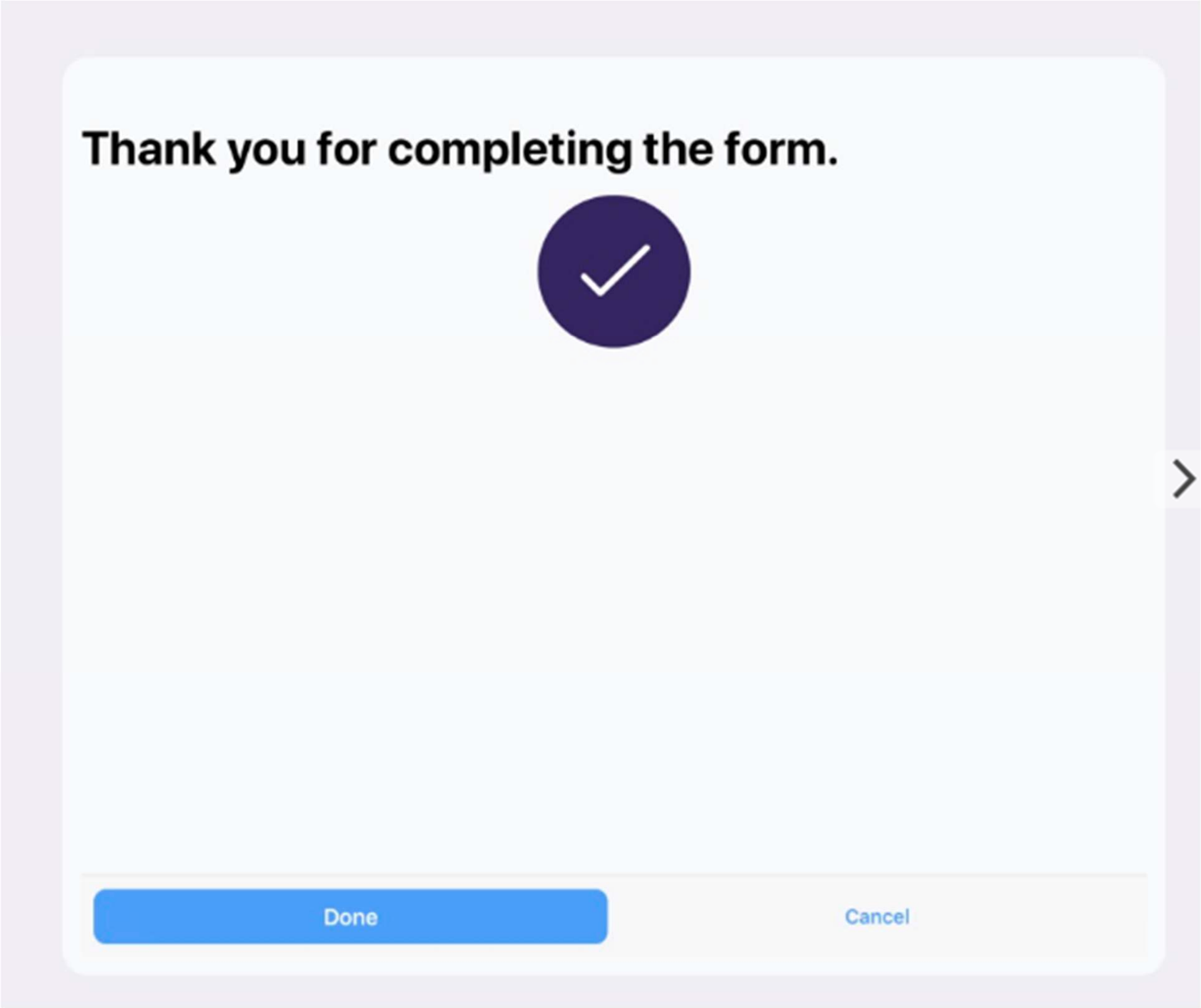
Thank you for completing the form.
Done
Cancel
