# Supplementary material for: The Patient, Investigator, Nurse, Carer Questionnaire (PINC-Q): a cross-sectional, retrospective, non-interventional study exploring the impact of less frequent medication administration with paliperidone palmitate 3-monthly as maintenance treatment for schizophrenia
Source: BMC Psychiatry. 2021 Jun 9;21:300. doi: 10.1186/s12888-021-03305-z (PMC8191017; doi:10.1186/s12888-021-03305-z)
Supplement: Supplementary file 5 — Additional file 5. English nurse one time questionnaire. [file 12888_2021_3305_MOESM5_ESM.pdf]

English Nurse Survey One Time

Welcome to the One Time Nurse Survey. The following questions will ask about you and the care you provide to patients. You only need to complete all questions 1 time.

Get Started

Cancel

Age

Tap to answer

Next

Cancel

Gender

Male

Female

Next

Cancel

Practice Setting (please select the facility you are working in):

- Inpatient facility (i.e. ward for >24- hours hospitalizations)
- Outpatient facility
- Day hospital
- Other

Next Skip Cancel

Type of Facility (please select the option that applies):

Private Facility

Public Facility

Next

Skip

Cancel

Experience as a psychiatric nurse (in years)

- Less than 5
- 5-10
- 11-15
- >15

Next Skip Cancel

How many patients living with schizophrenia do you care for in a month?

Tap to answer

Next Skip Cancel

How many long acting injections (LAIs) do you administer for the treatment of patients with schizophrenia? (in a month)

Tap to answer

Next

Skip

Cancel

How many PP3M injections do you give in a month?

Tap to answer

Next Skip Cancel

Whom do you find important to involve in the treatment decision? (multiple answers)

- Patient
- Carer
- Doctor
- Nurse
- Other

Next Skip Cancel

On the next screen, please review all of your answers. If there are any unanswered questions, please confirm that you intended not to answer those questions.

Next

Cancel

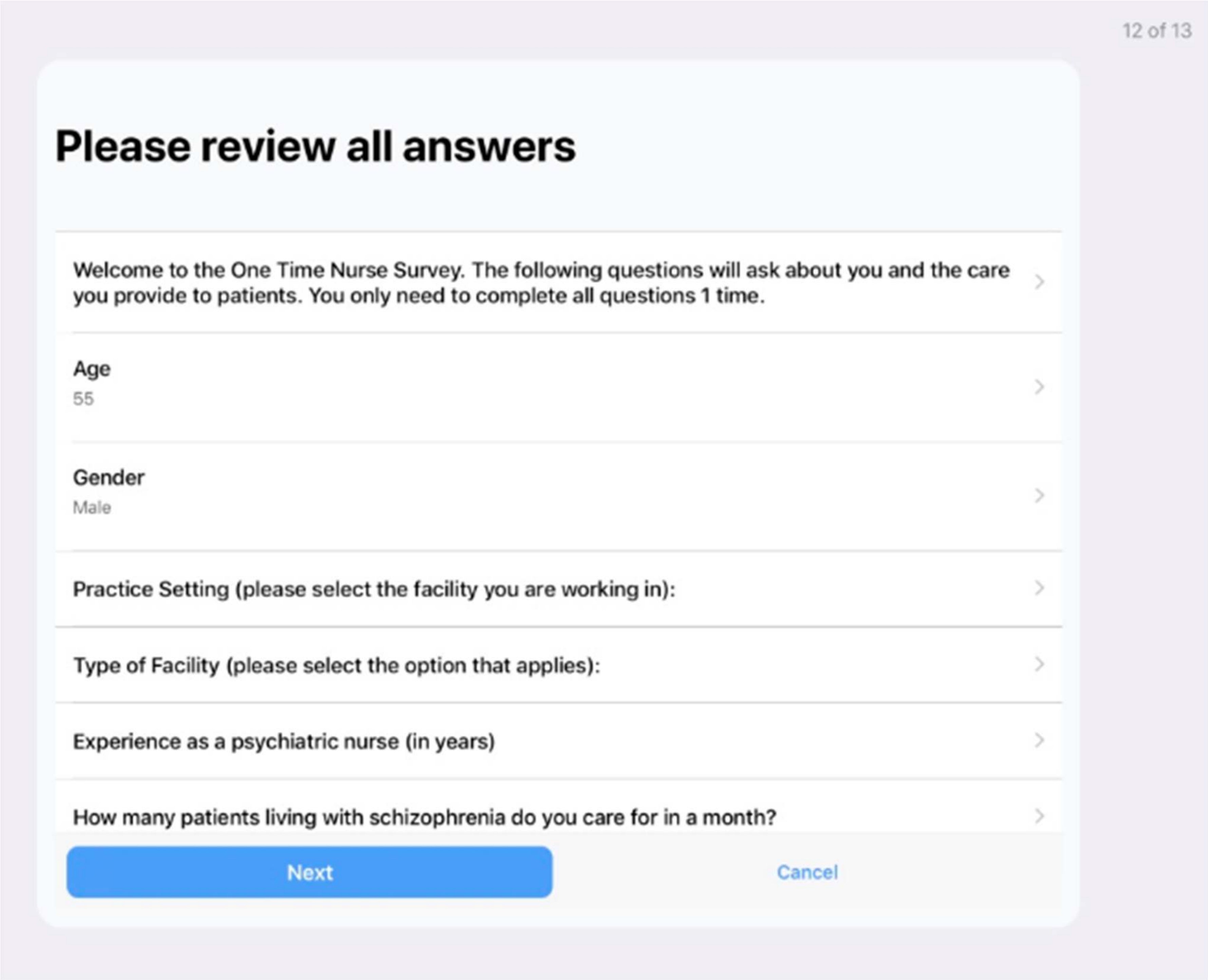
12 of 13
Please review all answers
Welcome to the One Time Nurse Survey. The following questions will ask about you and the care you provide to patients. You only need to complete all questions 1 time.
Age
55
Gender
Male
Practice Setting (please select the facility you are working in):
Type of Facility (please select the option that applies):
Experience as a psychiatric nurse (in years)
How many patients living with schizophrenia do you care for in a month?
Next
Cancel

Thank you for completing the form.

Done

Cancel
