# Supplementary material for: The Patient, Investigator, Nurse, Carer Questionnaire (PINC-Q): a cross-sectional, retrospective, non-interventional study exploring the impact of less frequent medication administration with paliperidone palmitate 3-monthly as maintenance treatment for schizophrenia
Source: BMC Psychiatry. 2021 Jun 9;21:300. doi: 10.1186/s12888-021-03305-z (PMC8191017; doi:10.1186/s12888-021-03305-z)
Supplement: Supplementary file 6 — Additional file 6. English nurse questionnaire. [file 12888_2021_3305_MOESM6_ESM.pdf]

English Nurse Survey

Welcome to the English Nurse Survey. This survey has approximately 30 questions, we anticipate it will take 10 minutes to complete.

Get Started

Cancel

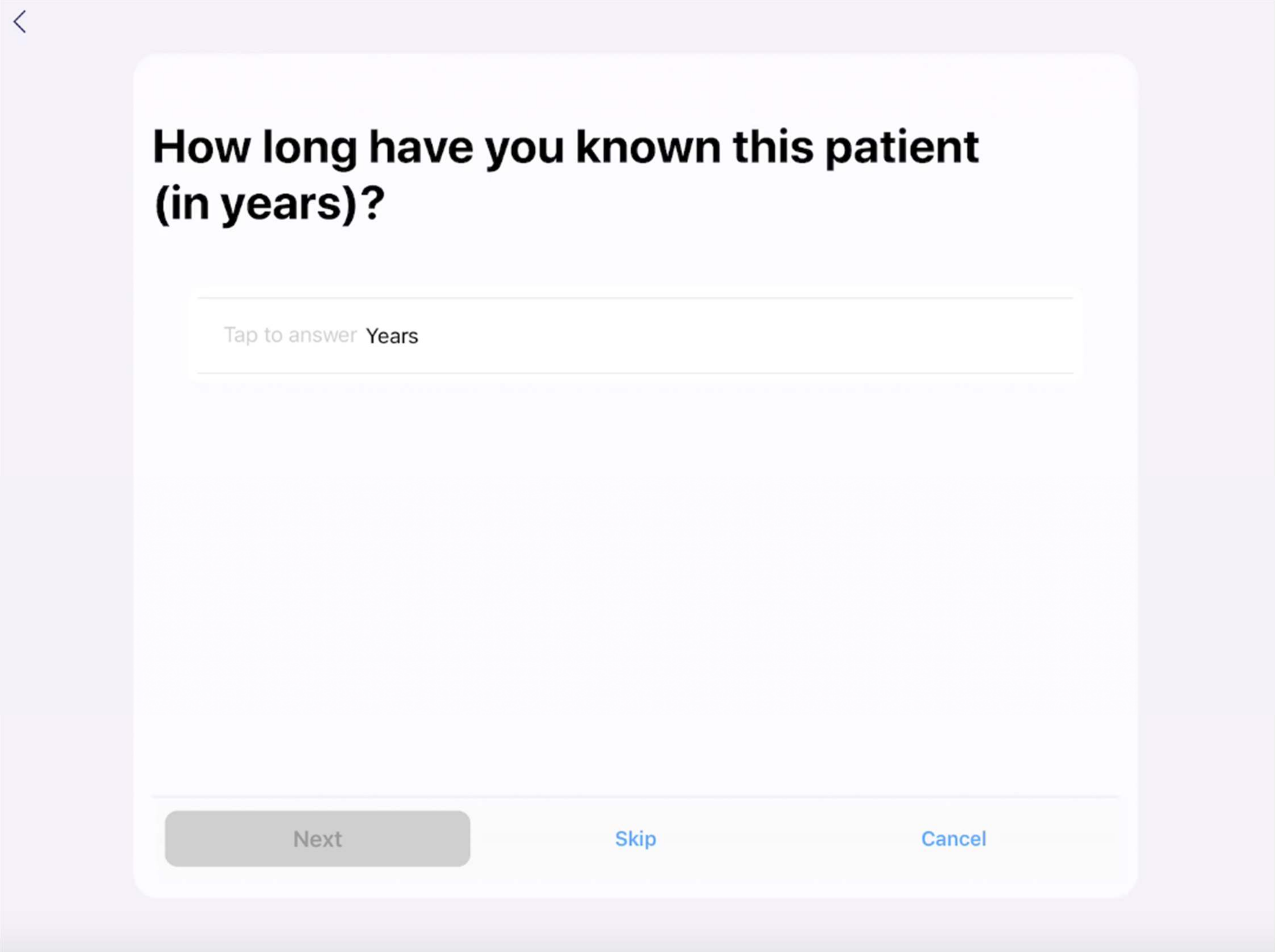
How long have you known this patient (in years)?
Tap to answer Years
Next
Skip
Cancel

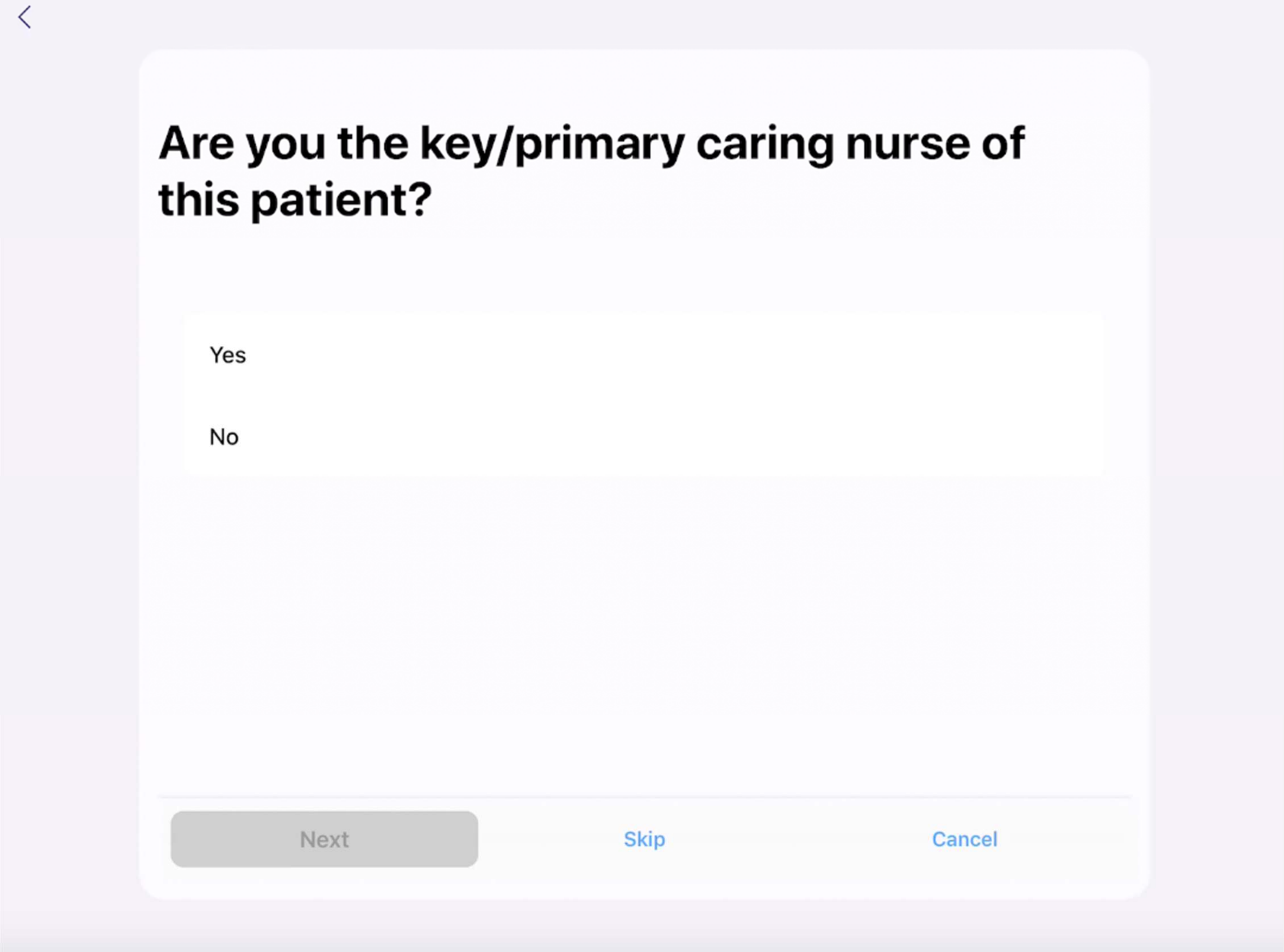
Are you the key/primary caring nurse of this patient?
Yes
No
Next
Skip
Cancel

The patient gets PP3M injection via:

Deltoid

Gluteal

Next

Skip

Cancel

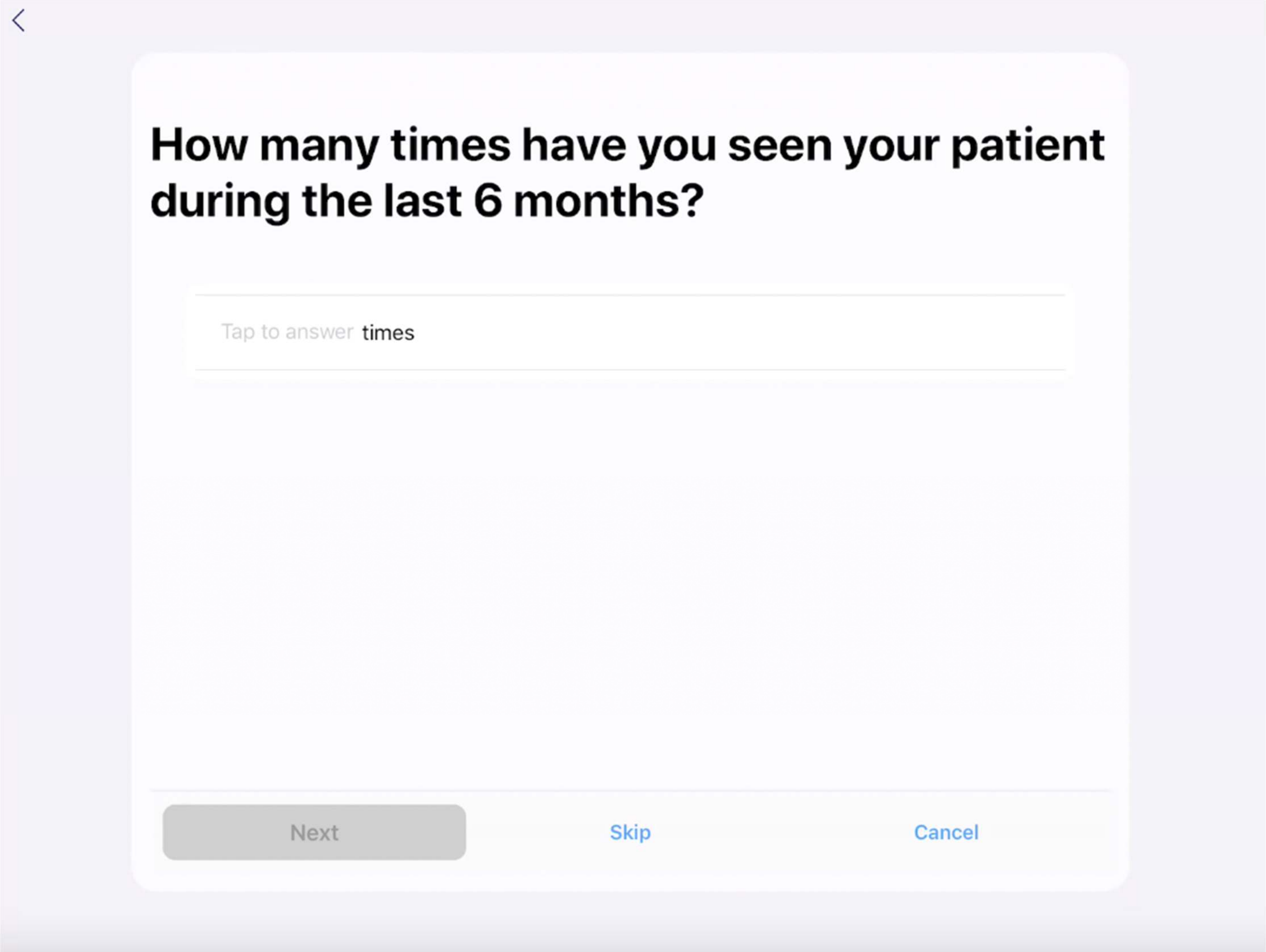
How many times have you seen your patient during the last 6 months?
Tap to answer times
Next
Skip
Cancel

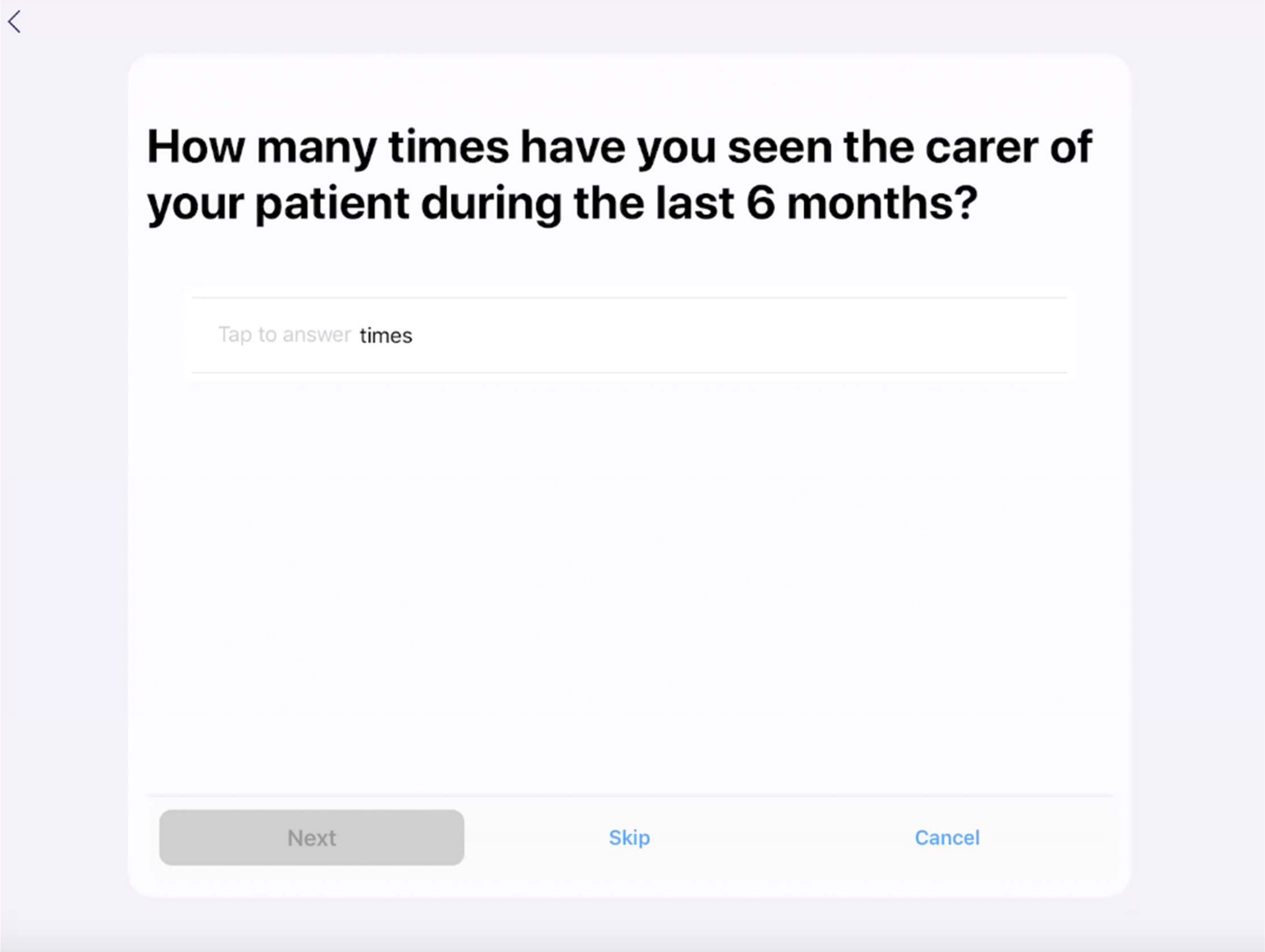
How many times have you seen the carer of your patient during the last 6 months?
Tap to answer times
Next
Skip
Cancel

Has the frequency in which you see your patient changed as a result of switching to PP3M?

- We meet more frequently
- We meet less frequently
- There is no change

Next   Skip   Cancel

How satisfied are you with the current frequency in which you see your patient?

Very Satisfied

Satisfied

Dissatisfied

Very Dissatisfied

Next

Skip

Cancel

Has the frequency in which you see the carer of your patient changed as a result of switching to PP3M?

We meet more frequently

We meet less frequently

There is no change

Next

Skip

Cancel

How satisfied are you with the current frequency in which you see the carer of your patient?

Very Satisfied

Satisfied

Dissatisfied

Very Dissatisfied

Next

Skip

Cancel

In your opinion, how often does this patient needs to be seen by his (her) doctor? (regardless of the injection frequency)

- More than once per month
- Monthly
- Once every three months
- Less than every three months

Next | Skip | Cancel

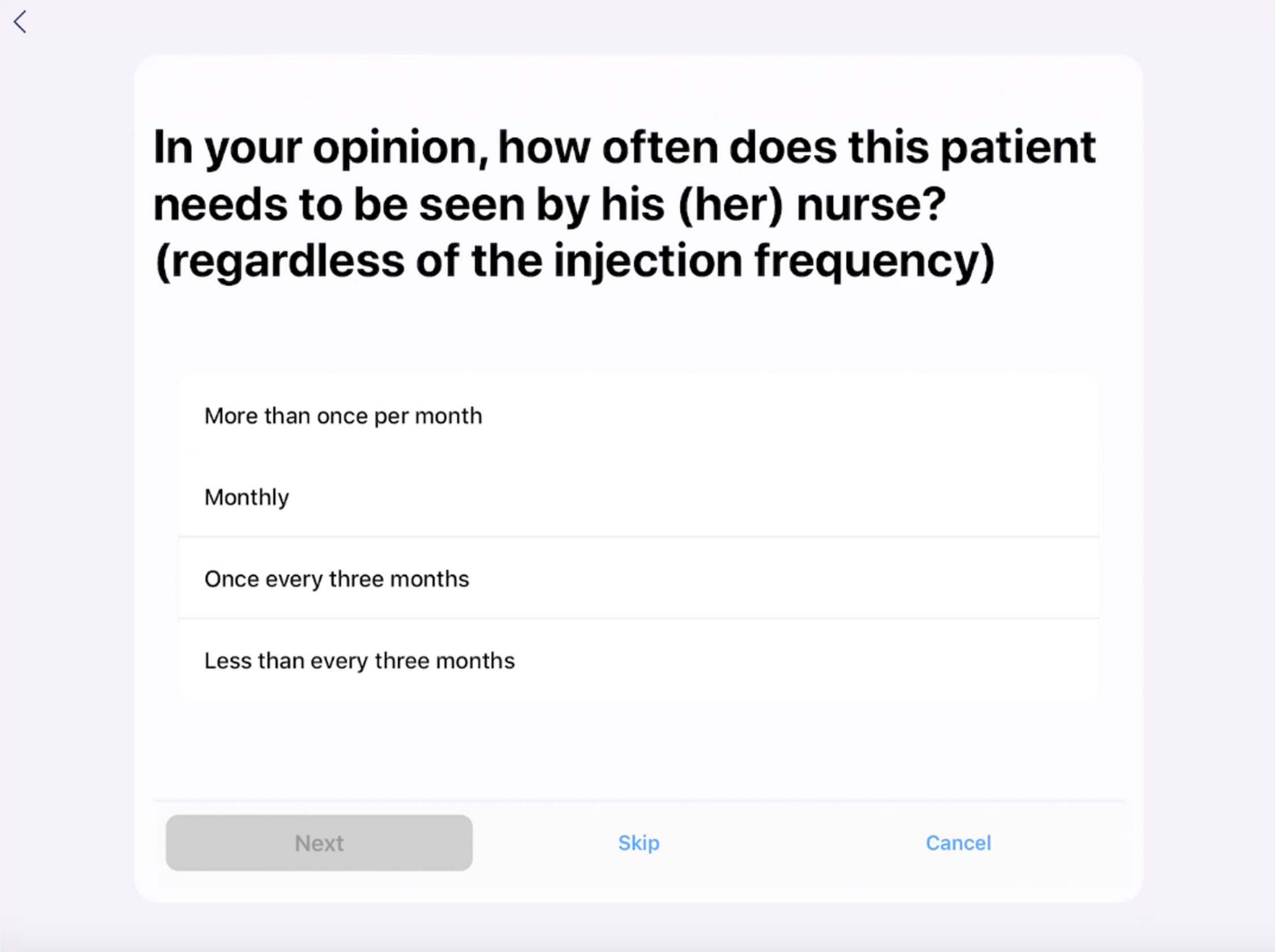
In your opinion, how often does this patient needs to be seen by his (her) nurse? (regardless of the injection frequency)
More than once per month
Monthly
Once every three months
Less than every three months
Next
Skip
Cancel

How much do you talk with your patient about topics other than medication as a result of switching to PP3M?

Talk more

Talk less

Same amount

Next

Skip

Cancel

If “TALK MORE”;

Please indicate topics that you were able to discuss more with your patient as a result of switching to PP3M? (multiple answers)

- Family relationships
- Work
- Education
- External/social communication
- Hobbies
- Healthy life style guidance (e.g. alcohol, smoking, diet, physical exercise)
- Non medication therapy (e.g. psychotherapy)
- Other topics

Next Skip Cancel

How much do you talk about your patient with his/her carer about topics other than medication as a result of switching to PP3M?

Talk more

Talk less

Same amount

Next   Skip   Cancel

If “TALK MORE”:

Please indicate topics that you were able to discuss more with your patients carer as a result of switching to PP3M? (multiple answers)

- Patients family relationships
- Patients work
- Patients education
- Patients external/social communication
- Patients healthy life style guidance (e.g. alcohol, smoking, diet, physical exercise)
- Patients non medication therapy (e.g. psychotherapy)
- Other topics

Next Skip Cancel

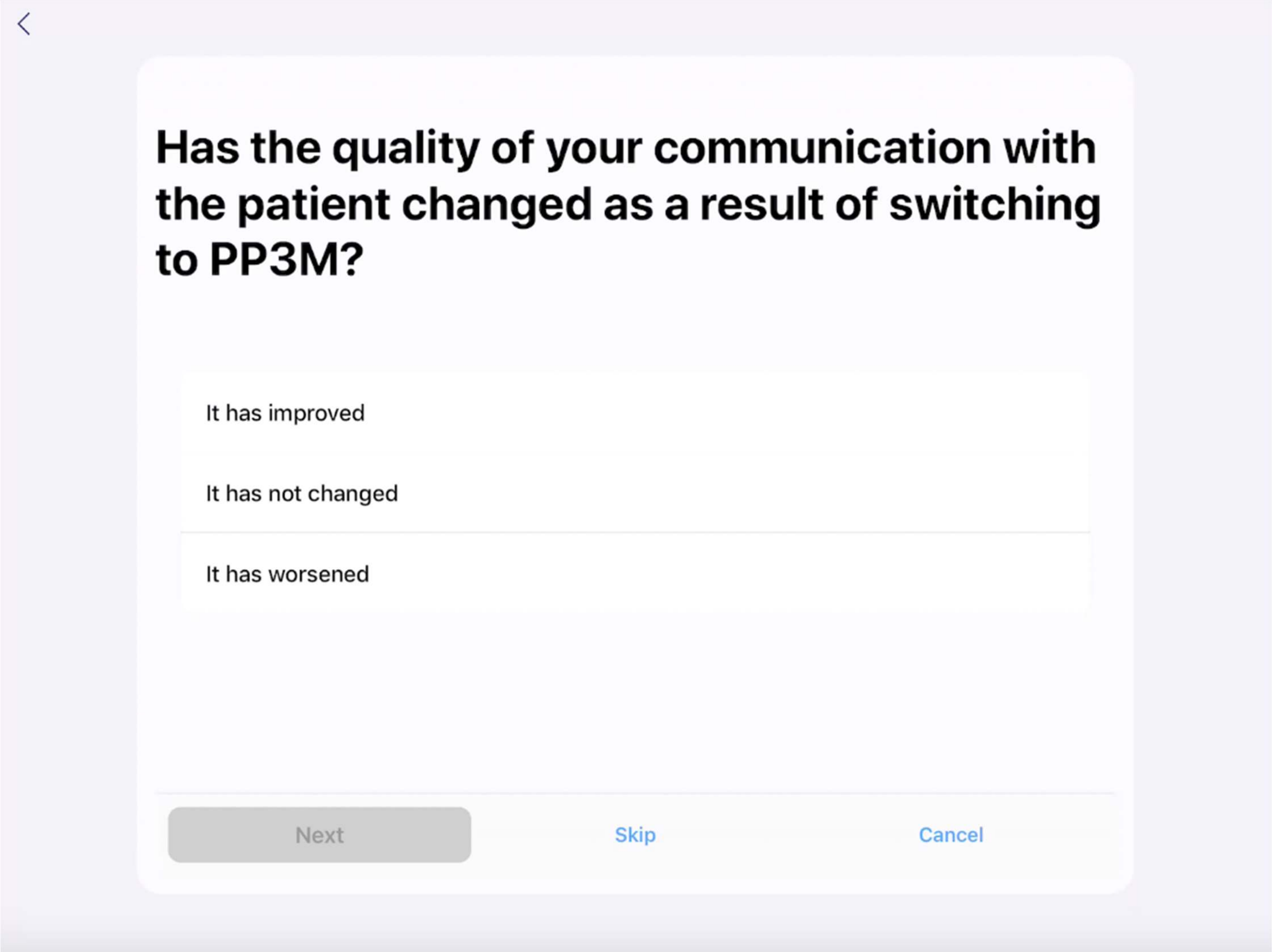
Has the quality of your communication with the patient changed as a result of switching to PP3M?
It has improved
It has not changed
It has worsened
Next
Skip
Cancel

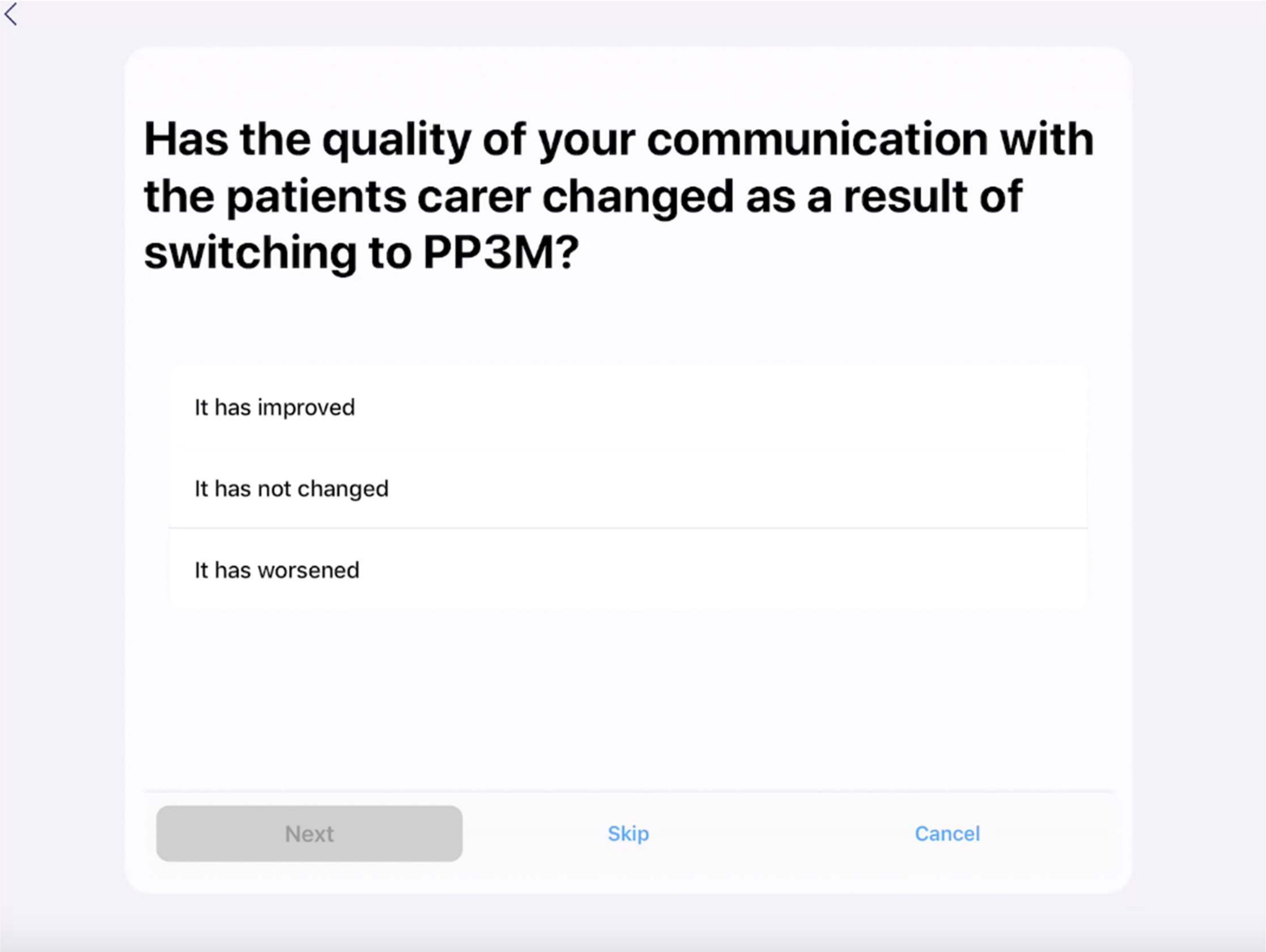
Has the quality of your communication with the patients carer changed as a result of switching to PP3M?
It has improved
It has not changed
It has worsened
Next
Skip
Cancel

To what extent were you involved in the decision to switch from PP1M to PP3M?

Highly involved

Involved to some extent

Not involved at all

Next

Skip

Cancel

How clear do you think your explanation to the patient was on the switch from PP1M to PP3M?

Very clear

Somewhat clear

Not clear

No explanation given

Next Skip Cancel

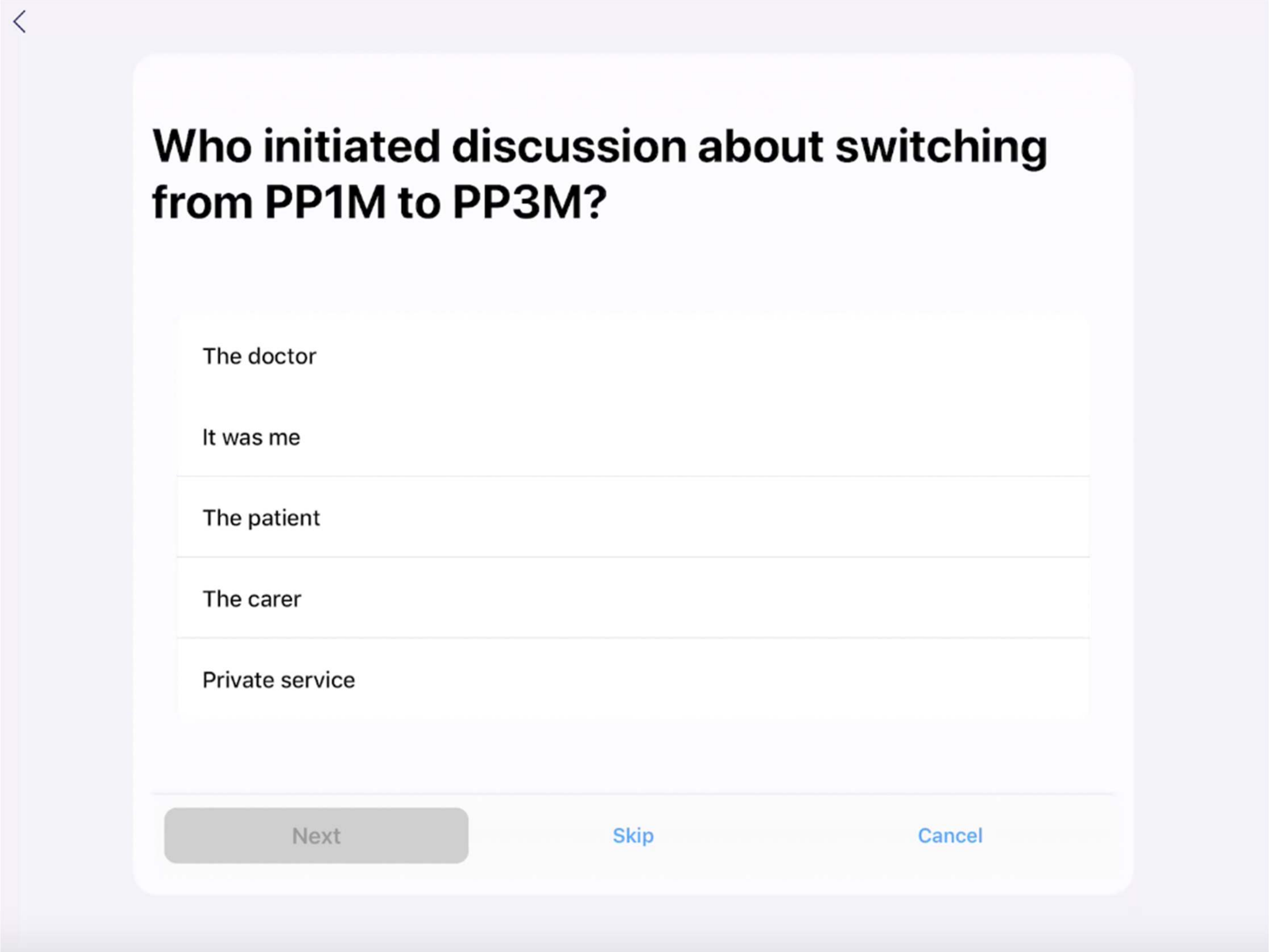
Who initiated discussion about switching from PP1M to PP3M?
The doctor
It was me
The patient
The carer
Private service
Next
Skip
Cancel

What were the main reasons to initiate/switch treatment of this patient to PP3M? Please check all that apply. Scroll to view all

To live a life that is as normal as possible

To reduce side effects

To improve treatment efficacy

To improve treatment adherence

To decrease stigma

Patient convenience

Reduce the risk of relapse

Medical staff convenience

Next | Skip | Cancel

What was the MOST important reason to initiate/switch treatment of this patient to PP3M? Only use reasons selected in previous question. Scroll to view all

To live a life that is as normal as possible

To reduce side effects

To improve treatment efficacy

To improve treatment adherence

To decrease stigma

Patient convenience

Reduce the risk of relapse

Medical staff convenience

Next | Skip | Cancel

If there was more than one reason to initiate/switch treatment of this patient to PP3M, please select the SECOND MOST important reason here. Scroll to view all

To live a life that is as normal as possible

To reduce side effects

To improve treatment efficacy

To improve treatment adherence

To decrease stigma

Patient convenience

Reduce the risk of relapse

Medical staff convenience

Next

Skip

Cancel

If there was more than one reason to initiate/switch treatment of this patient to PP3M, please select the THIRD MOST important reason here. Scroll to view all

- To live a life that is as normal as possible
- To reduce side effects
- To improve treatment efficacy
- To improve treatment adherence
- To decrease stigma
- Patient convenience
- Reduce the risk of relapse
- Medical staff convenience

Next | Skip | Cancel

As a result of switching to PP3M was there any change in your patient's activity level (e.g. sports / hobbies, education, work, seeing friends and/or family)?

Activity level increased

No changes

Activity level decreased

Next

Skip

Cancel

If “ACTIVITY LEVEL INCREASED”:

Please indicate in what way patient’s activity was increased as a result of switching to PP3M? (multiple answers)

He/she started new sporting activities/hobbies

He/she returned to previous sporting activities/hobbies

Started working again

Changed job

Spends more time in social situations

Next   Skip   Cancel

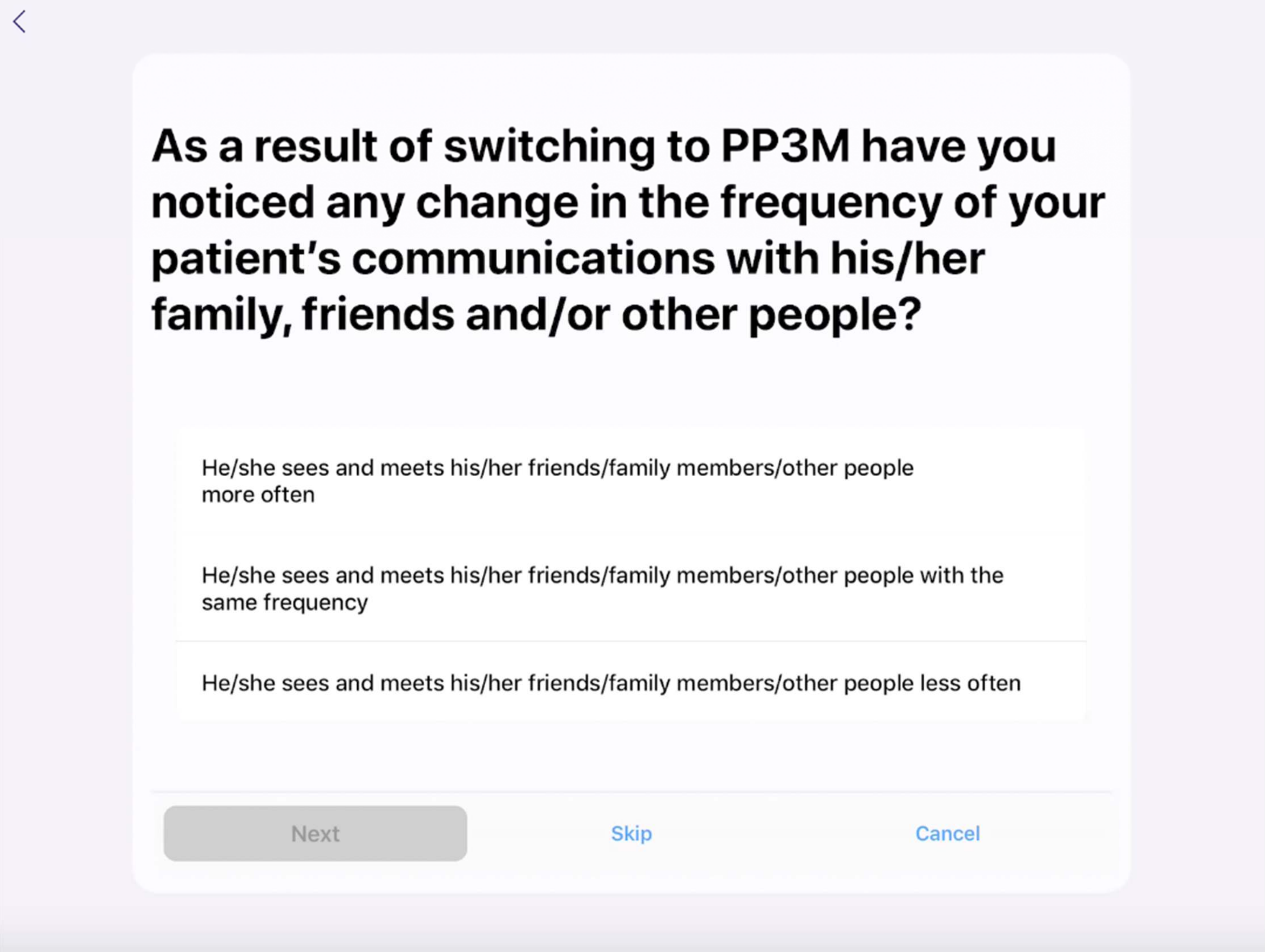
As a result of switching to PP3M have you noticed any change in the frequency of your patient's communications with his/her family, friends and/or other people?
He/she sees and meets his/her friends/family members/other people more often
He/she sees and meets his/her friends/family members/other people with the same frequency
He/she sees and meets his/her friends/family members/other people less often
Next
Skip
Cancel

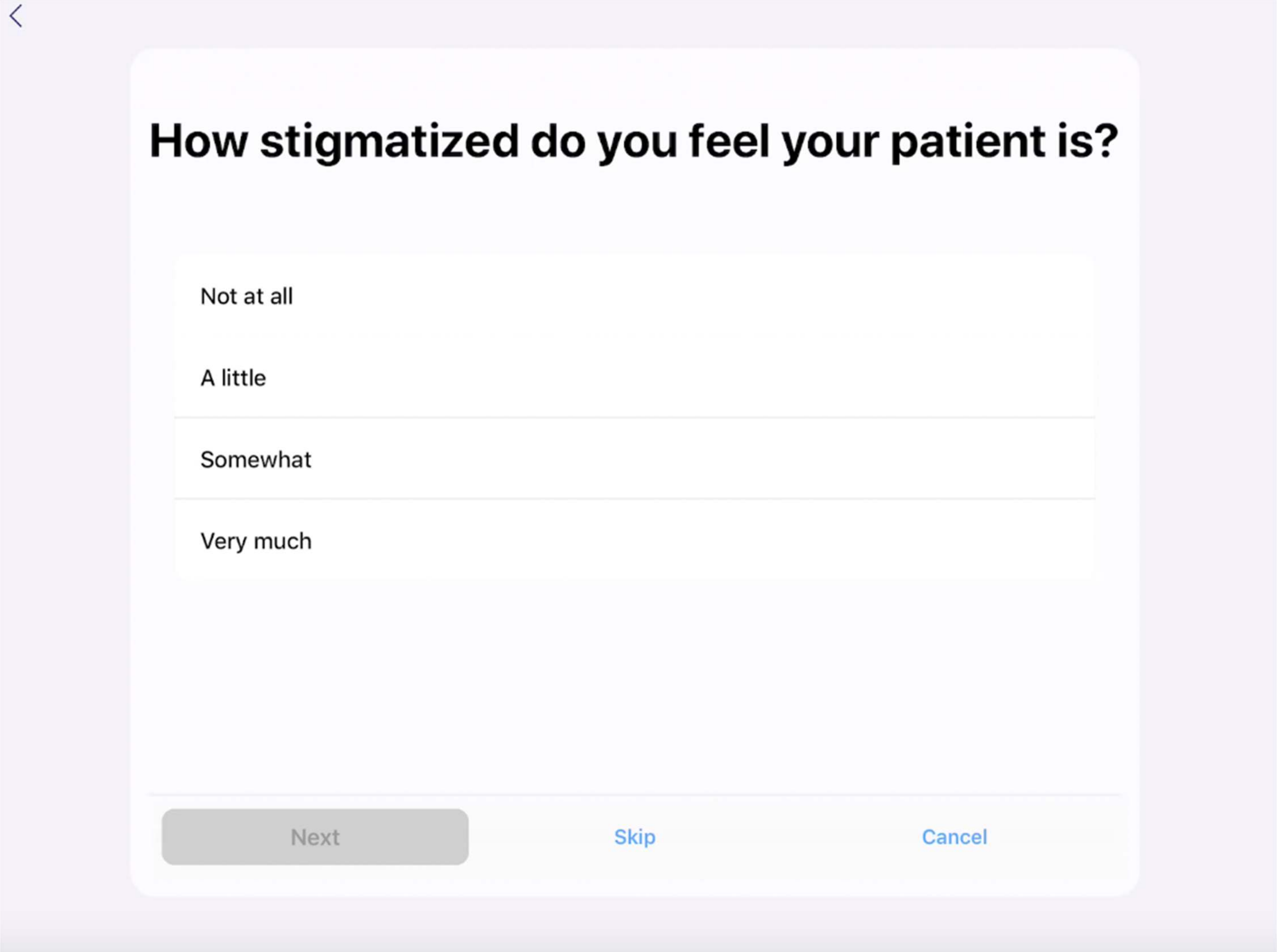
How stigmatized do you feel your patient is?
Not at all
A little
Somewhat
Very much
Next
Skip
Cancel

To what extent do you think that PP3M impacts on your patients feeling of being stigmatized?

No impact

Increases stigma

Decreases stigma

Next

Skip

Cancel

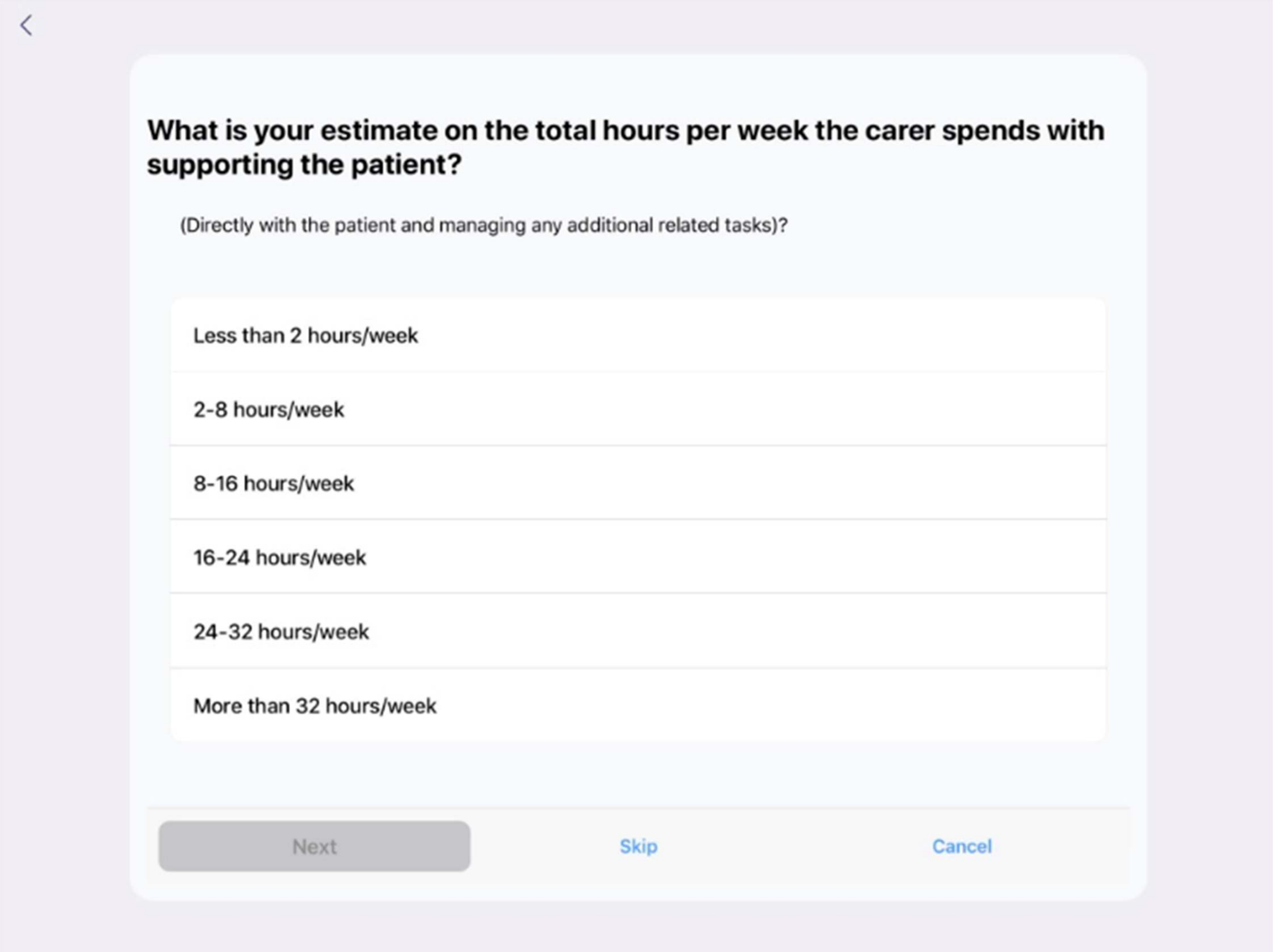
What is your estimate on the total hours per week the carer spends with supporting the patient?
(Directly with the patient and managing any additional related tasks)?
Less than 2 hours/week
2-8 hours/week
8-16 hours/week
16-24 hours/week
24-32 hours/week
More than 32 hours/week
Next
Skip
Cancel

Do you feel that the time required for support from the carer is less or more than before switching to PP3M?

Less

More

The same

Next | Skip | Cancel

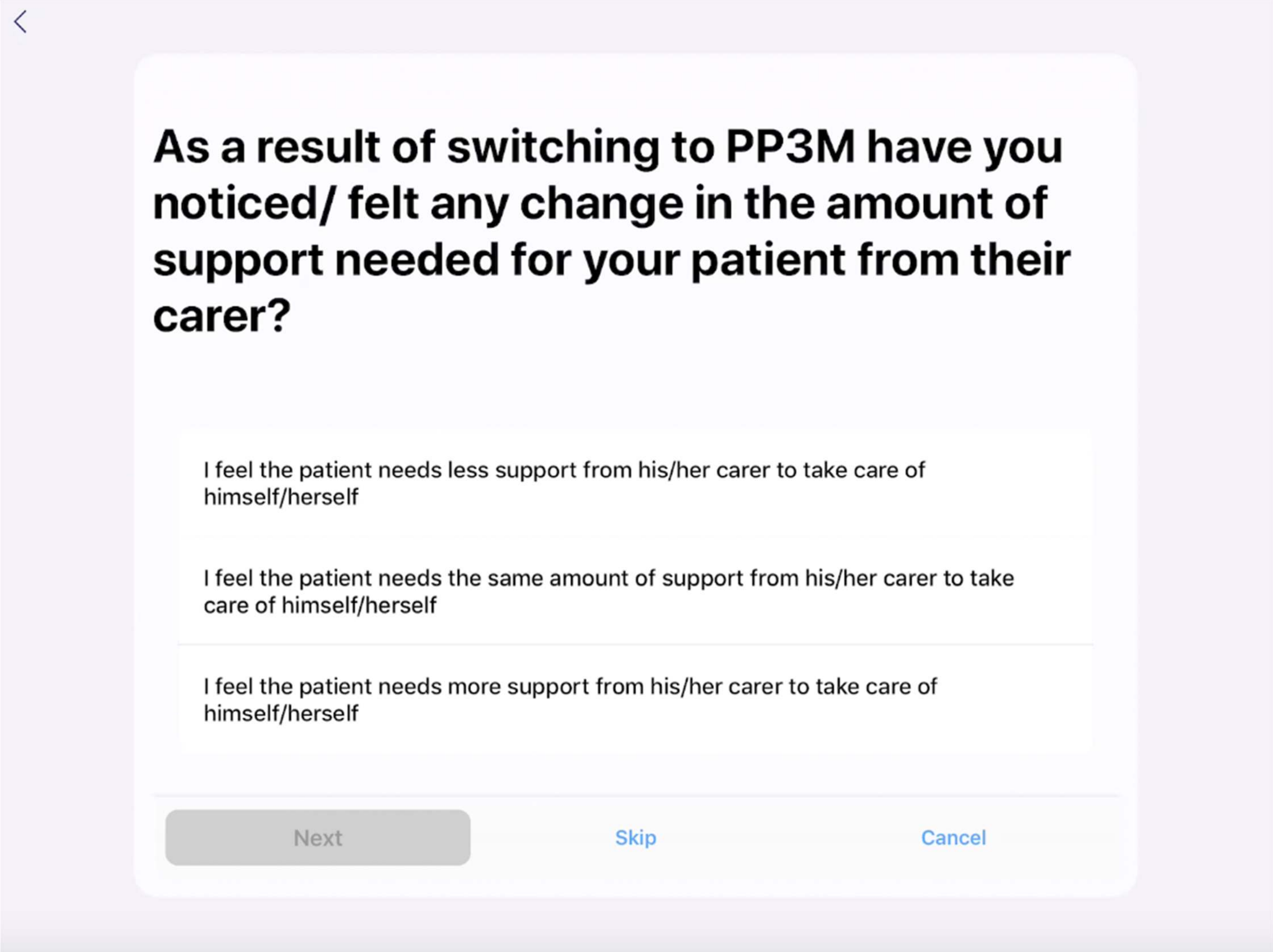
As a result of switching to PP3M have you noticed/ felt any change in the amount of support needed for your patient from their carer?
I feel the patient needs less support from his/her carer to take care of himself/herself
I feel the patient needs the same amount of support from his/her carer to take care of himself/herself
I feel the patient needs more support from his/her carer to take care of himself/herself
Next
Skip
Cancel

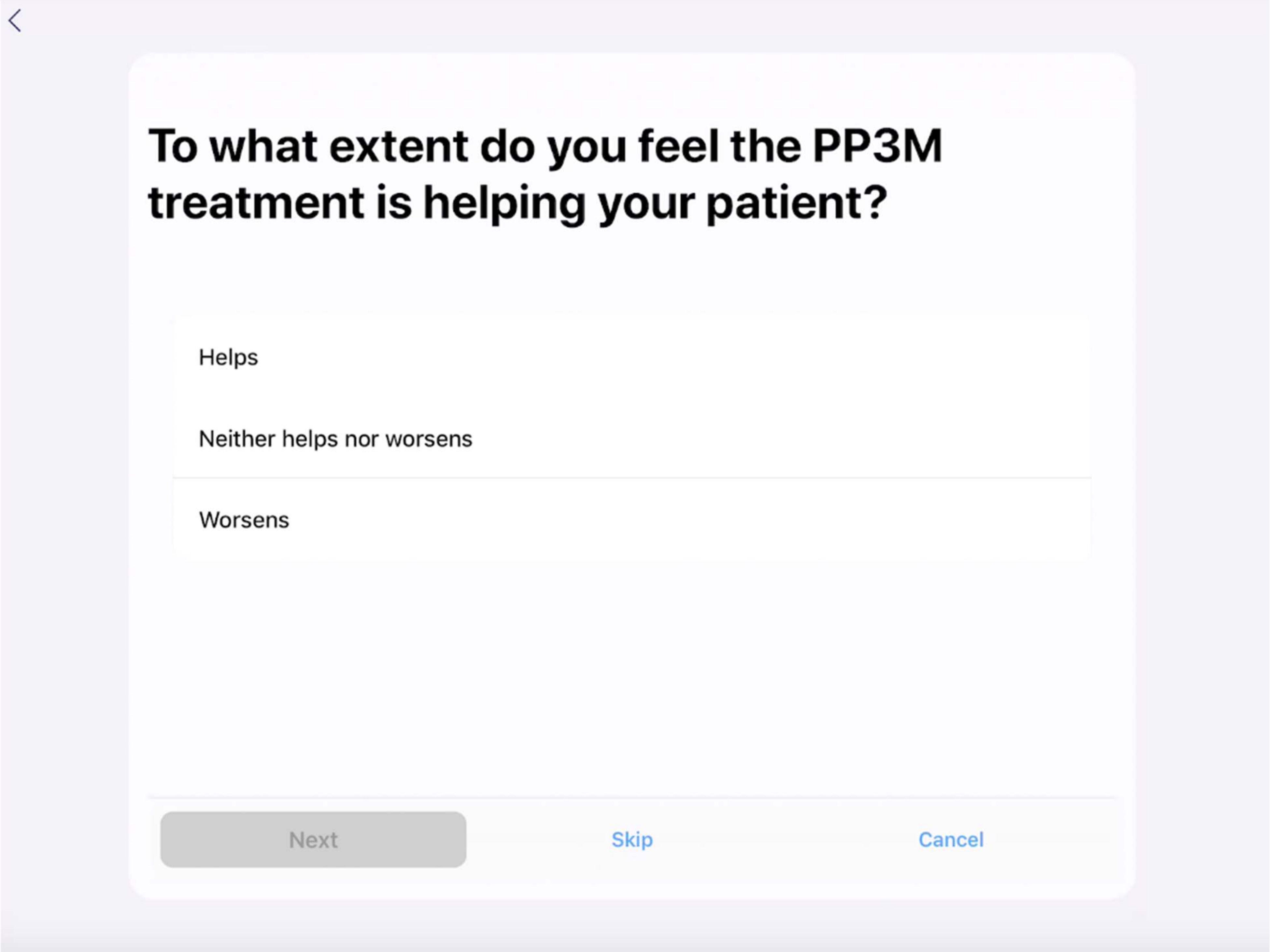

<

To what extent do you feel the PP3M treatment is helping your patient?

Helps

Neither helps nor worsens

Worsens

Next

Skip

Cancel

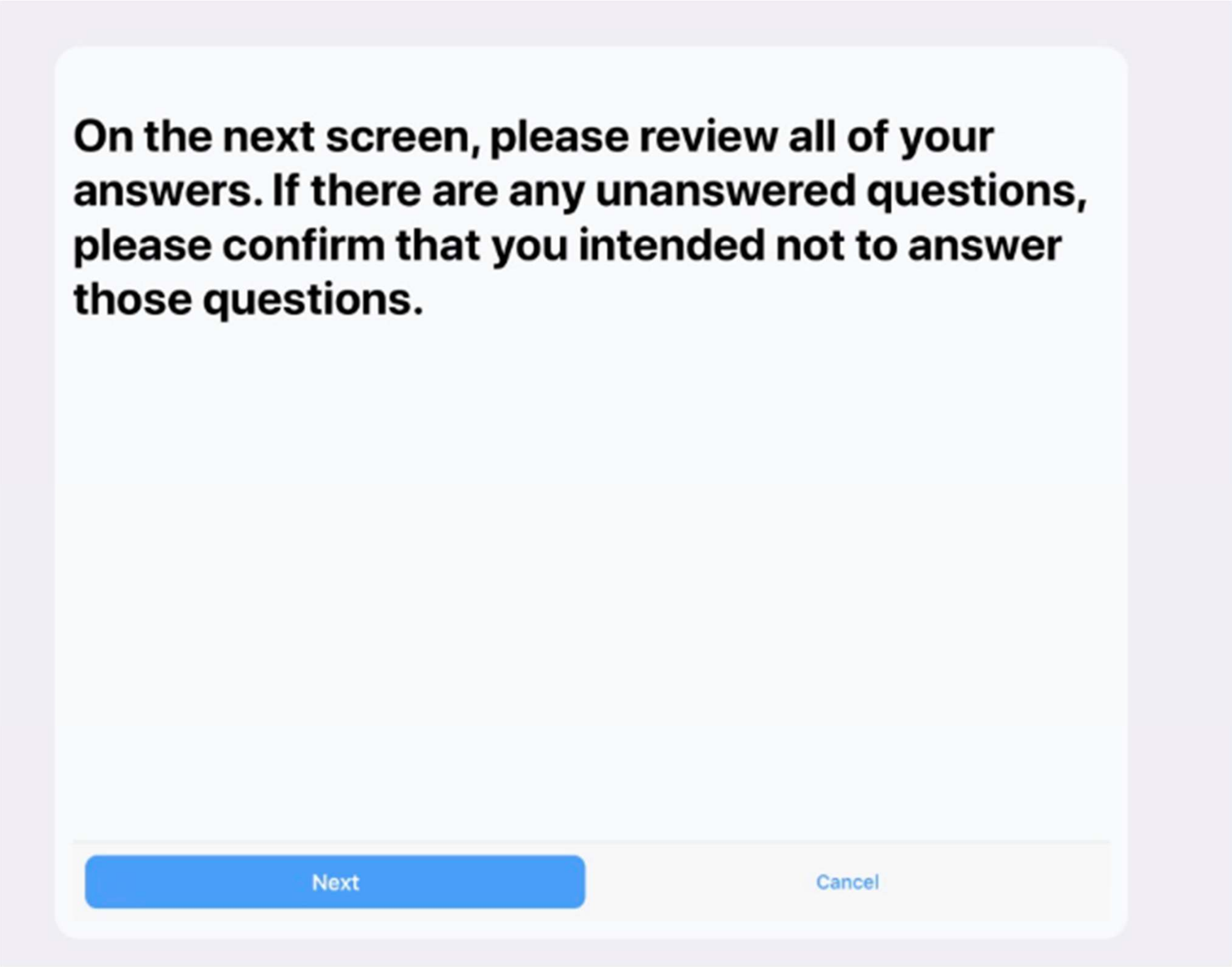
On the next screen, please review all of your answers. If there are any unanswered questions, please confirm that you intended not to answer those questions.
Next
Cancel

Please review your answers. Pressing “Next” will submit your answers

You can jump directly to update question by clicking on it in the list below

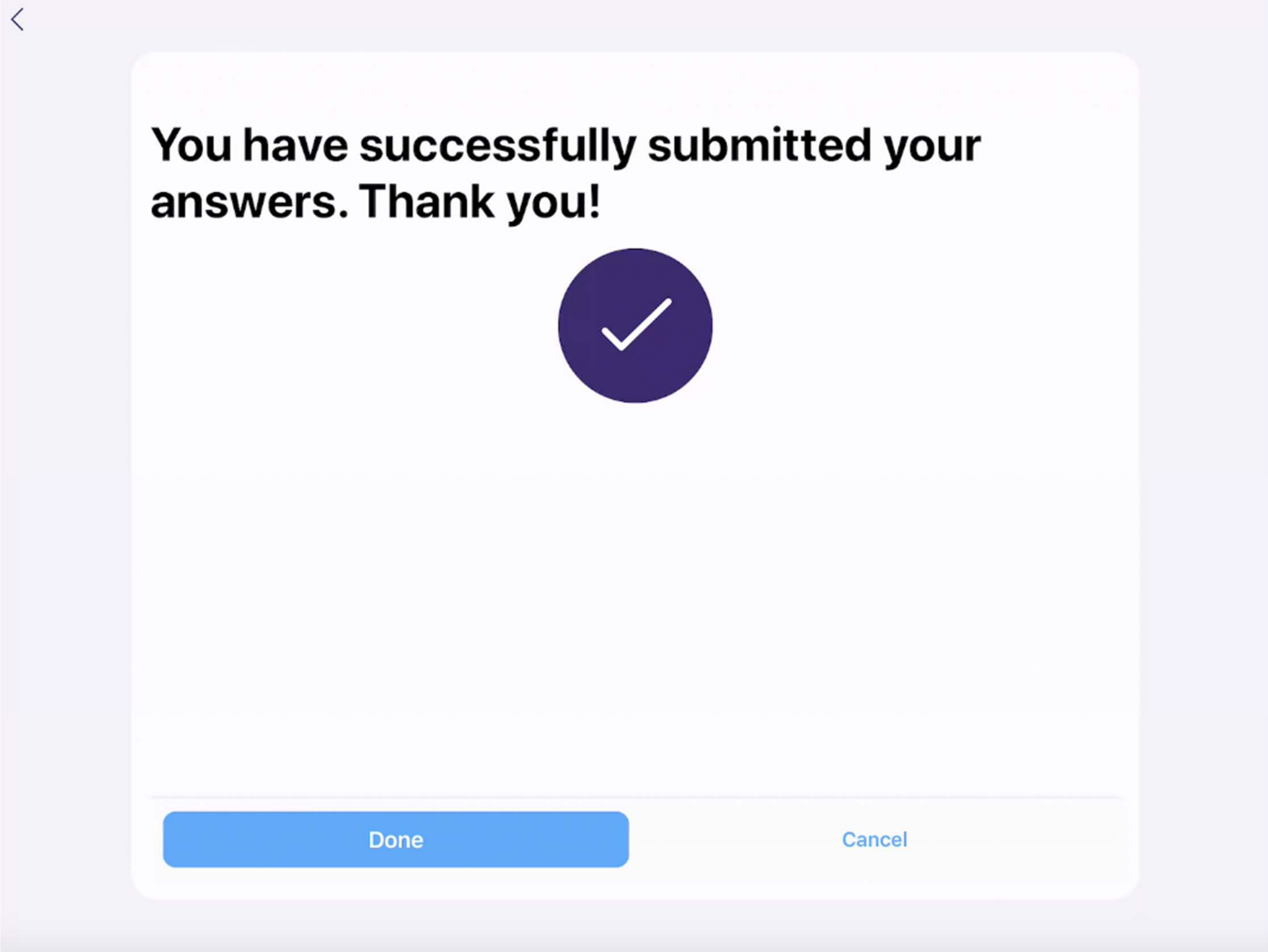
You have successfully submitted your answers. Thank you!
Done
Cancel
