# Supplementary material for: The Patient, Investigator, Nurse, Carer Questionnaire (PINC-Q): a cross-sectional, retrospective, non-interventional study exploring the impact of less frequent medication administration with paliperidone palmitate 3-monthly as maintenance treatment for schizophrenia
Source: BMC Psychiatry. 2021 Jun 9;21:300. doi: 10.1186/s12888-021-03305-z (PMC8191017; doi:10.1186/s12888-021-03305-z)
Supplement: Supplementary file 7 — Additional file 7. English patient questionnaire. [file 12888_2021_3305_MOESM7_ESM.pdf]

English Patient Survey

Welcome to the English Patient Survey. This survey has approximately 35 questions, we anticipate it will take 15-20 minutes to complete.

Get Started

Cancel

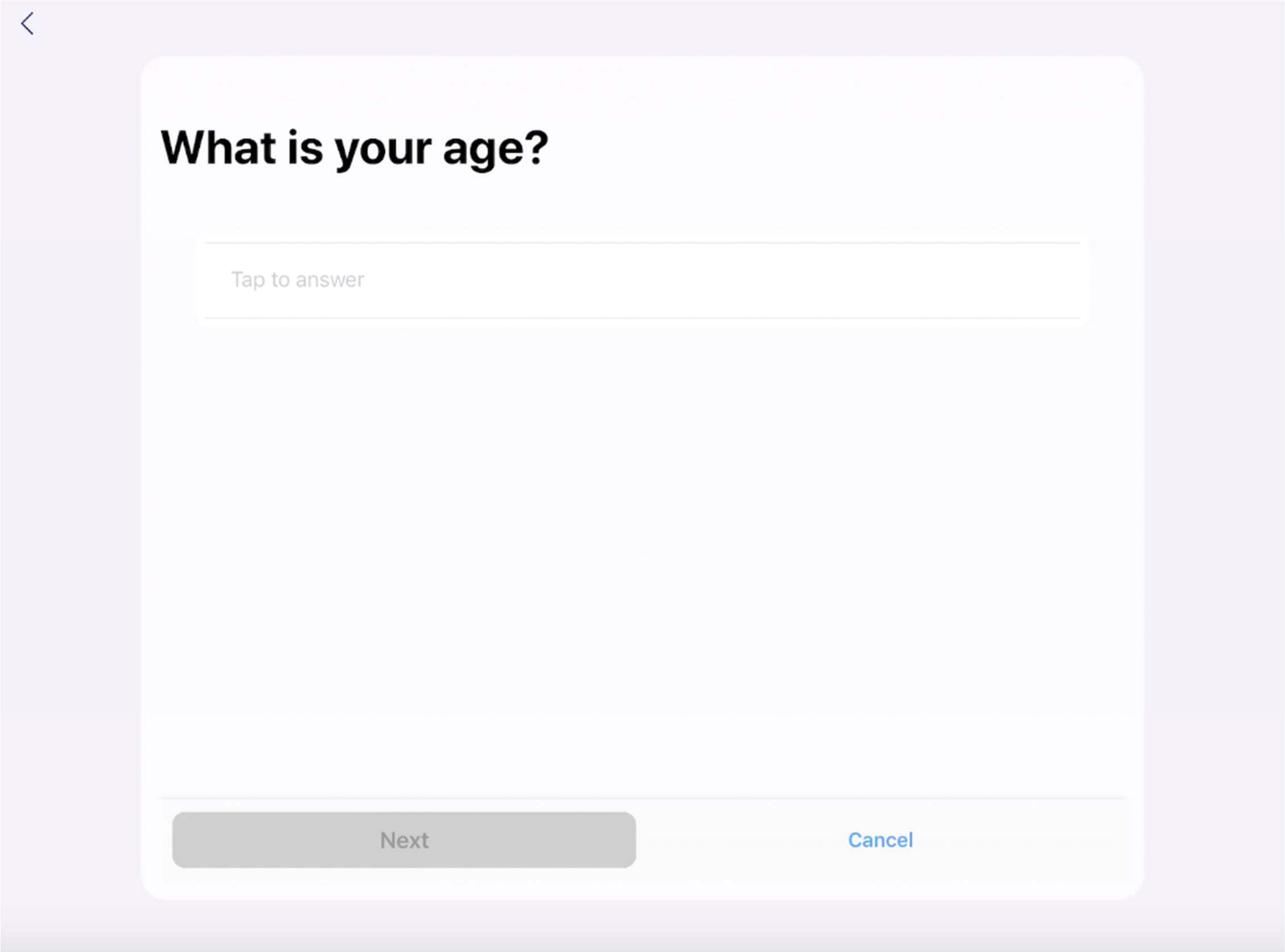
What is your age?
Tap to answer
Next
Cancel

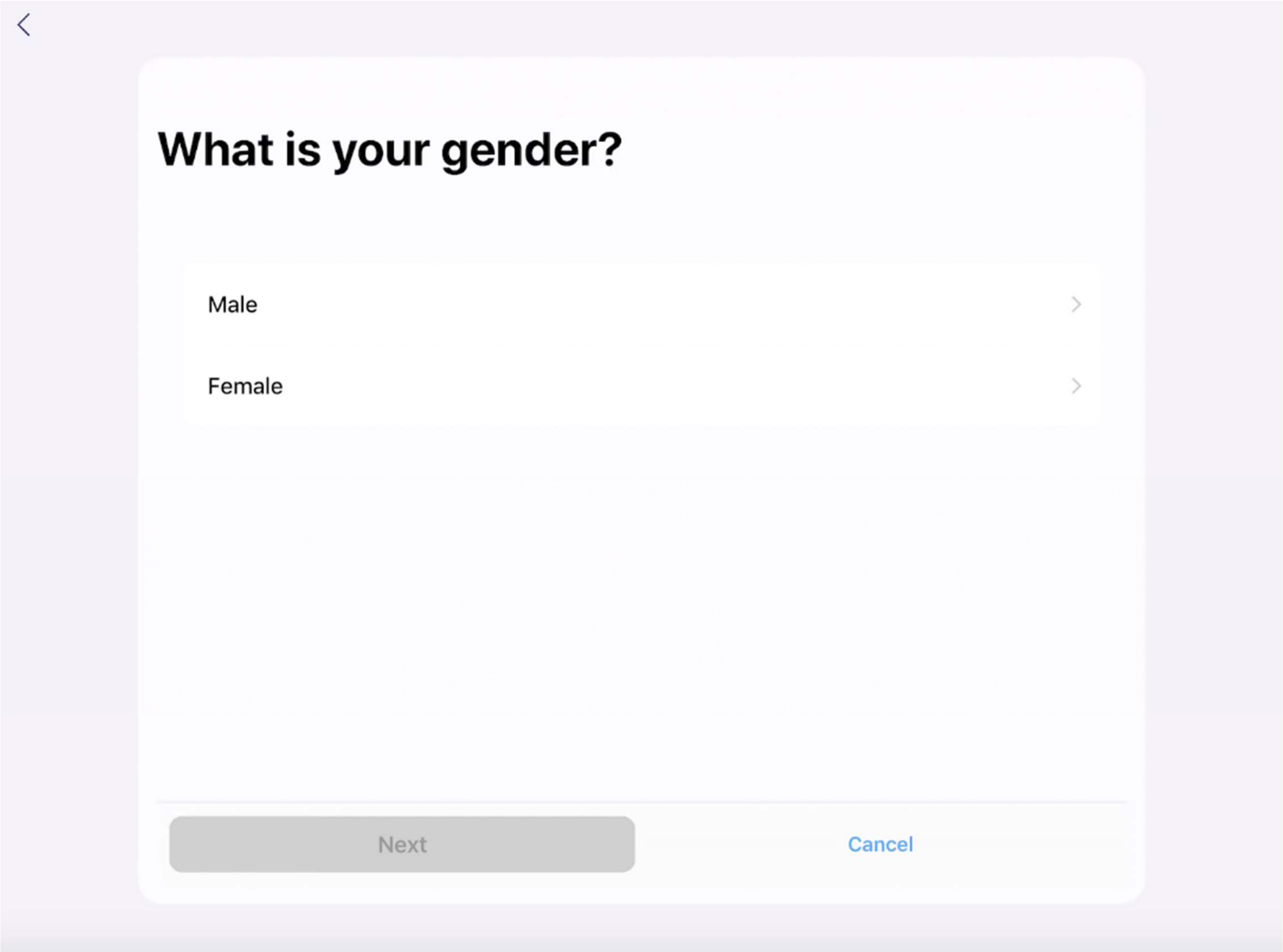
What is your gender?
Male
Female
Next
Cancel

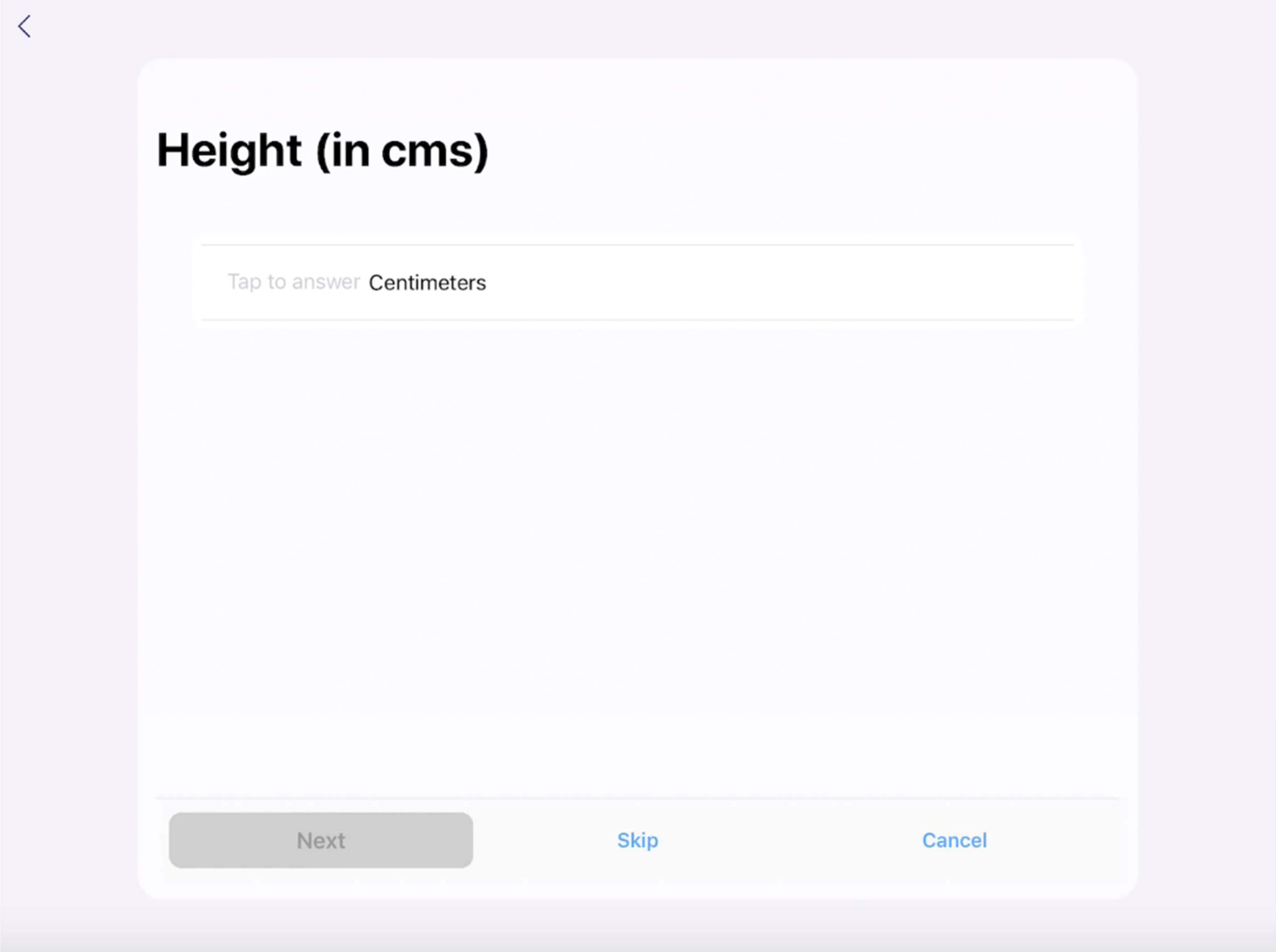
Height (in cms)
Tap to answer Centimeters
Next
Skip
Cancel

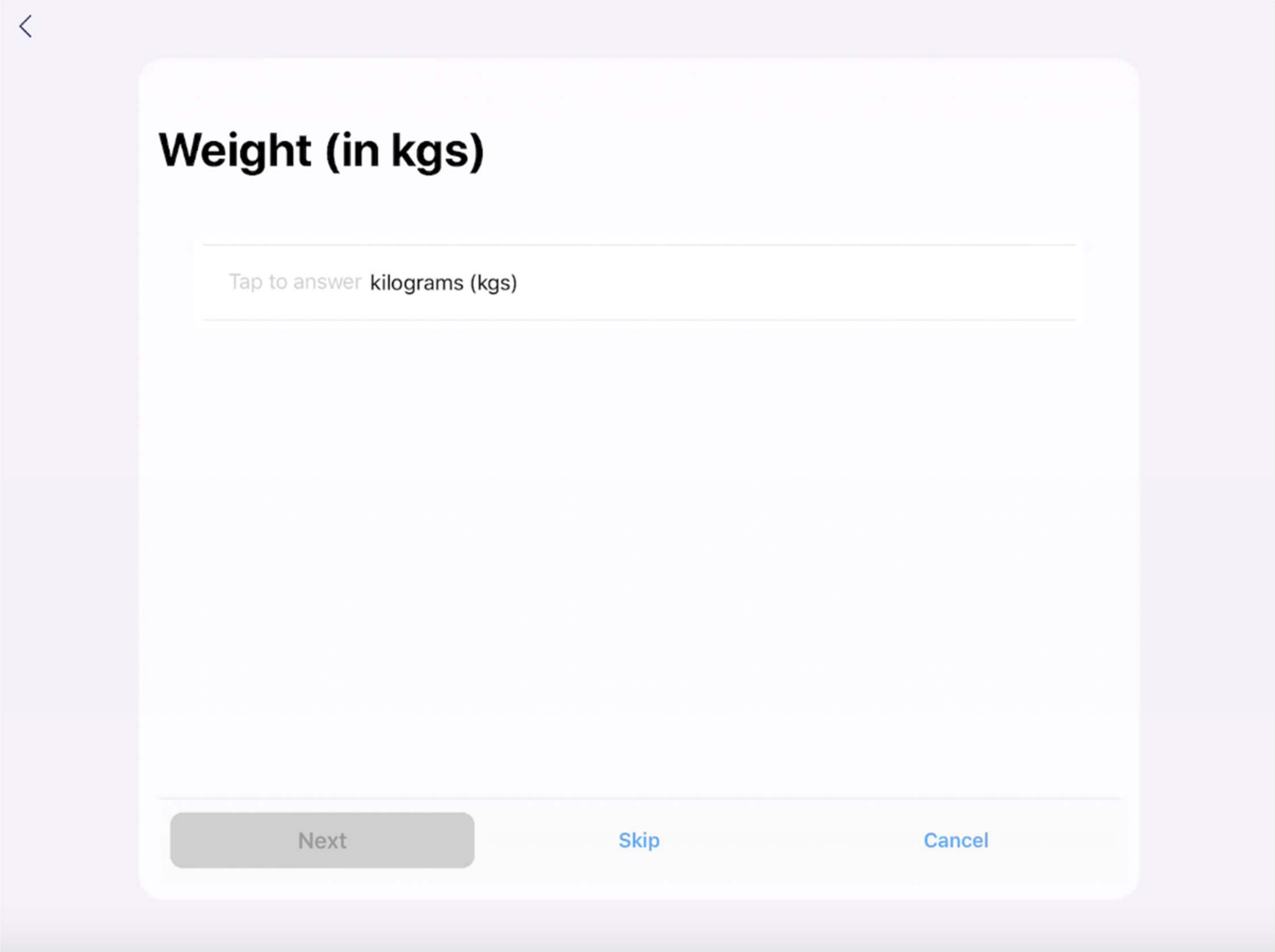
Weight (in kgs)
Tap to answer kilograms (kgs)
Next
Skip
Cancel

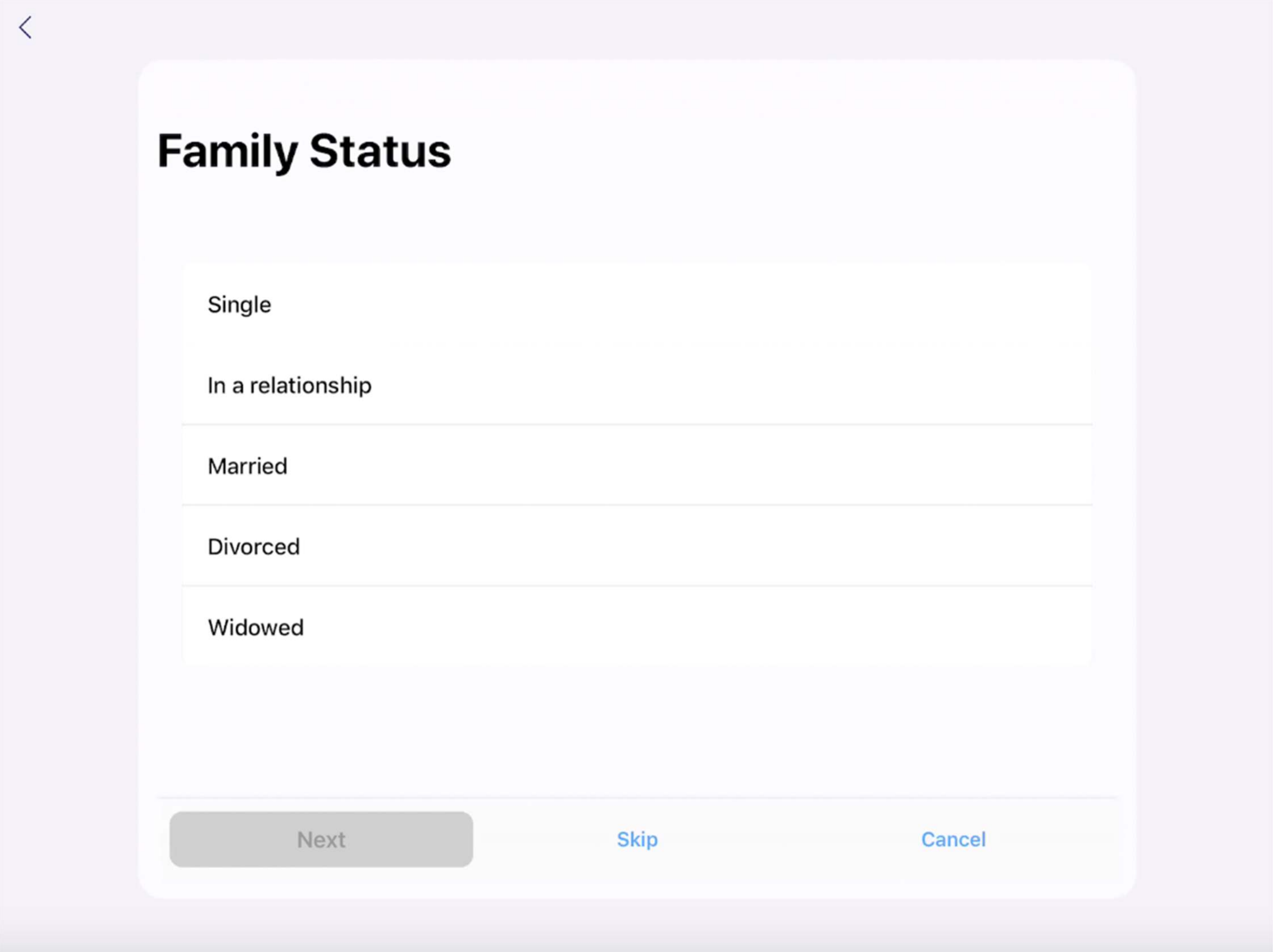
Family Status
Single
In a relationship
Married
Divorced
Widowed
Next
Skip
Cancel

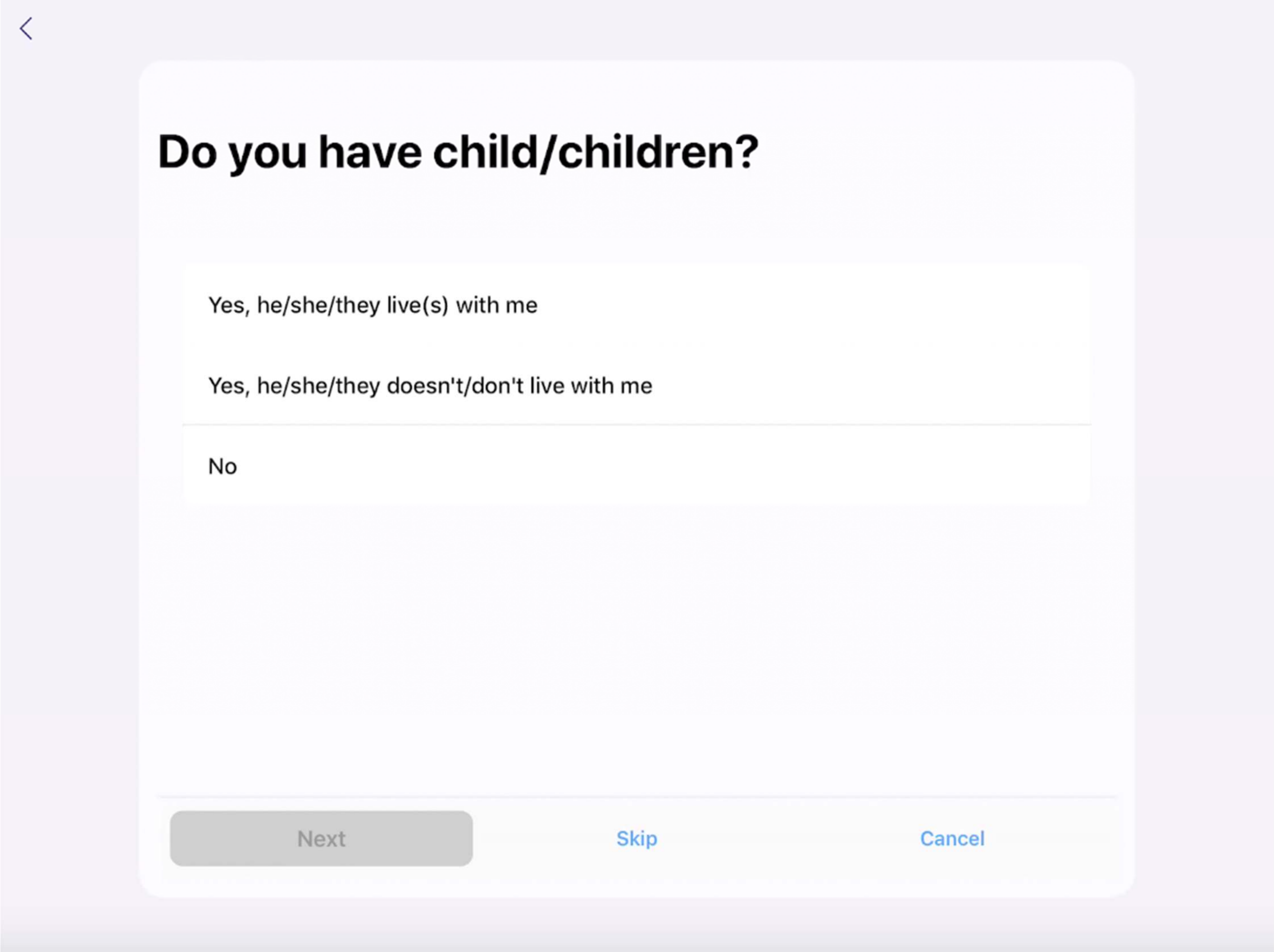
Do you have child/children?
Yes, he/she/they live(s) with me
Yes, he/she/they doesn't/don't live with me
No
Next
Skip
Cancel

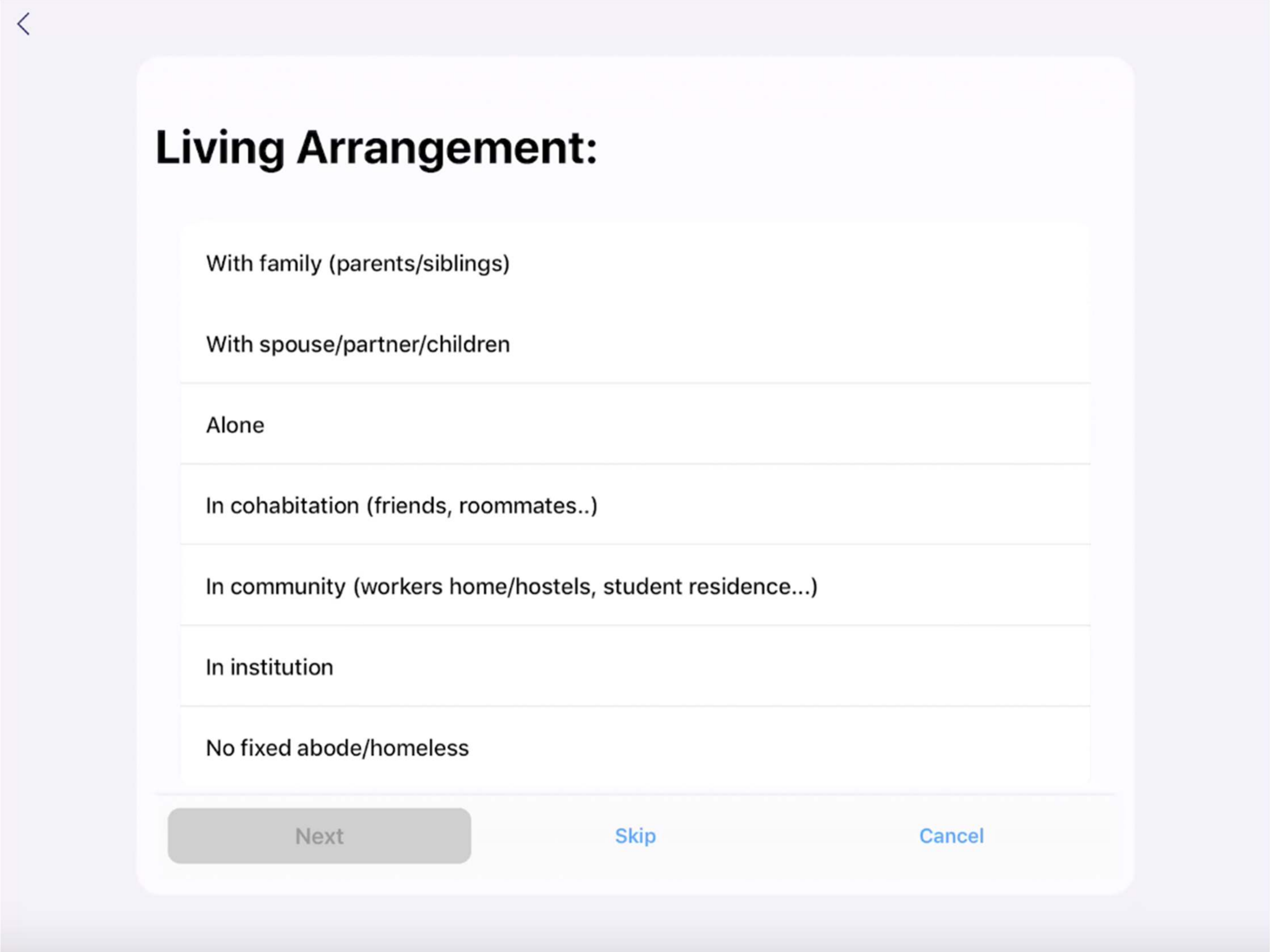
Living Arrangement:
With family (parents/siblings)
With spouse/partner/children
Alone
In cohabitation (friends, roommates..)
In community (workers home/hostels, student residence...)
In institution
No fixed abode/homeless
Next
Skip
Cancel

Education Level

Did not finish high school

Finished high school

Apprenticeship/Professional qualification

Higher education <3 years

University degree

University higher degree (Masters, PhD)

Next Skip Cancel

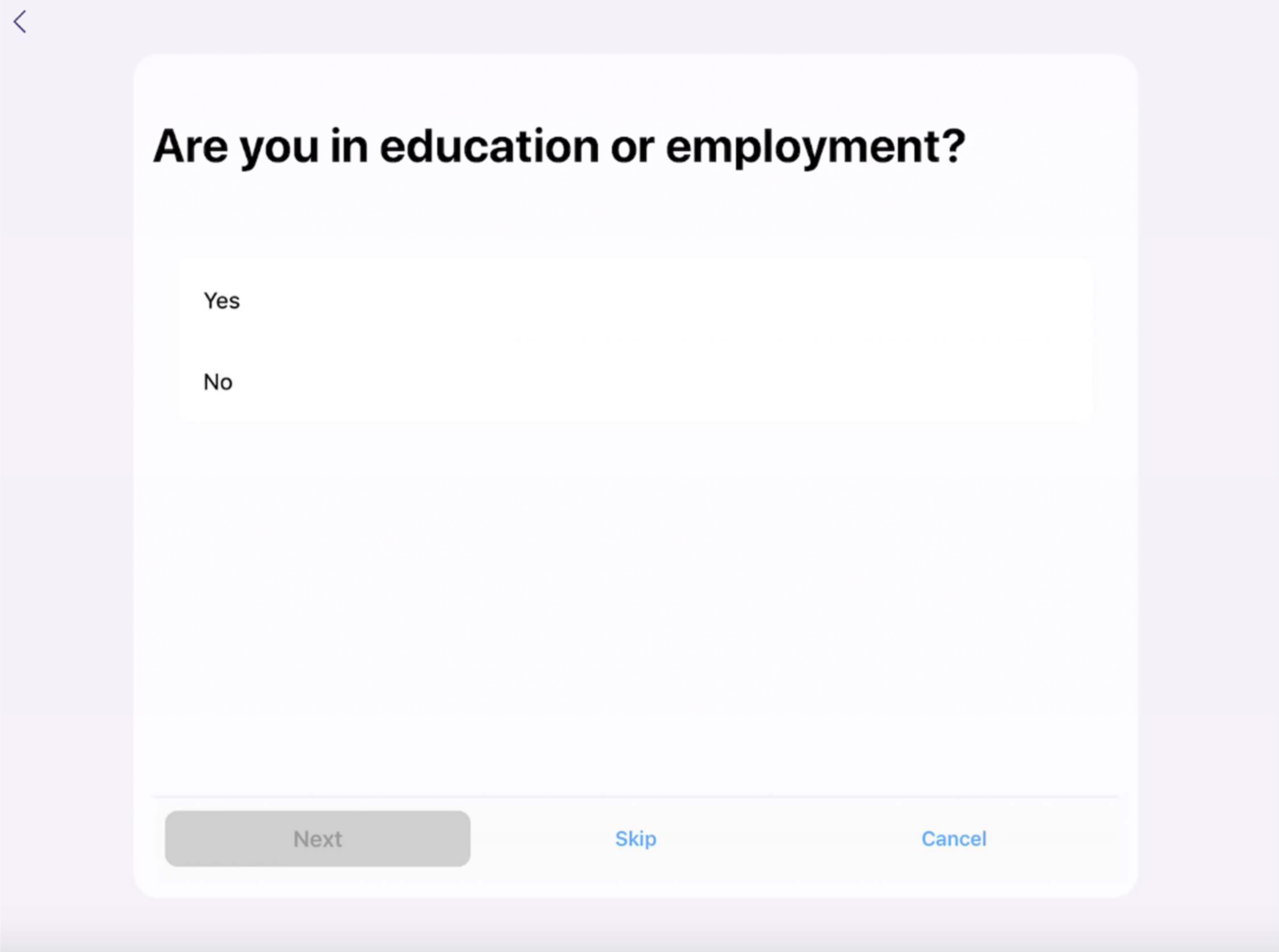
Are you in education or employment?
Yes
No
Next
Skip
Cancel

If YES, please specify:

Employee, paid

Employee, voluntary

Employee in specialized/protected employment settings

Self-employed worker

Student, professional course/training

Next

Skip

Cancel

If NO, please specify:

Unemployed

Receiving a disability allowance

Seeking employment

Next

Skip

Cancel

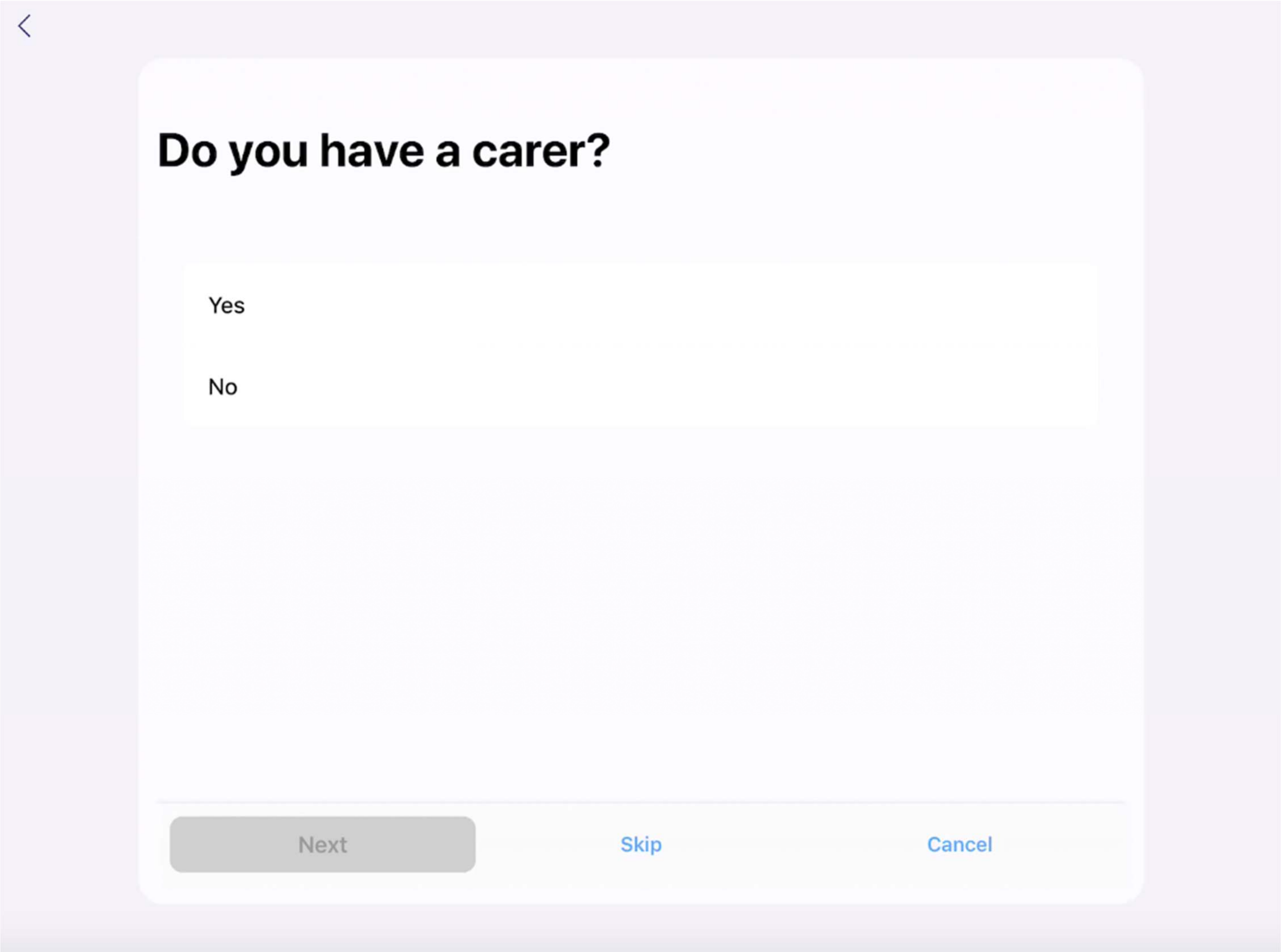
Do you have a carer?
Yes
No
Next
Skip
Cancel

Whom do you find important to involve in the treatment decision? (multiple answers)

Patient

Carer

Doctor

Nurse

Other

Next

Skip

Cancel

EQ-5D-5L

Health Questionnaire
English version for the UK

Previous

Next

© EuroQol Research Foundation. EQ-5D™ is a trade mark of the EuroQol Research Foundation

Cancel

Your mobility TODAY

I have no problems in walking about ☐

I have slight problems in walking about ☐

I have moderate problems in walking about ☐

I have severe problems in walking about ☐

I am unable to walk about ☐

Previous | Next

© EuroQol Research Foundation. EQ-5D™ is a trade mark of the EuroQol Research Foundation

Cancel

Your self-care TODAY

I have no problems washing or dressing myself ☐

I have slight problems washing or dressing myself ☐

I have moderate problems washing or dressing myself ☐

I have severe problems washing or dressing myself ☐

I am unable to wash or dress myself ☐

Previous Next

© EuroQol Research Foundation. EQ-5D™ is a trade mark of the EuroQol Research Foundation

Cancel

Your usual activities TODAY

(e.g. work, study, housework, family or leisure activities)

I have no problems doing my usual activities ☐

I have slight problems doing my usual activities ☐

I have moderate problems doing my usual activities ☐

I have severe problems doing my usual activities ☐

I am unable to do my usual activities ☐

Previous

Next

© EuroQol Research Foundation. EQ-5D™ is a trade mark of the EuroQol Research Foundation

Cancel

Your pain / discomfort TODAY

I have no pain or discomfort ☐

I have slight pain or discomfort ☐

I have moderate pain or discomfort ☐

I have severe pain or discomfort ☐

I have extreme pain or discomfort ☐

Previous | Next

© EuroQol Research Foundation. EQ-5D™ is a trade mark of the EuroQol Research Foundation

Cancel

Your anxiety / depression TODAY

I am not anxious or depressed ☐

I am slightly anxious or depressed ☐

I am moderately anxious or depressed ☐

I am severely anxious or depressed ☐

I am extremely anxious or depressed ☐

Previous Next

© EuroQol Research Foundation. EQ-5D™ is a trade mark of the EuroQol Research Foundation

Cancel

- We would like to know how good or bad your health is TODAY.
- This scale is numbered from 0 to 100.
- 100 means the best health you can imagine.
  0 means the worst health you can imagine.
- Please tap on the scale to indicate how your health is TODAY.

YOUR HEALTH
TODAY

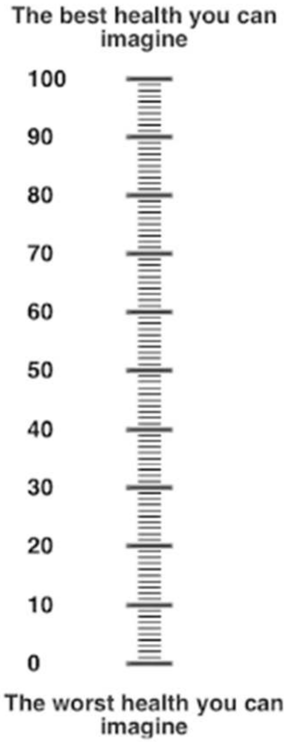

Previous

Next

© EuroQol Research Foundation. EQ-5D™ is a trade mark of the EuroQol Research Foundation

Cancel

How many times have you seen your doctor during the last 6 months?

Tap to answer times

Next

Skip

Cancel

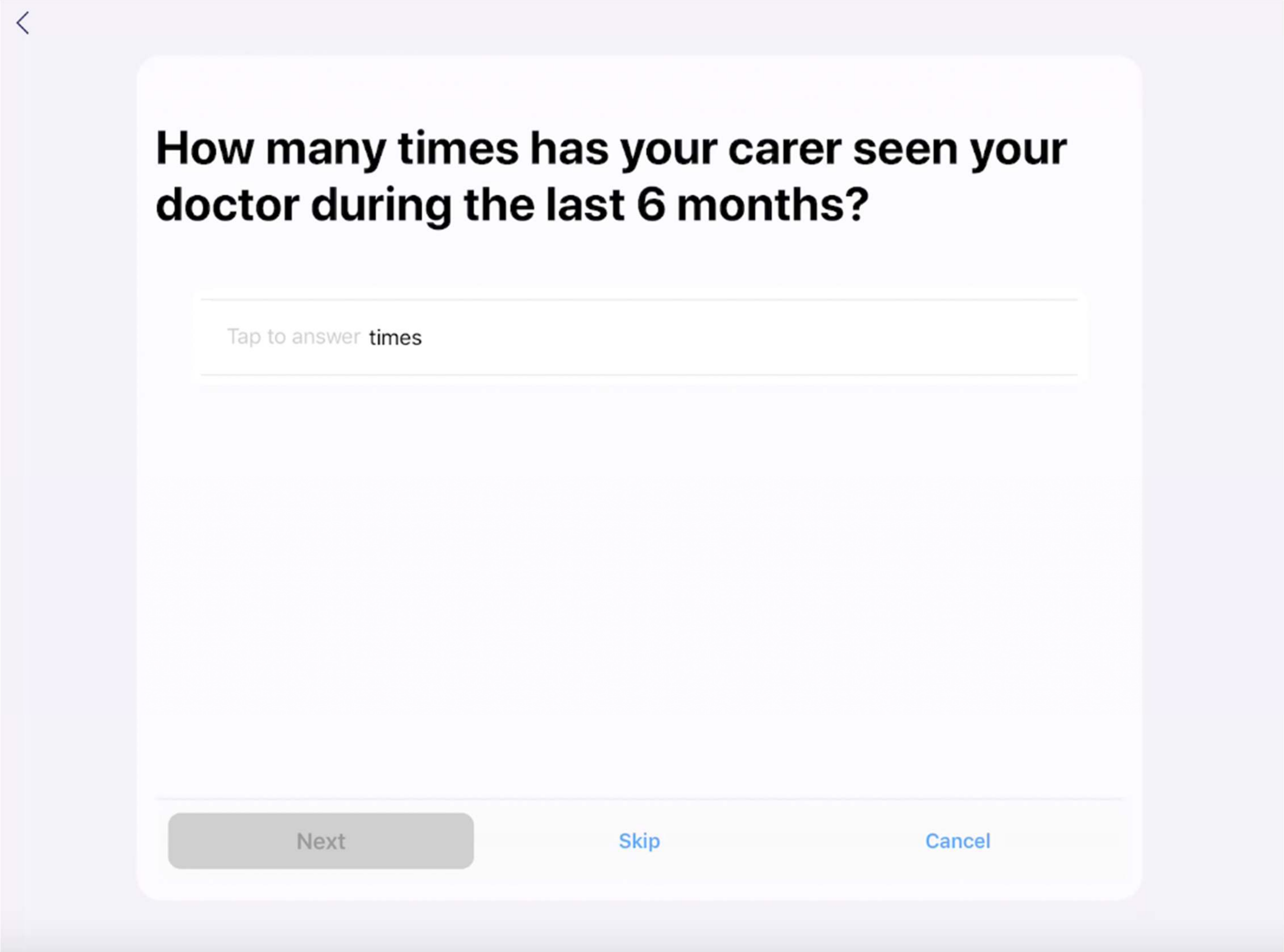
How many times has your carer seen your doctor during the last 6 months?
Tap to answer times
Next
Skip
Cancel

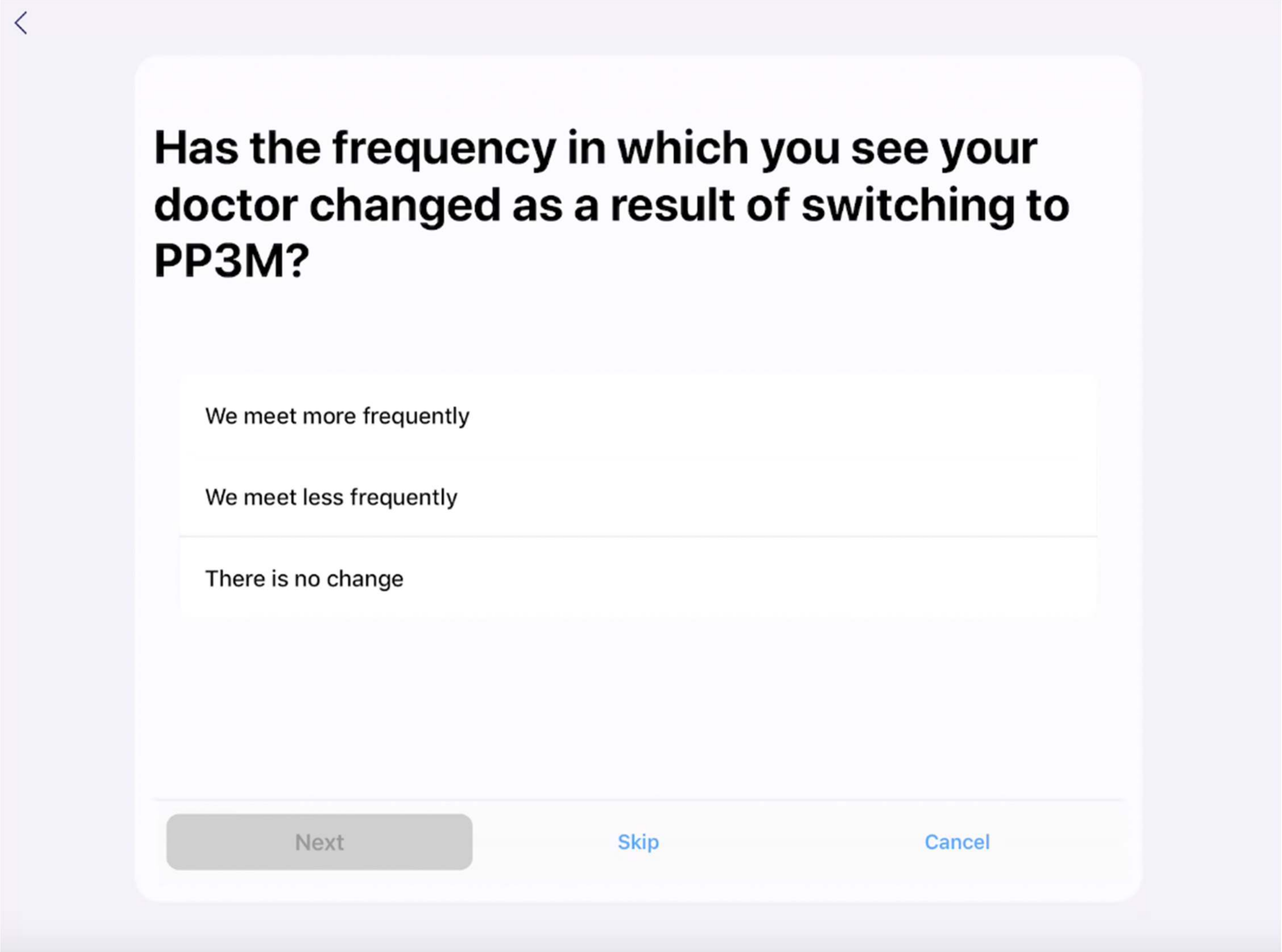
Has the frequency in which you see your doctor changed as a result of switching to PP3M?
We meet more frequently
We meet less frequently
There is no change
Next
Skip
Cancel

How satisfied are you with the current frequency in which you see your doctor?

Very Satisfied

Satisfied

Dissatisfied

Very Dissatisfied

Next Skip Cancel

Has the frequency in which your carer sees your doctor changed as a result of switching to PP3M?

They meet more frequently

They meet less frequently

There is no change

Next

Skip

Cancel

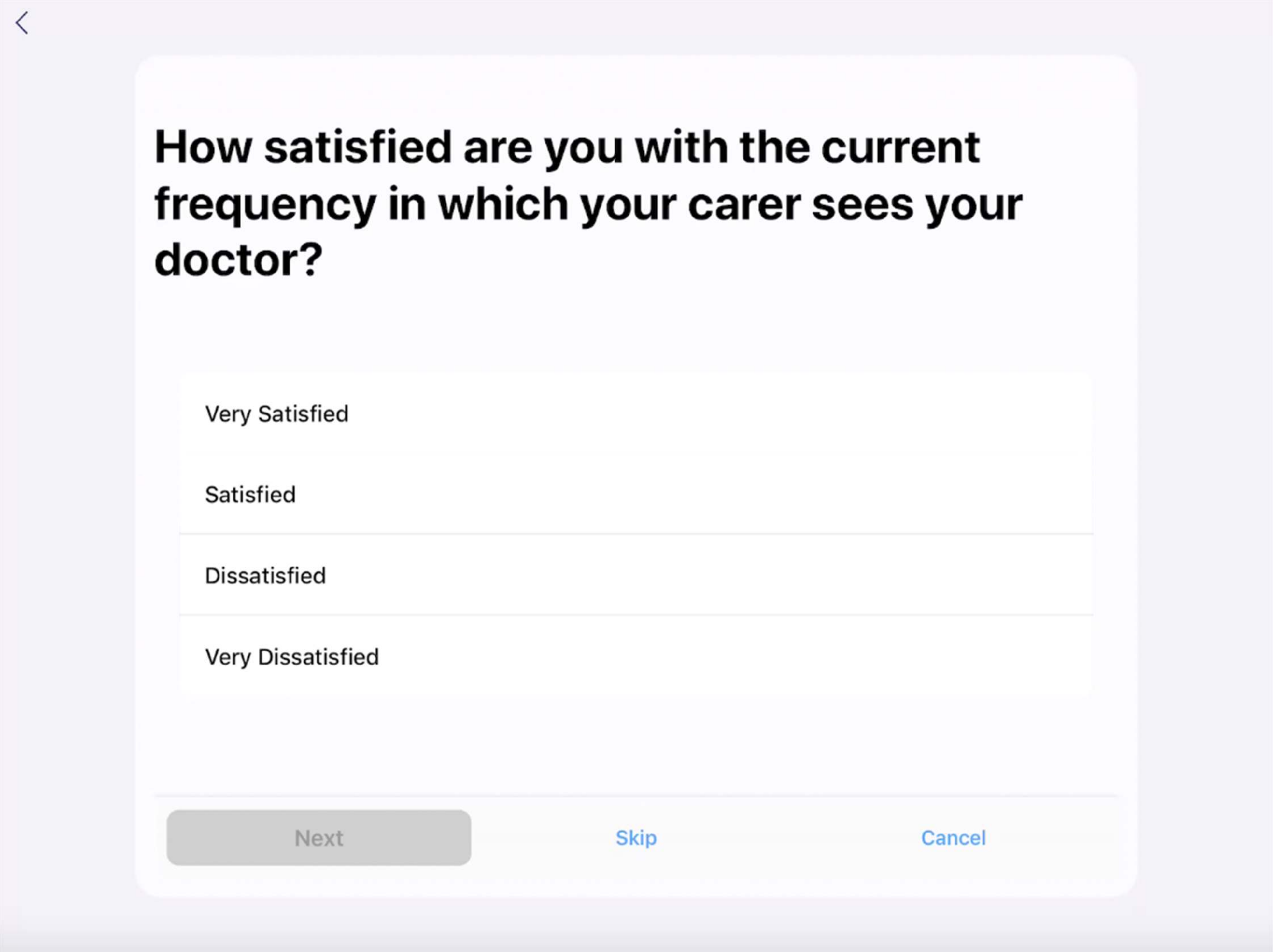
How satisfied are you with the current frequency in which your carer sees your doctor?
Very Satisfied
Satisfied
Dissatisfied
Very Dissatisfied
Next
Skip
Cancel

In your opinion, how often do you like/want to be seen by your doctor? (regardless of the injection frequency)

- More than once per month
- Monthly
- Once every three months
- Less than every three months

Next | Skip | Cancel

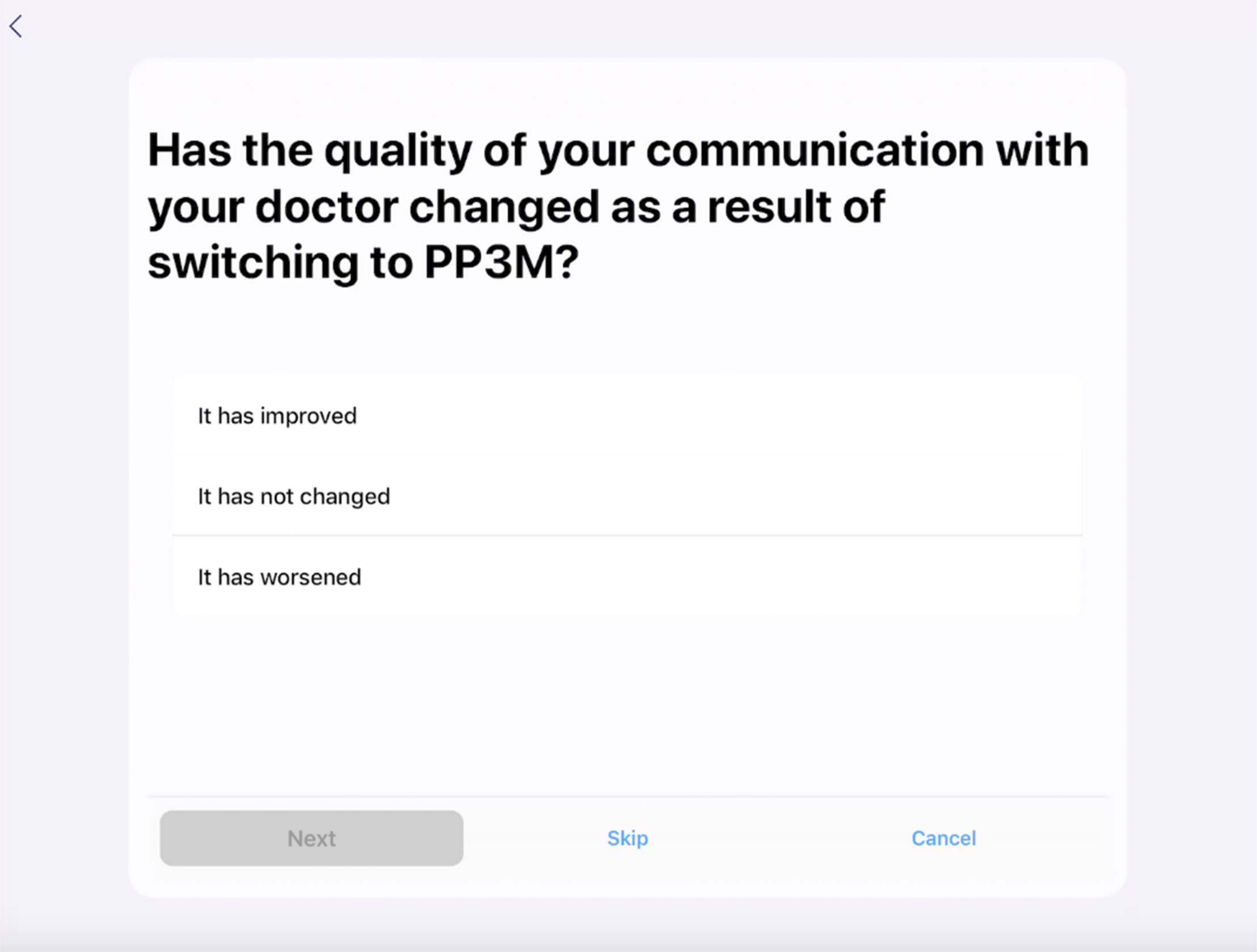
Has the quality of your communication with your doctor changed as a result of switching to PP3M?
It has improved
It has not changed
It has worsened
Next
Skip
Cancel

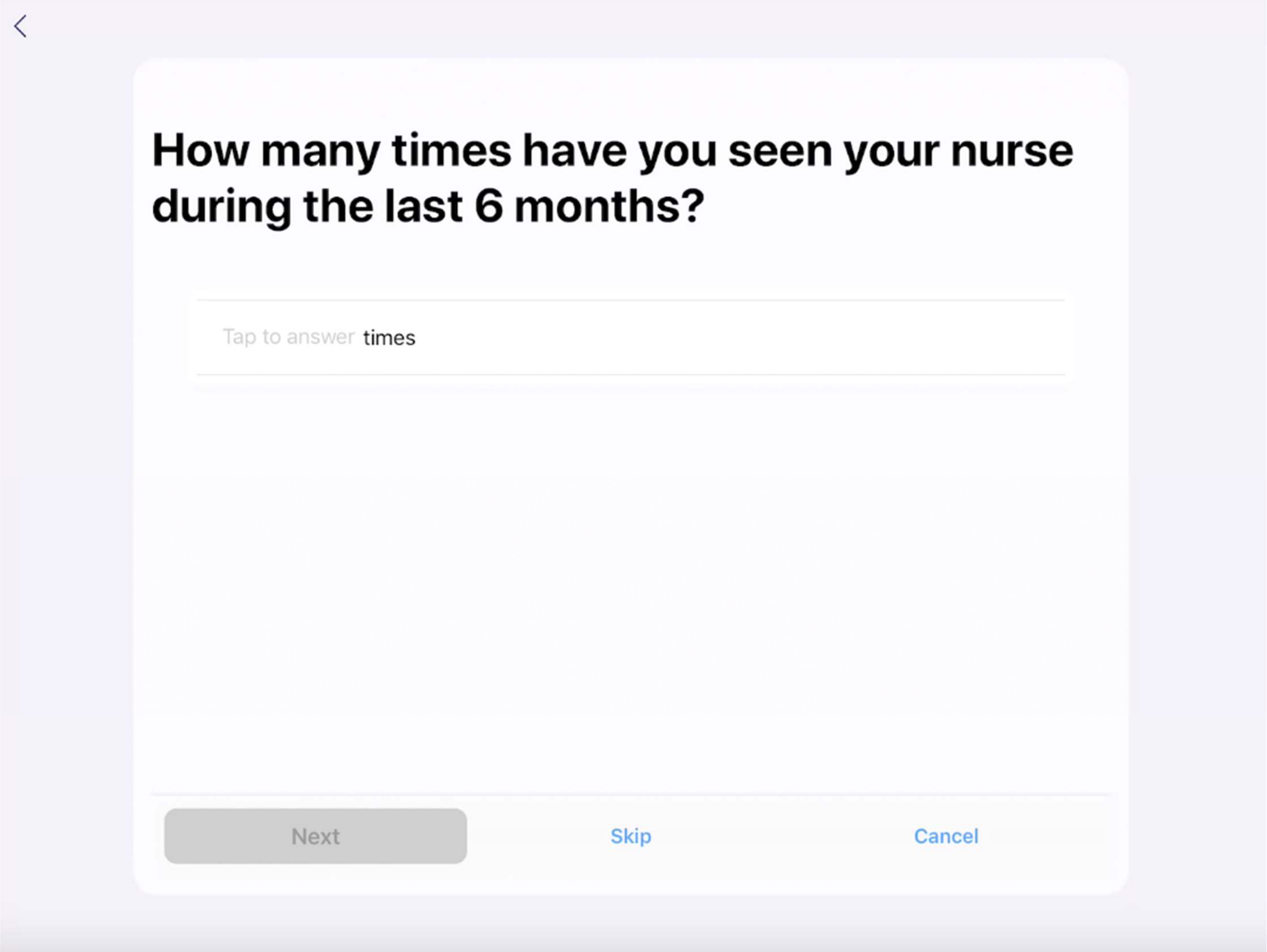
How many times have you seen your nurse during the last 6 months?
Tap to answer times
Next
Skip
Cancel

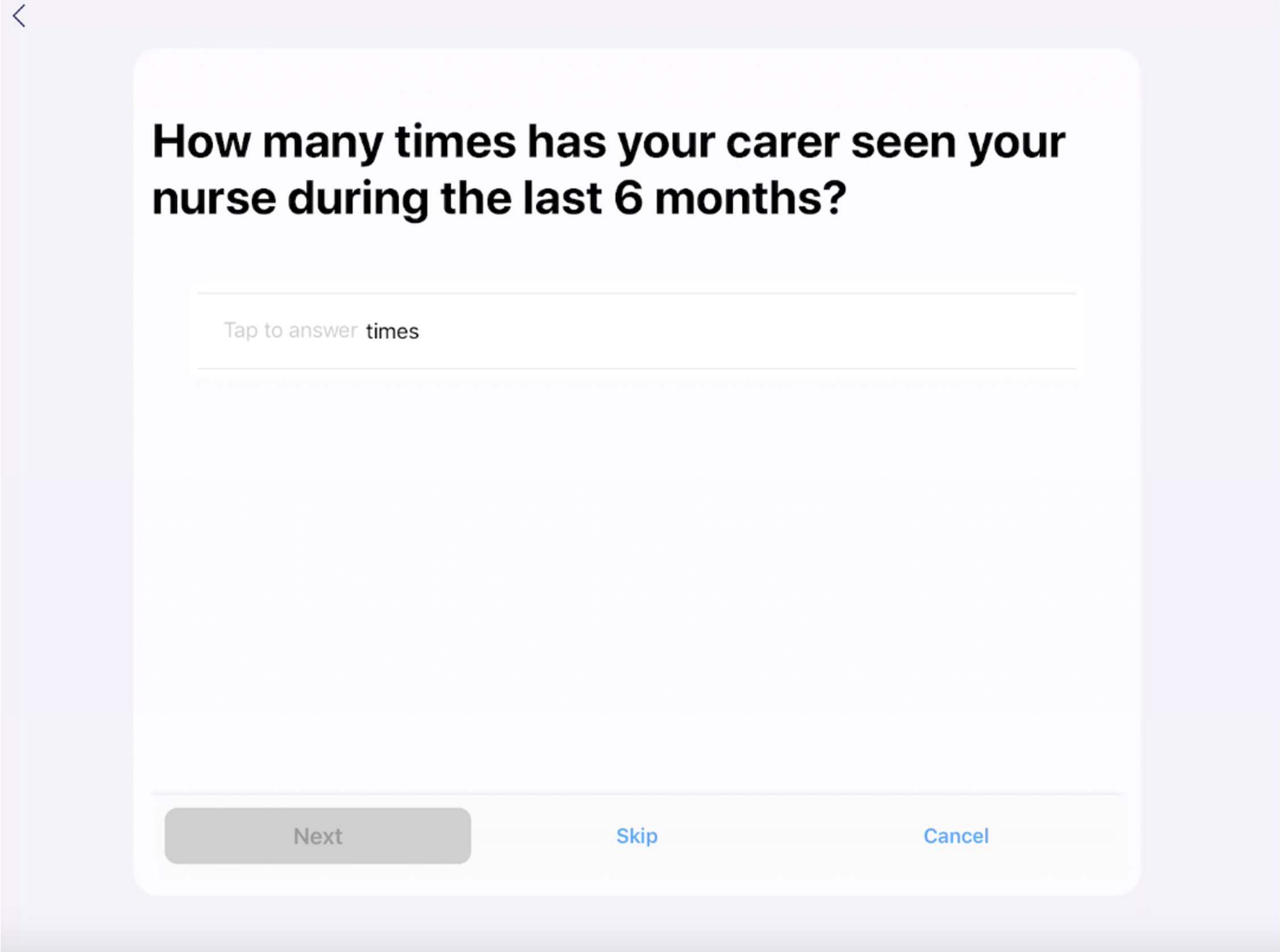
How many times has your carer seen your nurse during the last 6 months?
Tap to answer times
Next
Skip
Cancel

Has the frequency in which you see your nurse changed as a result of switching to PP3M?

We meet more frequently

We meet less frequently

There is no change

Next

Skip

Cancel

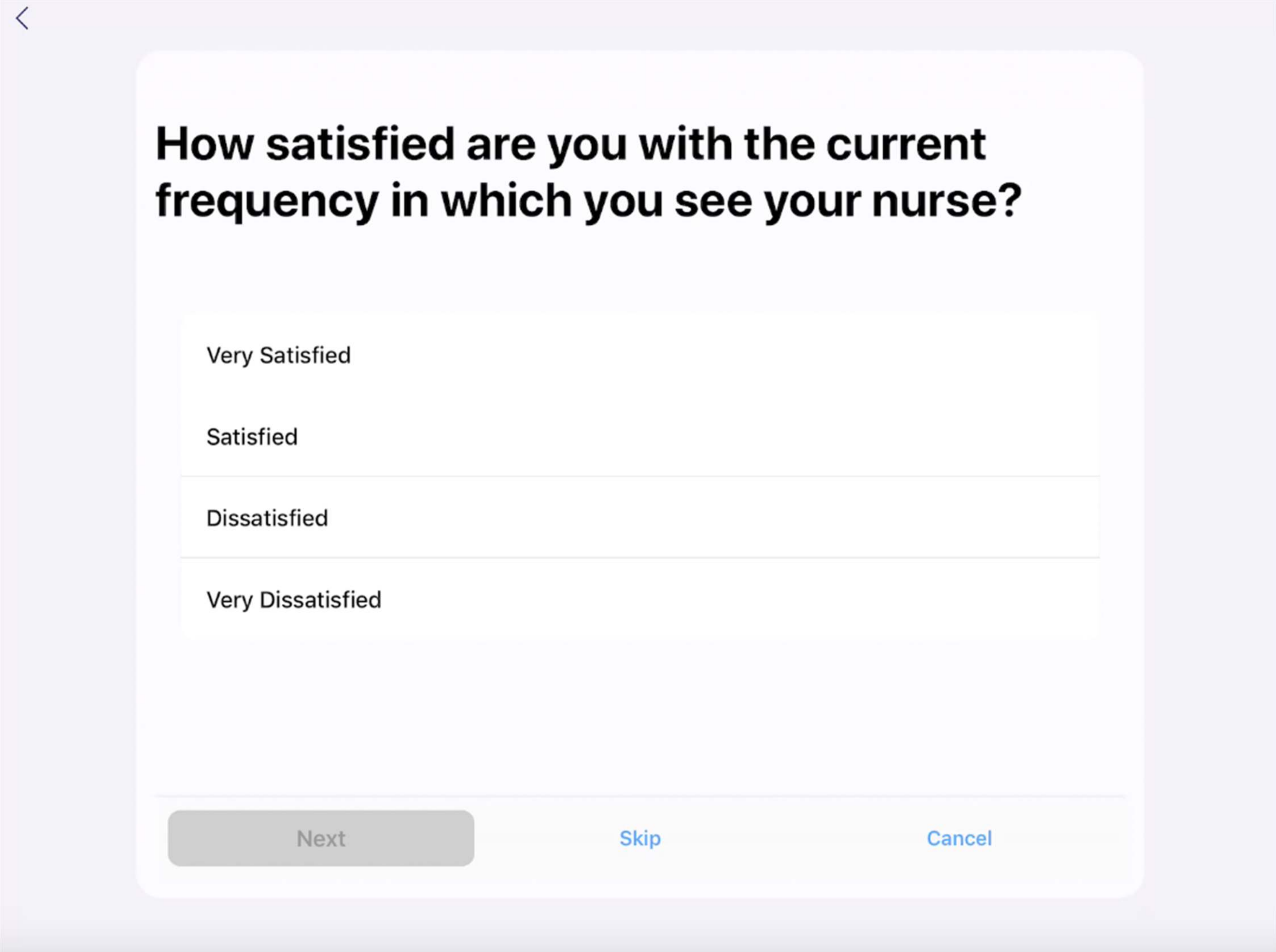
How satisfied are you with the current frequency in which you see your nurse?
Very Satisfied
Satisfied
Dissatisfied
Very Dissatisfied
Next
Skip
Cancel

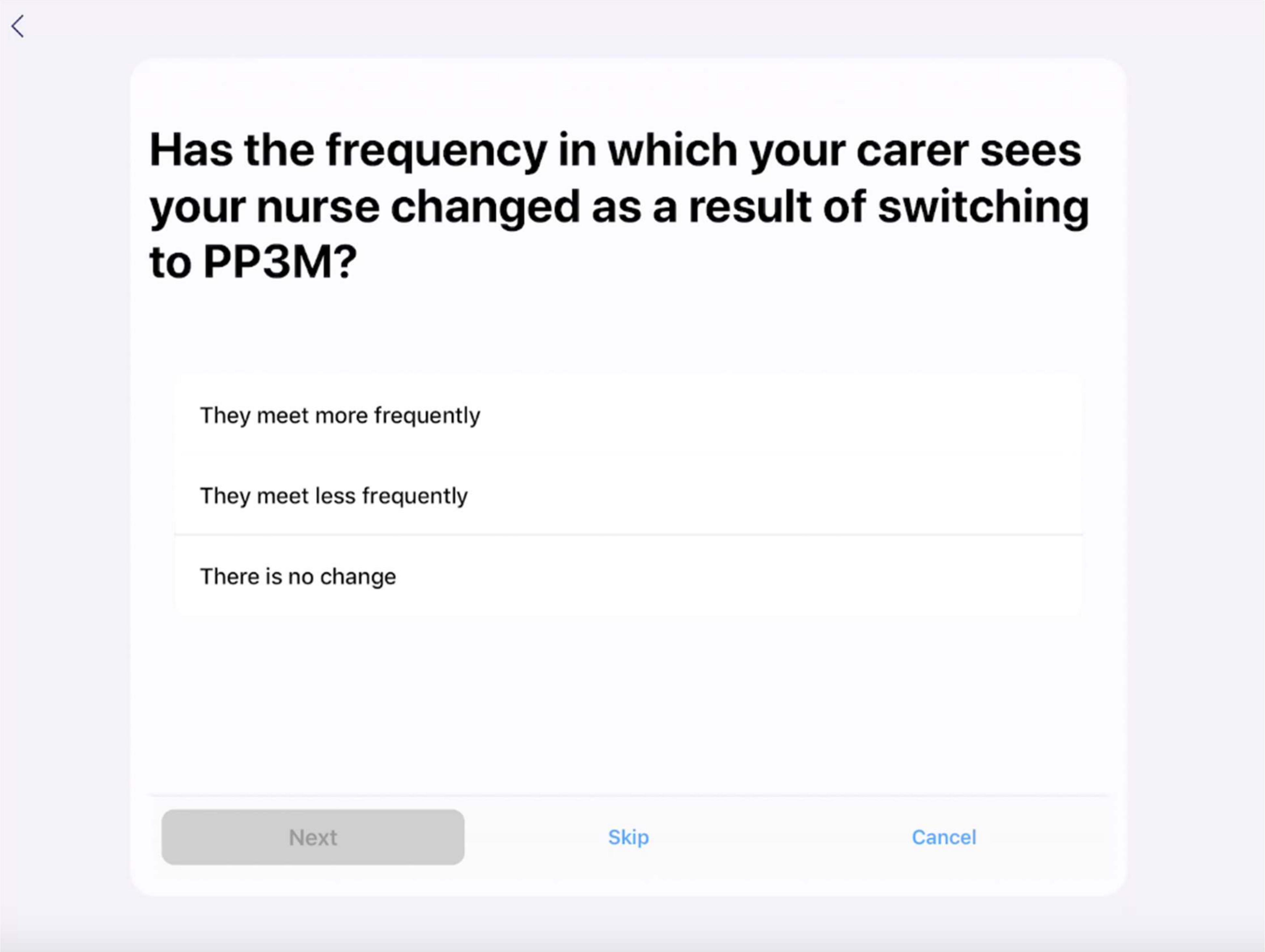
Has the frequency in which your carer sees your nurse changed as a result of switching to PP3M?
They meet more frequently
They meet less frequently
There is no change
Next
Skip
Cancel

How satisfied are you with the current frequency in which your carer sees your nurse?

Very Satisfied

Satisfied

Dissatisfied

Very Dissatisfied

Next

Skip

Cancel

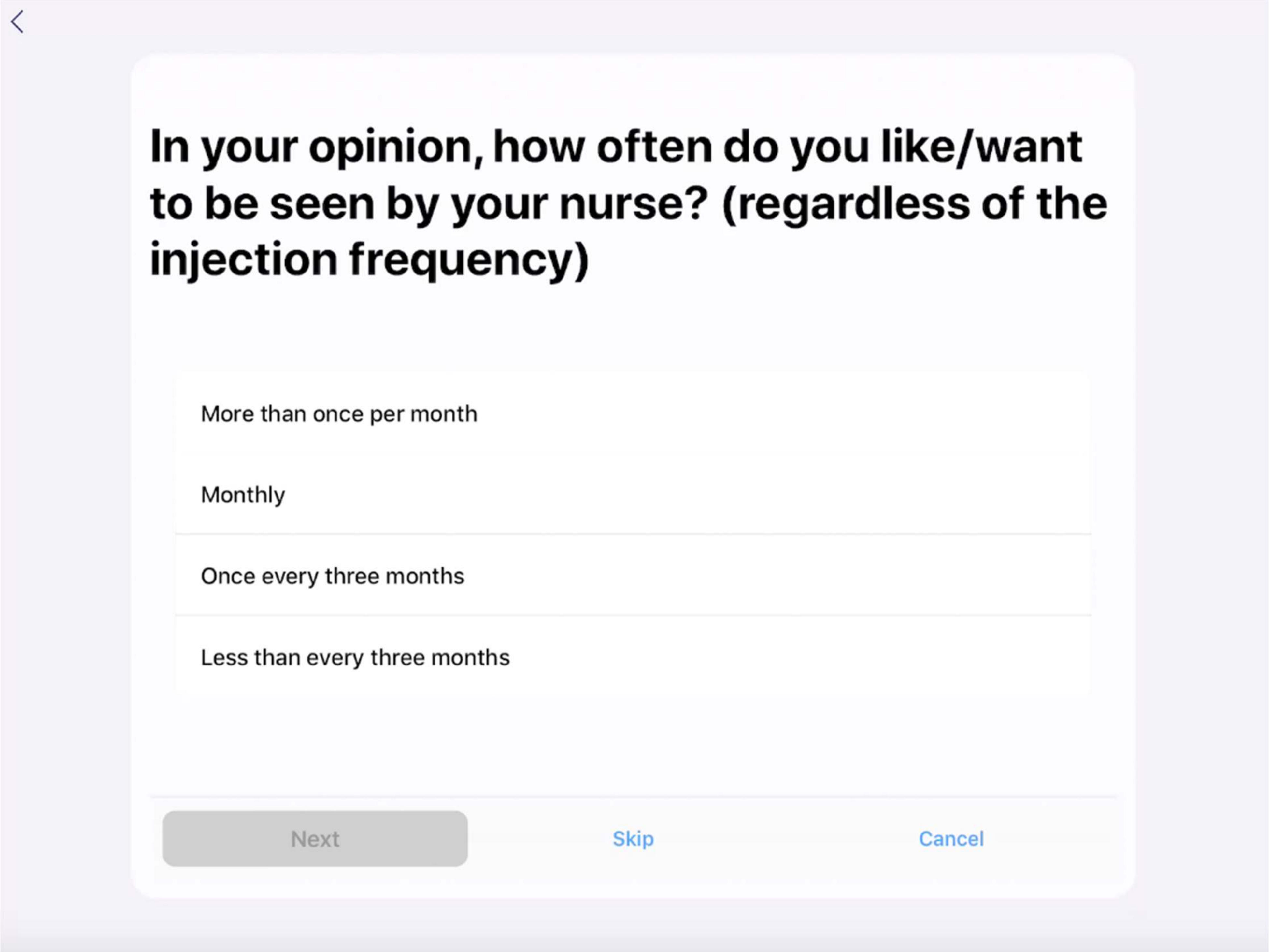
In your opinion, how often do you like/want to be seen by your nurse? (regardless of the injection frequency)
More than once per month
Monthly
Once every three months
Less than every three months
Next
Skip
Cancel

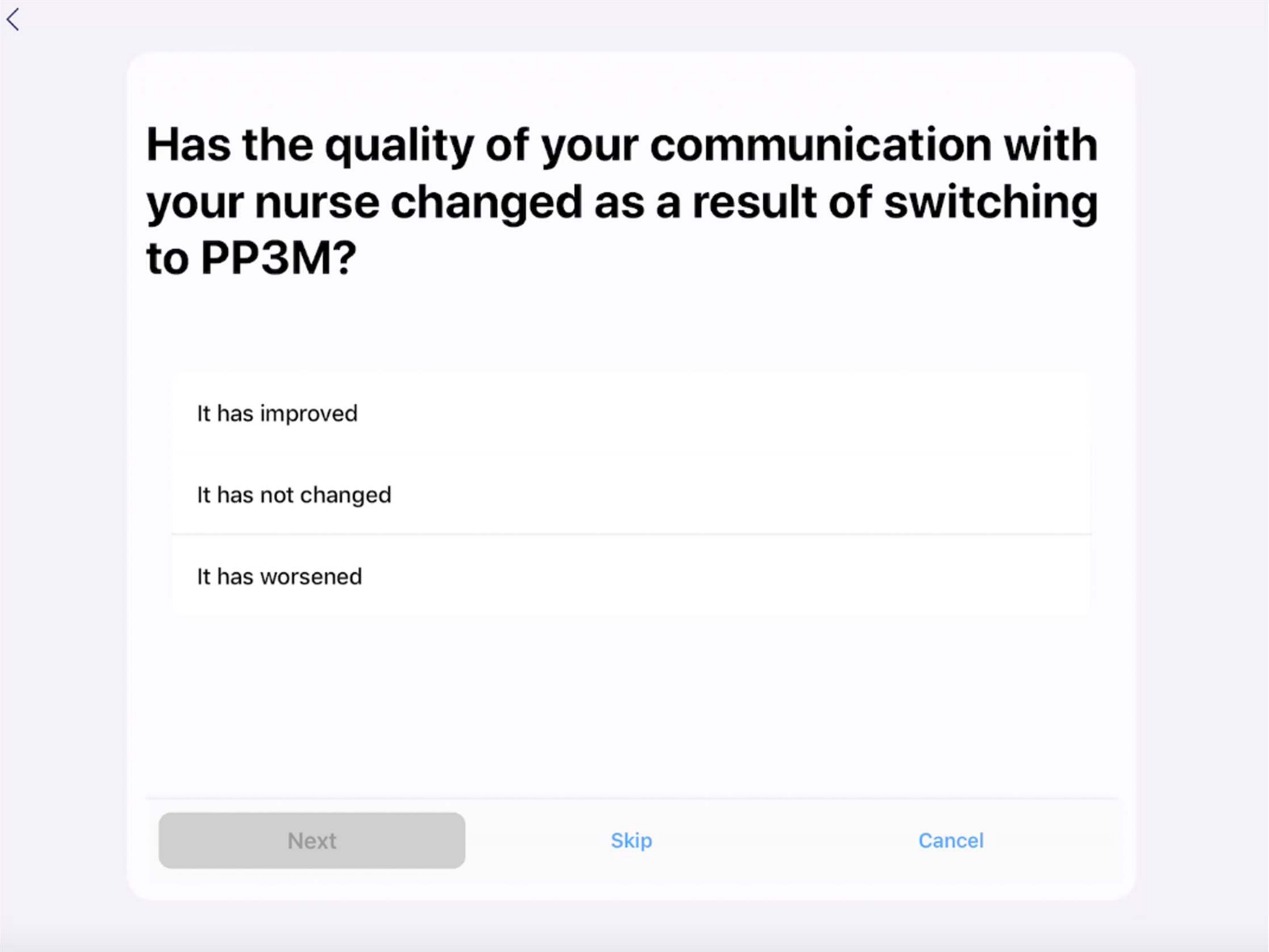
Has the quality of your communication with your nurse changed as a result of switching to PP3M?
It has improved
It has not changed
It has worsened
Next
Skip
Cancel

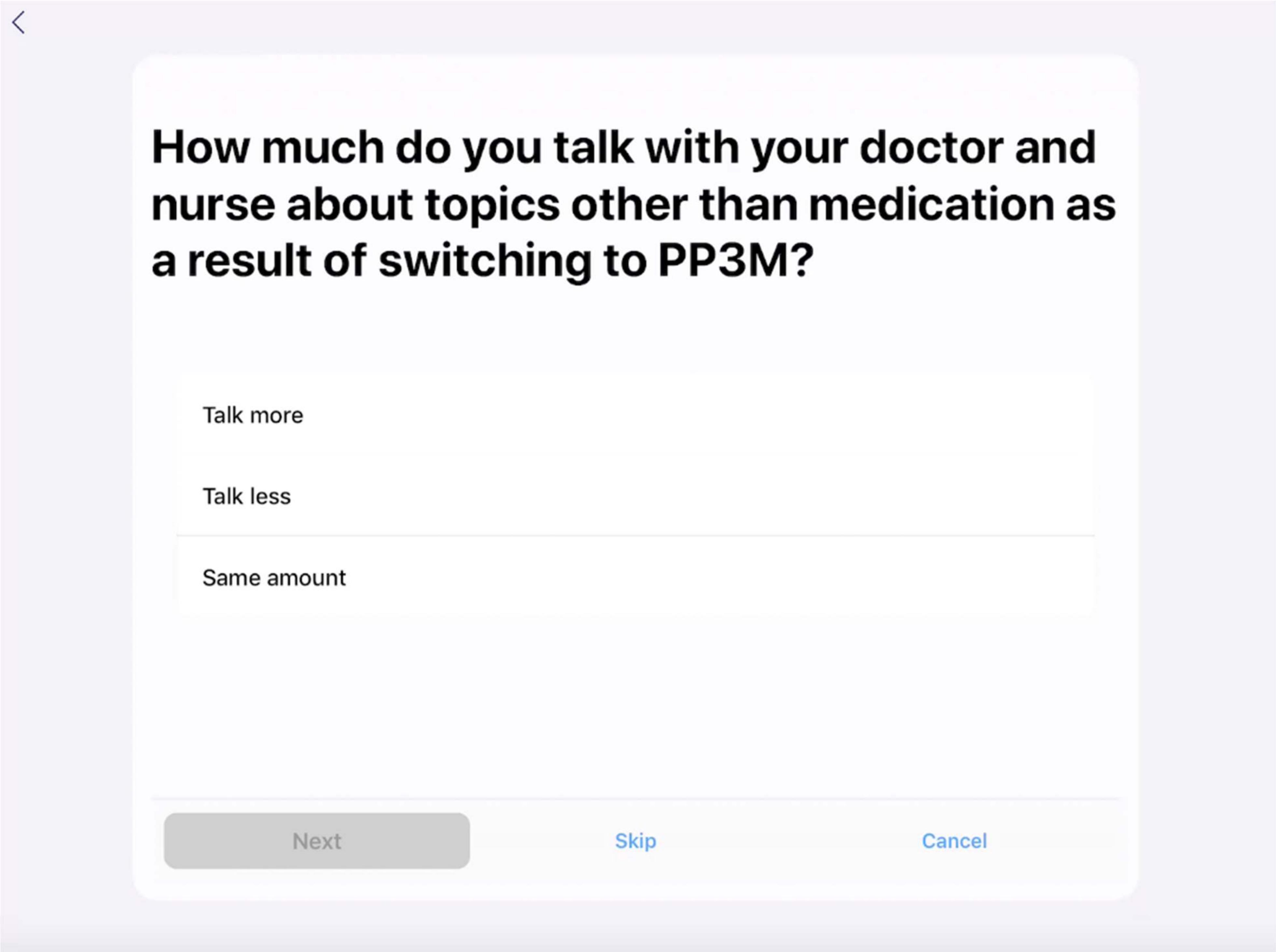
How much do you talk with your doctor and nurse about topics other than medication as a result of switching to PP3M?
Talk more
Talk less
Same amount
Next
Skip
Cancel

IF “Talk More”:

Please indicate topics that you were able to discuss more with your doctor and nurse as a result of switching to PP3M? (multiple answers) Scroll to view all

Family Relationships

Work

Education

External/social communication

Hobbies

Healthy life style guidance (e.g. alcohol, smoking, diet, physical exercise)

Non medication therapy (e.g. psychotherapy)

Other topics

Next   Skip   Cancel

To what extent were you involved in the decision to switch from PP1M to PP3M?

Highly involved

Involved to some extent

Not involved at all

Next

Skip

Cancel

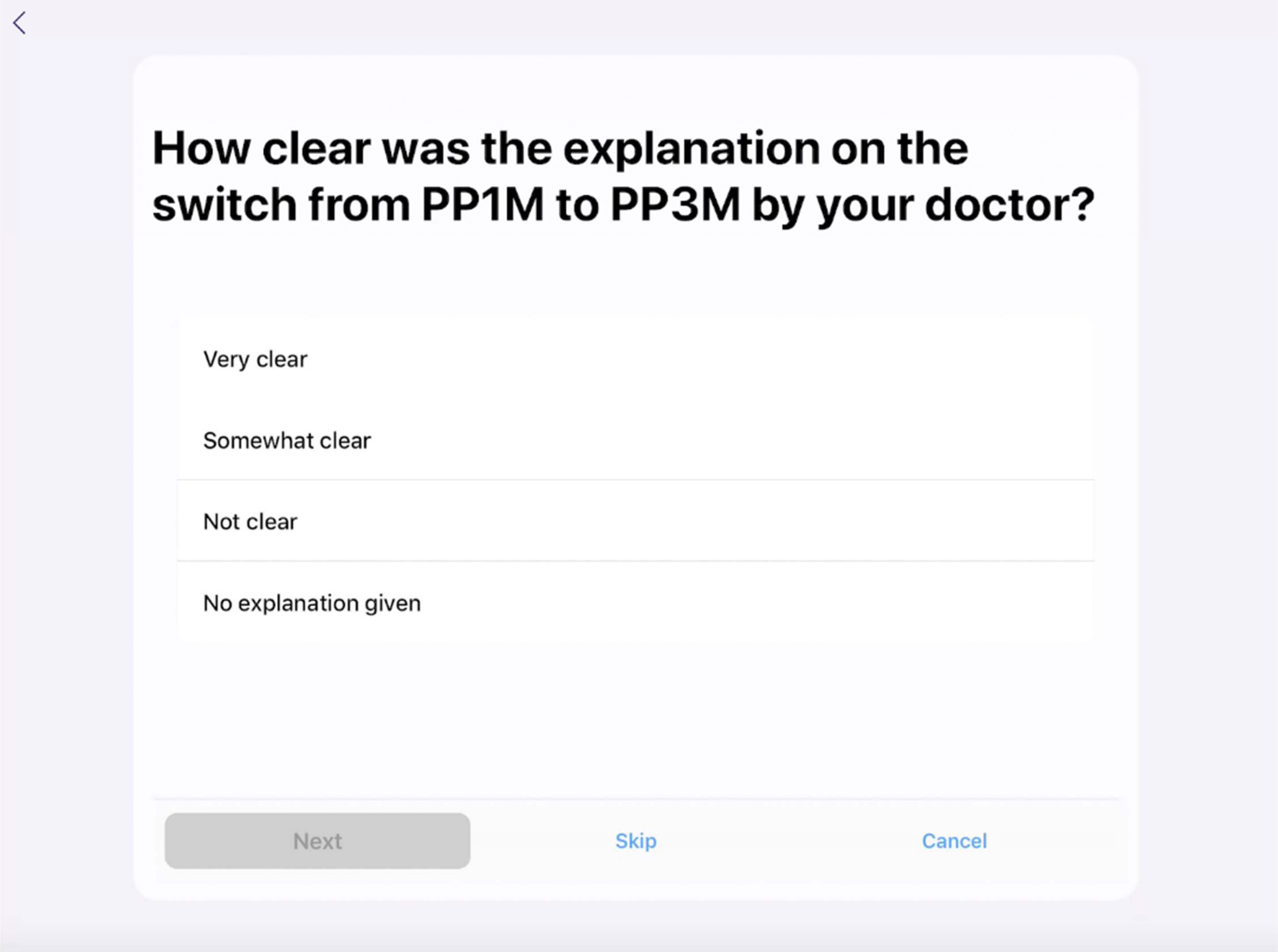
How clear was the explanation on the switch from PP1M to PP3M by your doctor?
Very clear
Somewhat clear
Not clear
No explanation given
Next
Skip
Cancel

Who initiated discussion about switching from PP1M to PP3M?

- The doctor
- The nurse
- It was me
- The carer
- Private service

Next | Skip | Cancel

What were your main reasons to initiate/switch treatment to PP3M? Select all that apply. Scroll to view all

To live a life that is as normal as possible

To reduce side effects

To improve treatment efficacy

To improve treatment adherence

To decrease stigma

For my convenience

Reduce the risk of relapse

Next Skip Cancel

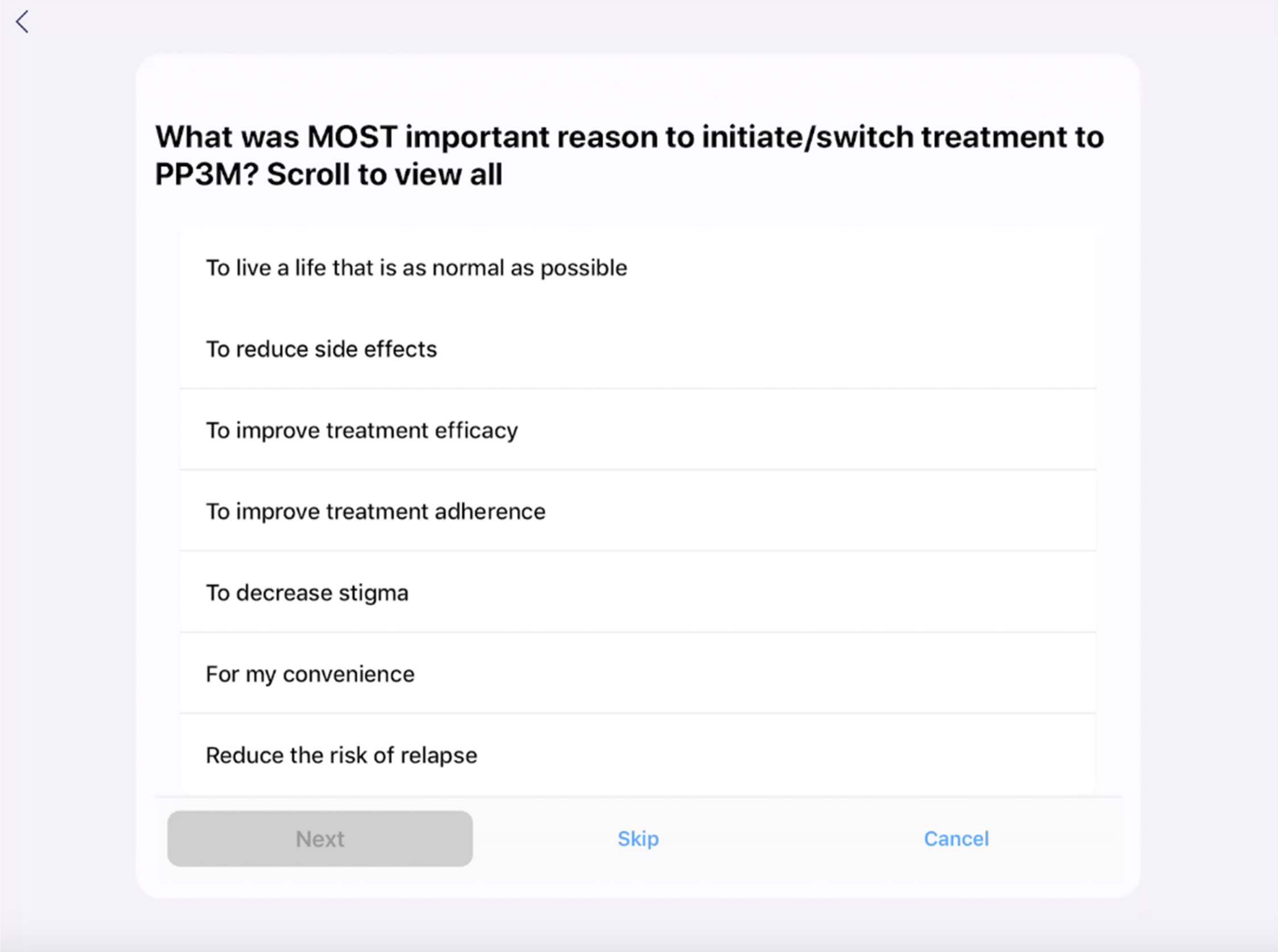
What was MOST important reason to initiate/switch treatment to PP3M? Scroll to view all
To live a life that is as normal as possible
To reduce side effects
To improve treatment efficacy
To improve treatment adherence
To decrease stigma
For my convenience
Reduce the risk of relapse
Next
Skip
Cancel

‹

Select 2nd MOST important reason. Scroll to view all

To live a life that is as normal as possible

To reduce side effects

To improve treatment efficacy

To improve treatment adherence

To decrease stigma

For my convenience

Reduce the risk of relapse

Next | Skip | Cancel

Select 3rd MOST important reason. Scroll to view all

To live a life that is as normal as possible

To reduce side effects

To improve treatment efficacy

To improve treatment adherence

To decrease stigma

For my convenience

Reduce the risk of relapse

Next

Skip

Cancel

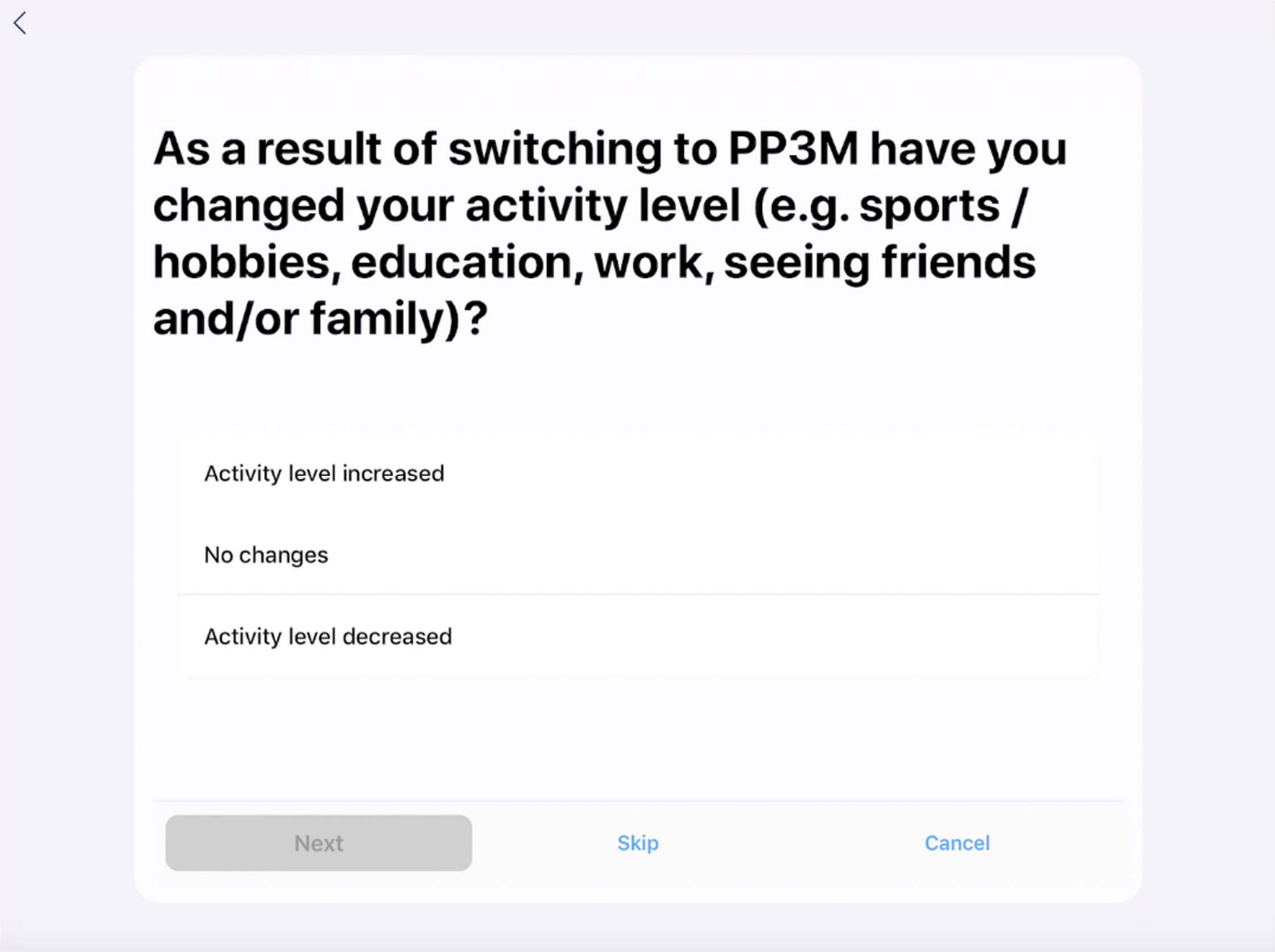
As a result of switching to PP3M have you changed your activity level (e.g. sports / hobbies, education, work, seeing friends and/or family)?
Activity level increased
No changes
Activity level decreased
Next
Skip
Cancel

If “Activity Level Increased”:

Please indicate in what way your activity level was increased as a result of switching to PP3M? (multiple answers)

- I started new sporting activities/hobbies
- I returned to previous sporting activities/hobbies
- I started working again
- I changed job
- I spend more time in social situations

Next Skip Cancel

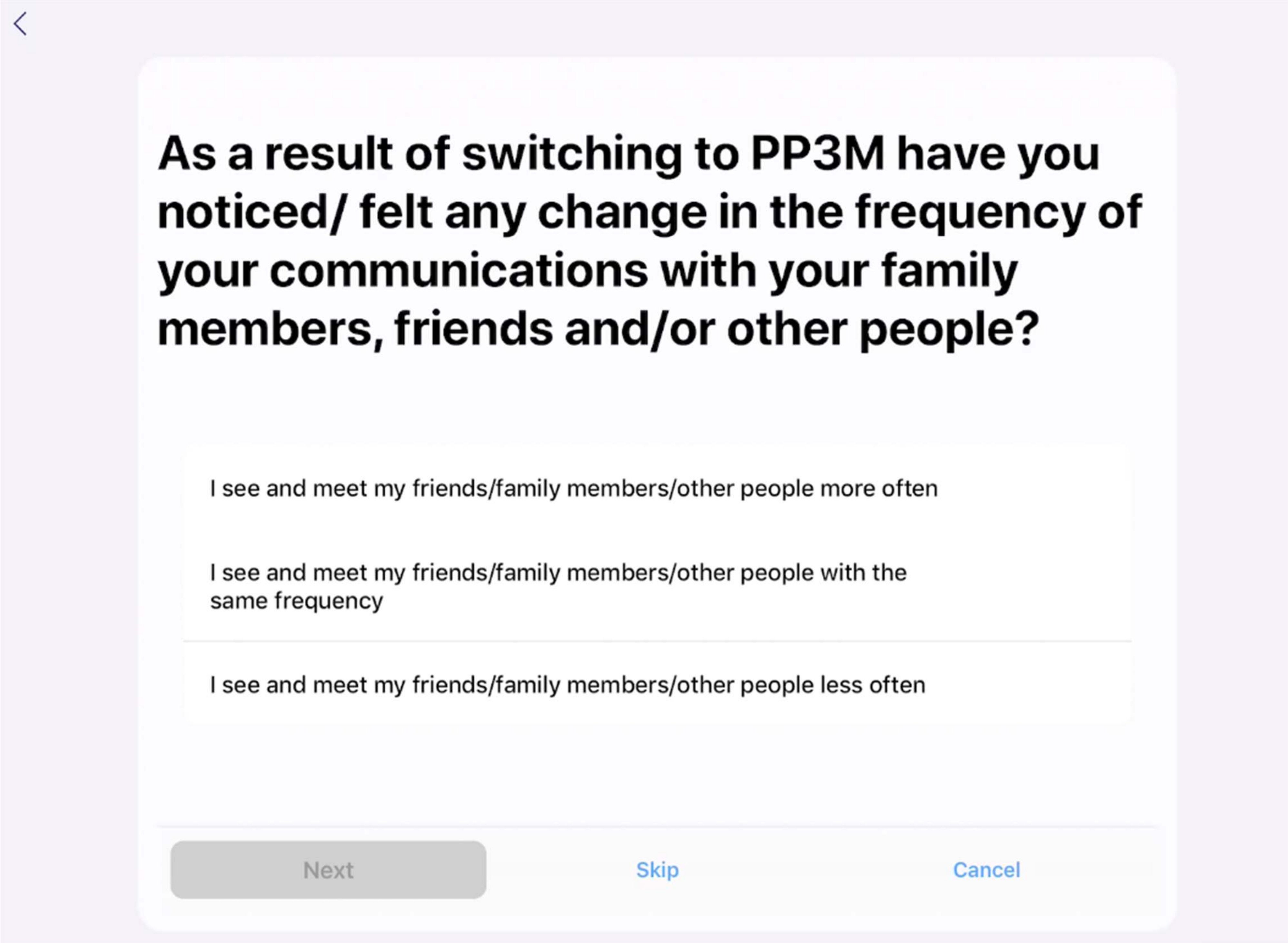
As a result of switching to PP3M have you noticed/ felt any change in the frequency of your communications with your family members, friends and/or other people?
I see and meet my friends/family members/other people more often
I see and meet my friends/family members/other people with the same frequency
I see and meet my friends/family members/other people less often
Next
Skip
Cancel

How stigmatized do you feel you are?

Not at all

A little

Somewhat

Very much

Next

Skip

Cancel

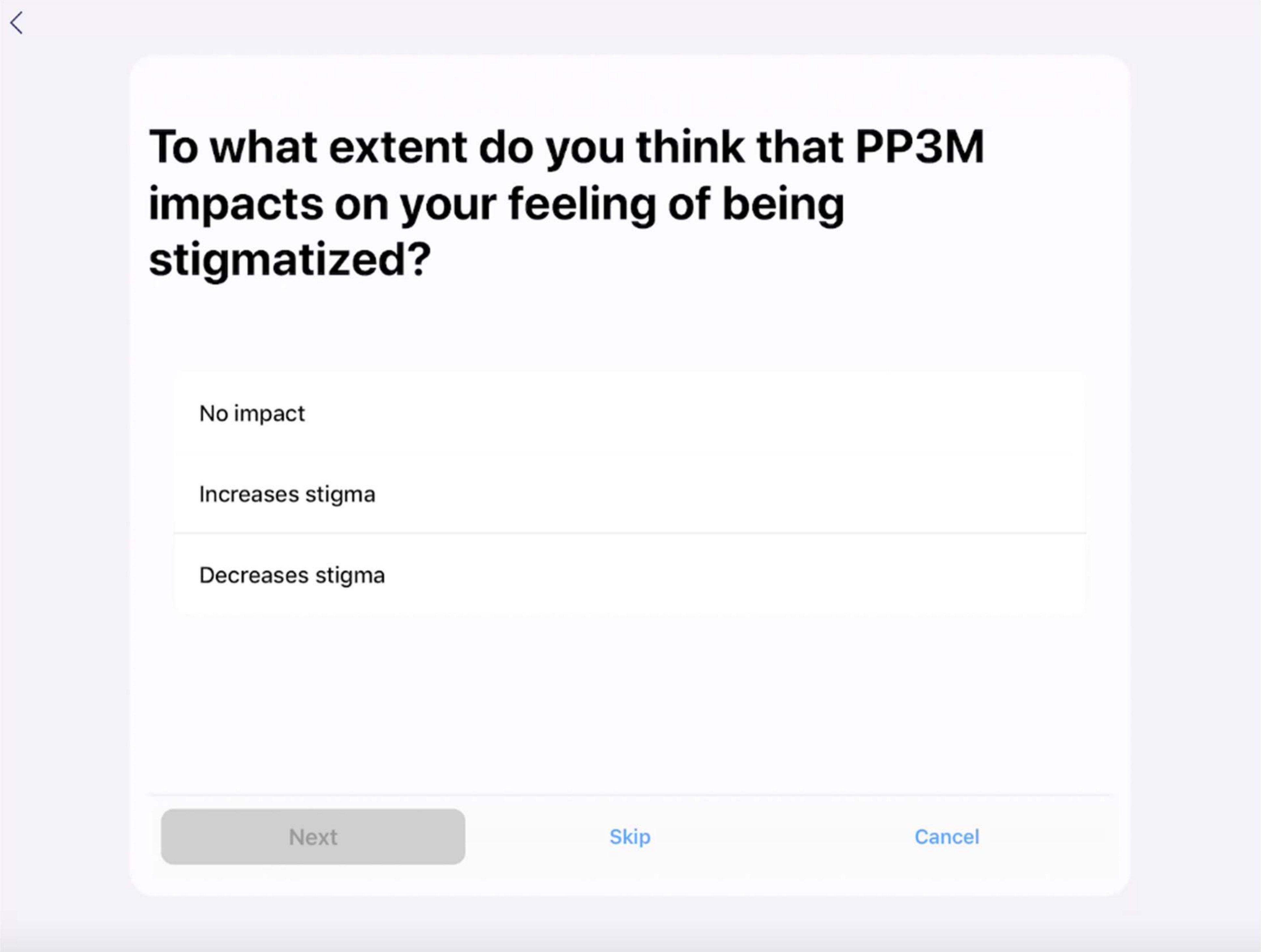
To what extent do you think that PP3M impacts on your feeling of being stigmatized?
No impact
Increases stigma
Decreases stigma
Next
Skip
Cancel

How much do you talk with your carer about topics other than medication as a result of switching to PP3M?

Talk more

Talk less

Same amount

Next

Skip

Cancel

If "TALK MORE";

Please indicate topics that you were able to discuss more with your carer as a result of switching to PP3M? (multiple answers) Scroll to view all

- Your family relationships
- Your work
- Your education
- Your external/social communication
- Your hobbies
- Healthy life style guidance (e.g. alcohol, smoking, diet, physical exercise)
- Non medication therapy (e.g. psychotherapy)
- Other topics

Next | Skip | Cancel

Are you living with your carer?

Yes

No

Next

Skip

Cancel

If “LIVING WITH CARER”:

How many times have you seen your carer during the last 2 months?

Tap to answer times

Next Skip Cancel

Has the frequency in which you see your carer changed as a result of switching to PP3M?

- We meet more frequently
- We meet less frequently
- There is no change
- Not applicable as I am living with my carer

Next Skip Cancel

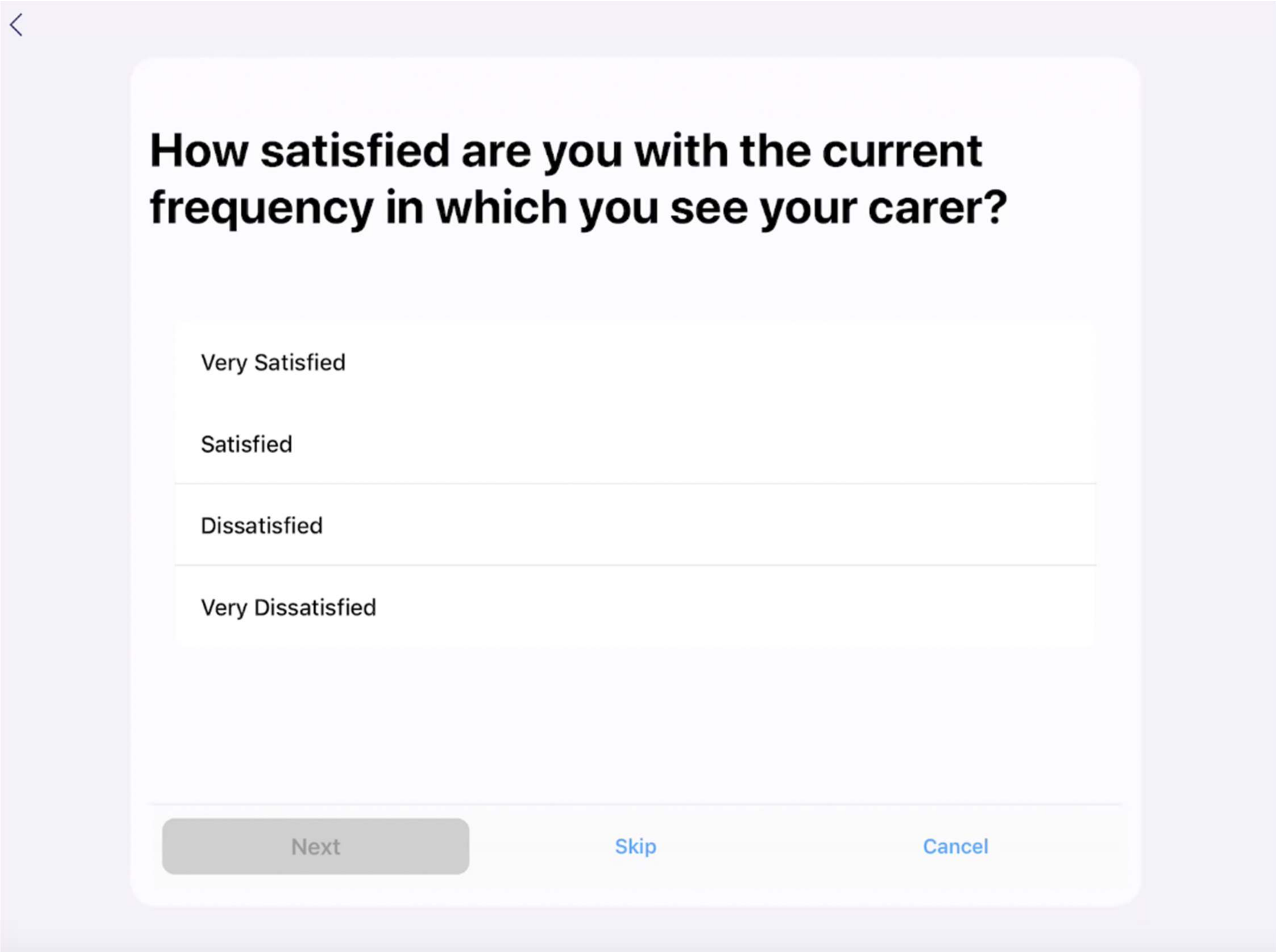
How satisfied are you with the current frequency in which you see your carer?
Very Satisfied
Satisfied
Dissatisfied
Very Dissatisfied
Next
Skip
Cancel

What is your estimate on the total hours per week your carer spends with supporting you (directly with you and managing any additional related tasks)?

Less than 2 hours/week

2-8 hours/week

8-16 hours/week

16-24 hours/week

24-32 hours/week

More than 32 hours/week

Next Skip Cancel

Do you feel that the time required for your support from your carer is less or more than before switching to PP3M?

Less

More

The same

Next

Skip

Cancel

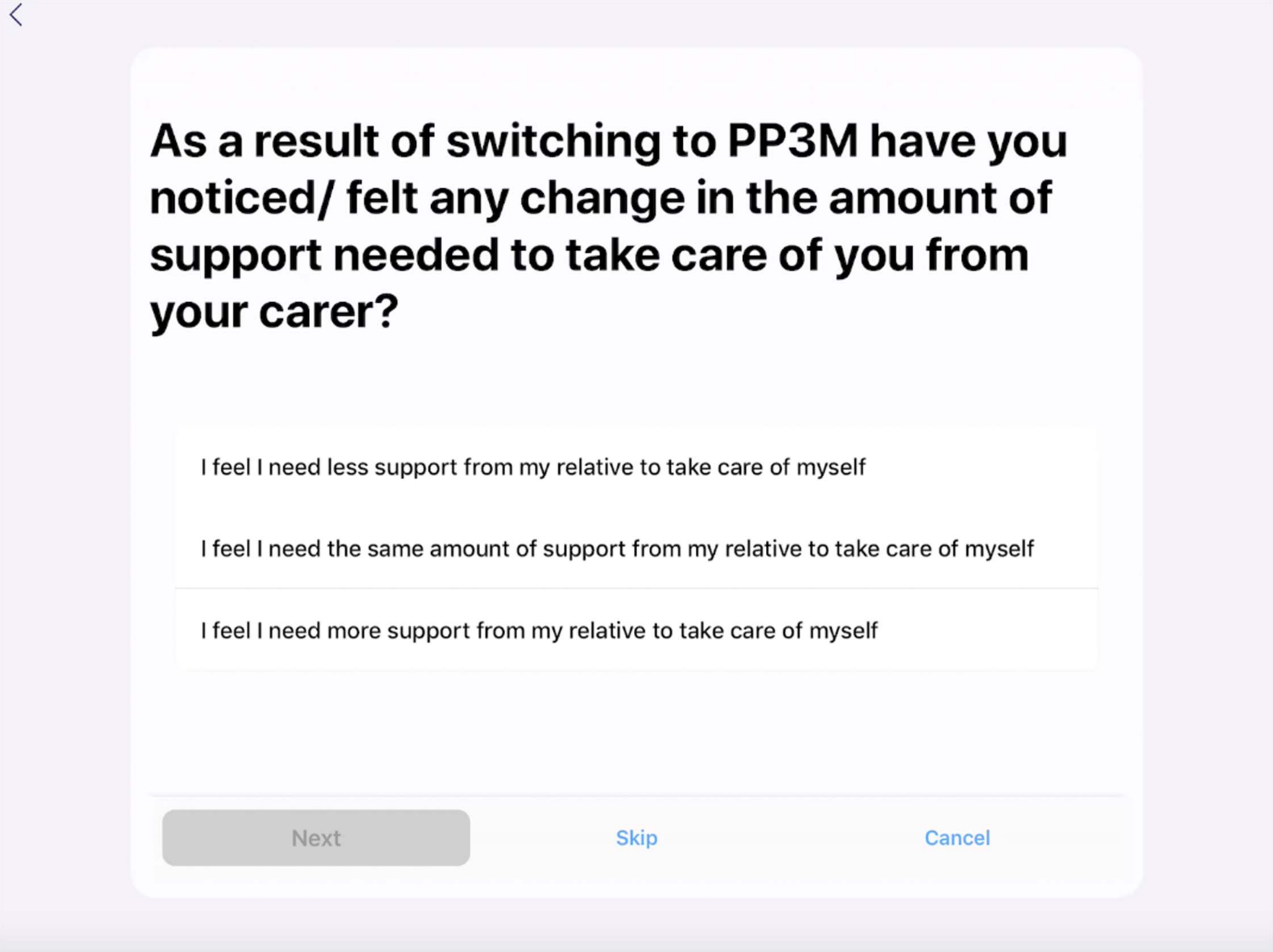
As a result of switching to PP3M have you noticed/ felt any change in the amount of support needed to take care of you from your carer?
I feel I need less support from my relative to take care of myself
I feel I need the same amount of support from my relative to take care of myself
I feel I need more support from my relative to take care of myself
Next
Skip
Cancel

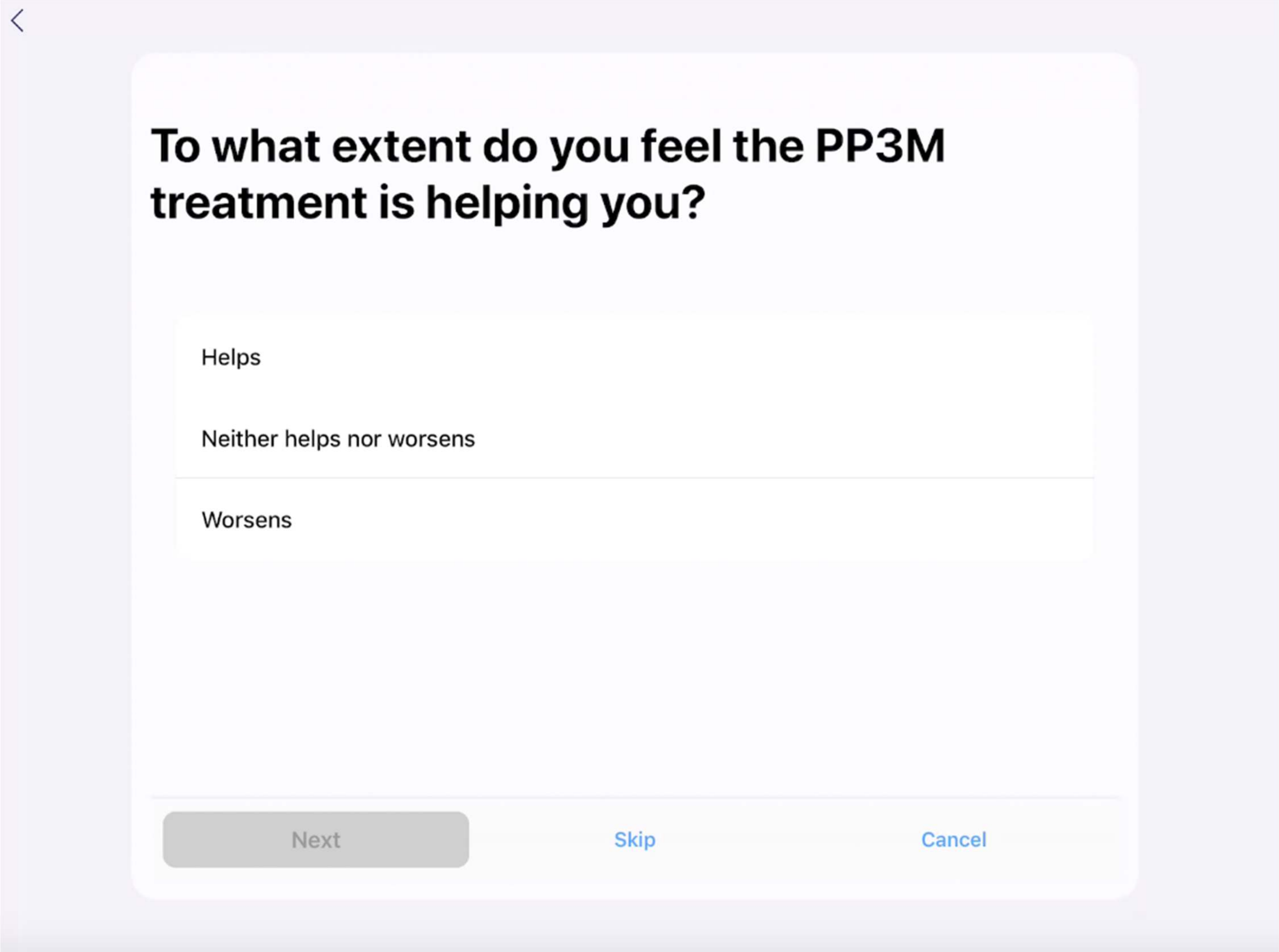
To what extent do you feel the PP3M treatment is helping you?
Helps
Neither helps nor worsens
Worsens
Next
Skip
Cancel

On the next screen, please review all of your answers. If there are any unanswered questions, please confirm that you intended not to answer those questions.

Next

Cancel

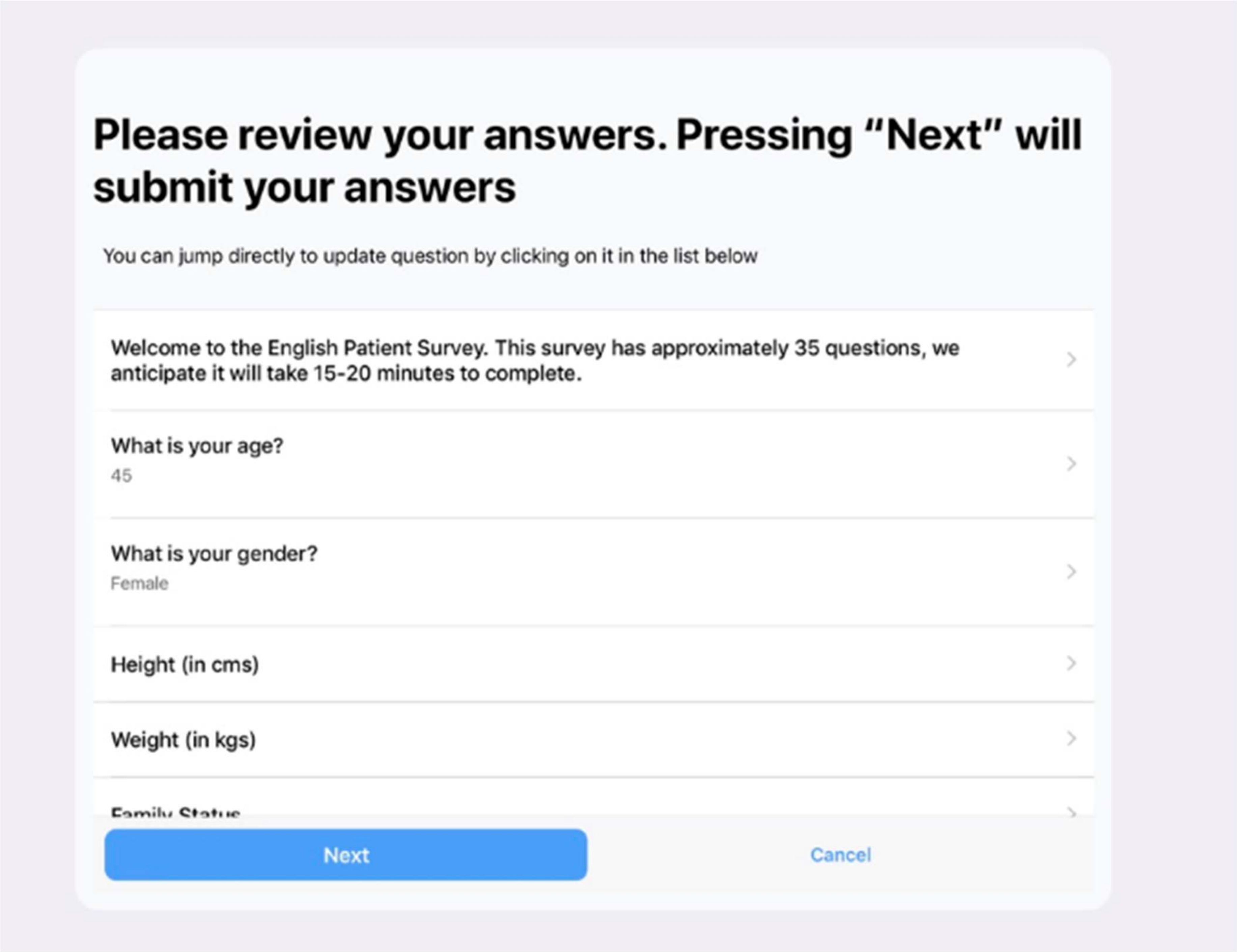
Please review your answers. Pressing "Next" will submit your answers
You can jump directly to update question by clicking on it in the list below
Welcome to the English Patient Survey. This survey has approximately 35 questions, we anticipate it will take 15-20 minutes to complete.
What is your age?
45
What is your gender?
Female
Height (in cms)
Weight (in kgs)
Next
Cancel

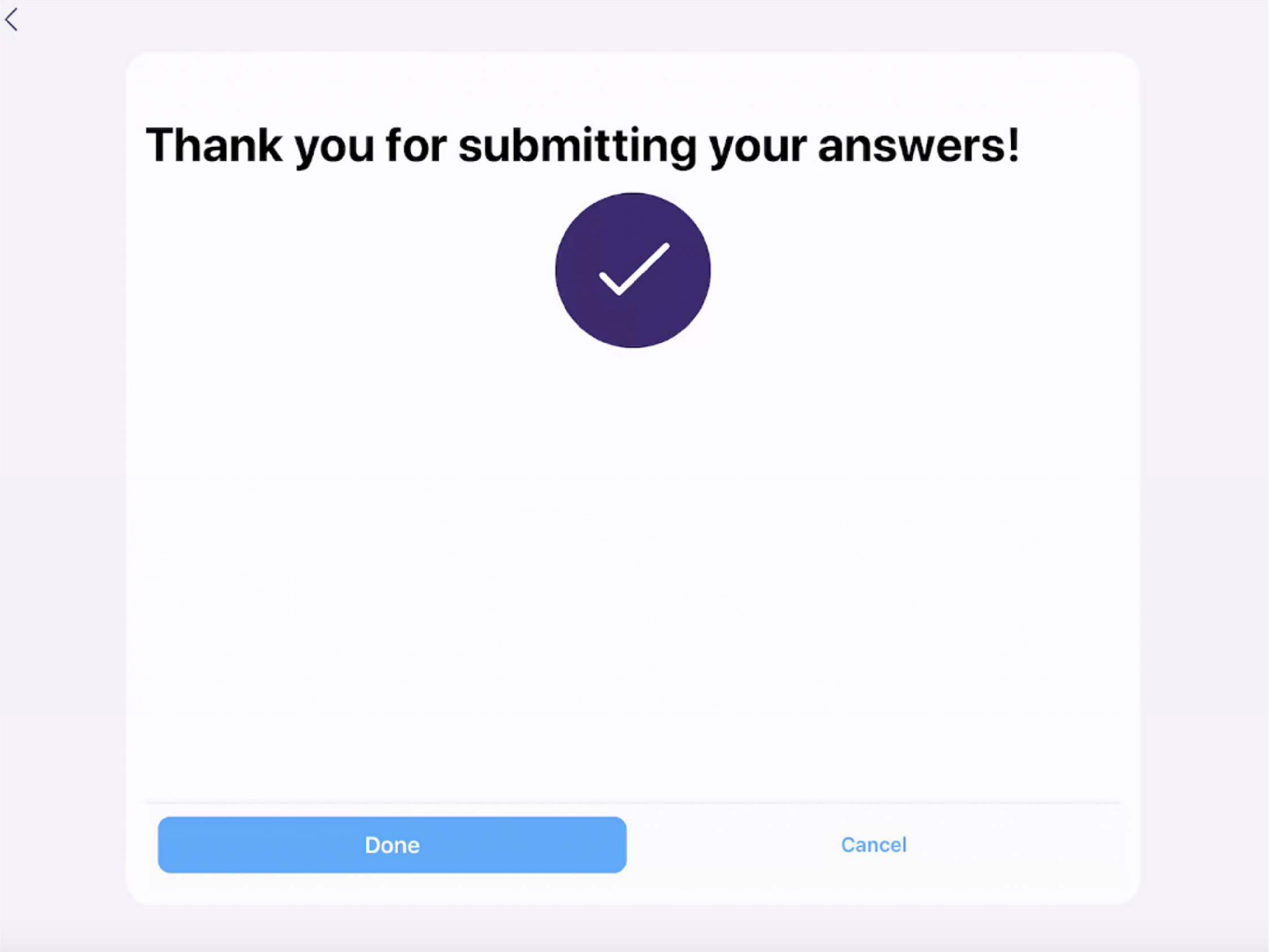
Thank you for submitting your answers!
Done
Cancel
