# Supplementary material for: The Patient, Investigator, Nurse, Carer Questionnaire (PINC-Q): a cross-sectional, retrospective, non-interventional study exploring the impact of less frequent medication administration with paliperidone palmitate 3-monthly as maintenance treatment for schizophrenia
Source: BMC Psychiatry. 2021 Jun 9;21:300. doi: 10.1186/s12888-021-03305-z (PMC8191017; doi:10.1186/s12888-021-03305-z)
Supplement: Supplementary file 8 — Additional file 8. English carer questionnaire. [file 12888_2021_3305_MOESM8_ESM.pdf]

English Carer Survey

Welcome to the English Carer Survey. This survey has approximately 50 questions, we anticipate it will take 15-20 minutes to complete.

Get Started

Cancel

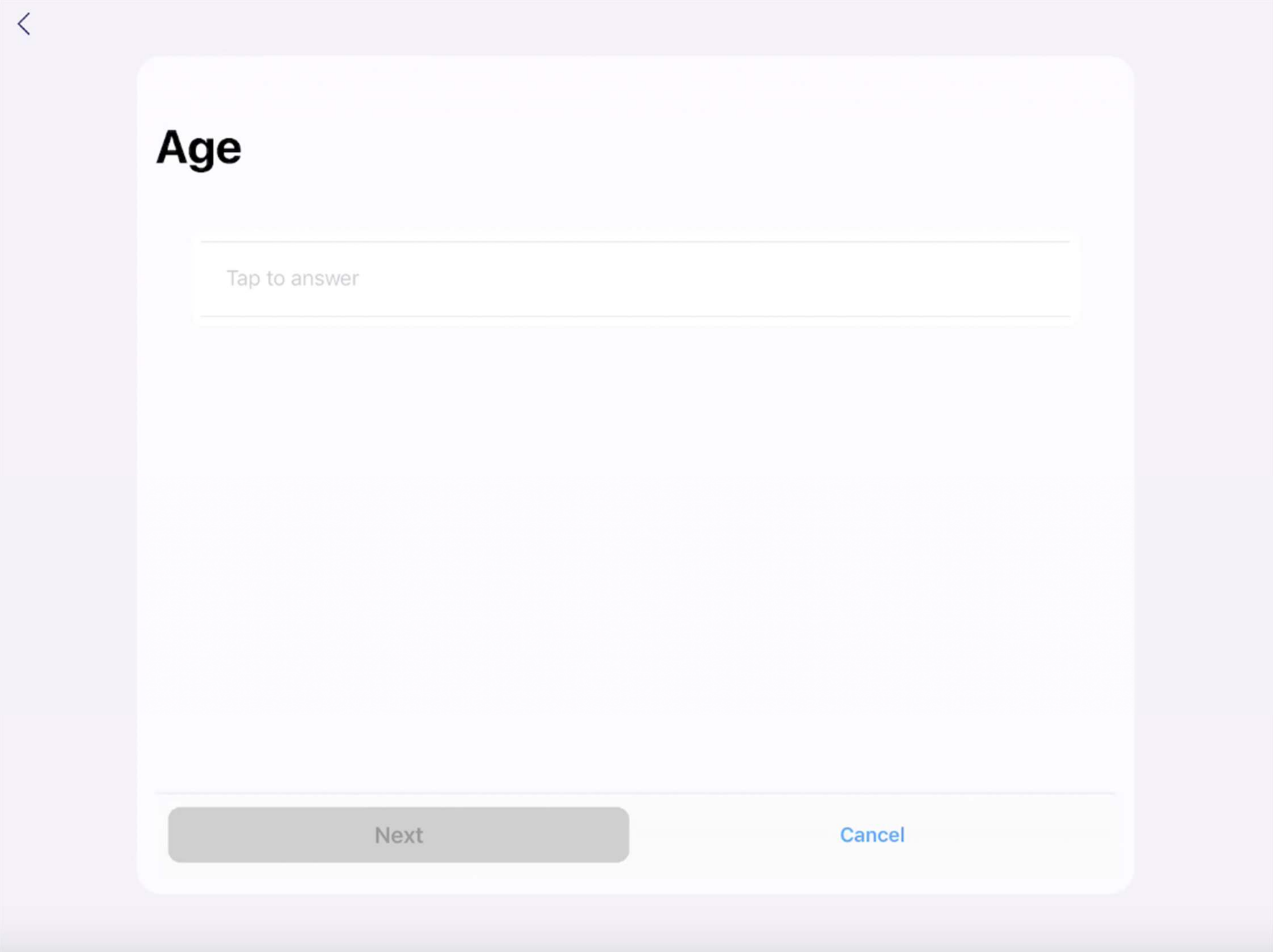
Age
Tap to answer
Next
Cancel

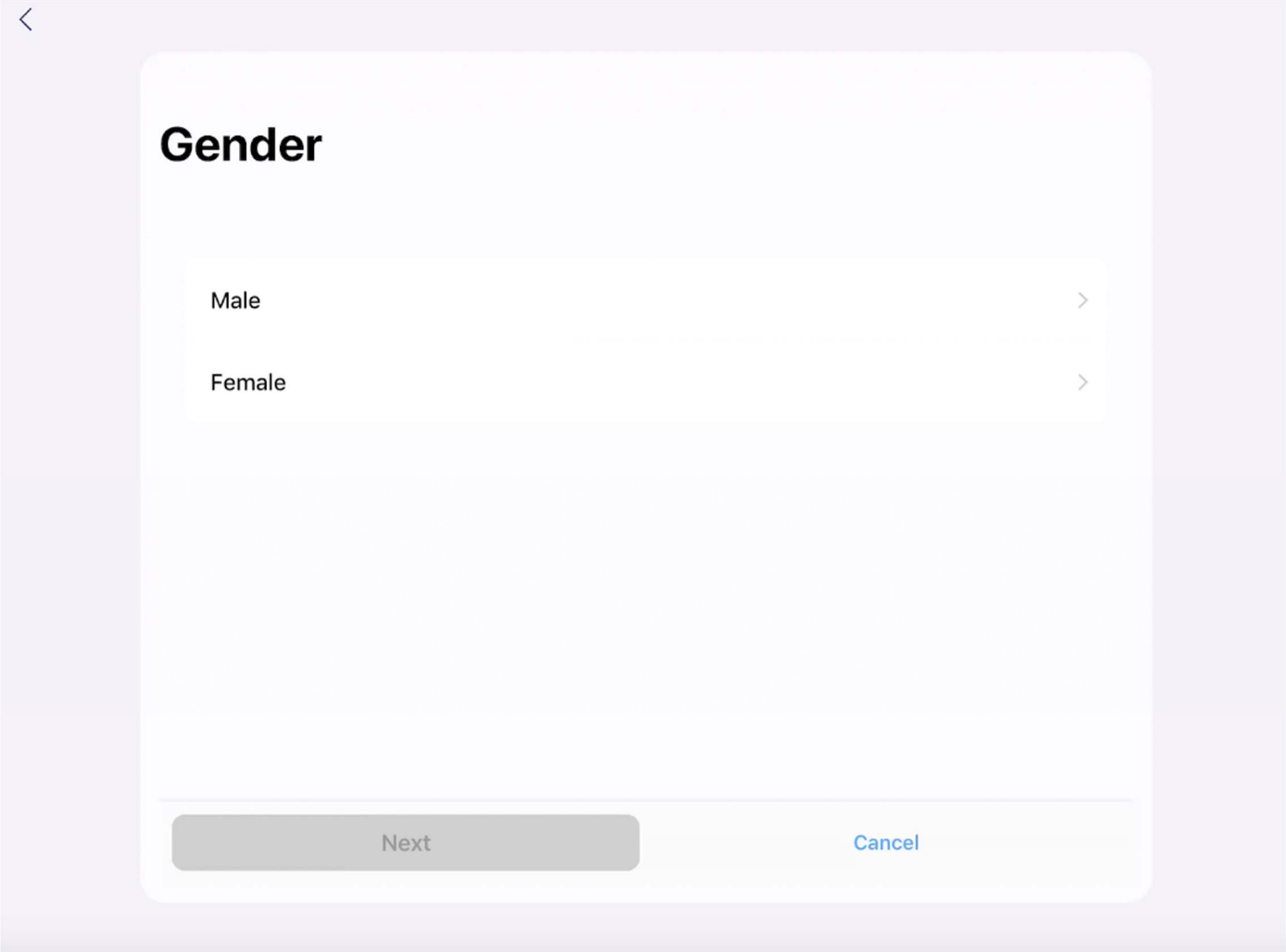
Gender
Male
Female
Next
Cancel

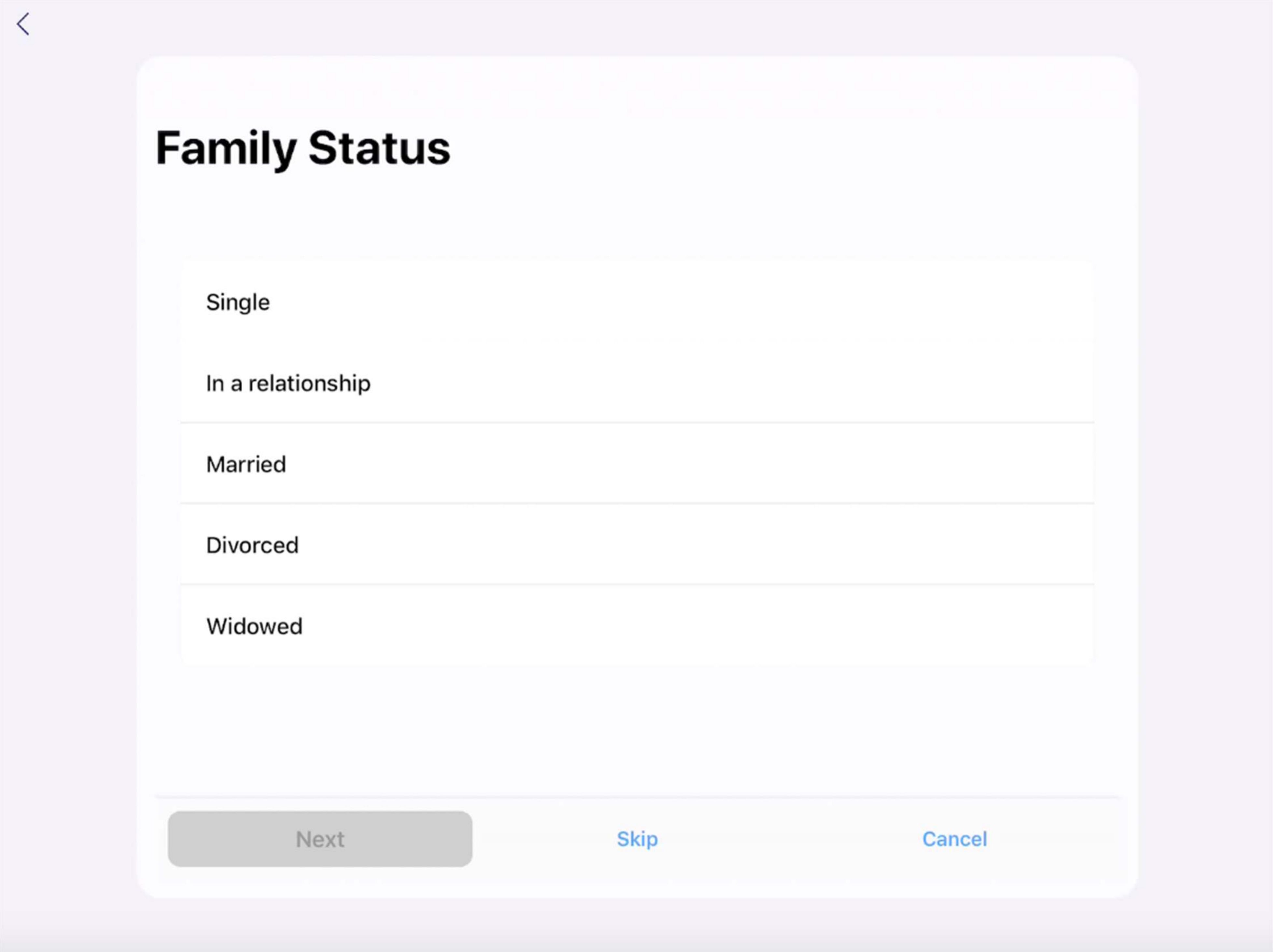
Family Status
Single
In a relationship
Married
Divorced
Widowed
Next
Skip
Cancel

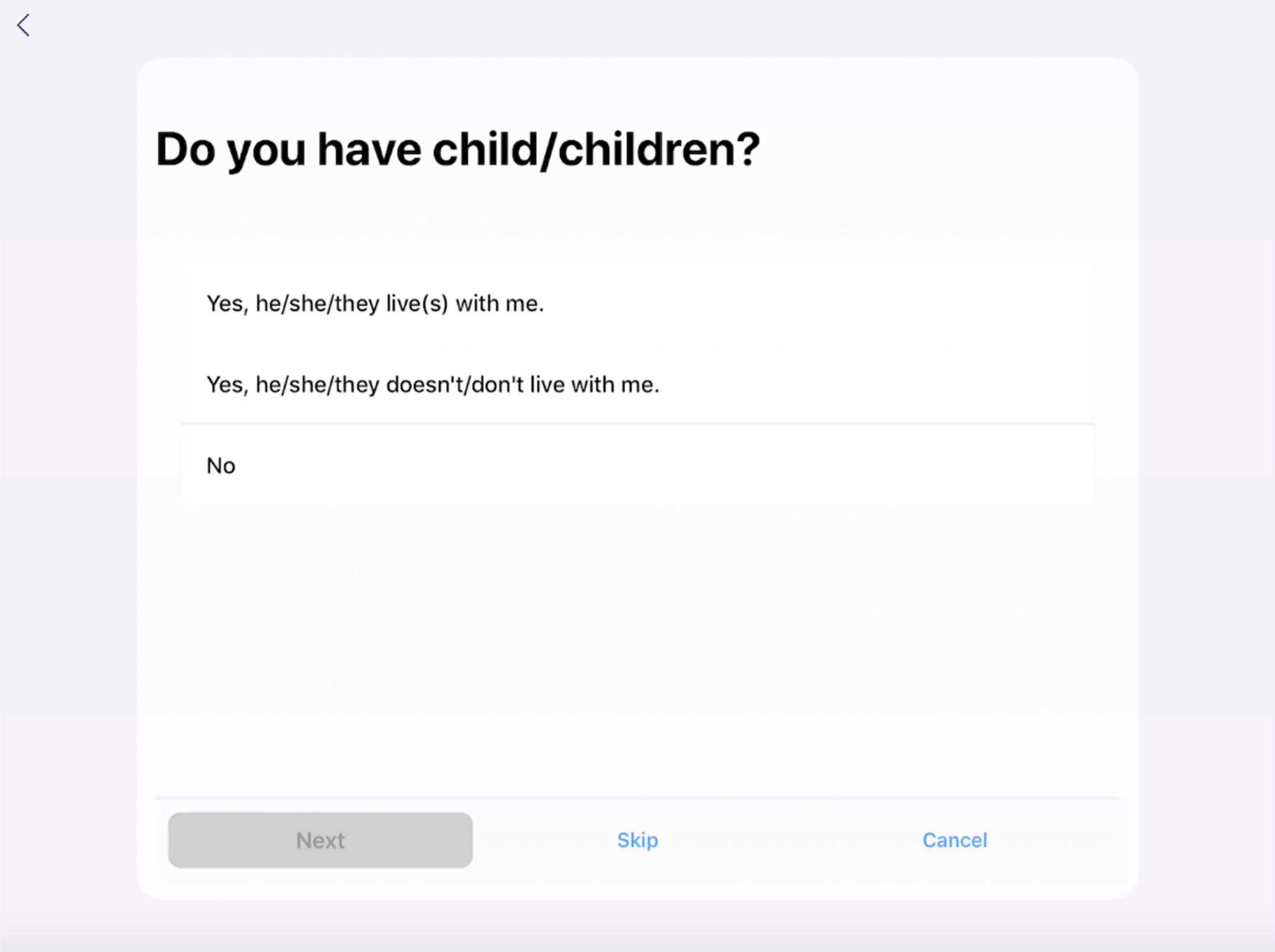
Do you have child/children?
Yes, he/she/they live(s) with me.
Yes, he/she/they doesn't/don't live with me.
No
Next
Skip
Cancel

Living arrangement:

- With family (parents / siblings)
- With spouse / partner / children
- Alone
- In cohabitation (friends, roommates.......)
- In community (workers home/hostels, student residence ....)
- In institution
- No fixed abode/homeless

Next   Skip   Cancel

How many people living with schizophrenia do you currently take care of?

One person

More than one person

Next

Skip

Cancel

Do you also take care of other people dealing with other mental health disorders?

Yes

No

Next

Skip

Cancel

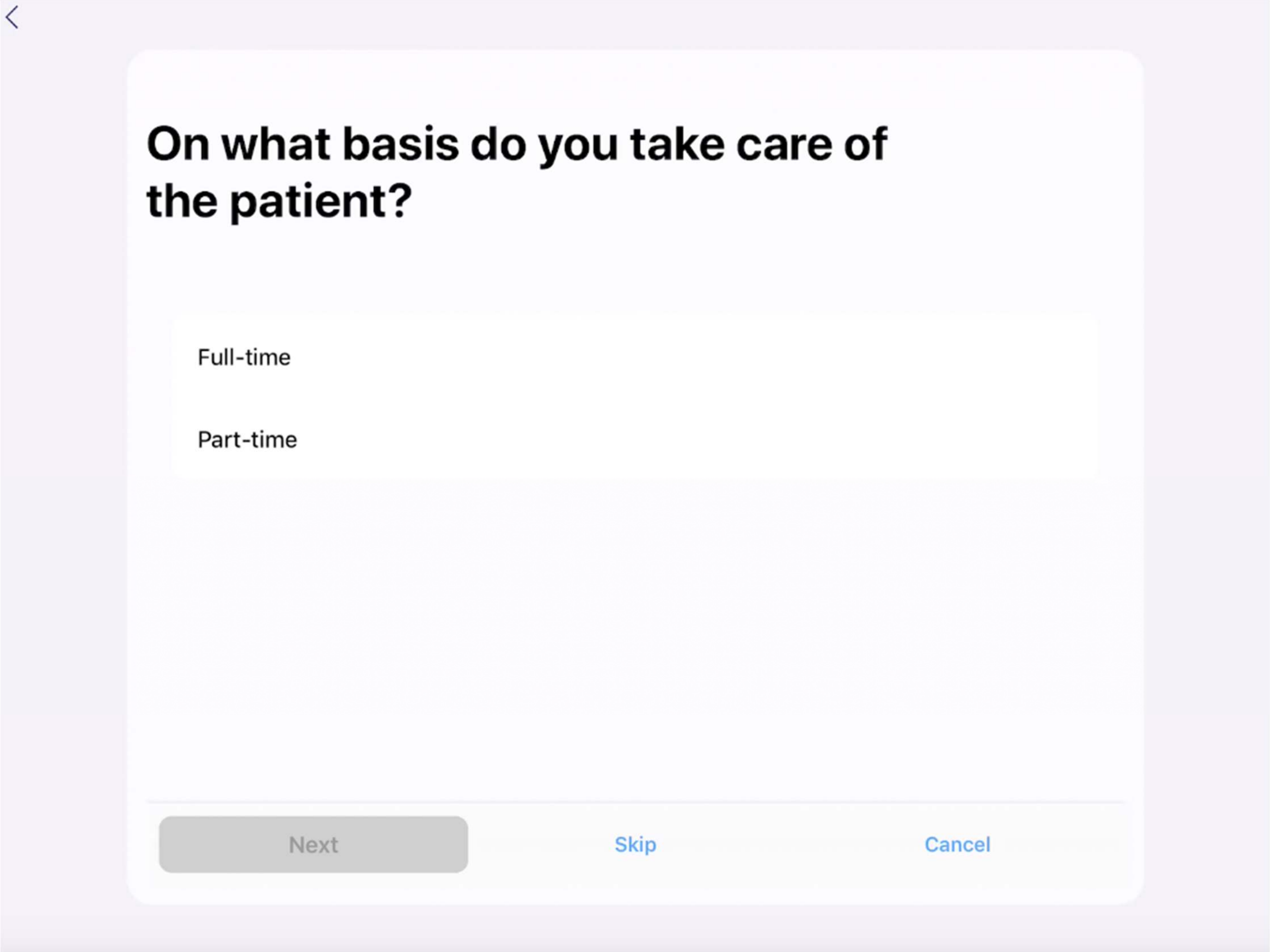
On what basis do you take care of the patient?
Full-time
Part-time
Next
Skip
Cancel

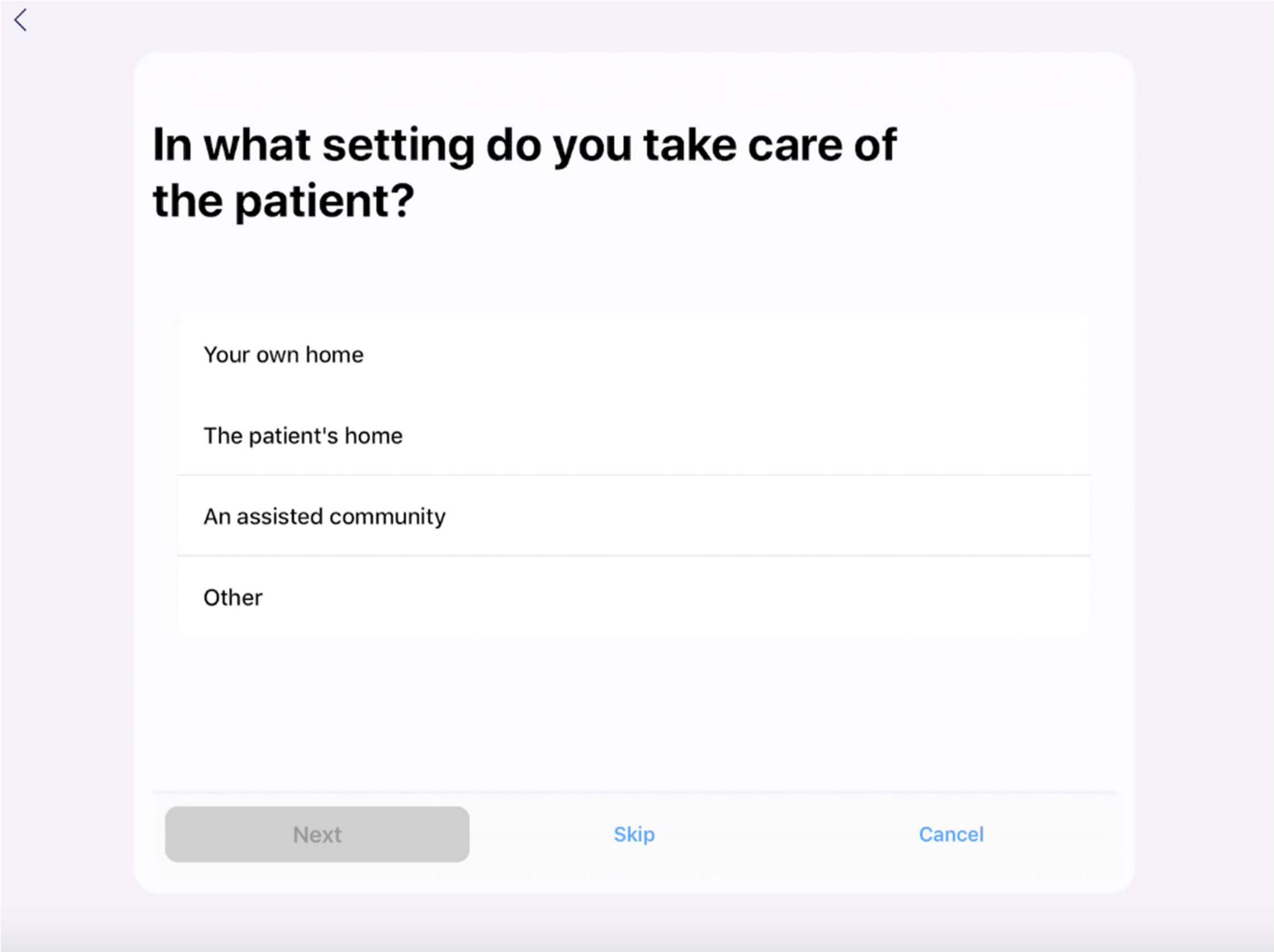
In what setting do you take care of the patient?
Your own home
The patient's home
An assisted community
Other
Next
Skip
Cancel

Indicate below your relationship with the person suffering from schizophrenia. Scroll to view all

Parent

Child

Sibling

Spouse/Partner

Friend

Other family member

Someone else

Next

Skip

Cancel

If “Someone Else”:

Are you professional carer and being paid for taking care of this patient?

Yes

No

Next

Skip

Cancel

Do you live together with the person you take care for?

Yes

No

Next

Skip

Cancel

How long do you know the person you take care for?

Less than 1 year

1-3 years

3-5 years

5-10 years

More than 10 years

Next

Skip

Cancel

Whom do you find important to involve in the treatment decision? (multiple answers)

Patient

Carer

Doctor

Nurse

Other

Next | Skip | Cancel

Are you in education or employment?

Yes

No

Next

Skip

Cancel

‹

If YES, please specify:

Employee, paid

Employee, voluntary

Employee in specialized / protected employment settings

Self-employed worker

Student, professional course/training

Next

Skip

Cancel

If NO, please specify:

Unemployed

Receiving a disability allowance

Seeking employment

Next

Skip

Cancel

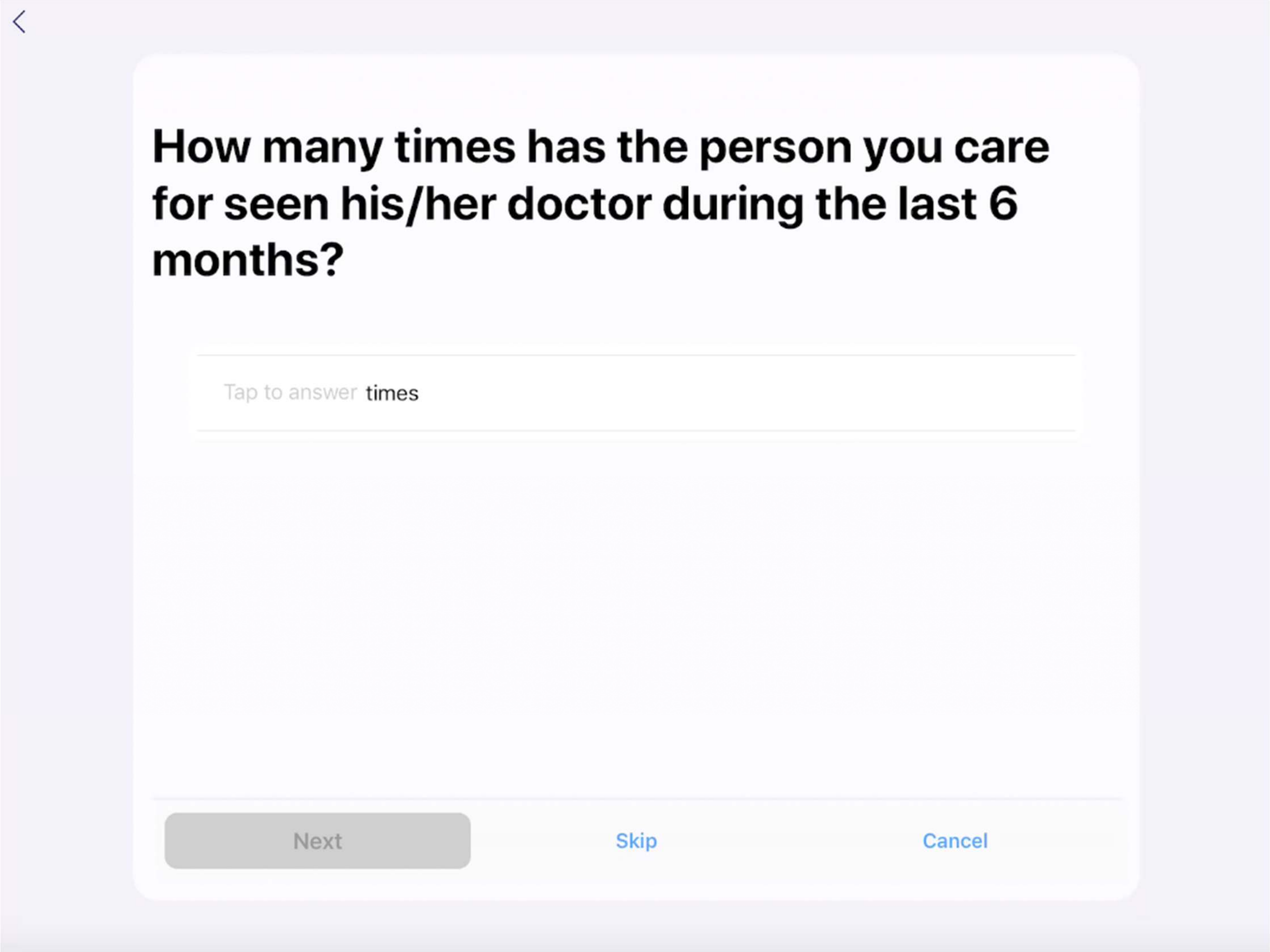
How many times has the person you care for seen his/her doctor during the last 6 months?
Tap to answer times
Next
Skip
Cancel

How many times have you seen the doctor of the person you care for during the last 6 months?

Tap to answer times

Next Skip Cancel

Has the frequency in which the person you care for sees his/her doctor changed as a result of switching to PP3M?

He/she meets more frequently

He/she meets less frequently

There is no change

Next   Skip   Cancel

How satisfied are you with the current frequency in which the person you care for sees his/her doctor?

Very Satisfied

Satisfied

Dissatisfied

Very Dissatisfied

Next

Skip

Cancel

Has the frequency in which you see the doctor of the person you care for changed as a result of switching to PP3M?

We meet more frequently

We meet less frequently

There is no change

Next

Skip

Cancel

How satisfied are you with the current frequency in which you see the doctor of the person you care for?

Very Satisfied

Satisfied

Dissatisfied

Very Dissatisfied

Next Skip Cancel

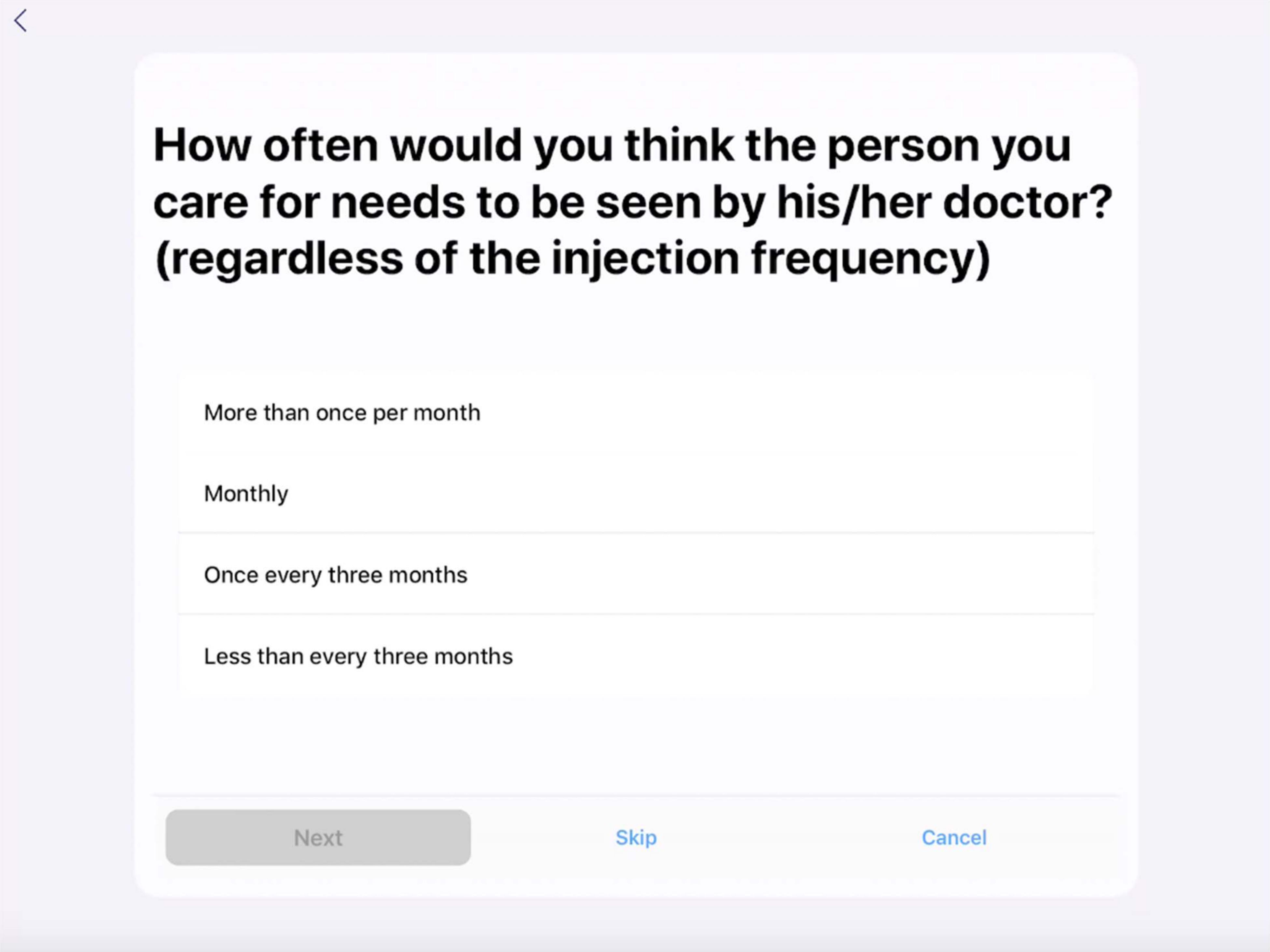
How often would you think the person you care for needs to be seen by his/her doctor? (regardless of the injection frequency)
More than once per month
Monthly
Once every three months
Less than every three months
Next
Skip
Cancel

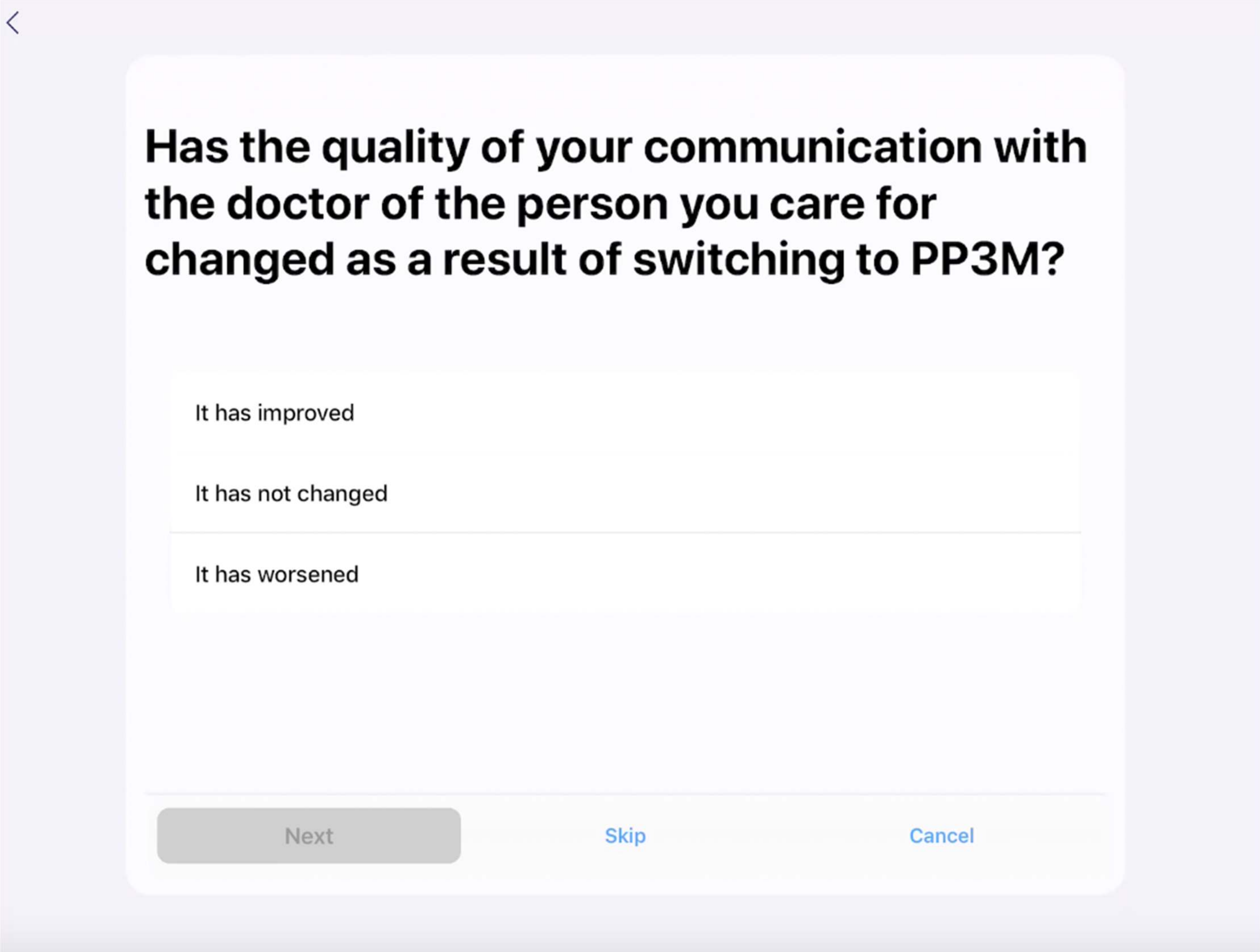
Has the quality of your communication with the doctor of the person you care for changed as a result of switching to PP3M?
It has improved
It has not changed
It has worsened
Next
Skip
Cancel

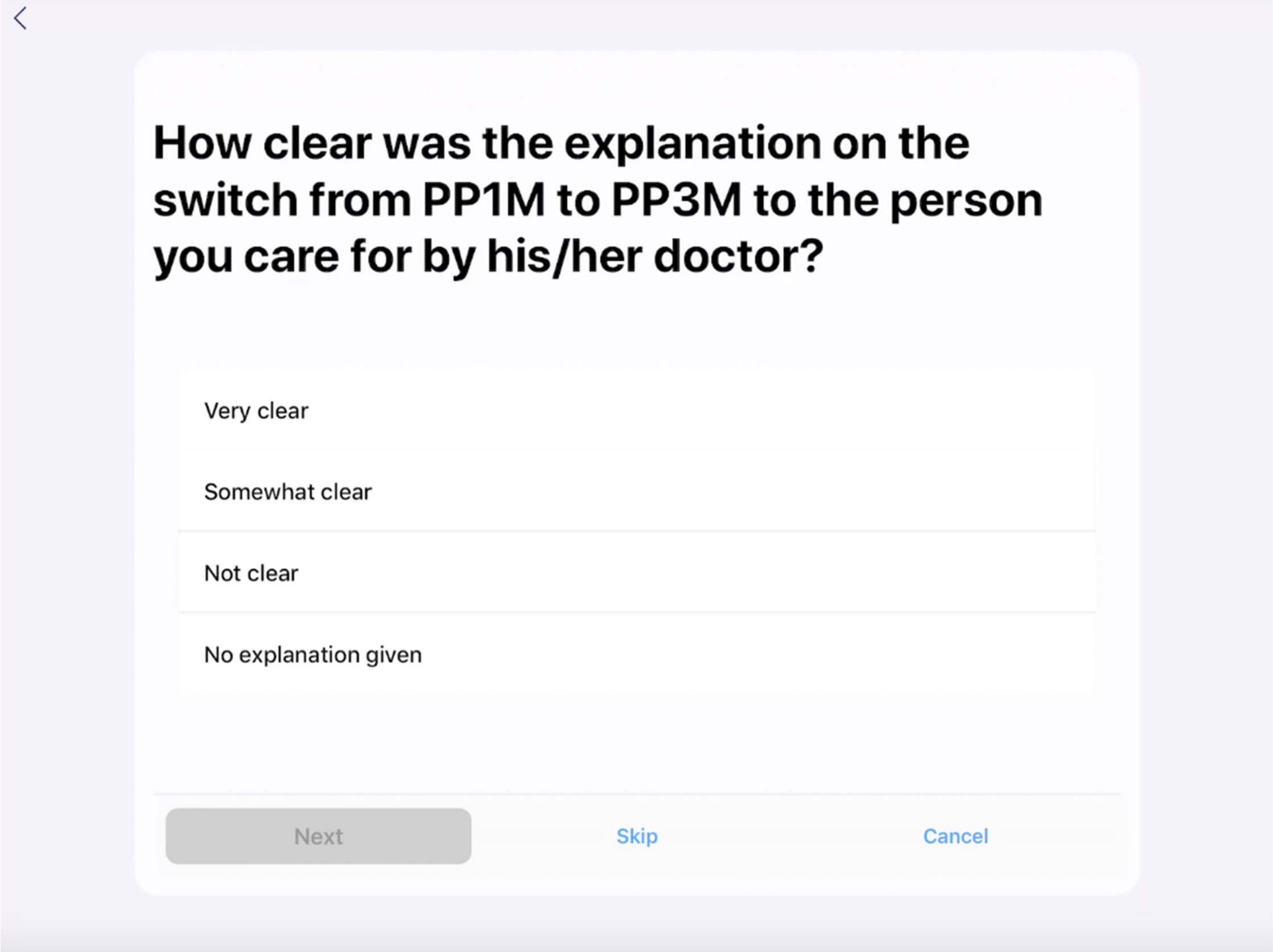
How clear was the explanation on the switch from PP1M to PP3M to the person you care for by his/her doctor?
Very clear
Somewhat clear
Not clear
No explanation given
Next
Skip
Cancel

How many times has the person you care for seen his/her nurse during the last 6 months?

Tap to answer times

Next Skip Cancel

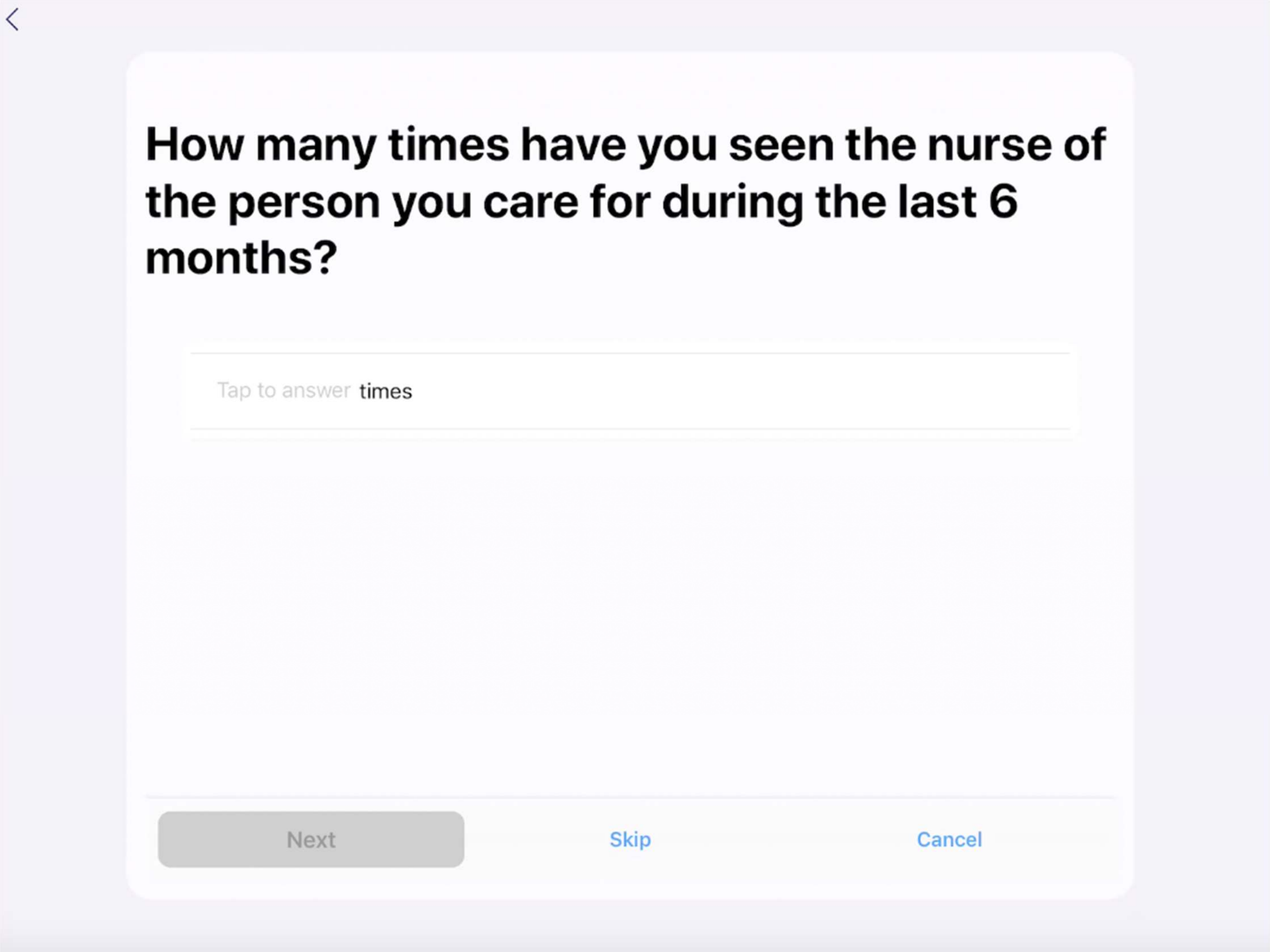
How many times have you seen the nurse of the person you care for during the last 6 months?
Tap to answer times
Next
Skip
Cancel

Has the frequency in which the person you care for sees his/her nurse changed as a result of switching to PP3M?

He/she meets more frequently

He/she meets less frequently

There is no change

Next

Skip

Cancel

How satisfied are you with the current frequency in which the person you care for sees his/her nurse?

Very Satisfied

Satisfied

Dissatisfied

Very Dissatisfied

Next Skip Cancel

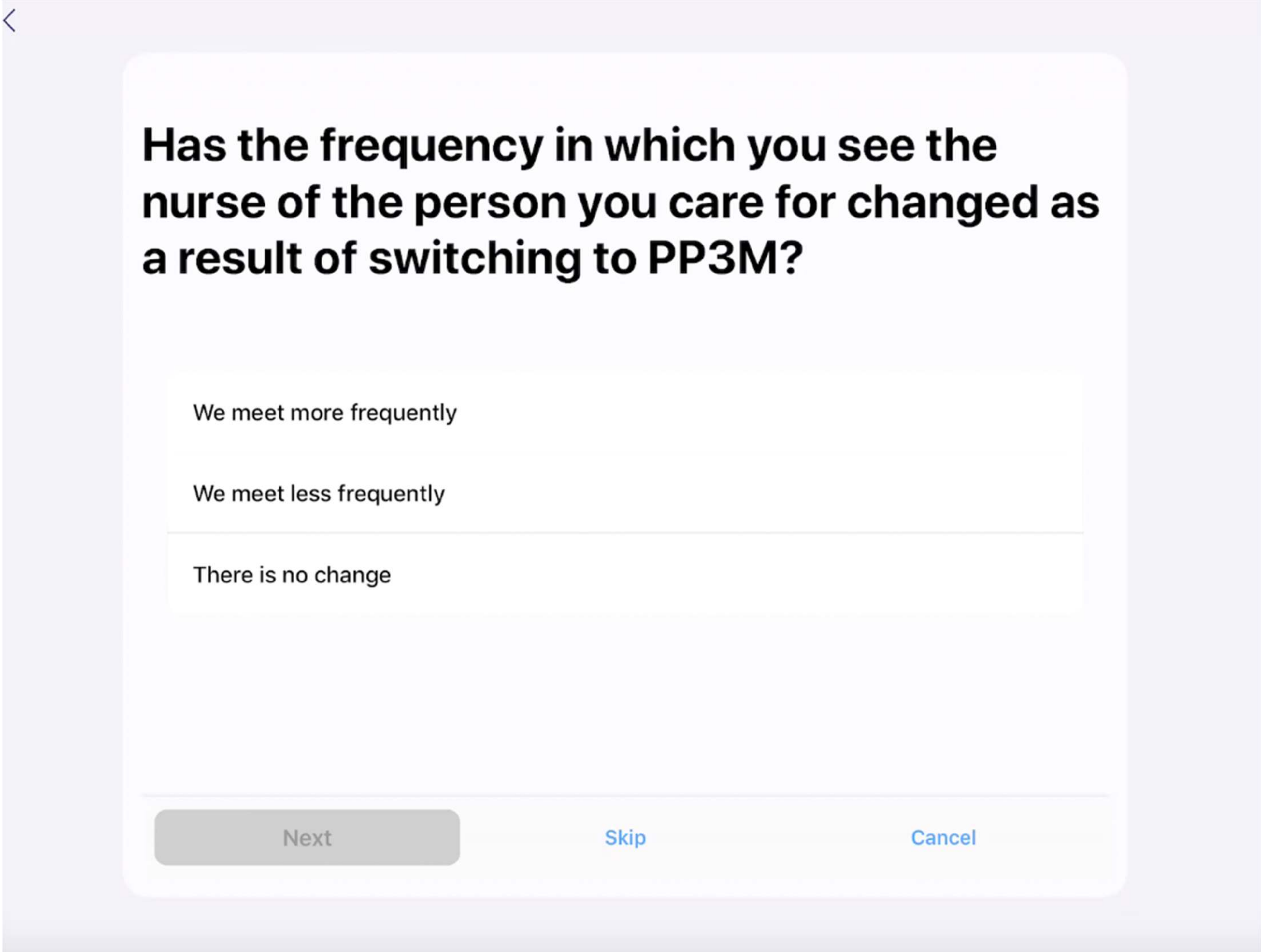
Has the frequency in which you see the nurse of the person you care for changed as a result of switching to PP3M?
We meet more frequently
We meet less frequently
There is no change
Next
Skip
Cancel

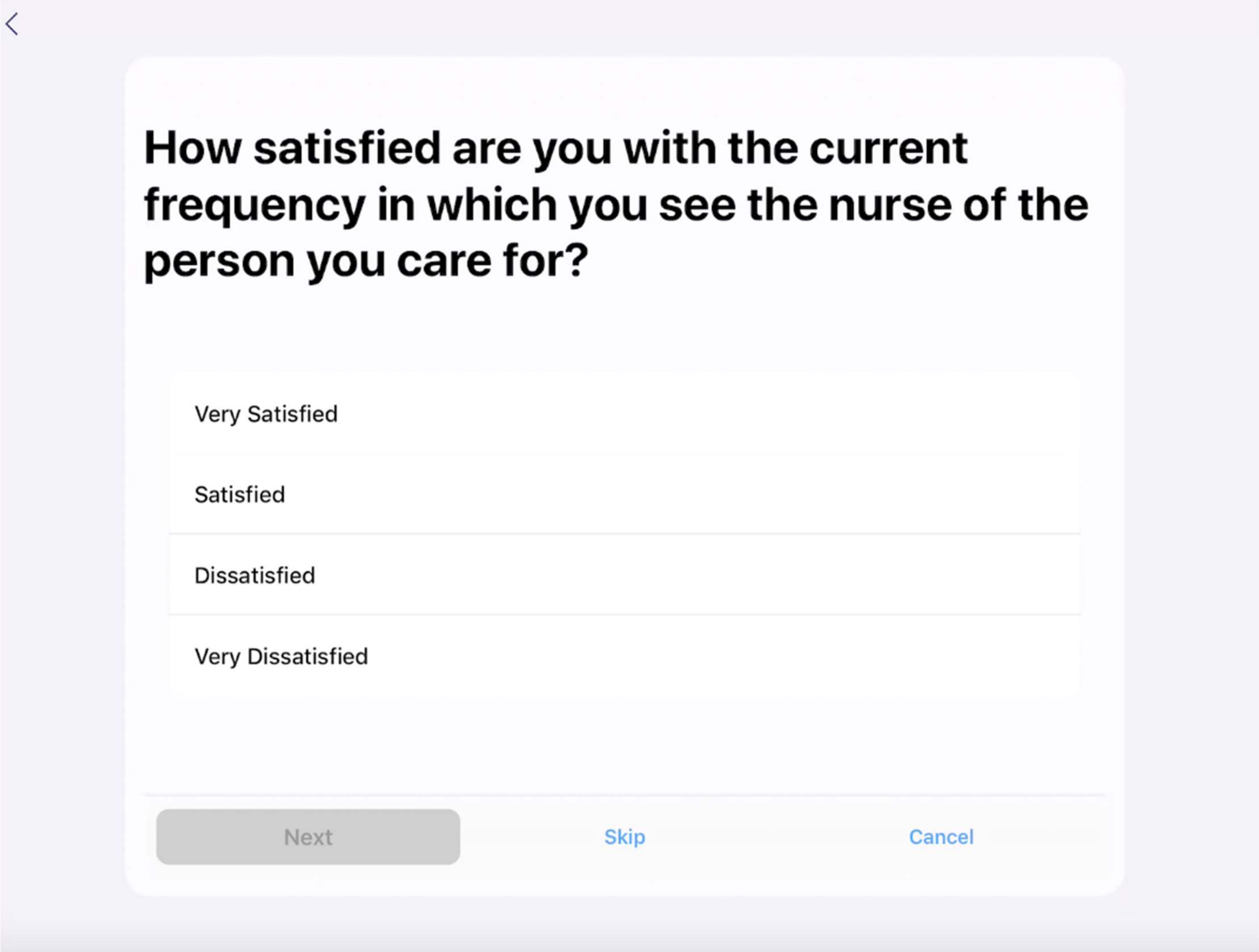
How satisfied are you with the current frequency in which you see the nurse of the person you care for?
Very Satisfied
Satisfied
Dissatisfied
Very Dissatisfied
Next
Skip
Cancel

How often would you think the person you care for needs to be seen by his/her nurse? (regardless of the injection frequency)

- More than once per month
- Monthly
- Once every three months
- Less than every three months

Next Skip Cancel

How clear was the explanation on the switch from PP1M to PP3M to the person you care for by his/her nurse?

Very clear

Somewhat clear

Not clear

No explanation given

Next   Skip   Cancel

Has the quality of your communication with the nurse of the person you care for changed as a result of switching to PP3M?

- It has improved
- It has not changed
- It has worsened

Next Skip Cancel

How much do you talk about the person you care for with his/her doctor or nurse about topics other than medication as a result of switching to PP3M?

Talk more

Talk less

Same amount

Next Skip Cancel

If "TALK MORE":

Please indicate topics that you were able to discuss more about the person you care for with the doctor and nurse as a result of switching to PP3M? (multiple answers) Scroll to view all

- His/her family relationships
- His/her work
- His/her education
- His/her external/social communication
- His/her hobbies
- His/her healthy life style guidance (e.g. alcohol, smoking, diet, physical exercise)
- His/her non medication therapy (e.g. psychotherapy)
- Other topics

Next | Skip | Cancel

To what extent were you involved in the decision to switch from PP1M to PP3M?

Highly involved

Involved to some extent

Not involved at all

Next   Skip   Cancel

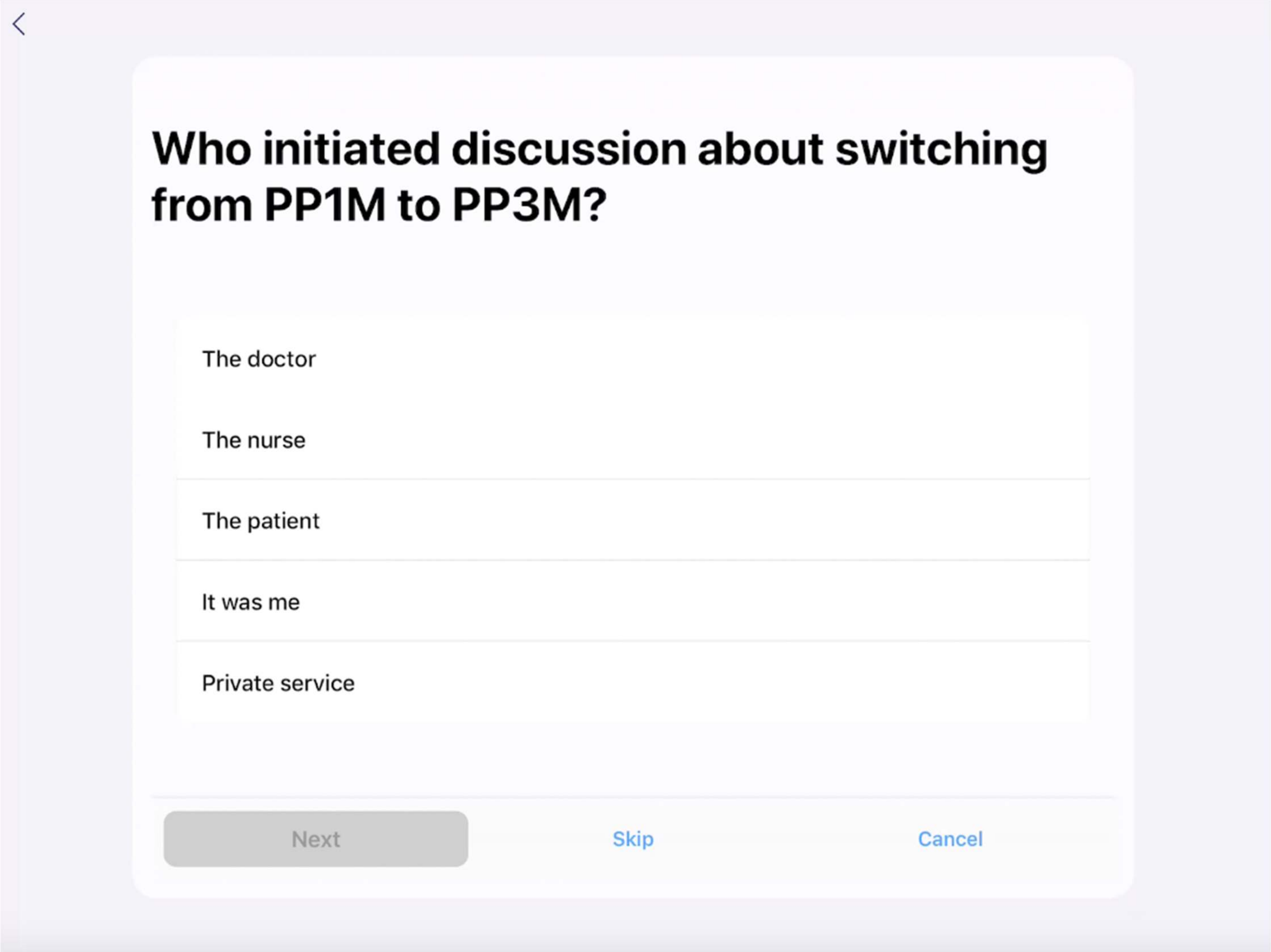
Who initiated discussion about switching from PP1M to PP3M?
The doctor
The nurse
The patient
It was me
Private service
Next
Skip
Cancel

What were the main reasons to initiate/switch their treatment to PP3M? Please check all that apply. Scroll to view all

- To live a life that is as normal as possible
- To reduce side effects
- To improve treatment efficacy
- To improve treatment adherence
- To decrease stigma
- Patient convenience
- Reduce the risk of relapse

Next | Skip | Cancel

Select MOST important reason. Scroll to view all

- To live a life that is as normal as possible
- To reduce side effects
- To improve treatment efficacy
- To improve treatment adherence
- To decrease stigma
- For his/her convenience
- Reduce the risk of relapse

Next | Skip | Cancel

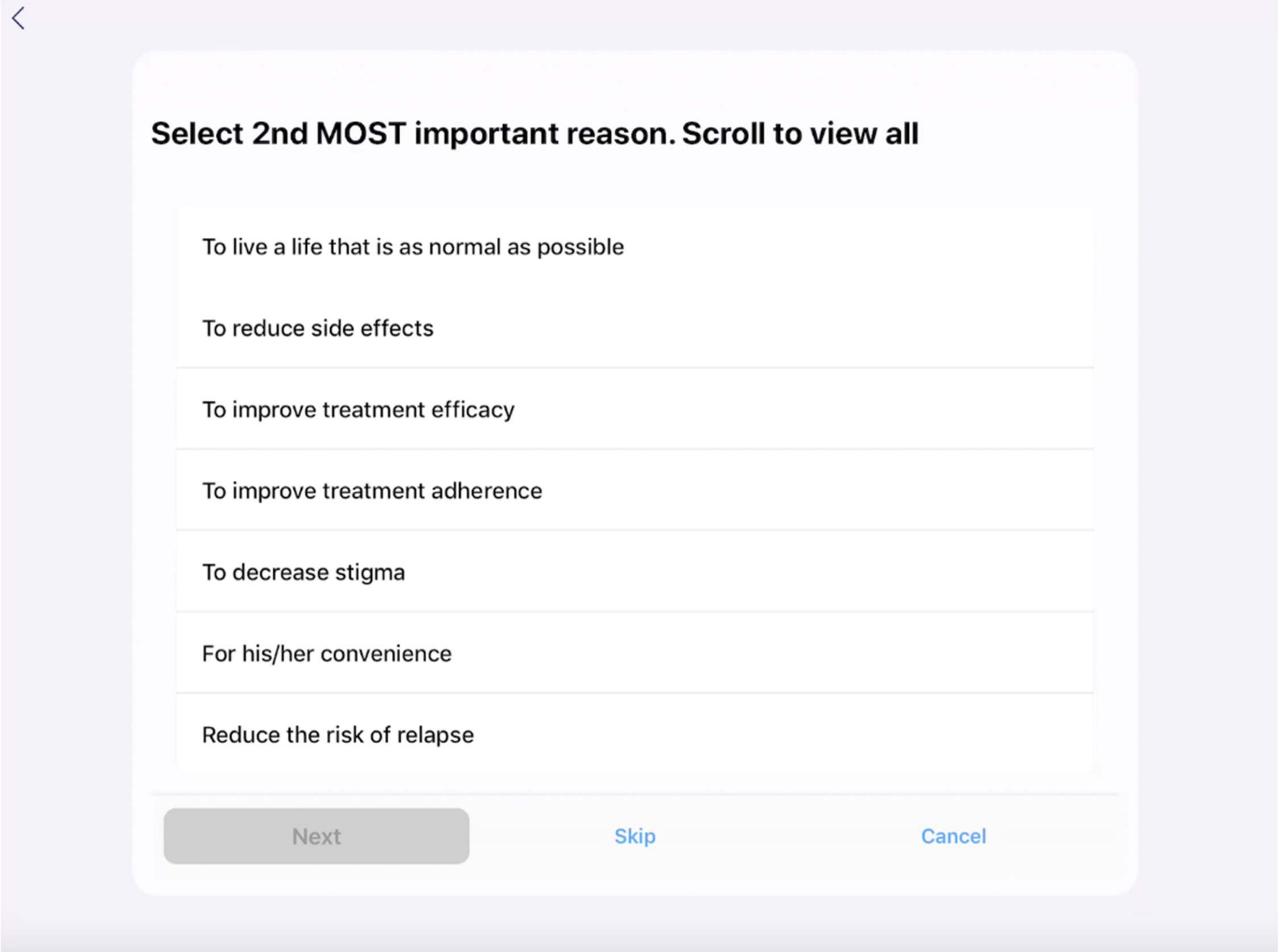
Select 2nd MOST important reason. Scroll to view all
To live a life that is as normal as possible
To reduce side effects
To improve treatment efficacy
To improve treatment adherence
To decrease stigma
For his/her convenience
Reduce the risk of relapse
Next
Skip
Cancel

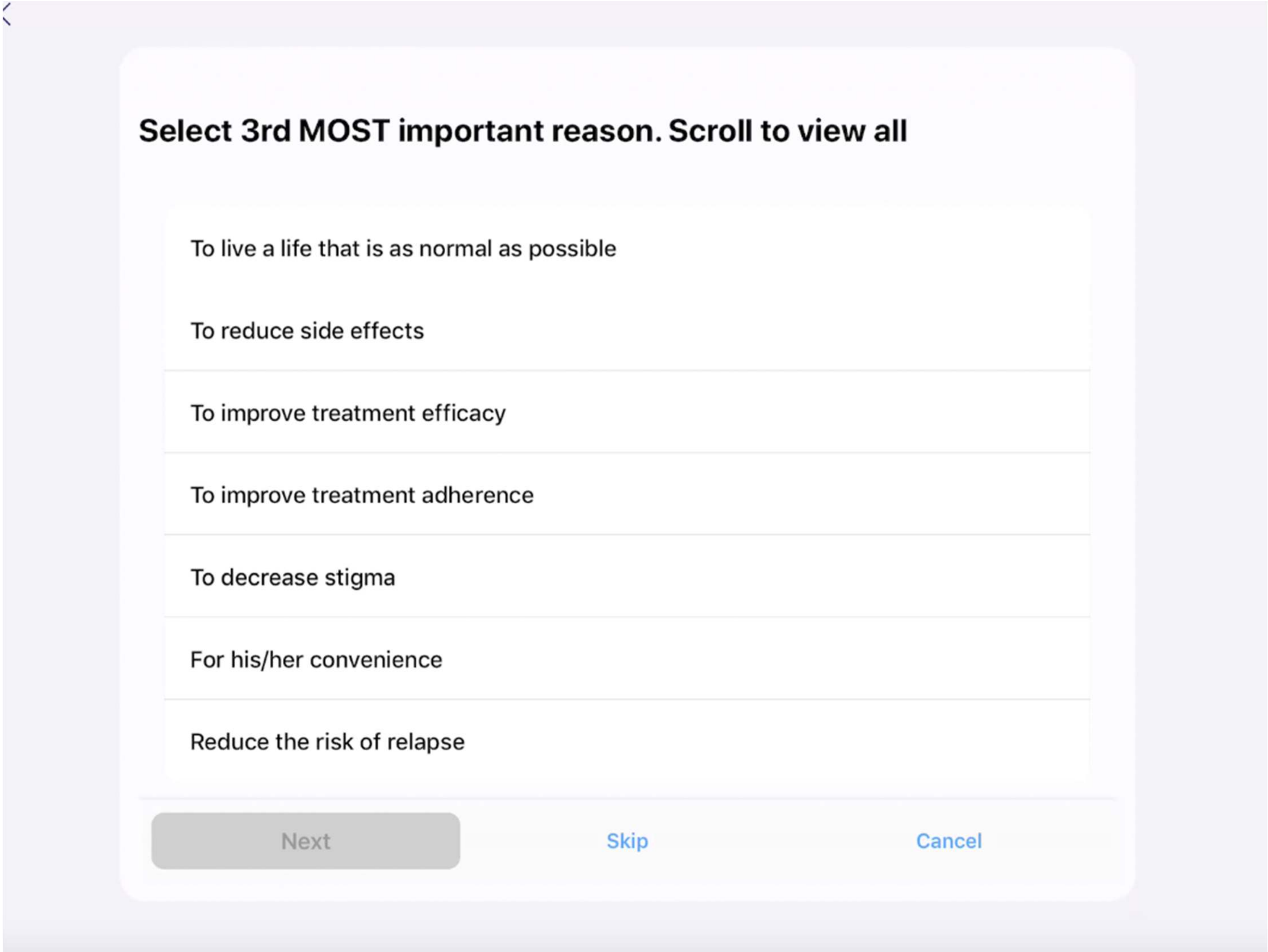

Select 3rd MOST important reason. Scroll to view all
To live a life that is as normal as possible
To reduce side effects
To improve treatment efficacy
To improve treatment adherence
To decrease stigma
For his/her convenience
Reduce the risk of relapse
Next
Skip
Cancel

As a result of switching to PP3M was there any change in activity level of the person you care for (e.g. sports / hobbies, education, work, seeing friends and/or family)?

Activity level increased

No changes

Activity level decreased

Next Skip Cancel

If "ACTIVITY LEVEL INCREASED":

Please indicate in what way the activity level of the person you care for was increased as a result of switching to PP3M? (multiple answers)

- He/she started new sporting activities/hobbies
- He/she returned to previous sporting activities/hobbies
- Started working again
- Changed job
- Spends more time in social situations

Next Skip Cancel

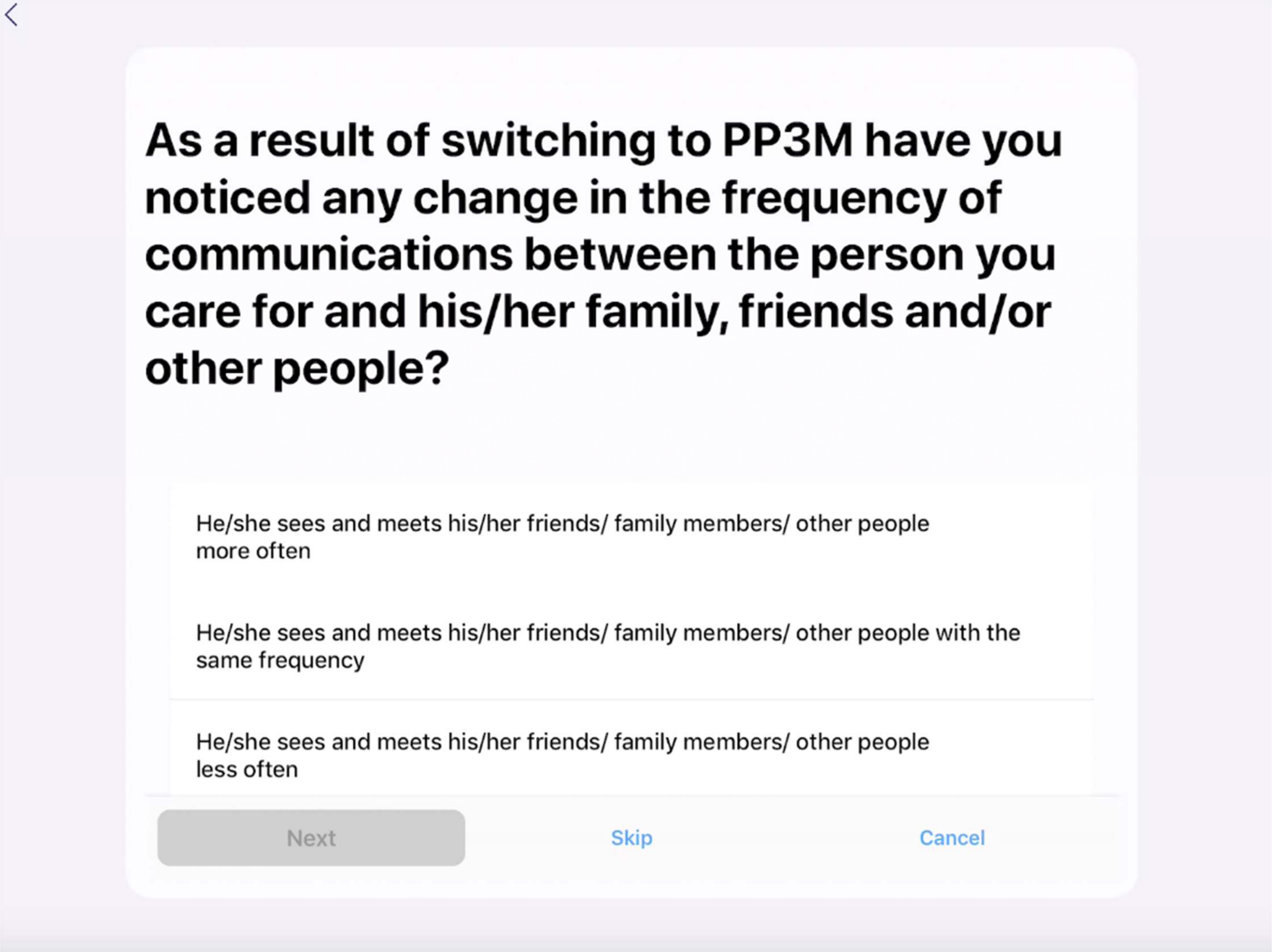
As a result of switching to PP3M have you noticed any change in the frequency of communications between the person you care for and his/her family, friends and/or other people?
He/she sees and meets his/her friends/ family members/ other people more often
He/she sees and meets his/her friends/ family members/ other people with the same frequency
He/she sees and meets his/her friends/ family members/ other people less often
Next
Skip
Cancel

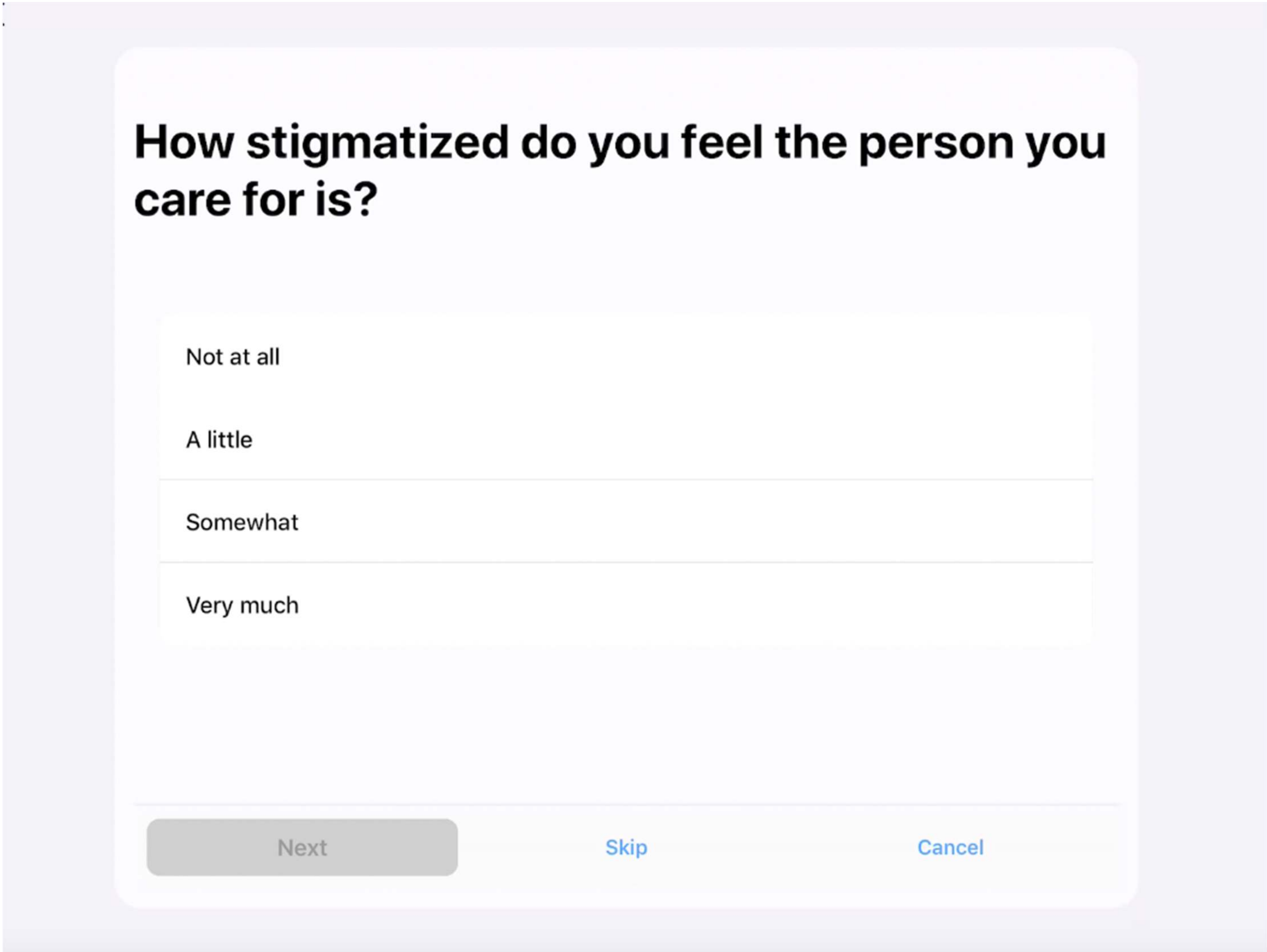
How stigmatized do you feel the person you care for is?
Not at all
A little
Somewhat
Very much
Next
Skip
Cancel

To what extent do you think that PP3M impacts on the feeling of the person you care for of being stigmatized?

No impact

Increases stigma

Decreases stigma

Next   Skip   Cancel

How much do you talk with the person you care for about topics other than medication as a result of switching to PP3M?

Talk more

Talk less

Same amount

Next   Skip   Cancel

If “TALK MORE”:

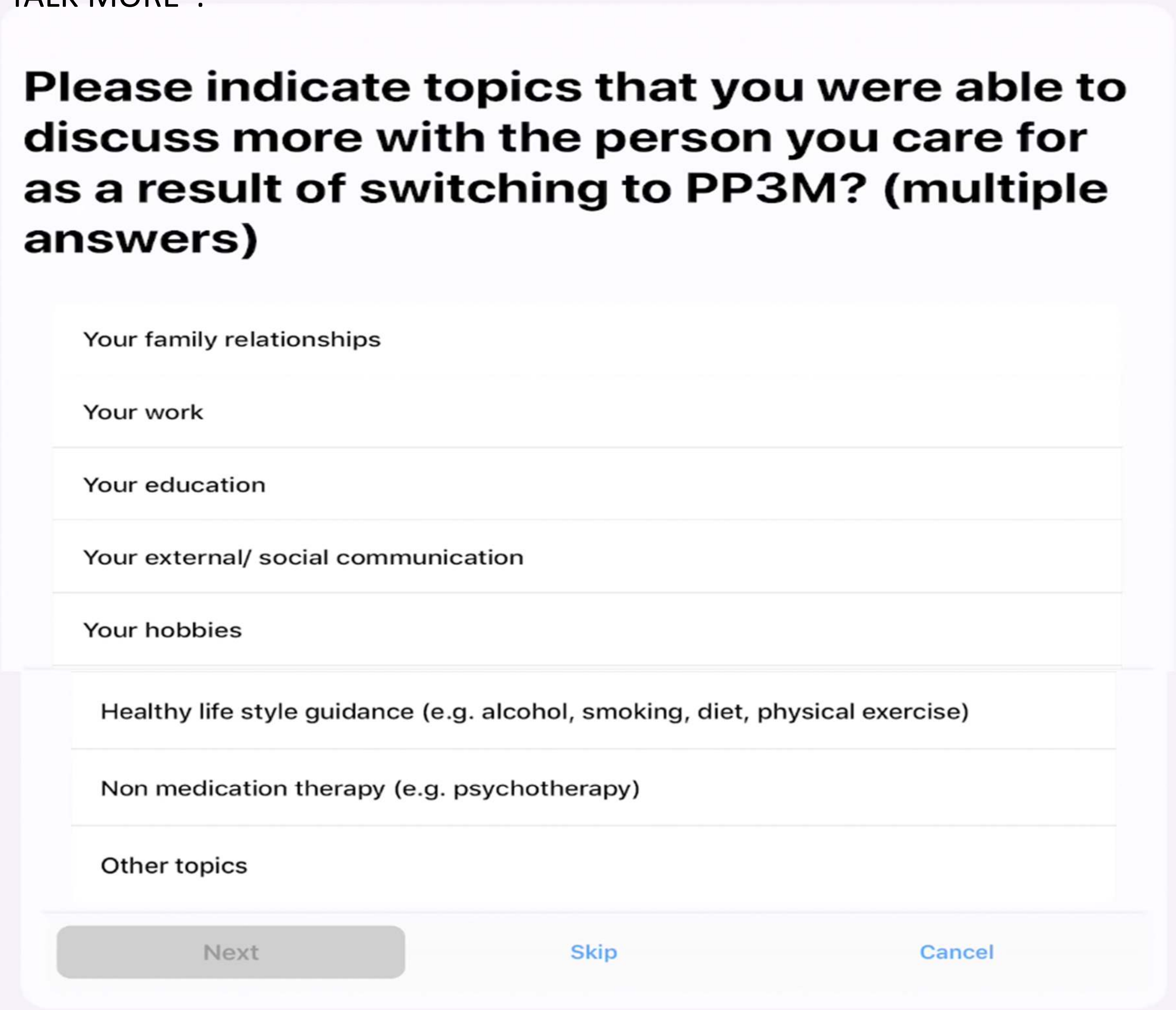
Please indicate topics that you were able to discuss more with the person you care for as a result of switching to PP3M? (multiple answers)
Your family relationships
Your work
Your education
Your external/ social communication
Your hobbies
Healthy life style guidance (e.g. alcohol, smoking, diet, physical exercise)
Non medication therapy (e.g. psychotherapy)
Other topics
Next
Skip
Cancel

Do you live with the person you care for?

Yes

No

Next

Skip

Cancel

If Not “Living with the Person you care for”:

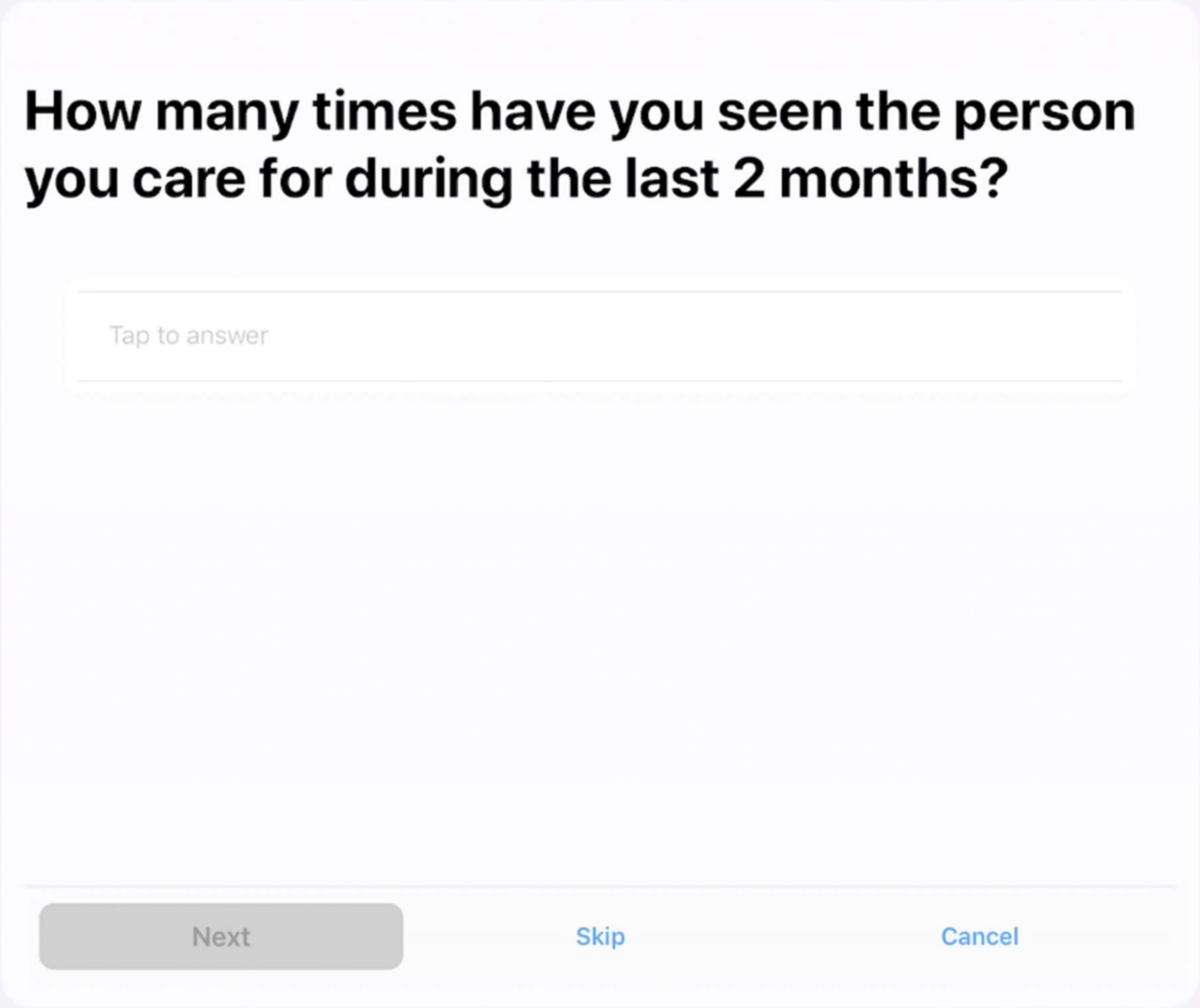

Has the frequency in which you see the person you care for changed as a result of switching to PP3M?

- We meet more frequently
- We meet less frequently
- There is no change
- Not applicable as I am living with the person I care for

Next Skip Cancel

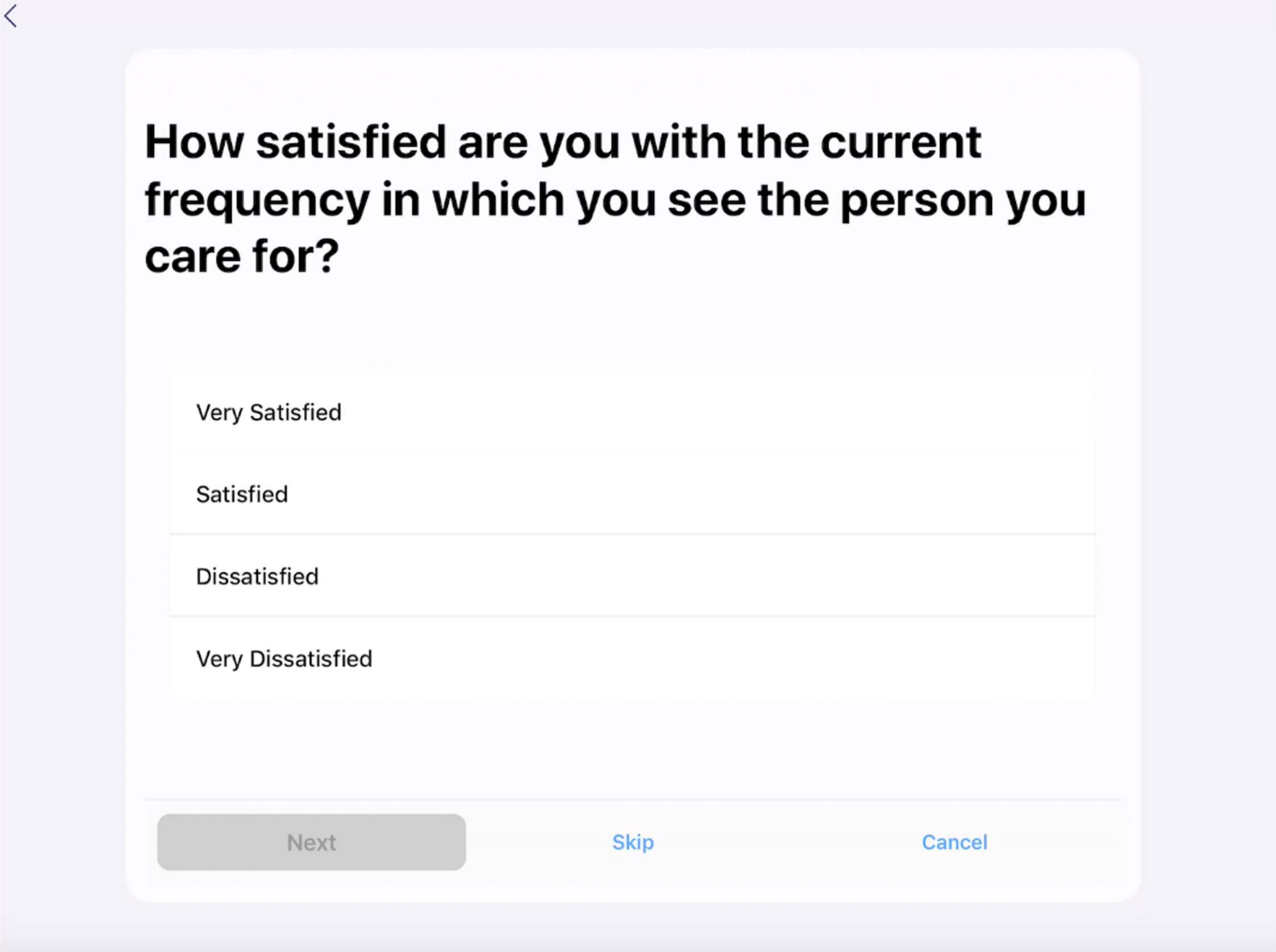
How satisfied are you with the current frequency in which you see the person you care for?
Very Satisfied
Satisfied
Dissatisfied
Very Dissatisfied
Next
Skip
Cancel

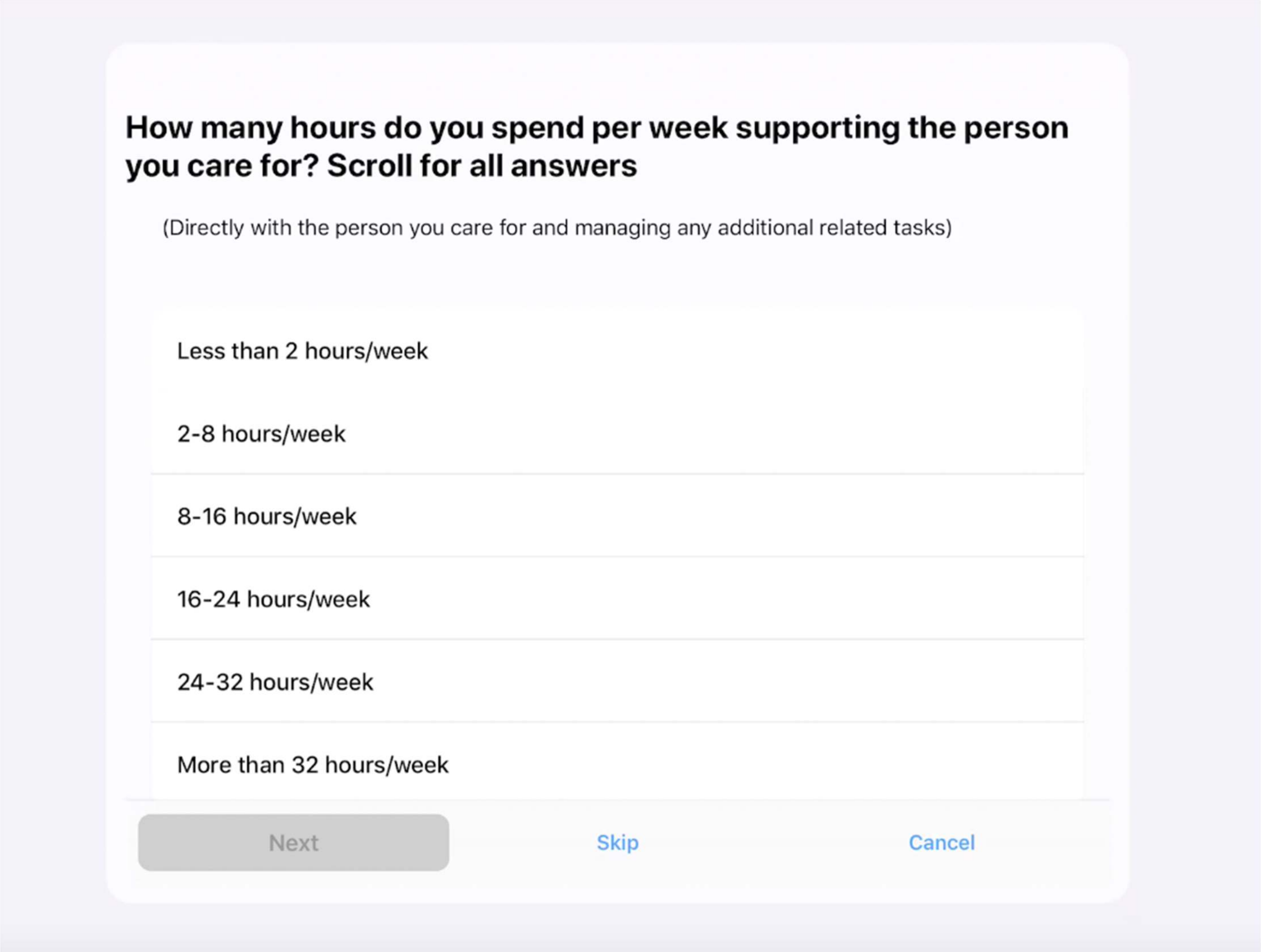
How many hours do you spend per week supporting the person you care for? Scroll for all answers
(Directly with the person you care for and managing any additional related tasks)
Less than 2 hours/week
2-8 hours/week
8-16 hours/week
16-24 hours/week
24-32 hours/week
More than 32 hours/week
Next
Skip
Cancel

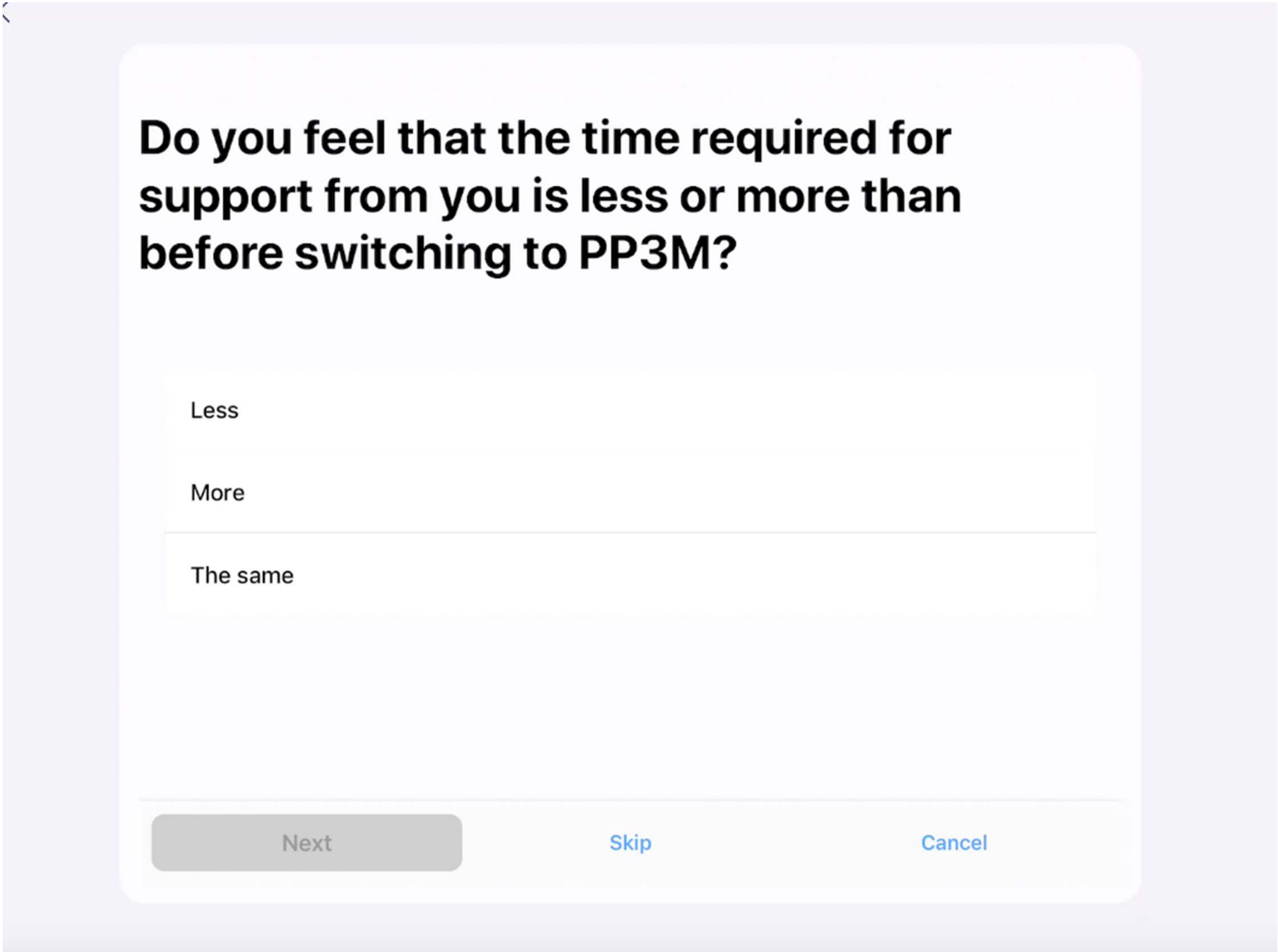
Do you feel that the time required for support from you is less or more than before switching to PP3M?
Less
More
The same
Next
Skip
Cancel

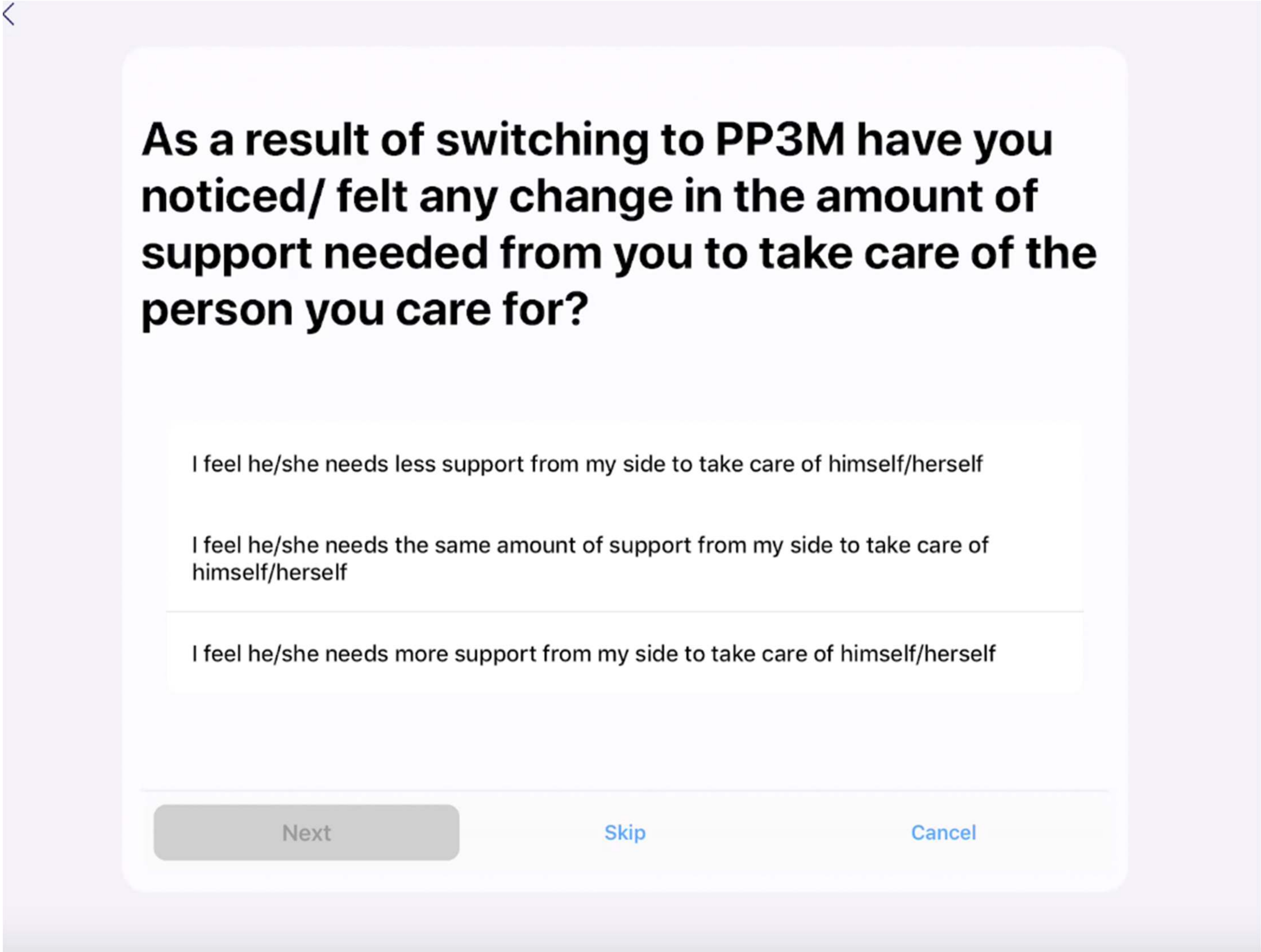
As a result of switching to PP3M have you noticed/ felt any change in the amount of support needed from you to take care of the person you care for?
I feel he/she needs less support from my side to take care of himself/herself
I feel he/she needs the same amount of support from my side to take care of himself/herself
I feel he/she needs more support from my side to take care of himself/herself
Next
Skip
Cancel

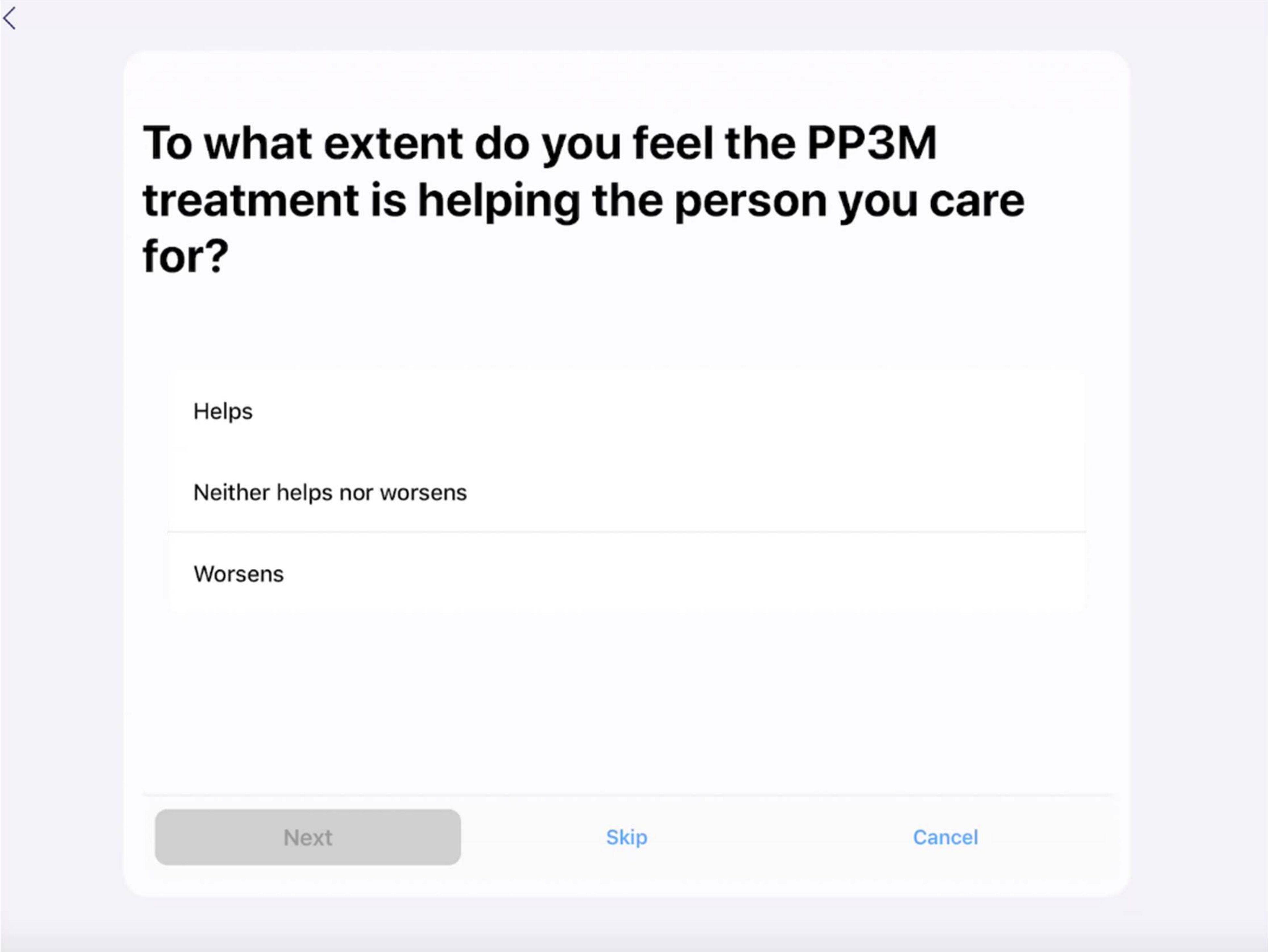
To what extent do you feel the PP3M treatment is helping the person you care for?
Helps
Neither helps nor worsens
Worsens
Next
Skip
Cancel

On the next screen, please review all of your answers. If there are any unanswered questions, please confirm that you intended not to answer those questions.

Next

Cancel

Please review your answers. Pressing “Next” will submit your answers

You can jump directly to update question by clicking on it in the list below

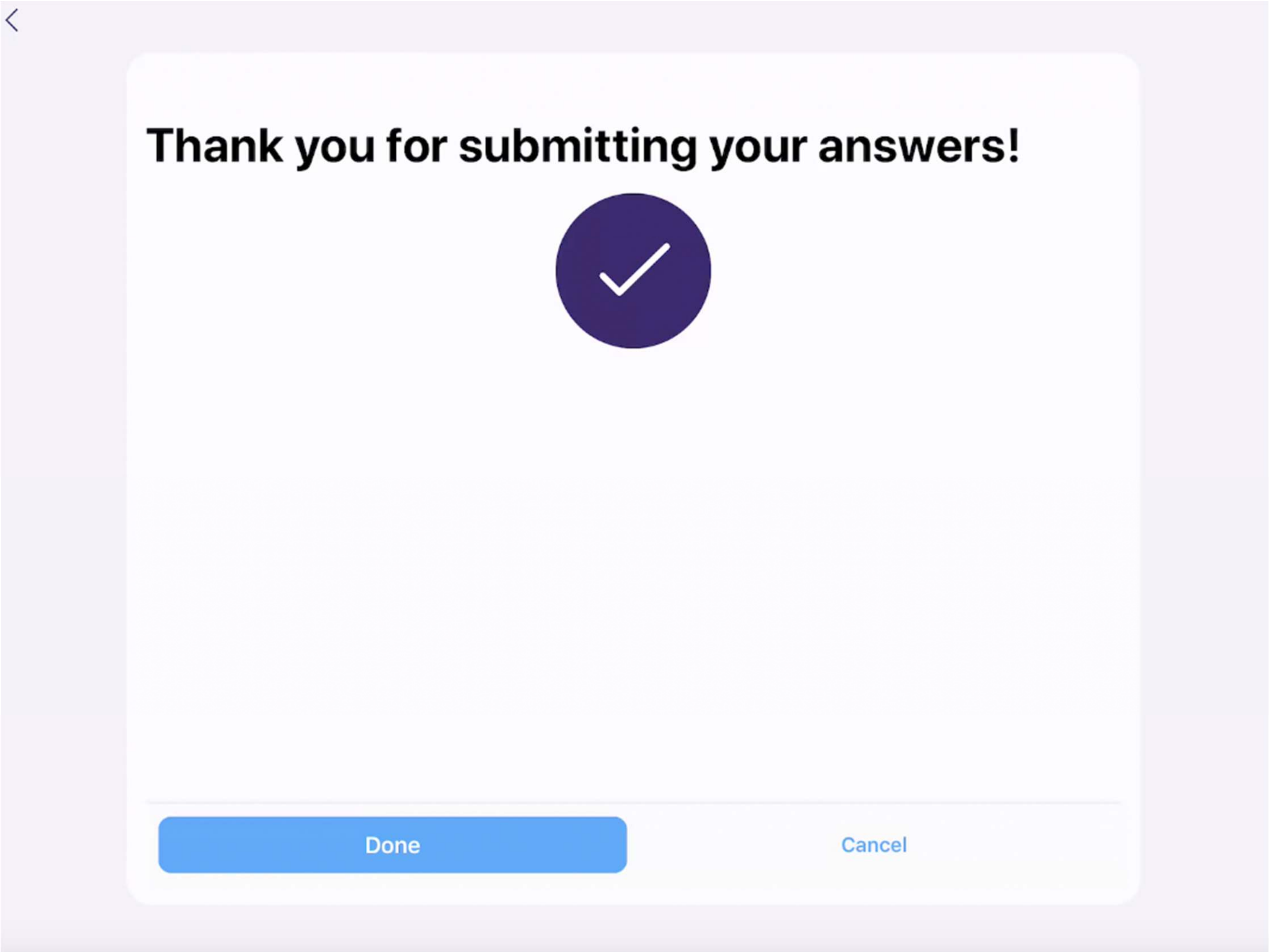
Thank you for submitting your answers!
Done
Cancel
