# Supplementary material for: The Patient, Investigator, Nurse, Carer Questionnaire (PINC-Q): a cross-sectional, retrospective, non-interventional study exploring the impact of less frequent medication administration with paliperidone palmitate 3-monthly as maintenance treatment for schizophrenia
Source: BMC Psychiatry. 2021 Jun 9;21:300. doi: 10.1186/s12888-021-03305-z (PMC8191017; doi:10.1186/s12888-021-03305-z)
Supplement: Supplementary file 9 — Additional file 9. English inclusion exclusion survey. [file 12888_2021_3305_MOESM9_ESM.pdf]

Inclusion/Exclusion Survey

Eligibility Criteria

There are 6 questions in this questionnaire that must be completed by a nurse or psychiatrist for each patient. You must complete these questions to unlock additional questionnaires for the patient, carer, nurse, or psychiatrist. If a patient does not meet any inclusion/exclusion criteria, they are not eligible to participate in this study.

Next

Cancel

The next 4 questions are inclusion criteria.

Get Started

Cancel

Be males or females aged ≥18 years

Yes

No

Next

Cancel

Have a diagnosis of schizophrenia (according to ICD-10)

Yes

No

Next

Cancel

Be currently receiving PP3M and have previously received 4 to 6 injections of PP3M

Yes

No

Next

Cancel

Have the required language skills to participate in the online questionnaire, in the opinion of the physician

Yes

No

Next

Cancel

The next 2 questions are exclusion criteria.

Next

Cancel

Has received involuntary treatment with PP3M

Yes

No

Next

Cancel

Was switched to PP3M treatment within a clinical trial

Yes

No

Next

Cancel

This patient is eligible for the study!

Done

Cancel
