# Supplementary material for: The Patient, Investigator, Nurse, Carer Questionnaire (PINC-Q): a cross-sectional, retrospective, non-interventional study exploring the impact of less frequent medication administration with paliperidone palmitate 3-monthly as maintenance treatment for schizophrenia
Source: BMC Psychiatry. 2021 Jun 9;21:300. doi: 10.1186/s12888-021-03305-z (PMC8191017; doi:10.1186/s12888-021-03305-z)
Supplement: Supplementary file 10 — Additional file 10. English enrolment task. [file 12888_2021_3305_MOESM10_ESM.pdf]

Enrolment Task

By completing this task and the following questions, you are enrolling a new subject in this study. If patient did not complete consent, or you do not wish to do this, please cancel.

Get Started

Cancel

Did the patient complete a paper consent?

Yes

No

Next

Cancel

When did the patient sign the consent form?

Please document date that patient completed consent form

September	7	2015
October	8	2016
November	9	2017
**December**	**10**	**2018**
January	11	2019
February	12	2020
March	13	2021

Next

Cancel

What is patient's gender?

Male

Female

Next

Cancel

What is the patient's age?

Tap to answer

Next

Cancel

Success! You enrolled a patient in the study and generated a subject ID for this patient

Done

Cancel
